# The effects of walking speed and mobile phone use on the walking dynamics of young adults

**DOI:** 10.1038/s41598-020-79584-5

**Published:** 2021-01-13

**Authors:** Patrick Crowley, Nicolas Vuillerme, Afshin Samani, Pascal Madeleine

**Affiliations:** 1 Sport Sciences – Performance and Technology, Department of Health Science and Technology, Aalborg University, Aalborg, Denmark; 2 University of Grenoble Alpes, AGEIS, Grenoble, France; 3 The National Research Centre for the Working Environment, Lersø Parkallé 105, Copenhagen, Denmark; 4 Institut Universitaire de France, Paris, France; 5 LabCom Telecom4Health, University of Grenoble Alpes and Orange Labs, Grenoble, France

**Keywords:** Motor control, Dynamical systems

## Abstract

Walking while using a mobile phone has been shown to affect the walking dynamics of young adults. However, this has only been investigated using treadmill walking at a fixed walking speed. In this study, the dynamics of over ground walking were investigated using lower trunk acceleration measured over 12 consecutive trials, following differing walking speed and mobile phone use instructions. Higher walking speed significantly increased the proportion of acceleration along the vertical measurement axis, while decreasing the proportion of acceleration along the anteroposterior axis (*p* < 0.001). Moreover, higher walking speed also resulted in increased sample entropy along all measurement axes (*p* < 0.05). When walking while texting, the maximum Lyapunov exponent increased along the anteroposterior and vertical measurement axes (*p* < 0.05), while sample entropy decreased significantly along the vertical axis (*p* < 0.001). Walking speed and mobile phone use both affect the walking dynamics of young adults. Walking while texting appears to produce a reduction in local dynamic stability and an increase in regularity, however, caution is required when interpreting the extent of this task effect, since walking speed also affected walking dynamics.

## Introduction

The speed at which we walk and the performance of a parallel task with a mobile phone will change how we walk^1–6^. Previous studies have established the effect of mobile phone use while walking, on the spatiotemporal parameters of walking^3,7–15^. However, to date just a few studies have investigated walking dynamics under this dual task condition^16–19^. These studies provide relevant and valuable information, but their approach to measuring the dynamics of walking are limited by two methodological aspects. Firstly, participants in these studies walked on a treadmill^16–19^. This may induce walking dynamics that differ from those observed in everyday settings involving over ground walking^20,21^. Secondly, a reduction in gait velocity appears inherent when performing a mobile phone dual task^3,7–15^. Therefore, a fixed walking speed may inadvertently impose what is actually a sub-optimal walking speed for that dual task condition. Therefore, the current study presents an alternative protocol using over ground walking and self-determined non-fixed walking speeds to more accurately reflect the dynamics of natural walking.

However, this protocol alone is not enough if traditional linear methods are subsequently used for analysis. Such methods only provide an indication of the extent of variability around a central point or mean. While some work has been done to define “optimal windows” of variability^22,23^, the potential for detecting dynamic change through linear analysis is fundamentally limited. This is because the analysis of mean change, by definition, ignores or averages over any dynamic change that may be present. Therefore, to investigate walking as a dynamic behaviour nonlinear analysis methods must be used. Dynamical systems theory (DST) states that nonlinear analysis methods the investigation of dynamic behaviour, which can be defined as the patterns of change in a system state over time^24^. In order to achieve functional healthy walking continuous adjustment in response to continuous stream of external and internal sensory input is required. Nonlinear analysis methods and DST provide a framework for understanding these adjustments and can therefore provide insight into the complexity of human movement^25,26^. A consensus on what constitutes a measure of complexity in human movement is difficult to reach. Primarily because of the inherent difficulty of establishing a ground truth for complexity. There are several definitions of complexity in the literature, and although differing in some aspects, each definition is linked through the acknowledgement that a complex system is one *“involving many interacting subsystems*^27^. Therefore, a full analysis of system complexity would require the analysis of each sub-system across multiple time scales. Since this is not always feasible, it may be more reasonable to aim for a partial analysis of system complexity through the extraction of a number of different traits representing the evolution pattern of a dynamic system over time. This is attempted in the current study through the analysis of regularity and predictability. Traits that can help to capture changes in complexity^28^. The ability to identify a system of lower complexity is relevant when investigating the effects of pathological conditions and aging^28–30^.

Acceleration of the lower trunk is an appropriate measure of walking dynamics, since it reflects both lower limb movement and the stabilization of the head, trunk and arms^31–33^. The root mean square ratio (RMSratio) was used to provide an indication of the effect of walking speed instruction and dual task conditions on the variability of acceleration along each measurement axis. The root mean square of acceleration is a more conventional measure of variability in trunk acceleration^34^, but a ratio of acceleration in each axial measurement direction to the modulus of acceleration is preferred because it facilitates comparisons across walking speeds^35^. It is reported alongside the sample entropy of trunk acceleration (SaEn) and the maximum Lyapunov exponent (MaxLyE) derived from the acceleration time series. SaEn is interpreted to determine how the regularity of walking—considered as a trait of complexity in the current study—is affected by the change in walking conditions. Further the MaxLyE is used to estimate the ability to respond to small local perturbations (*local* dynamic stability) while walking^36^, introduced by the noise interfering with movement execution.

Our study protocol used over ground walking, structured text messaging and semi-structured mobile phone conversations, to address the lack of scientific protocols aimed at capturing the natural walking dynamics under dual task conditions in a non-laboratory setting. As such, this study is the first to provide empirical evidence and insight for researchers, clinicians and practitioners, on the effects of walking speed and mobile phone use on the natural walking dynamics of young adults in ecologically valid setting using nonlinear analysis methods. The hypothesis was that change in walking dynamics would be affected by walking speed instruction and mobile phone use, when compared with the single task (walking only) condition. In particular, that local dynamic stability would decrease at a sub-optimal walking speed^37^ (induced by the fast walking speed instruction) and with mobile phone use due to the effect of the attentional demands produced by concurrent tasks^38,39^.

## Results

### Variability of trunk acceleration

**Figure 1:**
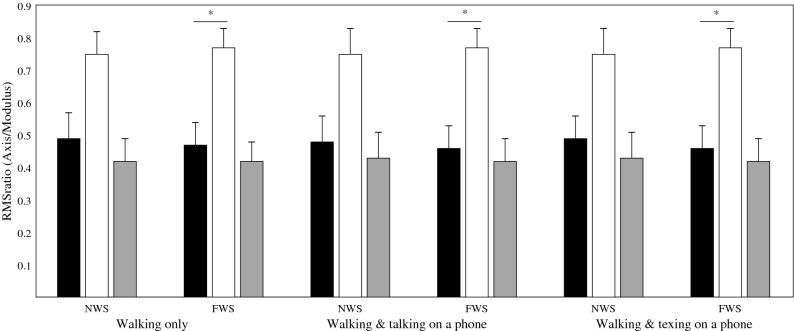
Mean root mean square ratio values when walking at self-selected normal (NWS) and fast walking speed (FWS), while texting on a mobile phone, talking, or performing no concurrent task (walking only). Black bars = anteroposterior axis, White bars = vertical axis, Grey bars = mediolateral axis. ‘*’ denotes a statistically significant effect of walking speed instruction.

Multivariate analysis indicated a significant effect of walking speed instruction (Λ = 0.37, F_3, 17_ = 9.6, *p* = 0.001) on the RMSratio (Table 1). Subsequent univariate analyses confirmed this effect along the VT (*p* < 0.001) and AP (*p* < 0.001) measurement axes (Table 1). Based on the average values presented in Fig. 1, the ratio of AP acceleration to the modulus of acceleration decreased from a normal to fast walking speed across all conditions while the ratio increased along the VT measurement axis.

**Table 1:** Multivariate and univariate analysis results following a 2 × 3 RMANOVA assessing the influence of walking speed instruction and task on the root-mean-square ratio (RMSratio).

	Task	Walking speed	Walking speed × task
**Multivariate**			
	F_6,72_ = 0.3	F_3,17_ = 9.6	F_6,72_ = 1.18
	(*p* = *0.95*)	**(p < 0.001)**	(*p* = *0.33*)
	Λ = 0.96	Λ = 0.37	Λ = 0.83

	Task (no multivariate effect)	Walking speed	Walking speed × task (no multivariate effect)
**Univariate**			
AP	F_1.4,27.1_ = 0.5	F_1,19_ = 18.4	F_2,38_ = 1.1
	(*p* = 0.53)	**(p < 0.001)**	(*p* = 0.35)
VT	F_2,38_ = 0.01	F_1,19_ = 23.2	F_2,38_ = 0.8
	(*p* = 0.99)	**(p < 0.001)**	(*p* = 0.46)
ML	F_2,38_ = 0.1	F_1,19_ = 0.1	F_2,38_ = 2.4
	(*p* = 0.87)	(*p* = 0.33)	(*p* = 0.11)

‘Λ’ denotes Wilk’s lambda; ‘F’ denotes the F-statistic (extent of the difference between group means).

‘*p*’ denotes the statistical significance; the threshold for significance was set at *p* < 0.05.

*ML* mediolateral measurement axis,* VT* vertical measurement axis,* AP* anteroposterior measurement axis.

### Nonlinear outcome measures

**Figure 2:**
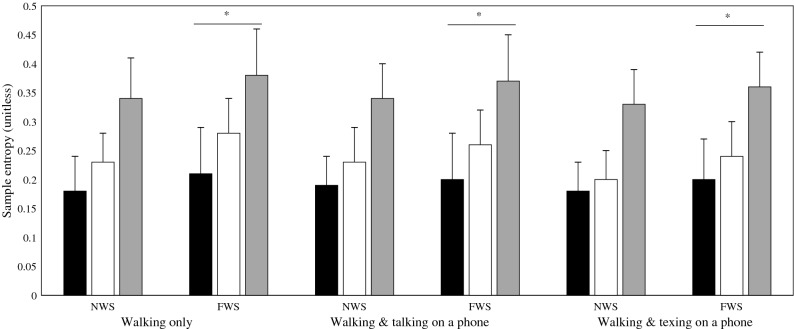
Mean sample entropy (SaEn) values when walking at self-selected normal (NWS) and fast walking speed (FWS) while texting on a mobile phone, talking, or performing no concurrent task (walking only). Black bars = anteroposterior axis, White bars = vertical axis, Grey bars = mediolateral axis. ‘*’ denotes a statistically significant effect of walking speed instruction. ‘t’ denotes a statistically significant effect of task.

Multivariate analysis indicated a significant influence of both walking speed instruction (Λ = 0.26, F_3, 17_ = 16.24, *p* < 0.001) and task (Λ = 0.36, F_6, 72_ = 8.00, *p* < 0.001) on SaEn (Table 2). Univariate analyses confirmed a significant effect of speed along the AP (*p* < 0.05), VT (*p* < 0.001) and ML (*p* < 0.001) measurement axes. On average SaEn values increased from normal to fast walking speed across all conditions. When comparing task conditions, significant effects were observed in the VT (*p* < 0.001) direction (Table 2). Post hoc analysis using a Bonferroni adjustment indicated that this significant decrease was only evident when walking while texting was compared with walking in the single task condition (Fig. 2).

**Table 2:** Multivariate and univariate analysis results following a 2 × 3 RMANOVA assessing the influence of walking speed instruction and task on entropy (SaEn).

	Task	Walking speed	Walking speed × task
**Multivariate**			
	F_6,72_ = 8.00	F_3,17_ = 16.24	F_6,72_ = 0.63
	**(p < 0.001)**	**(p < 0.001)**	(*p* = 0.70)
	Λ = 0.36	Λ = 0.26	Λ = 0.90

	Task	Walking speed	Walking speed × task (no multivariate effect)
**Univariate**			
AP	F_2,38_ = 1.32	F_1,19_ = 8.93	F_2,38_ = 1.21
	(*p* = 0.28)	**(p** **< 0.05)**	(*p* = 0.31)
VT	F_2,38_ = 30.44	F_1,19_ = 45.16	F_2,38_ = 1.32
	**(p < 0.001)**	**(p < 0.001)**	(*p* = 0.28)
ML	F_1.5,37.1_ = 1.53	F_1,19_ = 28.80	F_1.5,28.9_ = 0.22
	(*p* = 0.23)	**(p < 0.001)**	(*p* = 0.74)

‘Λ’ denotes Wilk’s lambda; ‘F’ denotes the F-statistic (extent of the difference between group means).

‘*p*’ denotes the statistical significance; the threshold for significance was set at *p* < 0.05.

*ML* mediolateral measurement axis, *VT* vertical measurement axis, *AP* anteroposterior measurement axis.

**Figure 3:**
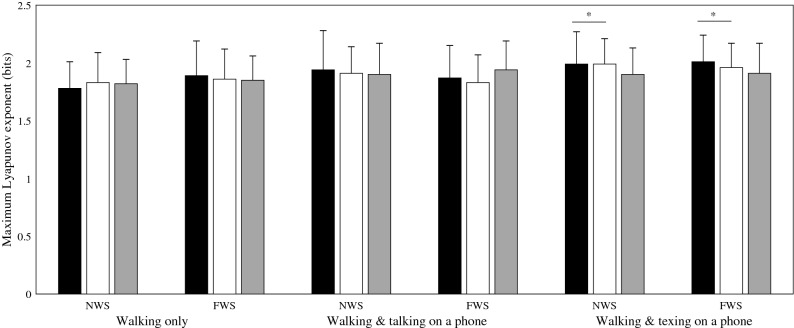
Mean maximum Lyapunov exponents when walking at self-selected normal (NWS) and fast walking speed (FWS), while texting on a mobile phone, talking, or performing no concurrent task (walking only). Black bars = anteroposterior axis, White bars = vertical axis, Grey bars = mediolateral axis. ‘*’ denotes a statistically significant effect of walking speed instruction. ‘t’ denotes a statistically significant effect of task.

Multivariate analysis indicated a significant effect of task (Λ = 0.61, F_6, 72_ = 3.41, *p* < 0.05) on MaxLyE (Table 3). This significant effect was confirmed by univariate analysis (Table 3), where an increase in MaxLyE was observed along the VT and AP measurement axes. Post hoc analysis using a Bonferroni adjustment indicated that these significant increases were only evident when walking while texting was compared with walking in the single task (walking only) condition (Fig. 3).

**Table 3:** Multivariate and univariate analysis results following a 2 × 3 RMANOVA assessing the influence of walking speed instruction and task on the maximum Lyapunov exponent (MaxLyE).

	Task	Walking speed	Walking speed × task
**Multivariate**			
	F_6,72_ = 3.41	F_3,17_ = 0.64	F_6,72_ = 0.71
	**(p < 0.05)**	(*p* = 0.60)	(*p* = 0.64)
	Λ = 0.61	Λ = 0.90	Λ = 0.89

	Task	Walking speed (no multivariate effect)	Walking speed × task (no multivariate effect)
**Univariate**			
AP	F_2,38_ = 6.47	F_1,19_ = 0.14	F_2,38_ = 1.43
	**(p < 0.05)**	(*p* = 0.72)	(*p* = 0.25)
VT	F_2,38_ = 4.63	F_1,19_ = 0.24	F_2,38_ = 0.73
	**(p < 0.05)**	(*p* = 0.63)	(*p* = 0.49)
ML	F_2,38_ = 3.22	F_1,19_ = 0.40	F_2,38_ = 0.24
	(*p* = 0.05)	(*p* = 0.54)	(*p* = 0.79)

‘Λ’ denotes Wilk’s lambda; ‘F’ denotes the F-statistic (extent of the difference between group means).

‘*p*’ denotes the statistical significance; the threshold for significance was set at *p* < 0.05.

*ML* mediolateral measurement axis, *VT* vertical measurement axis, *AP* anteroposterior measurement axis.

### Walking speed

**Figure 4:**
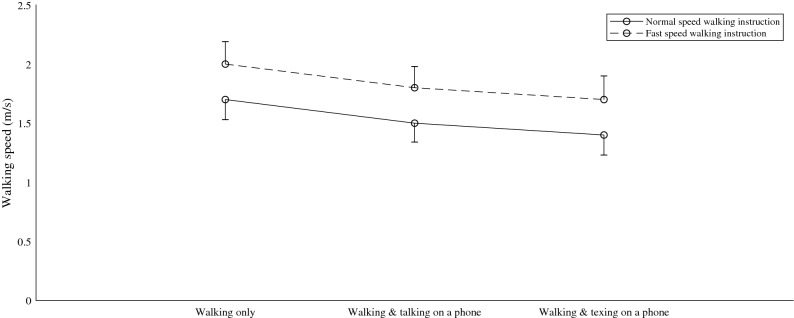
Mean walking speed with standard deviation for each task condition, following normal and fast walking speed instruction.

Multivariate analysis indicated a significant effect of both walking speed instruction (Λ = 0.18, F_1,19_ = 85.22, *p* < 0.001) and task (Λ = 0.20, F_2,18_ = 35.99, *p* < 0.001) on walking speed (Table 4). The interaction at a multivariate level between task and walking speed instruction was marginally below the level of statistical significance (Λ = 0.72, F_2, 18_ = 3.44, *p* = 0.054). Univariate analysis confirmed the effect of task (F_2, 38_ = 52.95, *p* < 0.001) and walking speed instruction (F_1, 19_ = 85.22, *p* < 0.001), but also indicated a significant interaction effect between the two (F_2, 38_ = 3.66, *p* < 0.05). Post hoc analysis, using a Bonferroni adjustment showed significant differences in walking speed between each task condition. The trend in walking speed across task conditions can be seen in Fig. 4, where the dashed line represents the walking speed following fast walking speed instruction.

**Table 4:** Mean walking speed for each walking condition and the coefficient of variation (CV) value. Values are presented as Mean (± 1 standard deviation).

	Walking only		Walking and talking on a phone		Walking and texting on a phone	
	NWS	FWS	NWS	FWS	NWS	FWS
Walking speed (m/s)	1.65 (0.17)	2.00 (0.19)	1.54 (0.16)	1.81 (0.18)	1.42 (0.18)	1.68 (0.20)
CV of walking speed	10.2	9.5	10.6	9.8	12.2	11.9

*NWS* self-selected normal walking speed, *FWS* self-selected fast walking speed.

CV = (Standard deviation/mean)*100.

## Discussion

In this study, the dynamics of over ground walking among young, healthy adults were investigated. Walking was measured under three different mobile phone use conditions, performed at two different walking speeds. Our findings suggest that both walking speed and mobile phone dual task affected walking dynamics. At a self-selected fast walking speed, these effects were evident in an increase in the sample entropy of trunk acceleration and a shift in the ratio of trunk acceleration in favor of the VT measurement axis. Further, with the addition of a texting task while walking local dynamic stability reduced and sample entropy decreased.

Concerning walking speed, the observed shift in the ratio between axial acceleration and the modulus, from the AP measurement axis, in favor of the VT measurement axis is somewhat unexpected. Previously a shift in the RMSratio in the ML measurement axis was reported^35^, whereas no significant shift in the ratio of acceleration along this axis was observed in the current study. With regard to changes in sample entropy, our results are similar to those previously observed in the stride parameters of young adults walking at different walking speeds^6^.

Concerning the effects of mobile phone use, our findings are less surprising but still somewhat in contrast with the findings of previous studies. Firstly, the effect of mobile phone dual tasks were not the same. Whereas the dynamics of walking while talking on a mobile phone showed little difference from walking dynamics in the single task condition, there were differences observed when walking while texting. Under this condition, a significant increase in MaxLyE value along the VT measurement axis was observed; suggesting decreased local dynamic stability. Similar findings of decreased local dynamic stability have previously been reported in a study similar to the current study, but making use of a treadmill^16^. However, the increases in that study were along the AP and ML measurement axes^16^. Moreover, a further two comparable treadmill-based studies reported no changes in dynamic stability^17,18^. Clearly, a definitive consensus is difficult to achieve and a number of potential explanations for these discrepancies must be considered.

The first possible explanation is that walking while texting is detrimental to the walking dynamics of young adults causing reduced local dynamic stability. An increased MaxLyE value is indicative of a less dynamically stable system. For example, an increase in MaxLyE values has been repeatedly observed among fall-prone older adults—a group typically considered to represent poor walking ability^40–42^. However, compared with a relative difference in MaxLyE values between a healthy and a patient group with a known neurological condition impairing gait (a difference of approximately 21%)^43^, the differences in MaxLyE values between single task walking and walking while texting was relatively small (14% and 9% at normal and fast walking speeds respectively). Therefore, it would seem that while the present changes observed when walking while texting change the dynamics relative to normal walking, they might not be sufficient to have practical implications in terms of local dynamic stability.

A second possible explanation acknowledges the possibility of an inverse relationship between sample entropy and maximum Lyapunov exponent, whereby higher entropy may lead to a lower exponent value as suggested by recent work investigating the effects of noisy time series^44^. For our results, this would mean that some of the observed task-effect could be explained by the changes in walking speed. Moreover, while the current study reports an increased MaxLyE along the VT measurement axis, it has been stated that the ML axis that is the “best candidate” to detect detrimental changes in local dynamic stability^43^.There was no significant change along this axis. In addition, MaxLyE values in the VT have been shown to increase with walking speed without any additional dual task^37^, but it must also be mentioned that methodological differences make direct comparisons difficult. A difficulty highlighted in a recent review on the MaxLyE^45^.

Our interpretation is that both explanations are likely to play a role in our findings. It seems unlikely that texting while walking would not cause distraction, but it may be that young adults have the cognitive acuity to handle the competition for attentional resources and the changed demand on visual processing systems, with only slight adjustments to their dynamics of walking^19,38,39,46^. However, further research is required to confirm the link between biomechanical variables, neural processes and perception.

A strength of our study is a protocol design aimed at capturing the natural walking dynamics. However, it is not without limitations. Firstly, the choice of over ground walking led to a shorter time series for analysis (30 strides). This leads to theoretical difficulty in calculating the MaxLyE and achieving a stable estimate. Fifty or 150 strides have been recommended to achieve a stable estimate^44,47^. The current study presents a protocol design that can be considered ecologically valid meaning that longer time series would not represent real texting while walking task. Thus, the stability of the MaxLyE estimate was investigated and it was found to be relatively stable at 30 strides (Supplementary information). Secondly, over ground walking at a self-determined, non-fixed, walking speeds allows for changes in walking speed during each walking trial. This makes it difficult to distinguish the effect of walking speed from the other concurrent task like texting. To address this, walking speed across task conditions was compared and showed similar trends in walking speed across conditions (Table 4 and Fig. 4). Thirdly, while over ground walking was chosen to reflect the natural walking dynamics, it does introduce the element of changing sensory input arising from the walking environment (e.g. changing visual input as the participant progresses along the corridor). However, since environmental conditions were largely identical across conditions in the current study, this is also judged to have had little effect on the reported results. A further final limitation of the current study lies in the choice of algorithm for the detection of the maximum Lyapunov exponents, as each algorithm has its own inherent limitations.

The Wolf algorithm was chosen in the current study, as it appears to be more appropriate for short experimental data sets; however, it is susceptible to noise and relies on the selection of initial parameters^48^. To account for these limitations, a low pass filter was applied to reduce the noise level as suggested^44,49,50^. In addition, the selected initial parameters are reported in the methods section to facilitate comparison in future studies. The embedding dimension and time delay were fixed (average of the individual embedding dimension and time delay of each series) in line with the recommendations of recent findings^51,52^.

In conclusion, our findings suggest that both walking speed and mobile phone use affect the walking dynamics of over ground walking among young, healthy adults. Walking speed had a clear effect on the variability of trunk acceleration as quantified by RMSratio, and the complexity or regularity of trunk accelerations as quantified by sample entropy. The mobile phone dual task of walking while talking had no significant effect on walking dynamics, whereas texting while walking increased the variability of trunk acceleration, decreased the regularity or complexity of trunk accelerations, and decreased the local dynamic stability of walking as quantified by the maximum Lyapunov exponent. Despite these changes in walking dynamics, the current sample of young, healthy adults, were able to cope with the mobile phone dual task conditions. Future research in this area may consider more collaboration across research groups aimed at implementing the next-generation tools proposed by Ioannidis^53^, such as prospective meta-analyses, as an effective and efficient approach to achieving consensus on these important questions. Nonlinear analysis methods need to be supported by further evidence and research so that their practical implications and implementation can be realized.

## Methods

### Participants

Twenty healthy young adults (11 males, 9 females; age: 27 ± 5.5 yrs. (mean ± 1 standard deviation); height: 174 ± 7.7 cm and body mass: 71 ± 10.6 kg) participated. A sub-sample of the present participants (nine out of 20) has previously been used to investigate the changes in spatiotemporal stride parameters based on shoe-worn accelerometers^9^, however, in the current study trunk worn accelerometers, not used elsewhere, are used. Participants were required to have had possession of their current mobile phone for longer than one month and engage in regular use (at least once every hour). All participants provided written informed consent as per the ethics committee of the North Denmark Region (LBK nr. 1083) guidelines and the declaration of Helsinki. This study was carried out following approval from the secretariat for the Scientific Ethics Committee in the North Denmark Region.

### Experimental protocol

Participants repeated six conditions consisting of self-selected normal and fast speed over ground walking, while texting on a mobile phone, talking, or performing no concurrent task. As such, each participant completed 12 walking trials in total along an approximately 80-m long and 1.6 m wide indoor office corridor. Walking trials were performed after working hours and under well-lit conditions. The participant’s walking speed was self-determined following simple instruction to walk at a normal or fast walking speed (e.g. “this time I would like you to walk at a normal speed”). The sequence of conditions followed a randomized partial counterbalance design and were without specific task prioritization. This is because young adults seem to exhibit a prioritization-dependent increase or decrease gait speed and texting accuracy^54^. A tri-axial accelerometer Physilog (Gait Up SA, Lausanne, Switzerland) was attached to the skin at the level of L4/L5 spinous process to record trunk accelerations during each trial. Raw accelerations files (.bin) were downloaded using the Research Toolkit v1.5.0 (Gait Up SA, Lausanne, Switzerland) and subsequently imported to Matlab R2018b (MathWorks, Natick, MA).

### Accelerometer data pre-processing

Data was sampled at 200 Hz. A correction procedure described by Moe-Nilsson (1998) ^55^—consisting of decomposing acceleration signals into the static gravity and dynamic acceleration components—removed the confounding effects of both the gravity measurement and inevitable accelerometer misalignment^55^.

### Stride detection

Thirty strides were located in the mid-section of the recorded signal using the ‘peakfinder’ Matlab function with a minimum peak distance of 50 samples for fast walking speed trials and 75 samples for normal walking speed trials. Upward and downward peak detection threshold was set as:$Threshold=rms\left(VT\right)-0.05$, where VT is the acceleration along the vertical measurement axis. To facilitate a more accurate peak detection a 5th order zero-phase digital band pass filter [0.5–10 Hz] was applied. This filter was used for stride detection only. A single stride was the period between consecutive heel strikes of the same limb (i.e., signified by every second acceleration peak in the VT signal, counting from the end of the first stride)^56^.

### Calculation of walking speed

Walking speed was calculated as the time taken to cover the 80-m walking distance (i.e. for the entire trial and not just the 30-stride mid-section) and reported as meters per second.

### Calculation of the root-mean-square ratio

The RMSratio describes the change in the relative magnitude of acceleration along each measurement axes while walking, reflecting the variability in acceleration. Since the magnitude of acceleration will be higher at increasing walking speeds^57,58^, comparison between trials at different walking speeds are confounded without the use of a ratio. Therefore, we chose to calculate the RMSratio over the traditional root-mean-square, as it is robust to differences in walking speeds^35^. RMSratio was calculated for trunk accelerations along the AP, VT, and ML measurement axes. Calculation followed two steps, (1) the calculation of the total vector magnitude [Eq. (1) and (2)] calculation of the RMSratio (Eq. 2):

1 $${T}_{RMS}=\sqrt{{RMS}_{AP}^{2}+{RMS}_{VT}^{2}+{RMS}_{ML}^{2}}$$

where T_RMS_ is the RMS total vector magnitude.

2 $${RMS}_{ratio}={RMS}_{d}/{T}_{RMS}$$

where RMS_ratio_ is the ratio between RMS of acceleration (m/s^2^) in each direction and the RMS of total vector magnitude and *‘d’* indicates the direction of acceleration.

### Nonlinear metrics

Takens’ theorem states that it is possible to construct characteristically identical copies of a dynamic system using a number of time-delayed copies of the trajectory evolution of a single point in that system^59^. Using this construction, it is then possible to analysis the patterns of system behaviour. Since it has been suggested that the Wolf algorithm is particularly susceptible to the effects of noise, we applied a zero phase 5^th^ order low pass Butterworth filter, with a cut off frequency of 50 Hz. All calculations were performed in Matlab R2019b (The MathWorks, Natick, MA). SaEn and MaxLyE calculations were completed with the help of the University of Nebraska at Omaha Biomechanics Nonlinear Analysis Toolbox (UNO Biomechanics 2019, https://www.mathworks.com/matlabcentral/fileexchange/71907-uno-biomechanics-nonlinear-analysis-toolbox).

### Calculation of sample entropy

SaEn was calculated for all walking trials as an indicator or trait of complexity using the method described by Richman & Moorman^60^ and implemented by others^61,62^. A lower value of SaEn (unit-less) signifies a greater regularity of a time series or a trait of lower complexity^27,60^. For the analysis, *m* = 2 and *r* = 0.3*standard deviation.

### Calculation of the maximum Lyapunov exponent

We calculated the MaxLyE for each participant under all conditions. An increase in the MaxLyE indicates a decrease in local dynamic stability^45^. MaxLyE (bits) was calculated using the Wolf algorithm^49^. This algorithm uses the average divergence of nearby trajectories from a single reference trajectory, providing the largest MaxLyE representing the largest rate of divergence^45^. Since walking at different speeds produces time series of different length, which can influence the MaxLyE estimation^63^, all trials were resampled to uniform length 3000 data points without enforcing a uniform number of samples per stride. The average of mutual information analysis was used to determine the appropriate time delay (τ) at which sufficient new information about the evolution of the system was apparent and we assessed the appropriate number of embedding dimensions (dE) by identifying false nearest neighbours using global false nearest neighbour analysis. τ and dE were calculated individually for each time series, however to facilitate comparison between trials, fixed τ and dE values were used for MaxLyE calculation^51^. Fixed τ and dE values were derived as the overall average of the individual trials τ and dE values (τ = 12 and dE = 7). R-tolerance and A-tolerance were set at 15 and 2 respectively, while the evolutions were set between 0.5 and 1.5 cycles (evolve iteration = 200)^49^. Since the calculation was over just 30 strides, producing a relatively short time series, a sensitivity analysis was performed to verify the consistency of the estimate. This sensitivity analysis consisted of plotting the MaxLyE estimate at each evolution of the algorithm. The plots resulting from this analysis can be found in the Supplementary Information.

### Statistical analysis

We used Q–Q plot and Kolmogorov–Smirnov tests to assess the distribution of data. A 2 × 3 within-subject repeated-measures analysis of variance (2 × 3 RMANOVA) assessed the influence of walking speed (normal and fast), task (single task, dual task of texting on a mobile phone, dual task of talking on a mobile phone), and interactions between the walking speed and task on RMSratio, SaEn, and MaxLyE. We report both the Wilk’s Lambda (Λ), where lower values indicate a larger effect contribution and the test-statistic indicating the F-distribution. For walking speed, the influence of *walking speed instruction* and task, and interactions between instruction and task, were investigated. In the case of a significant finding at the multivariate level, analysis at a univariate level followed to investigate each of the outcomes individually. Finally, post-hoc analyses were conducted, using a Bonferroni adjustment for multiple comparison, to assess pair-wise comparisons between the three task conditions. All statistical analysis was done using SPSS (IBM Statistics Data Editor V24). *p* < 0.05 was considered significant.

## Supplementary Information

Supplementary Information.

Supplementary Information.

## Data Availability

The results of sensitivity analyses have been included as supplementary information. Data can be made available upon reasonable request.
