# Supplementary material for: The effects of walking speed and mobile phone use on the walking dynamics of young adults
Source: Sci Rep. 2021 Jan 13;11:1237. doi: 10.1038/s41598-020-79584-5 (PMC7806980; doi:10.1038/s41598-020-79584-5)
Supplement: Supplementary file 1 — Supplementary Information. [file 41598_2020_79584_MOESM1_ESM.docx]

**The effects of walking speed and mobile phone use on the gait dynamics of young adults**

Patrick Crowley^1,2,3,*^, Nicolas Vuillerme ^1,2,4,5^, Afshin Samani ^1^ & Pascal Madeleine ^1^

^1^ Sport Sciences – Performance and Technology, Dept. of Health Science and Technology, Aalborg University, Aalborg, Denmark

^2^ Univ. Grenoble Alpes, AGEIS, Grenoble, France

^3^ The National Research Centre for the Working Environment, Copenhagen, Denmark

^4^ Institut Universitaire de France, Paris, France

^5^ LabCom Telecom4Health, Univ. Grenoble Alpes & Orange Labs, Grenoble, France

^*^ Correspondence to: Patrick Crowley, the National Research Centre for the Working Environment, Lersø Parkallé 105, Copenhagen, Denmark. pjc@nfa.dk

**Supplementary Information**

This supplementary information is provide in the format of .png files portraying the maximum Lyapunov exponent estimate for each evolution defined in the Wolf algorithm (Wolf et al. 1985).

Each .png file contains a three tiles for the anteroposterior (AP), vertical (V) and mediolateral (ML) measurement axes (from top to bottom).

Evolution iterations are plotted on the X-axis of each graph and the maximum Lyapunov exponent estimate is plotted on the Y-axis.

The labels at each iteration provide the absolute difference (in bits) between the current iteration (n) and the previous estimate (n-1).

NB: Note the general the consistency of the Maximum Lyapunov after approximately three evolution iterations.

Each participant completed 12 walking trials, as presented here. Participant ID numbers are only assigned for the purpose of this supplementary information.

Reference: Wolf, A., Swift, J. B., Swinney, H. L., & Vastano, J. A. Determining Lyapunov exponents from a time series. *Physicia*. **16(3),** 285-317 (1985).
