# Supplementary figures and images for: The effects of walking speed and mobile phone use on the walking dynamics of young adults (part 2 of 2)
Source: Sci Rep. 2021 Jan 13;11:1237. doi: 10.1038/s41598-020-79584-5 (PMC7806980; doi:10.1038/s41598-020-79584-5)

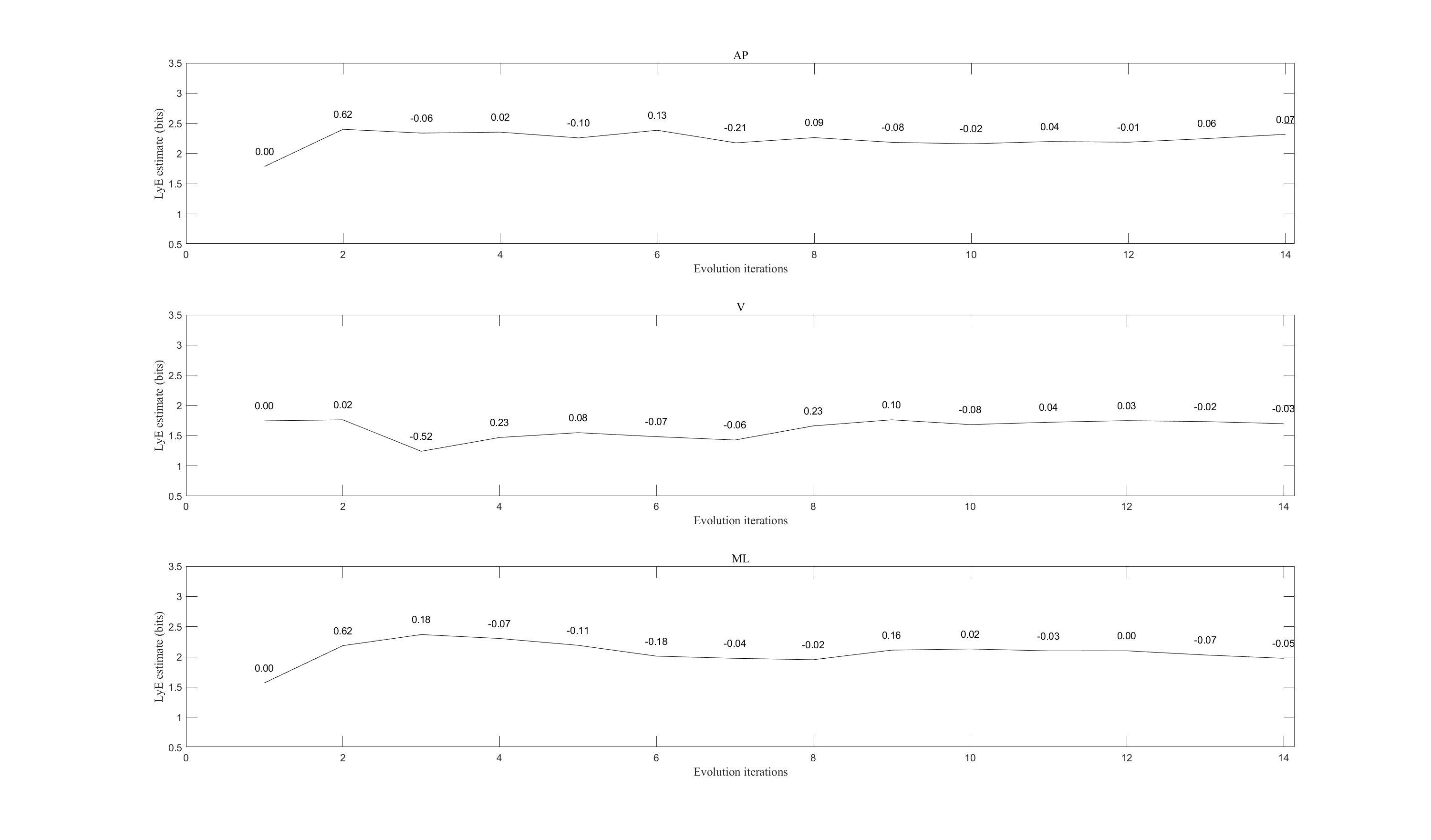

Supplement: Supplementary file 2 — Supplementary Information. [file 41598_2020_79584_MOESM2_ESM.zip › Participant17_trial2.png]

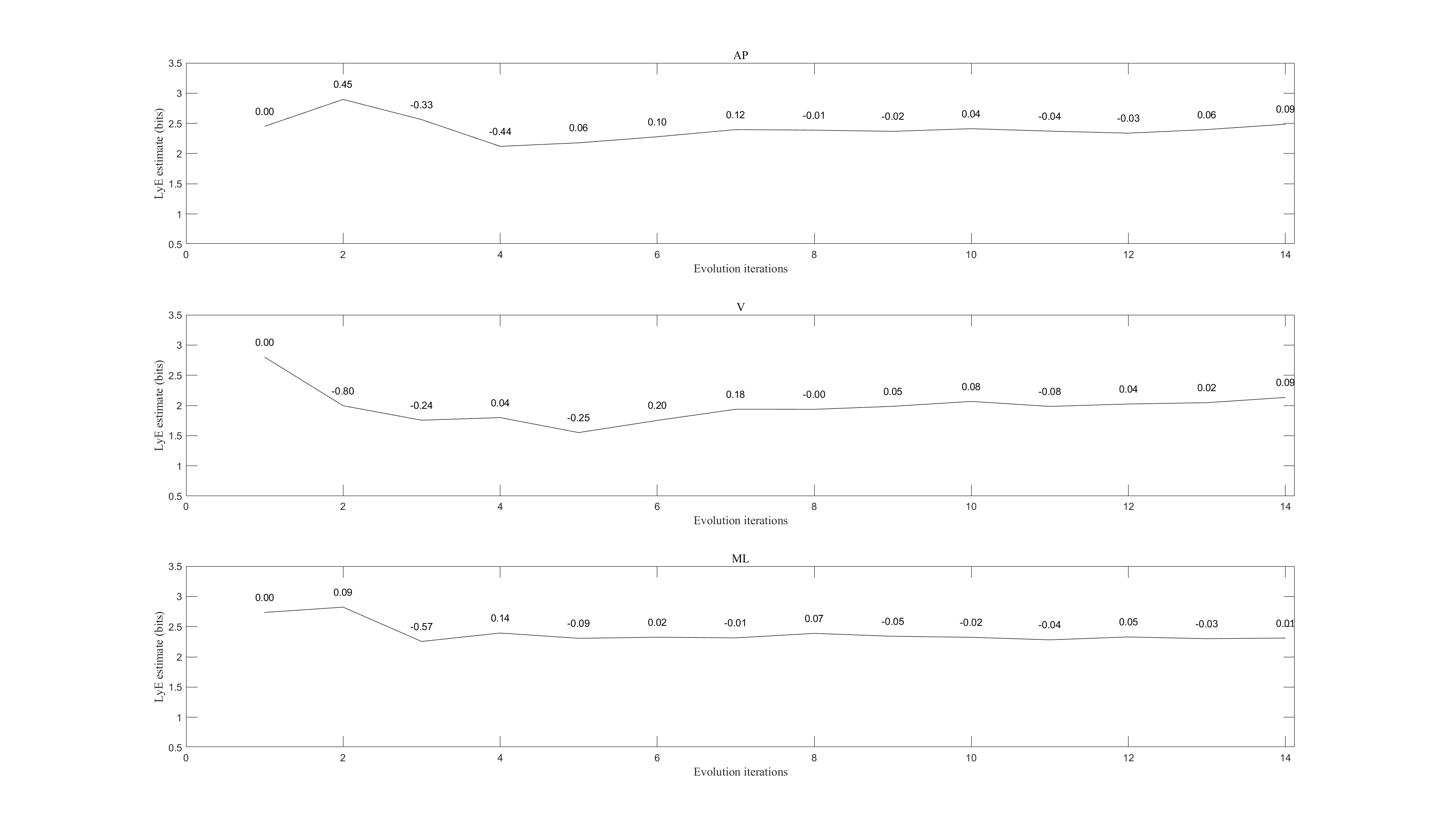

Supplement: Supplementary file 2 — Supplementary Information. [file 41598_2020_79584_MOESM2_ESM.zip › Participant17_trial3.png]

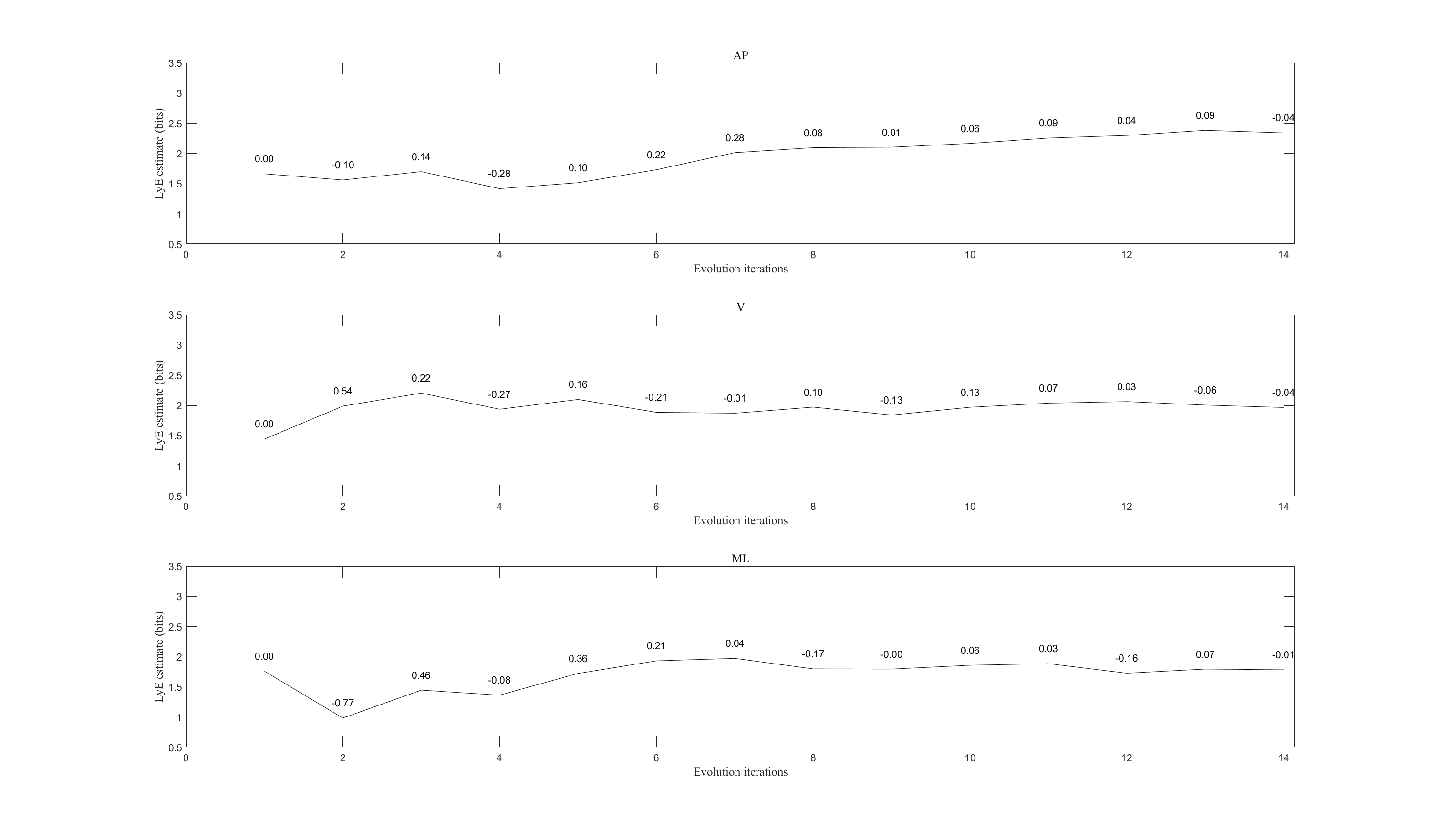

Supplement: Supplementary file 2 — Supplementary Information. [file 41598_2020_79584_MOESM2_ESM.zip › Participant17_trial4.png]

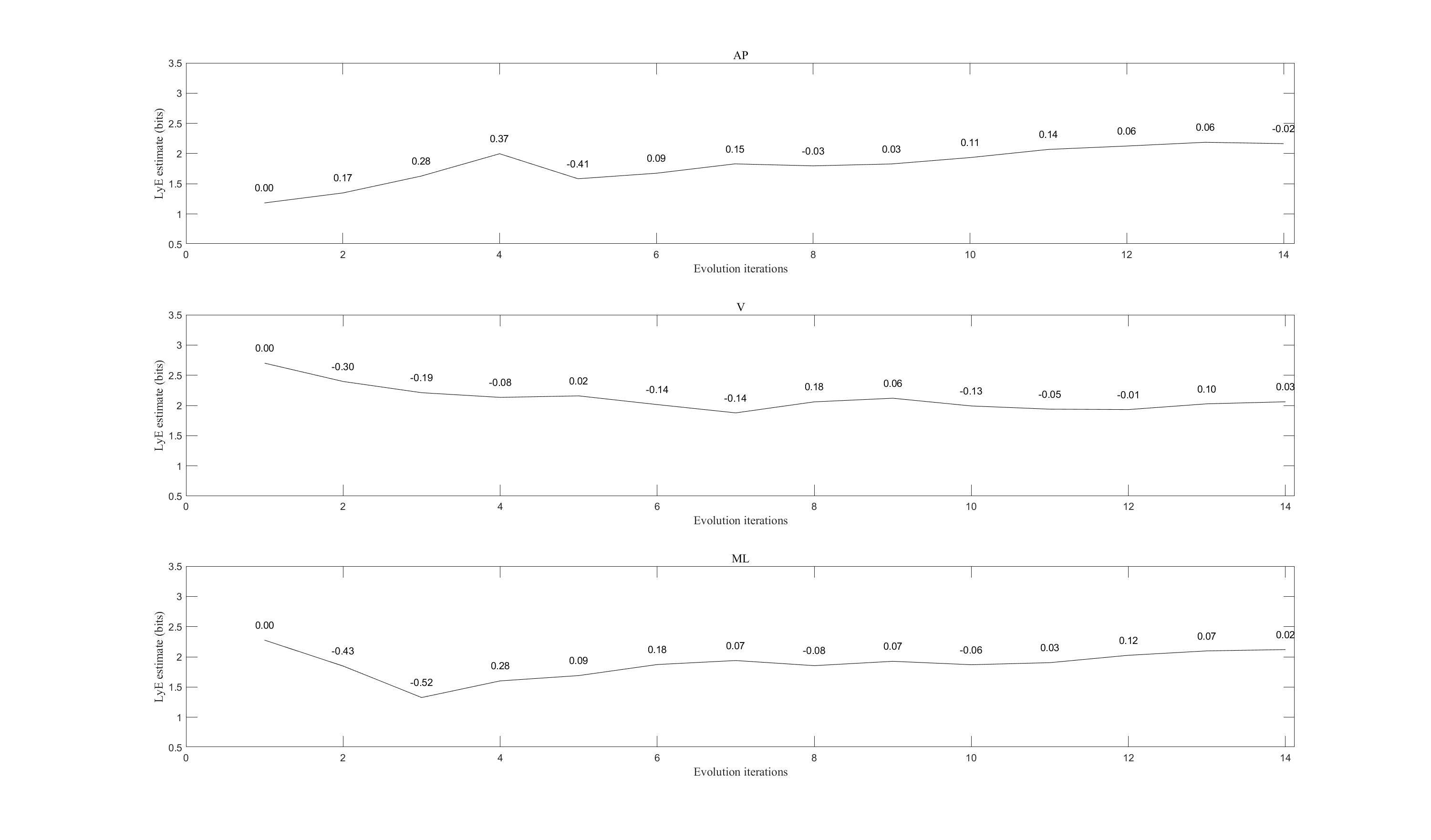

Supplement: Supplementary file 2 — Supplementary Information. [file 41598_2020_79584_MOESM2_ESM.zip › Participant17_trial5.png]

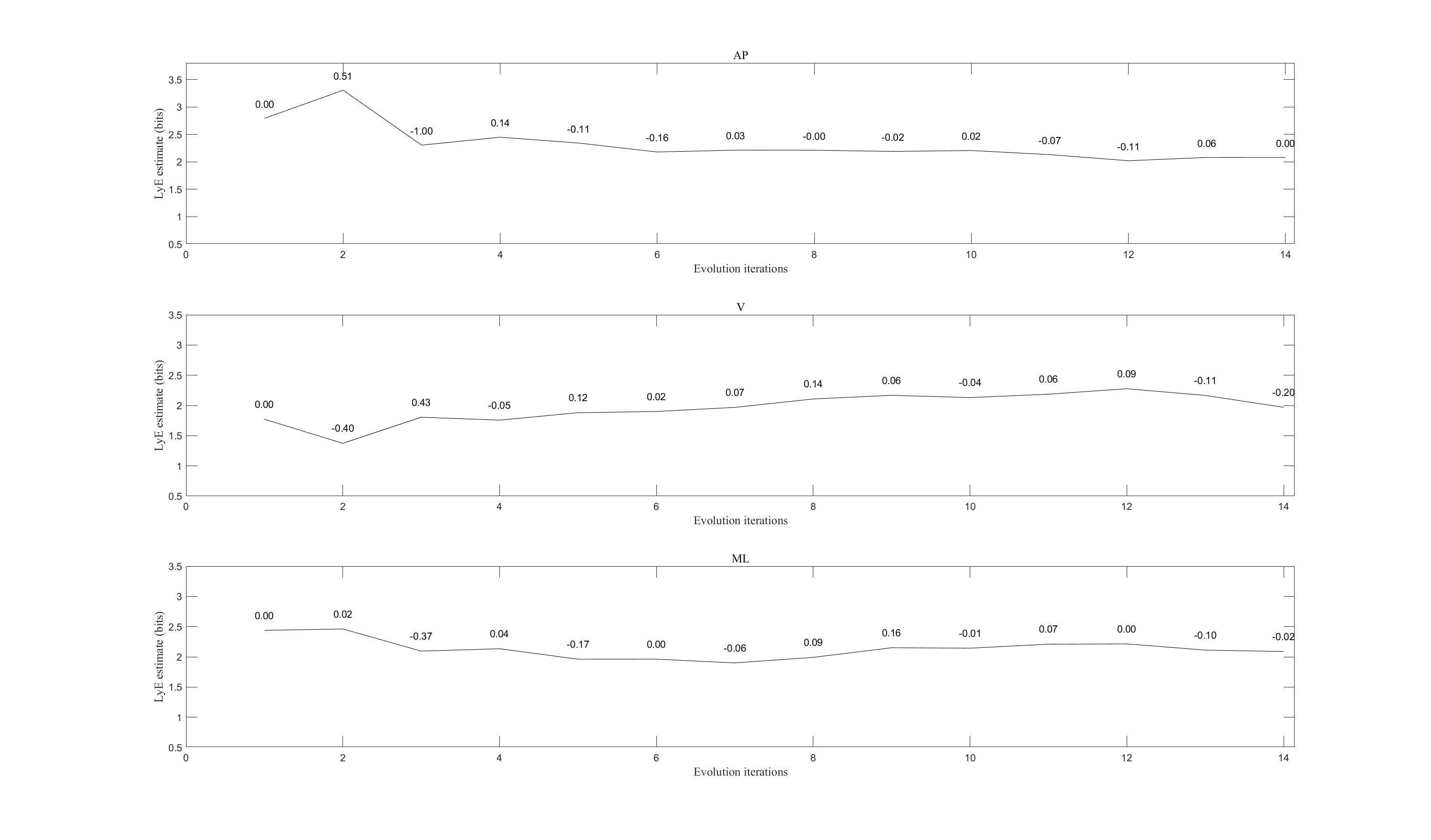

Supplement: Supplementary file 2 — Supplementary Information. [file 41598_2020_79584_MOESM2_ESM.zip › Participant17_trial6.png]

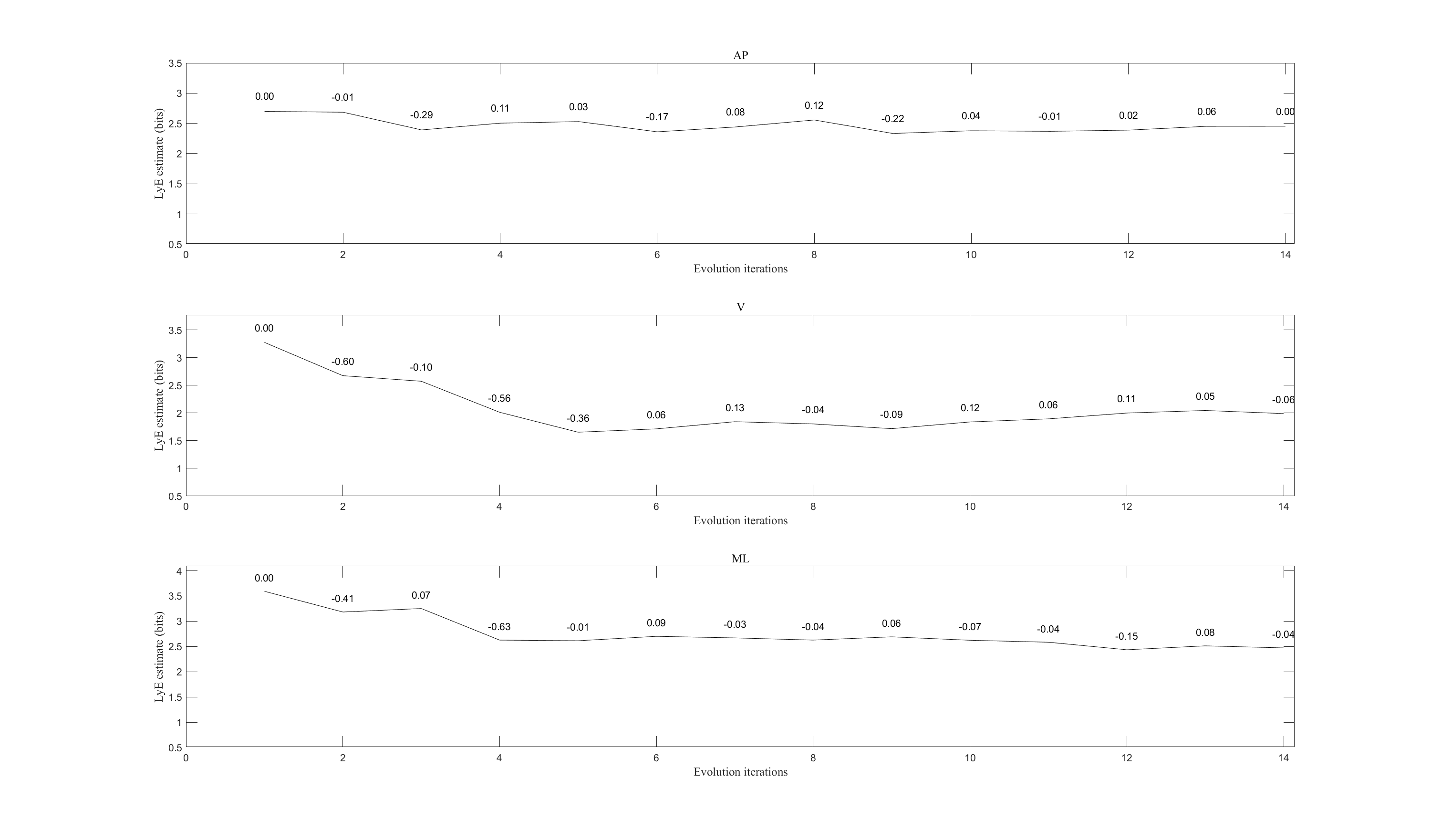

Supplement: Supplementary file 2 — Supplementary Information. [file 41598_2020_79584_MOESM2_ESM.zip › Participant17_trial7.png]

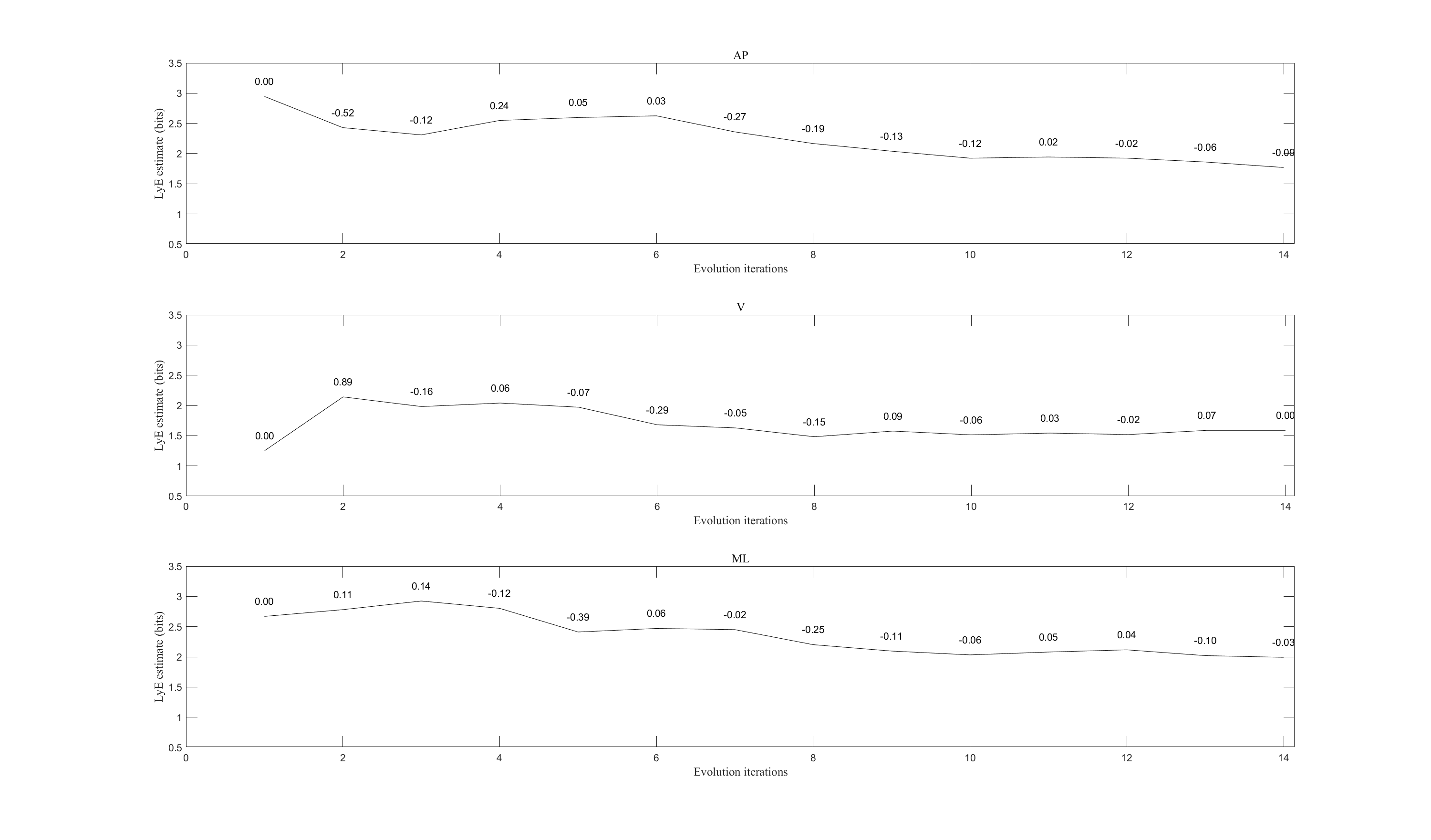

Supplement: Supplementary file 2 — Supplementary Information. [file 41598_2020_79584_MOESM2_ESM.zip › Participant17_trial8.png]

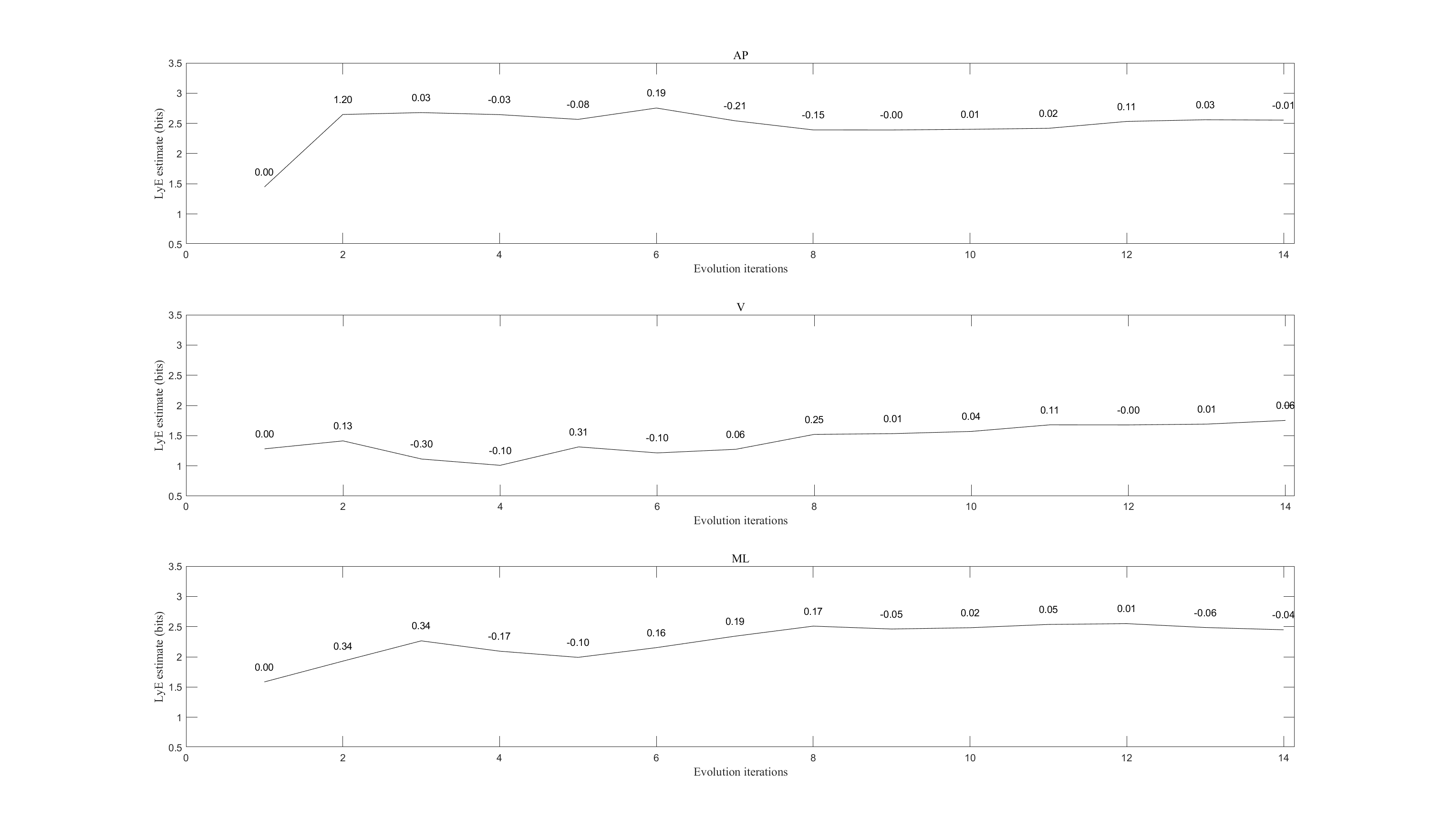

Supplement: Supplementary file 2 — Supplementary Information. [file 41598_2020_79584_MOESM2_ESM.zip › Participant17_trial9.png]

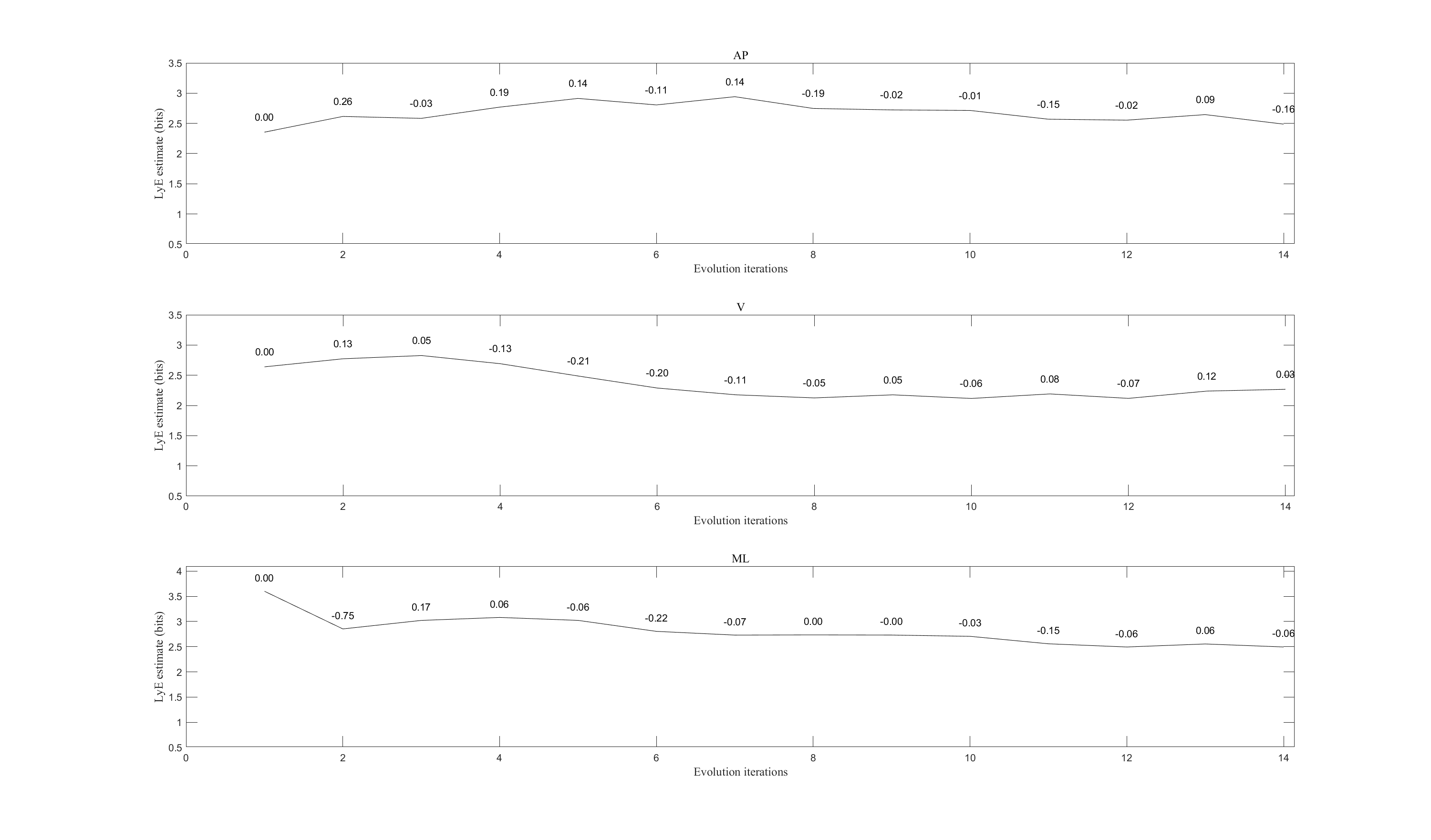

Supplement: Supplementary file 2 — Supplementary Information. [file 41598_2020_79584_MOESM2_ESM.zip › Participant18_trial1.png]

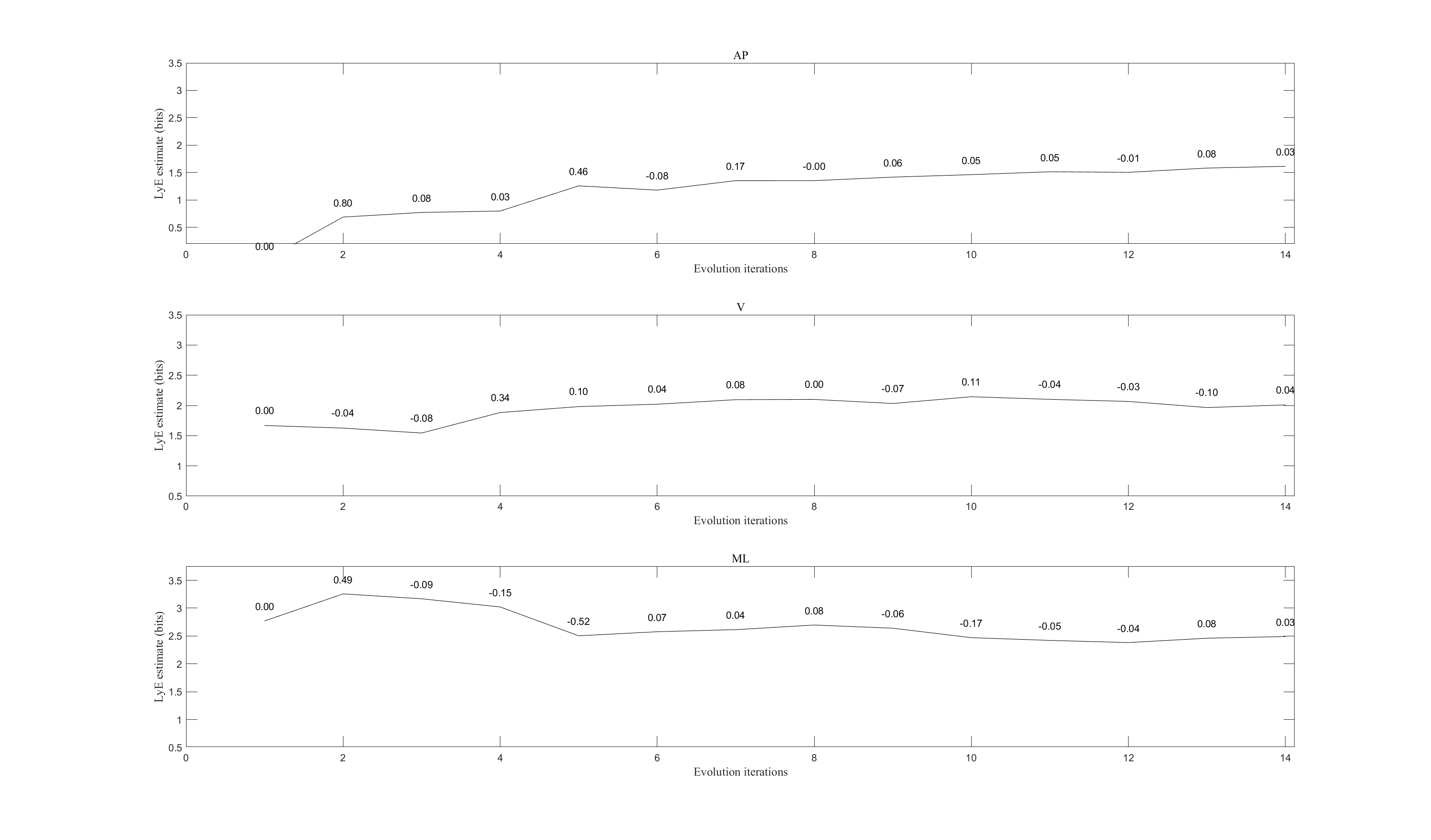

Supplement: Supplementary file 2 — Supplementary Information. [file 41598_2020_79584_MOESM2_ESM.zip › Participant18_trial10.png]

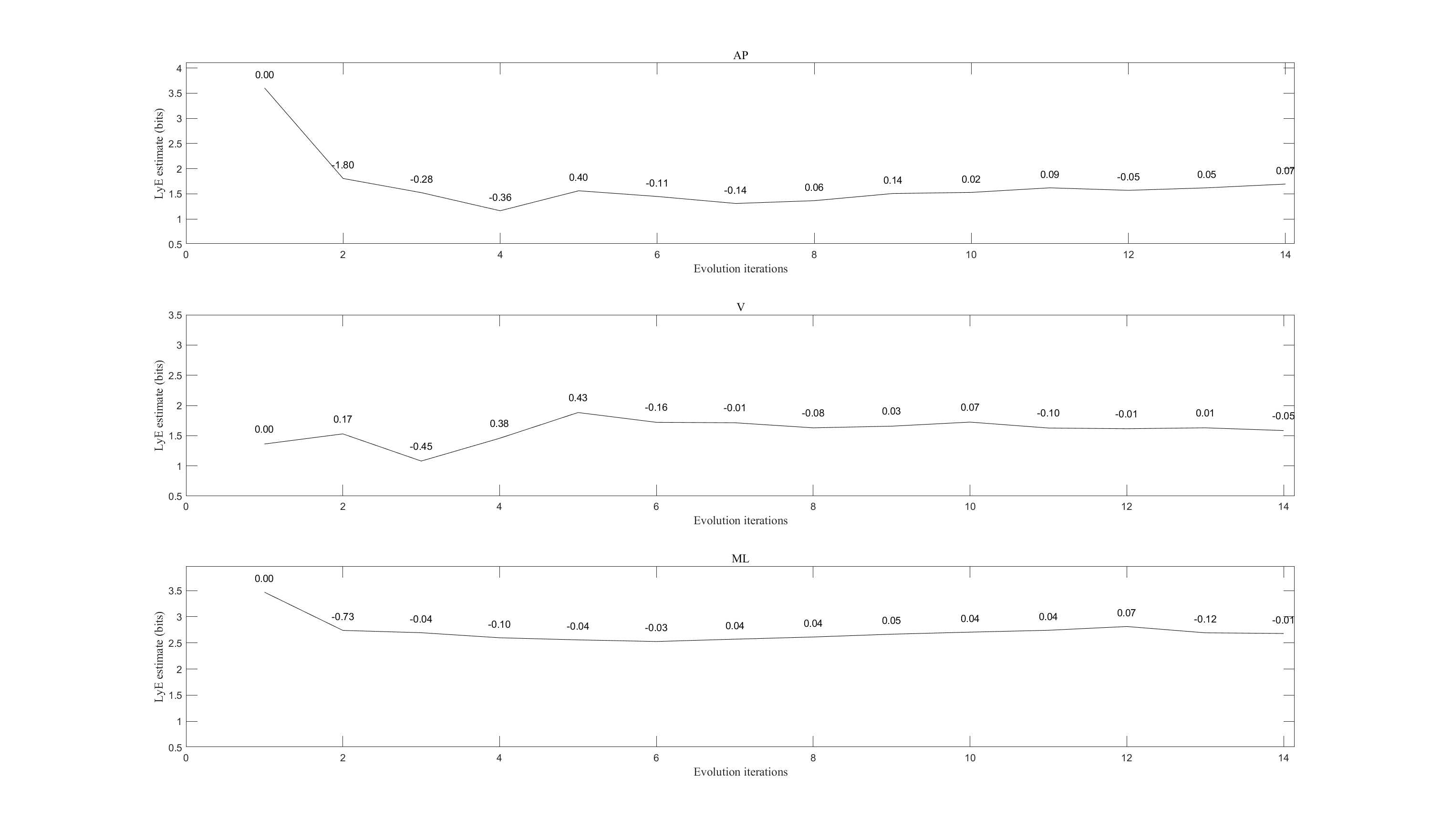

Supplement: Supplementary file 2 — Supplementary Information. [file 41598_2020_79584_MOESM2_ESM.zip › Participant18_trial11.png]

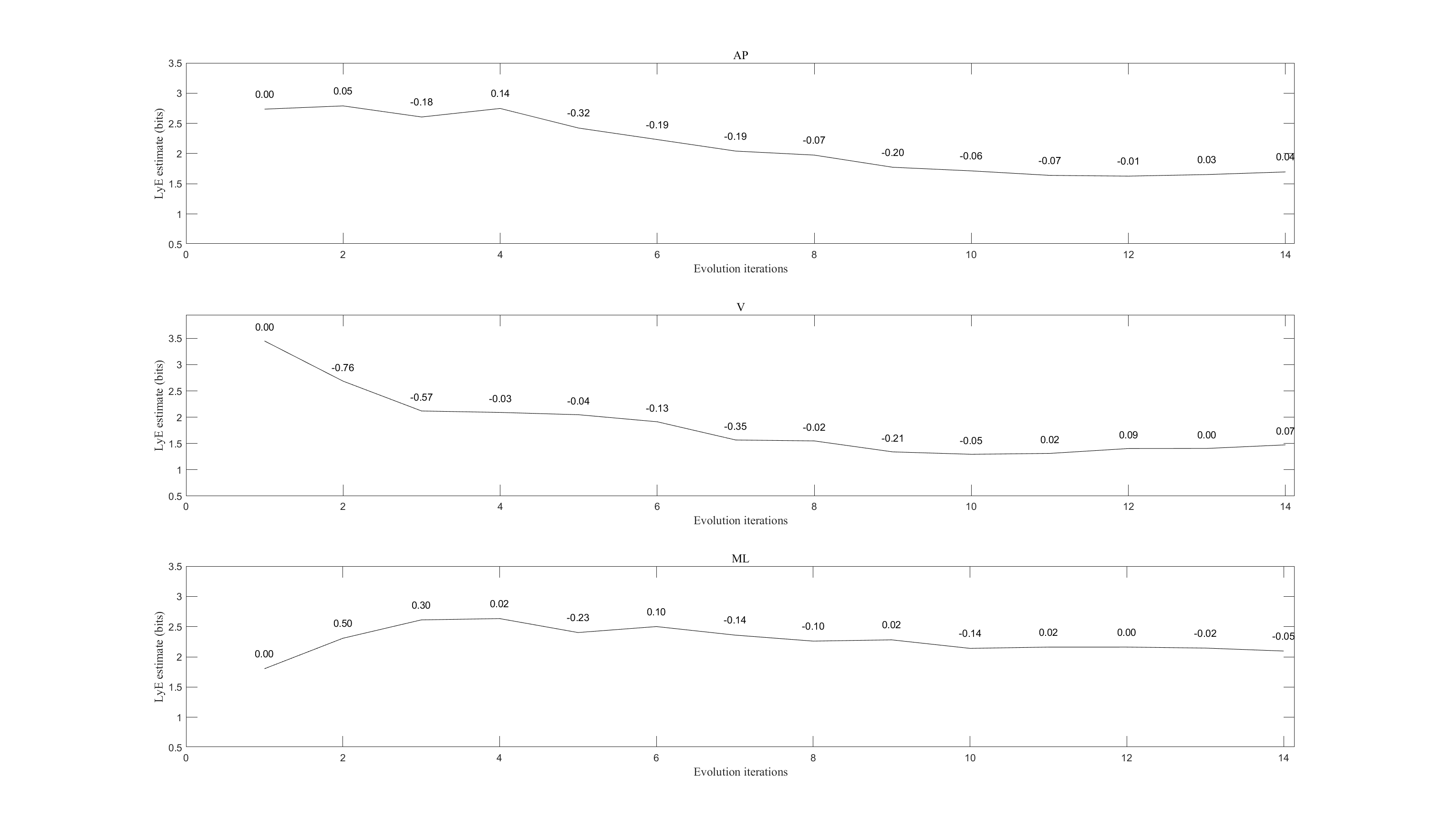

Supplement: Supplementary file 2 — Supplementary Information. [file 41598_2020_79584_MOESM2_ESM.zip › Participant18_trial12.png]

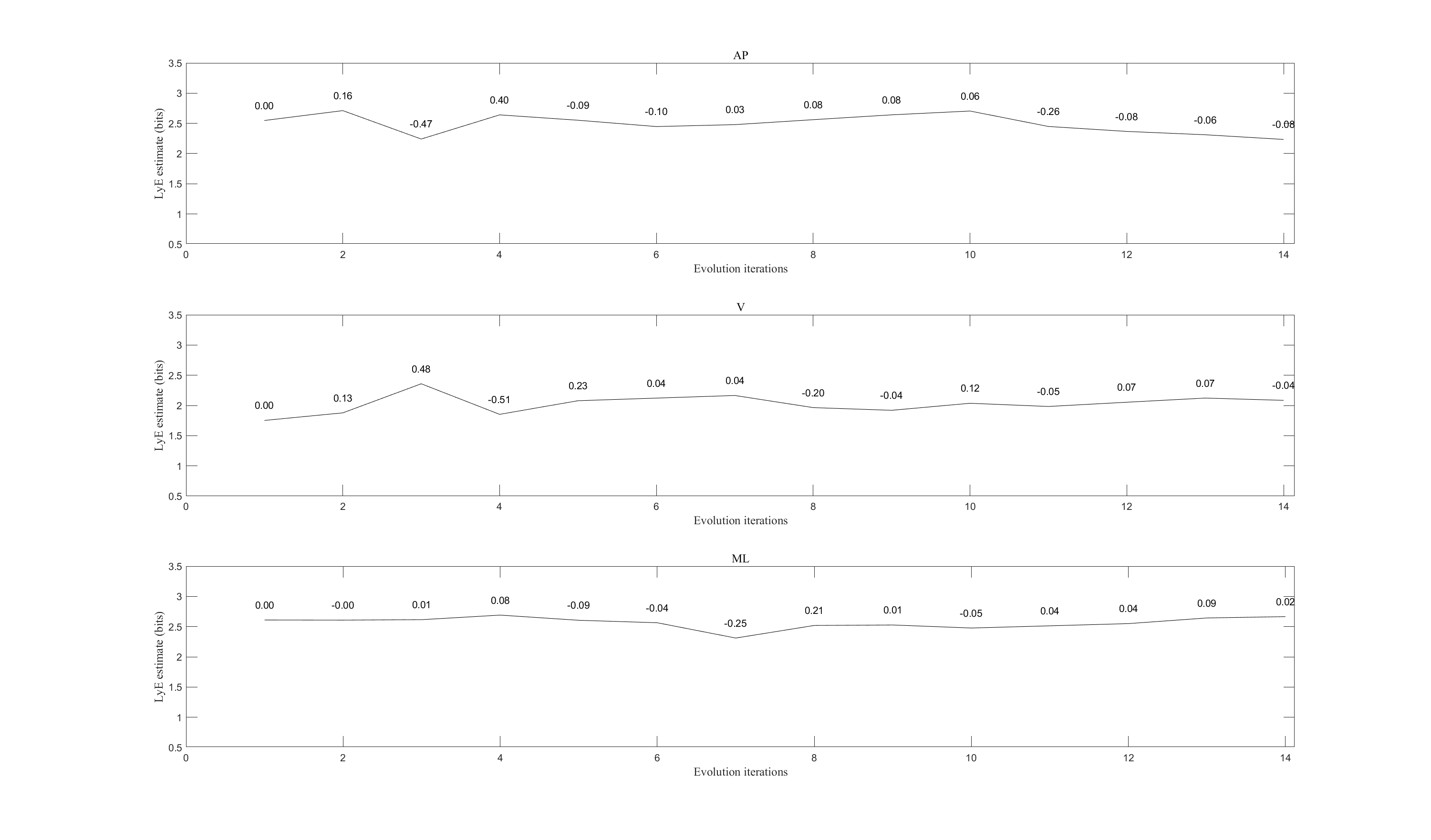

Supplement: Supplementary file 2 — Supplementary Information. [file 41598_2020_79584_MOESM2_ESM.zip › Participant18_trial2.png]

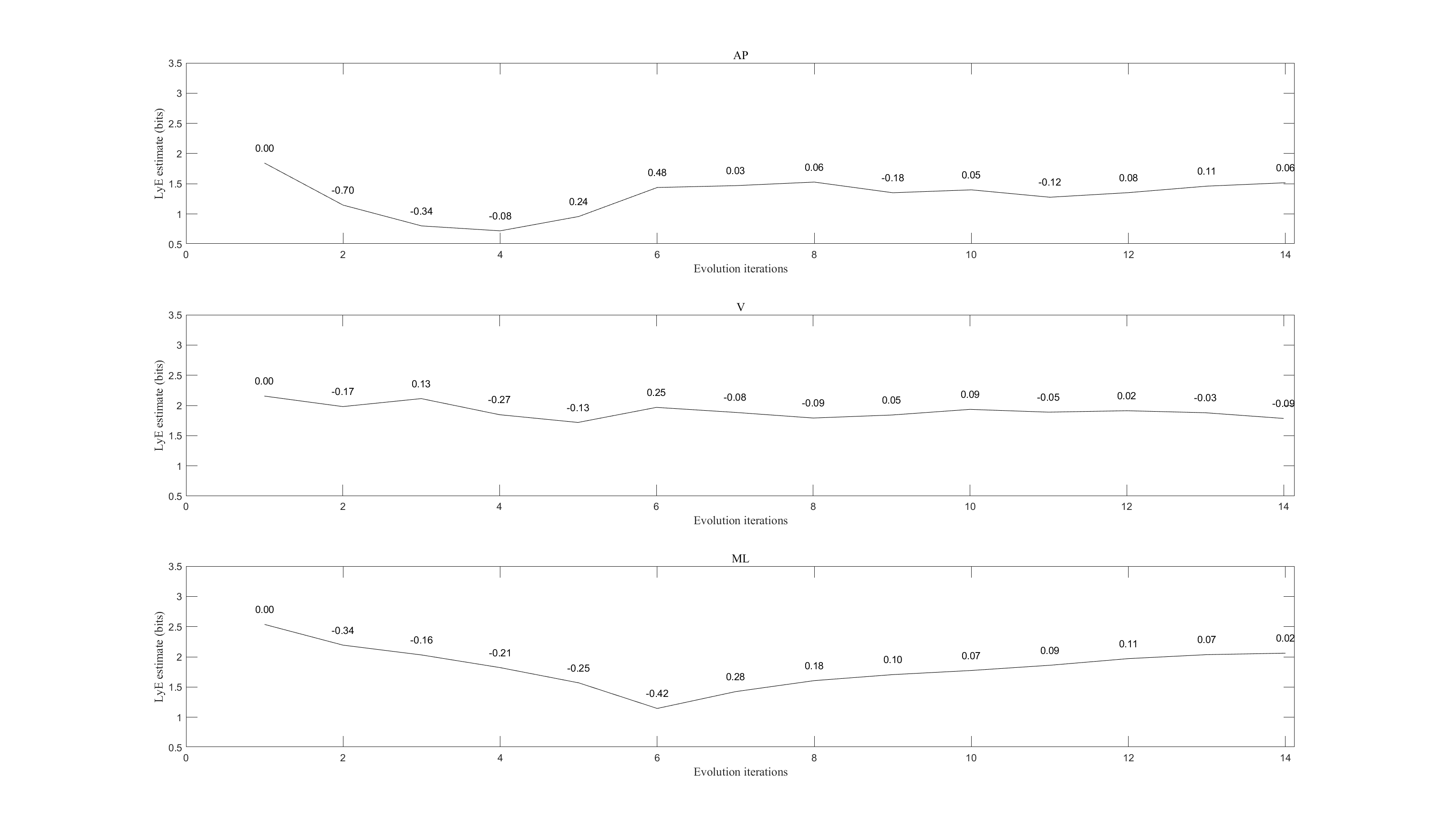

Supplement: Supplementary file 2 — Supplementary Information. [file 41598_2020_79584_MOESM2_ESM.zip › Participant18_trial3.png]

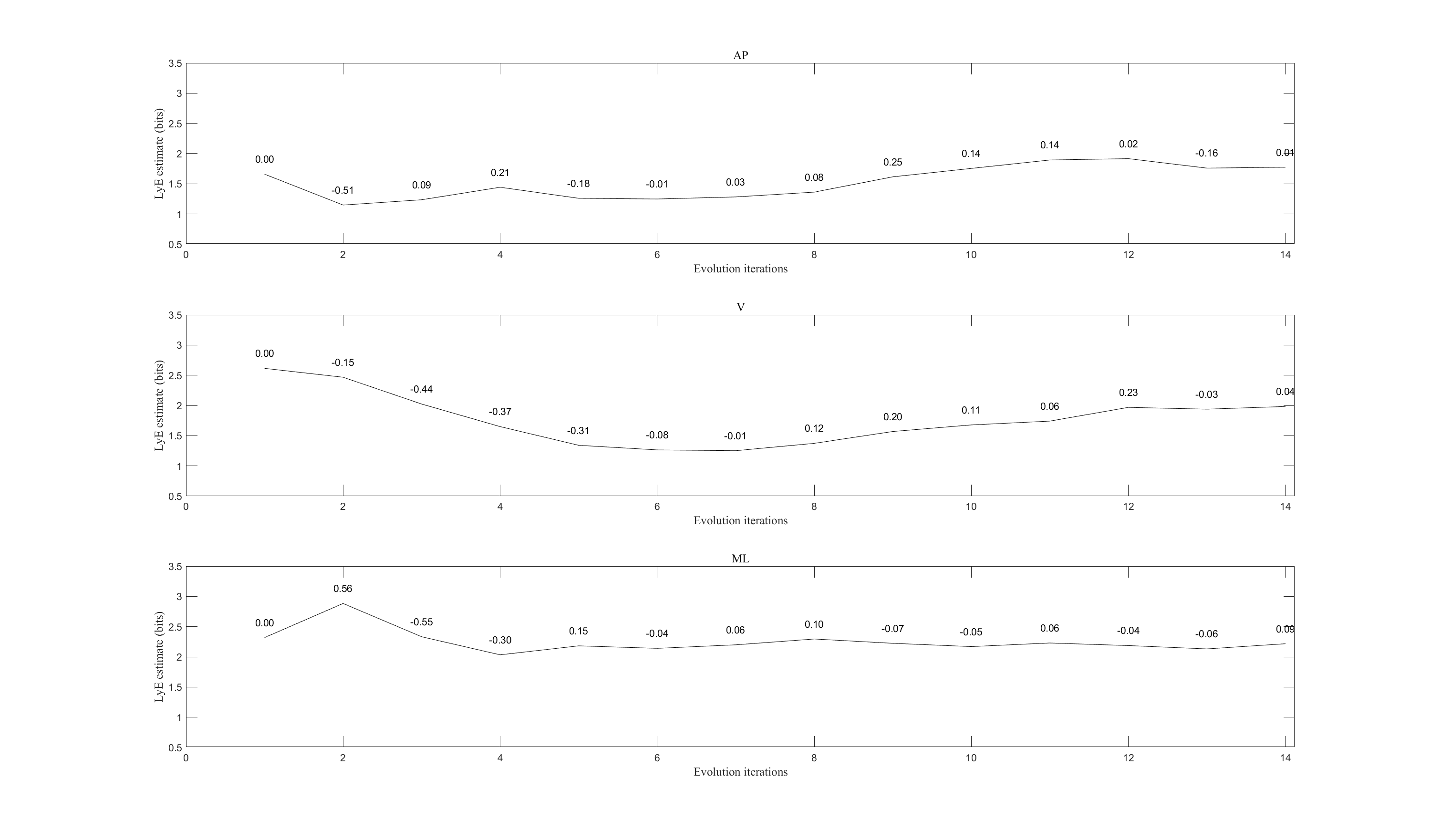

Supplement: Supplementary file 2 — Supplementary Information. [file 41598_2020_79584_MOESM2_ESM.zip › Participant18_trial4.png]

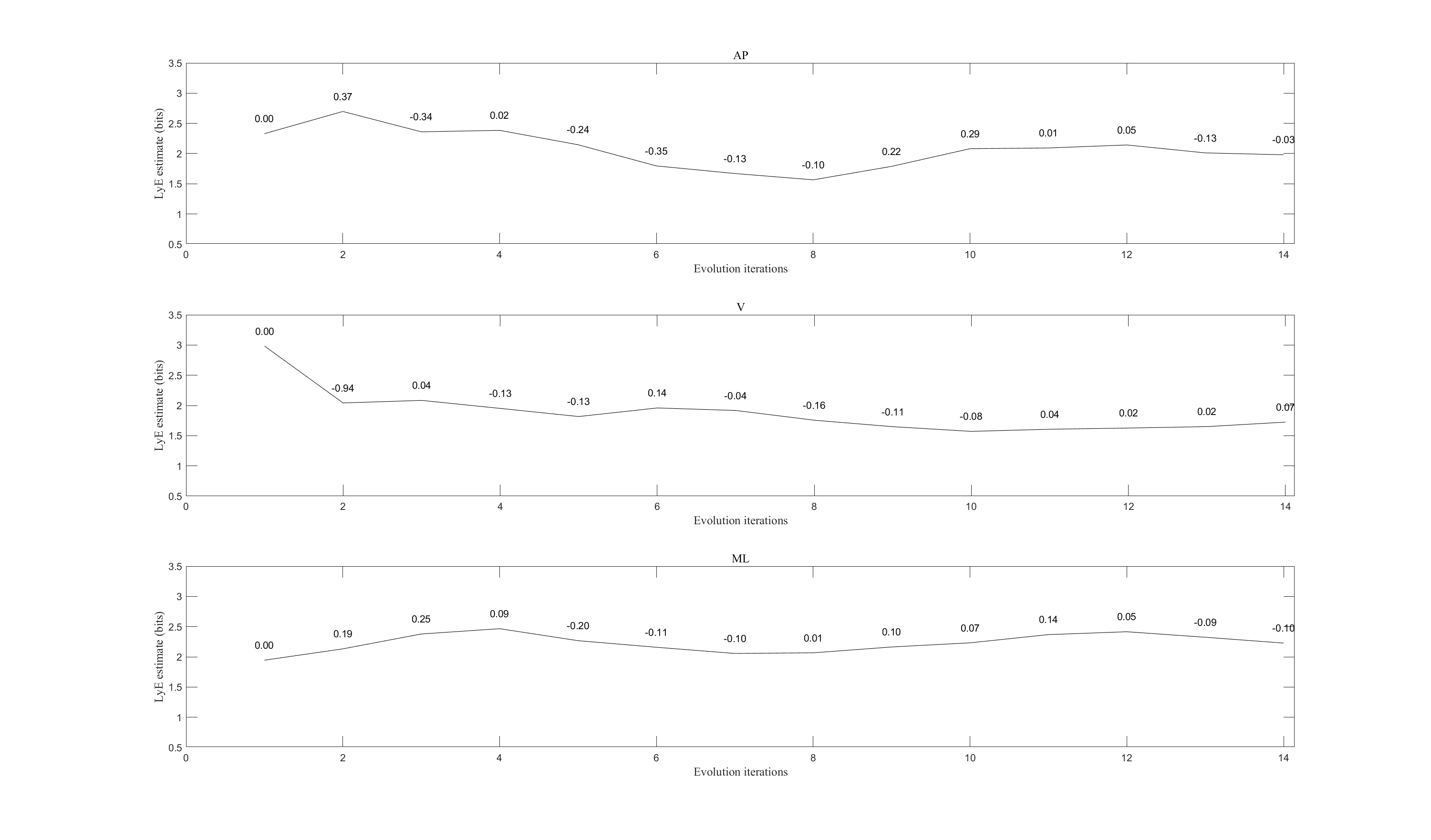

Supplement: Supplementary file 2 — Supplementary Information. [file 41598_2020_79584_MOESM2_ESM.zip › Participant18_trial5.png]

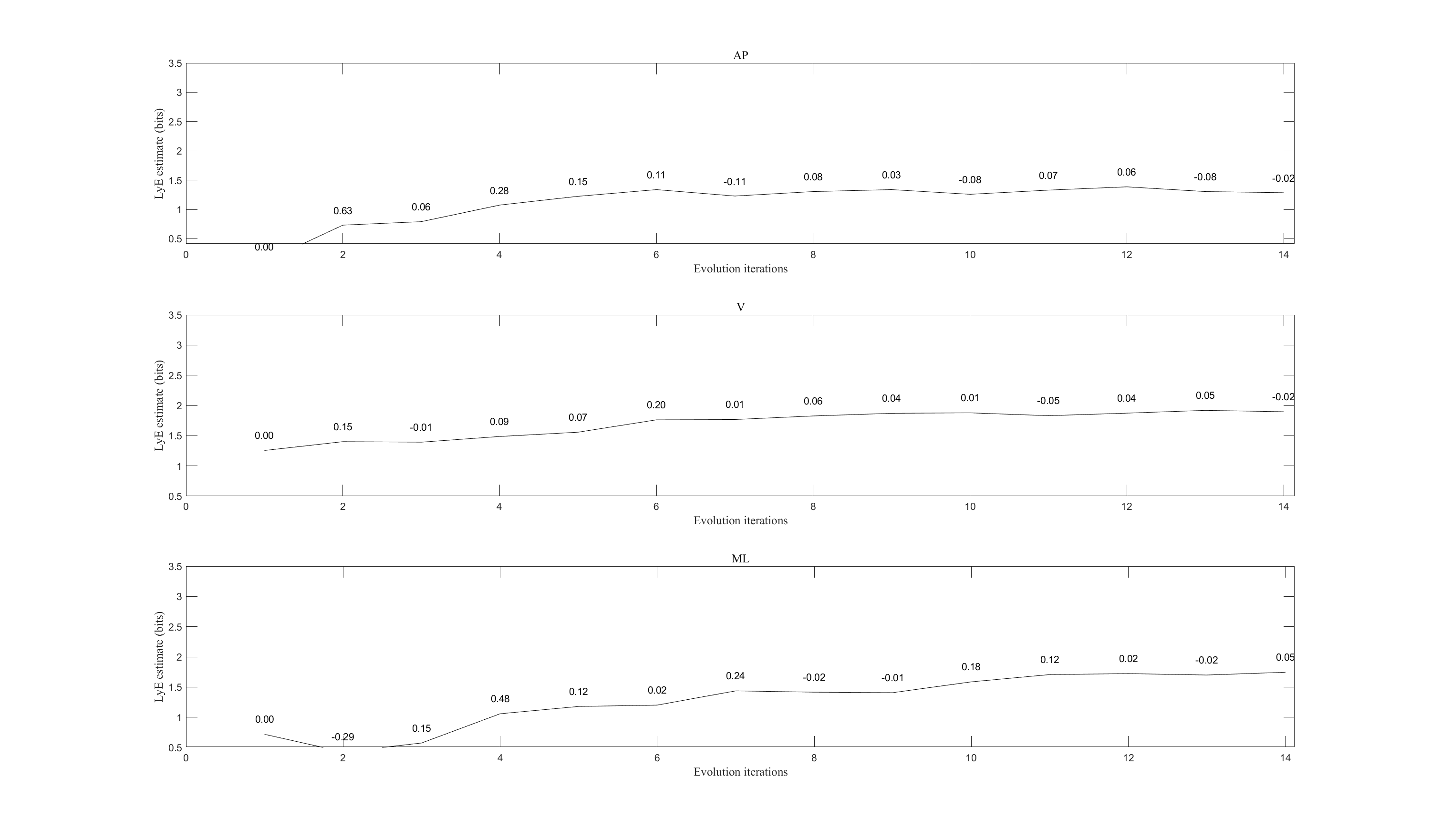

Supplement: Supplementary file 2 — Supplementary Information. [file 41598_2020_79584_MOESM2_ESM.zip › Participant18_trial6.png]

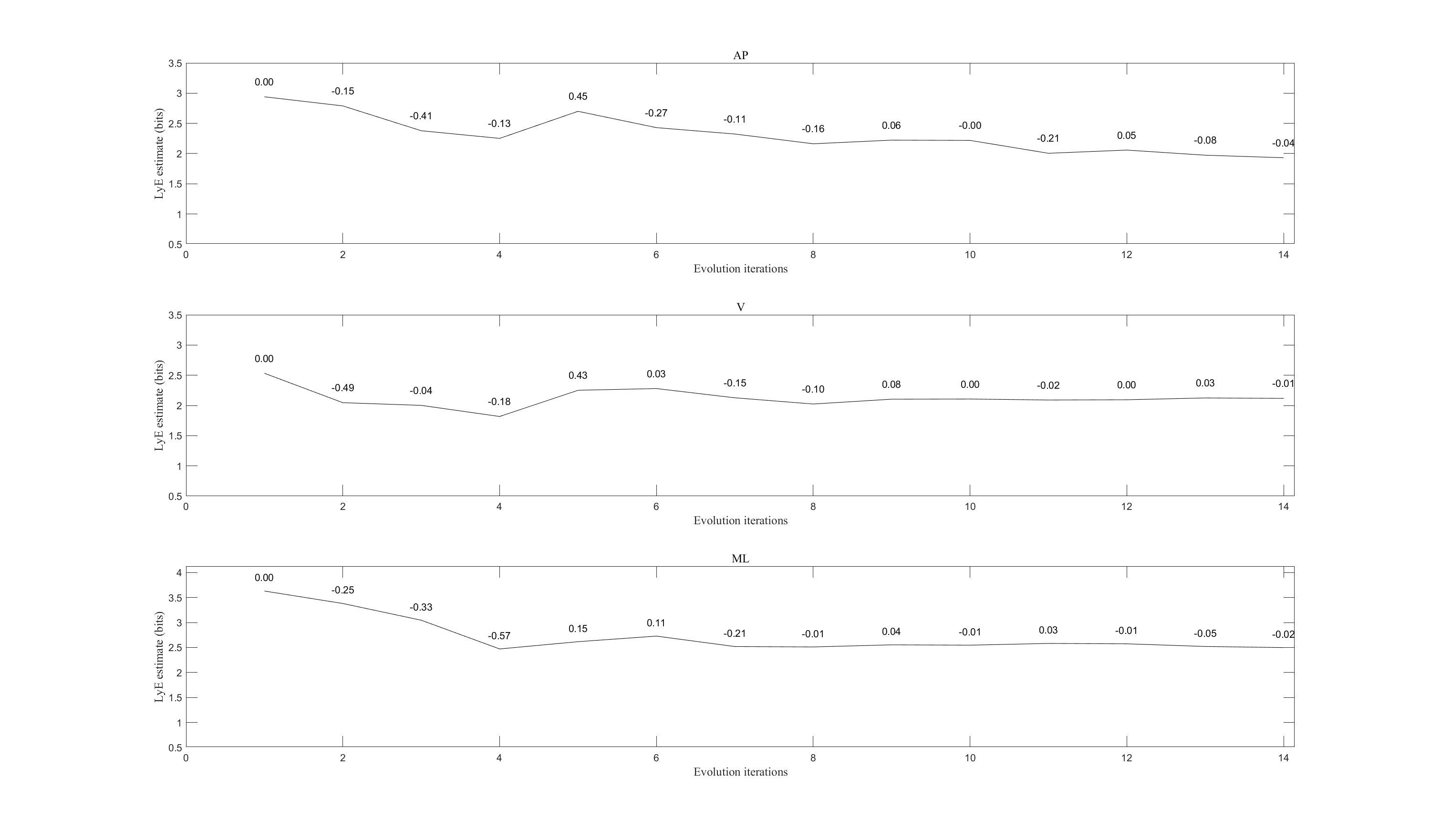

Supplement: Supplementary file 2 — Supplementary Information. [file 41598_2020_79584_MOESM2_ESM.zip › Participant18_trial7.png]

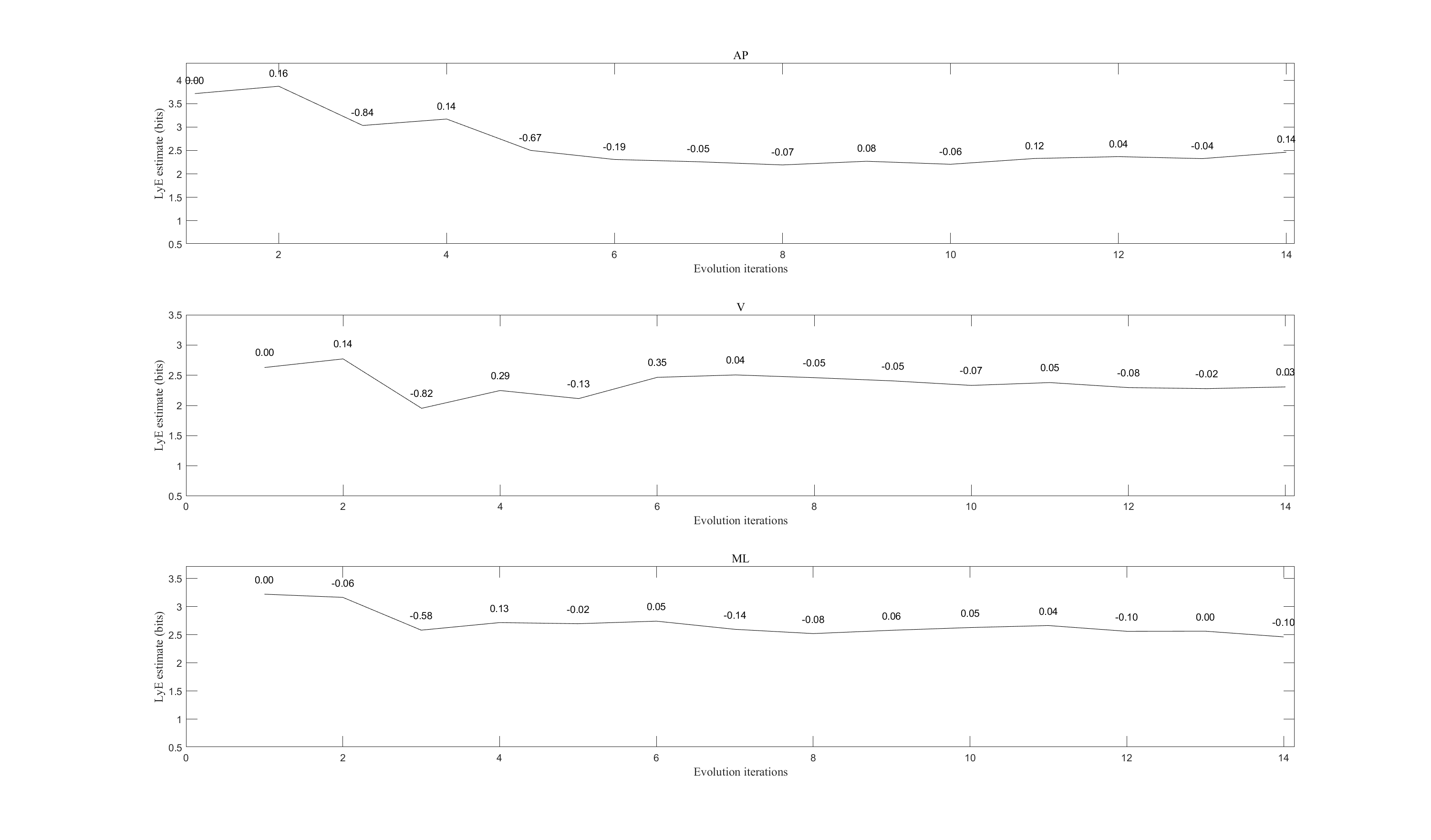

Supplement: Supplementary file 2 — Supplementary Information. [file 41598_2020_79584_MOESM2_ESM.zip › Participant18_trial8.png]

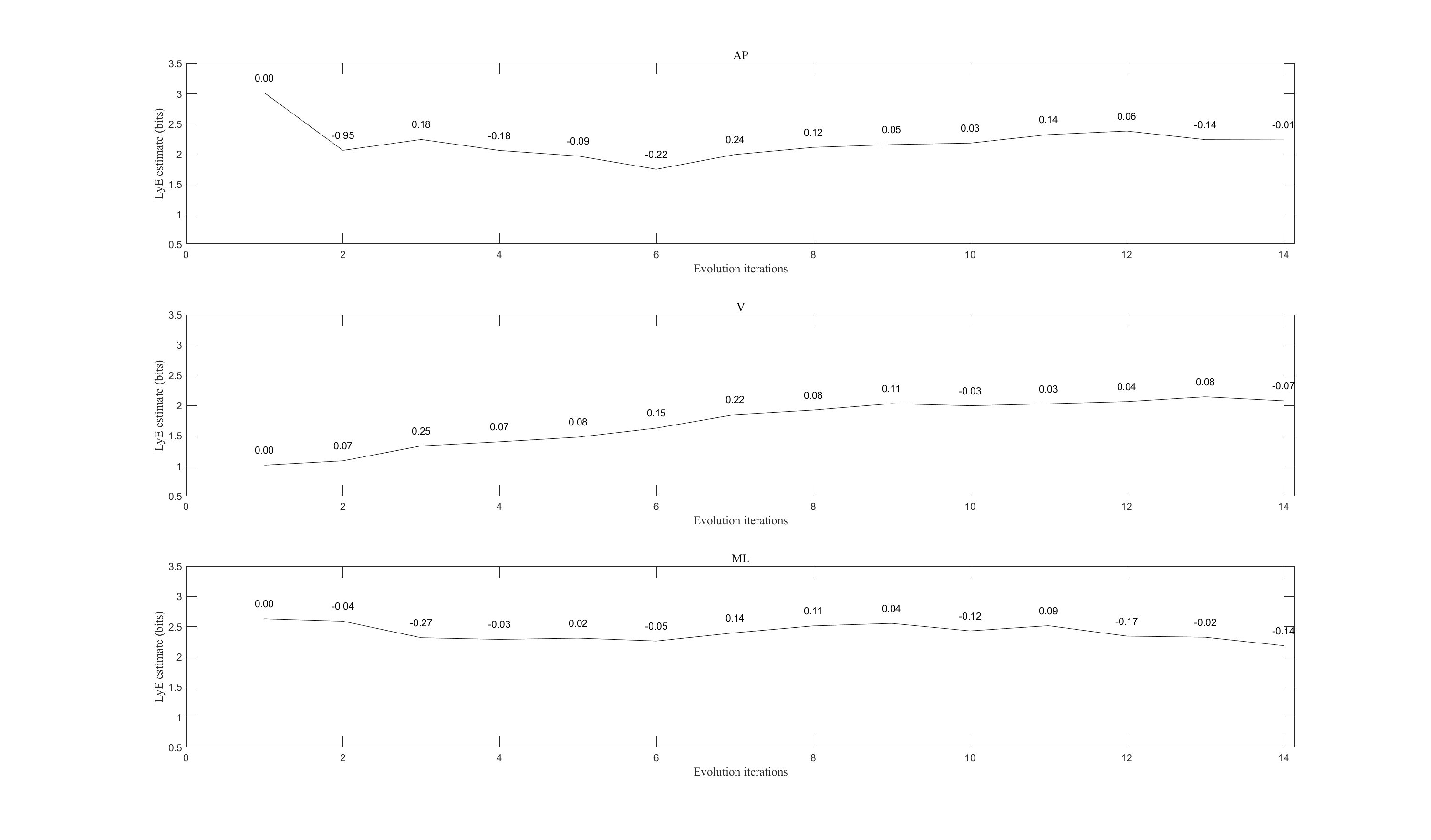

Supplement: Supplementary file 2 — Supplementary Information. [file 41598_2020_79584_MOESM2_ESM.zip › Participant18_trial9.png]

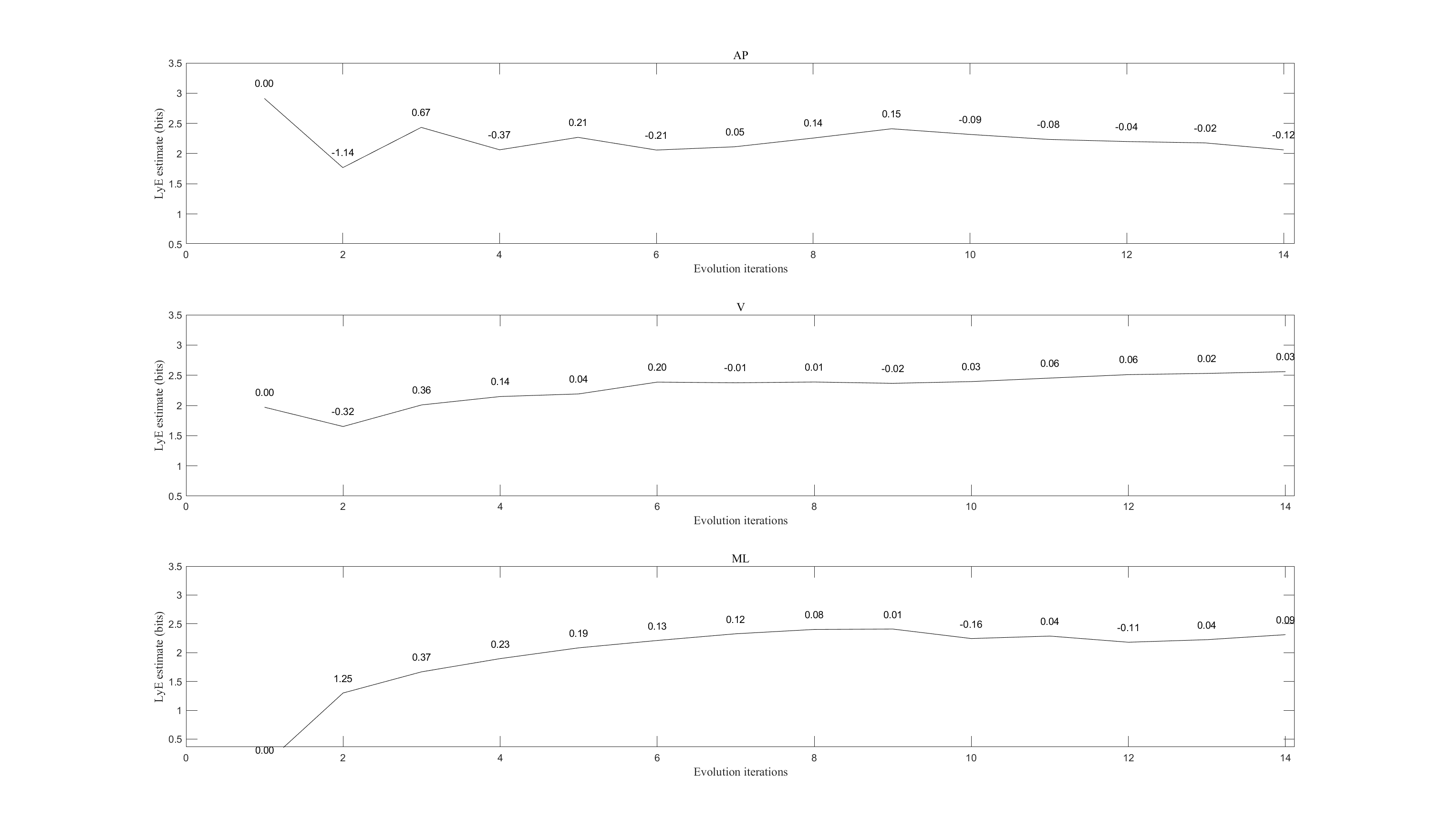

Supplement: Supplementary file 2 — Supplementary Information. [file 41598_2020_79584_MOESM2_ESM.zip › Participant19_trial1.png]

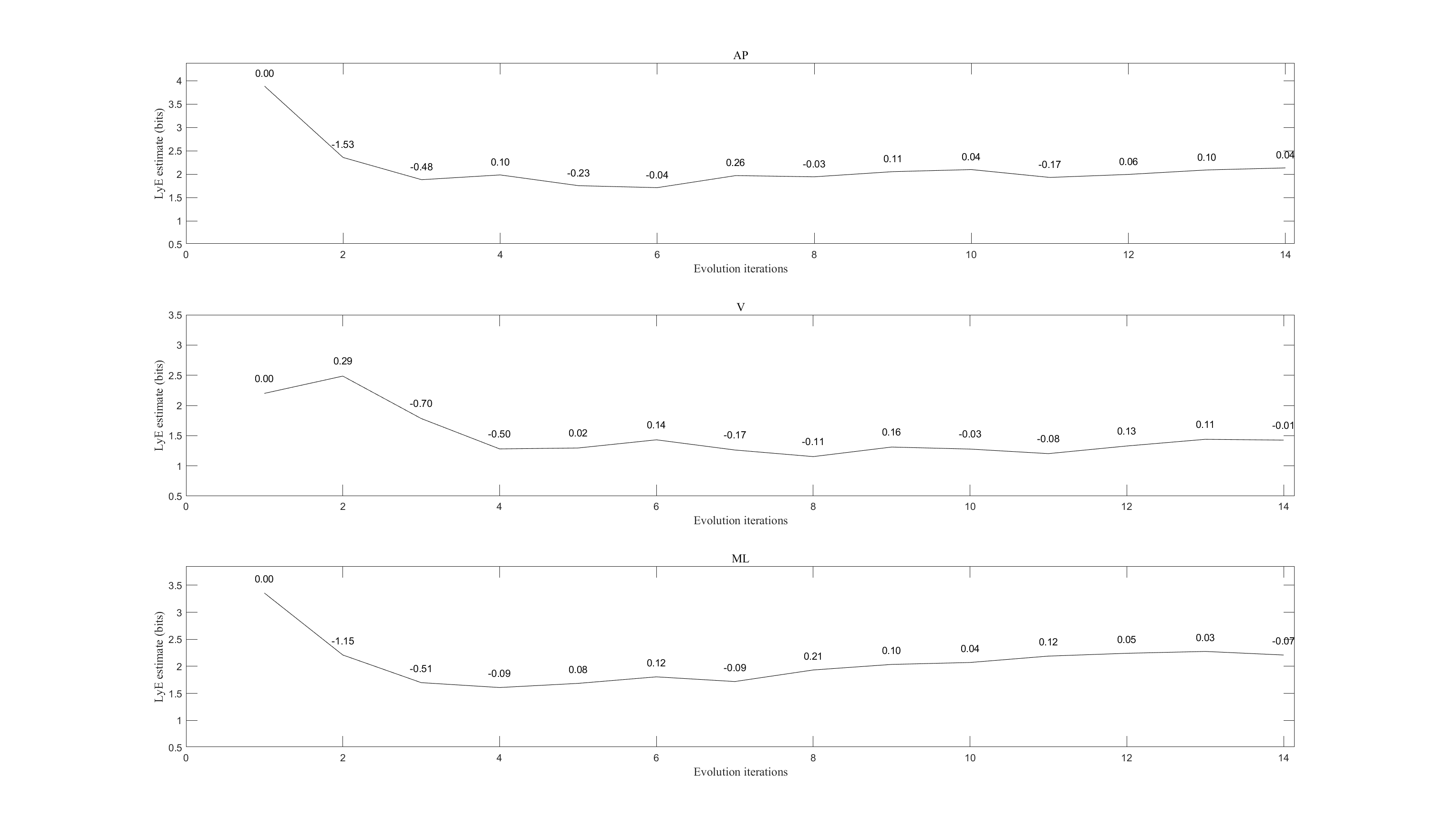

Supplement: Supplementary file 2 — Supplementary Information. [file 41598_2020_79584_MOESM2_ESM.zip › Participant19_trial10.png]

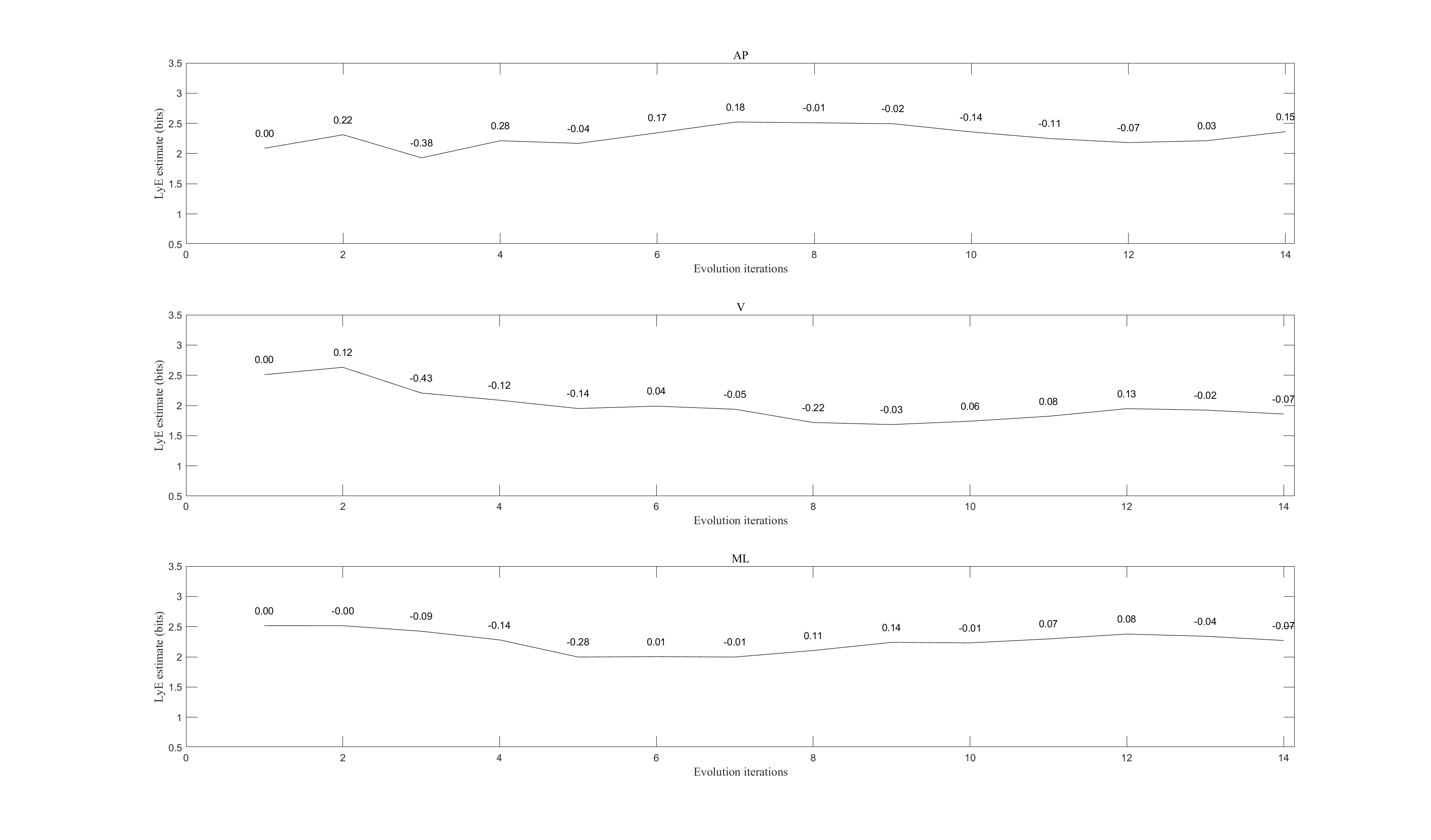

Supplement: Supplementary file 2 — Supplementary Information. [file 41598_2020_79584_MOESM2_ESM.zip › Participant19_trial11.png]

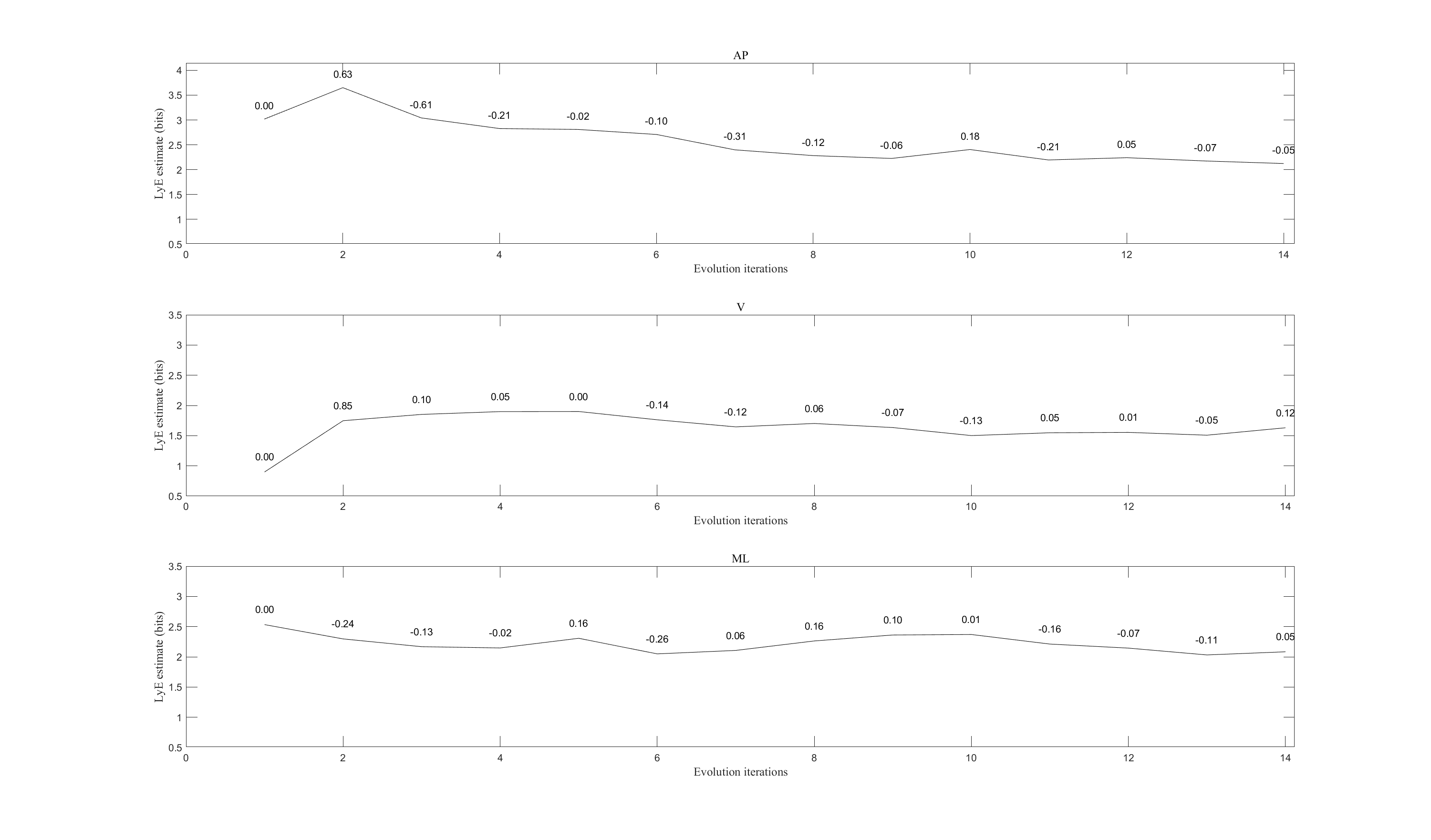

Supplement: Supplementary file 2 — Supplementary Information. [file 41598_2020_79584_MOESM2_ESM.zip › Participant19_trial12.png]

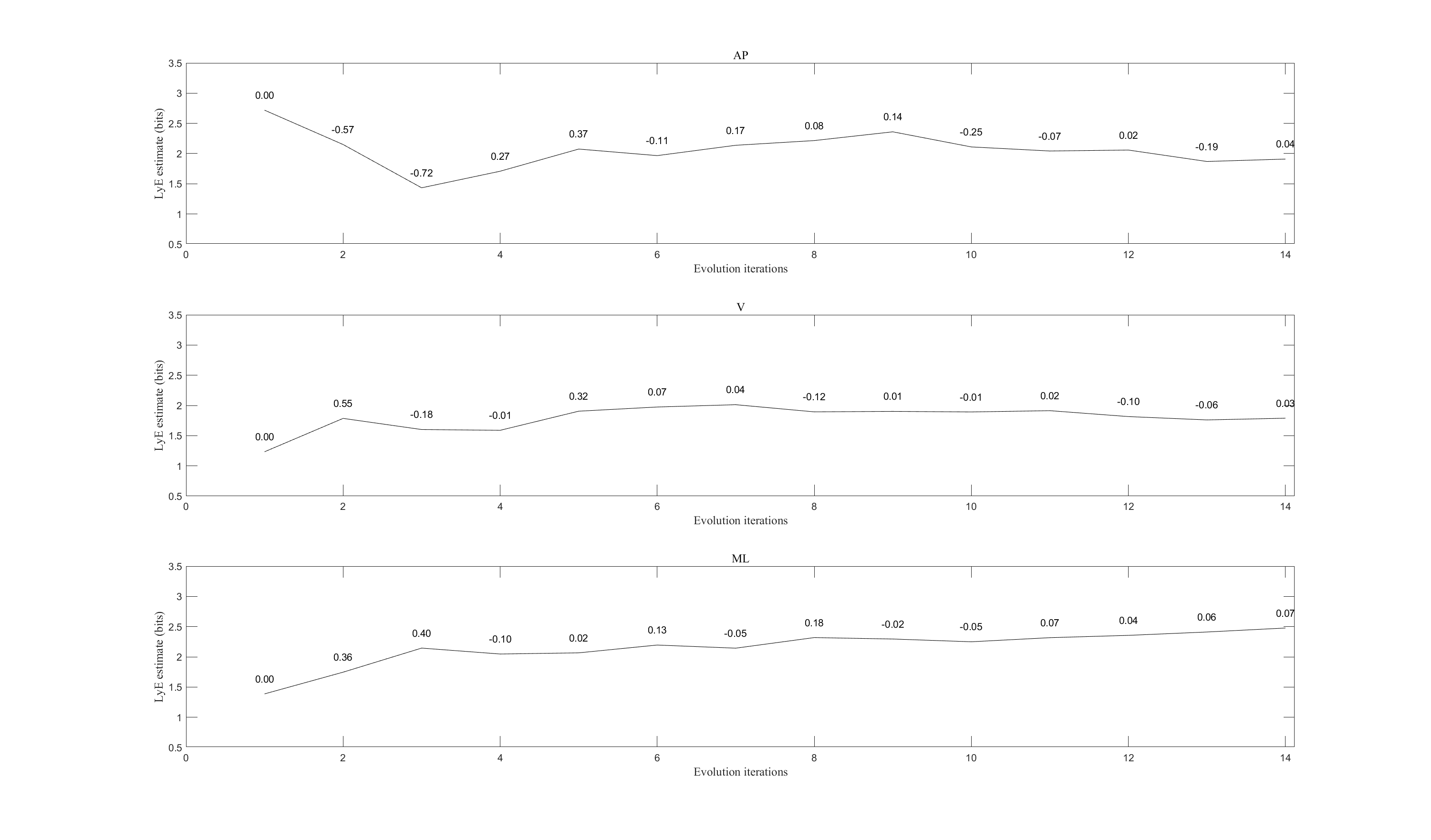

Supplement: Supplementary file 2 — Supplementary Information. [file 41598_2020_79584_MOESM2_ESM.zip › Participant19_trial2.png]

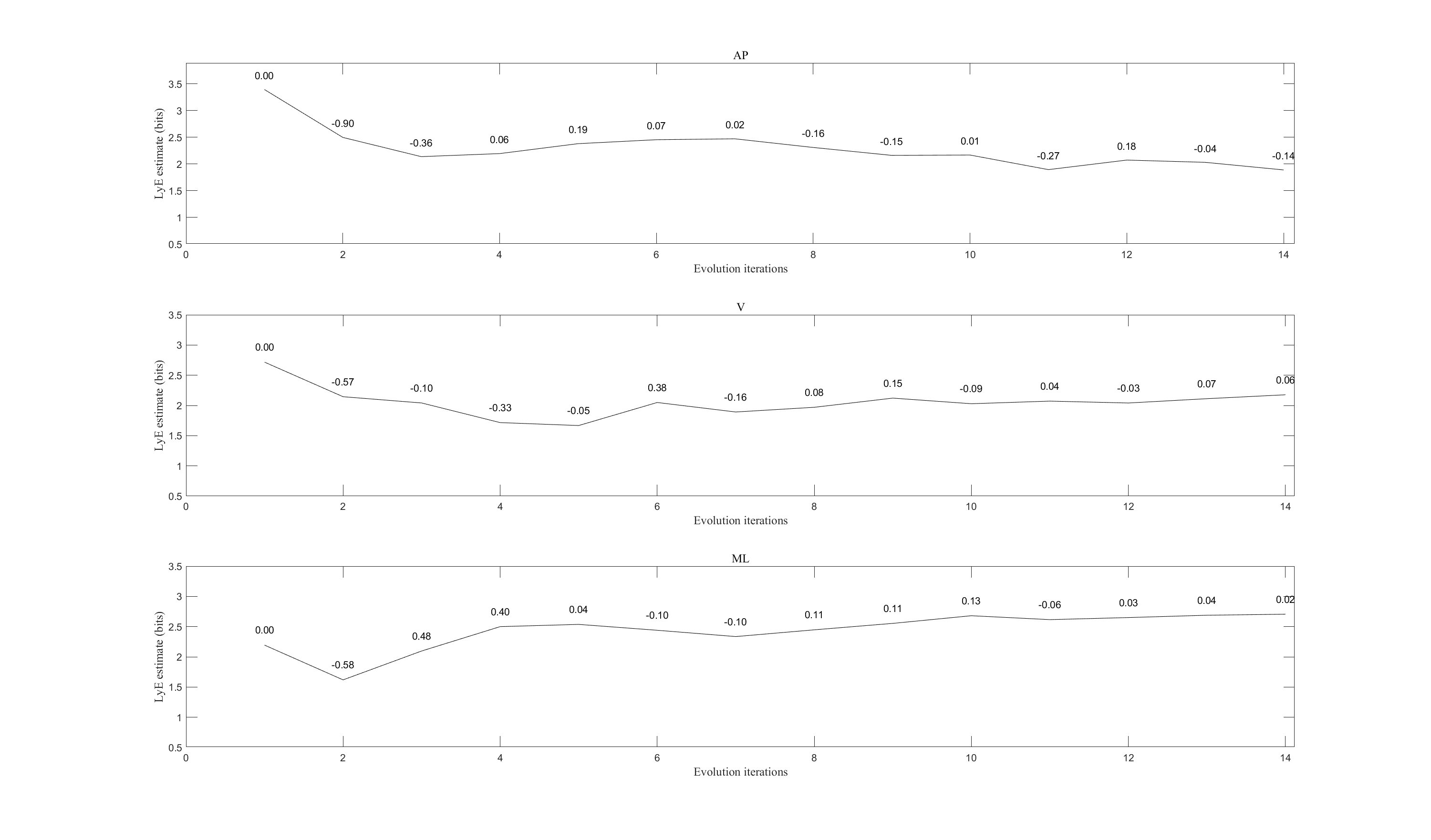

Supplement: Supplementary file 2 — Supplementary Information. [file 41598_2020_79584_MOESM2_ESM.zip › Participant19_trial3.png]

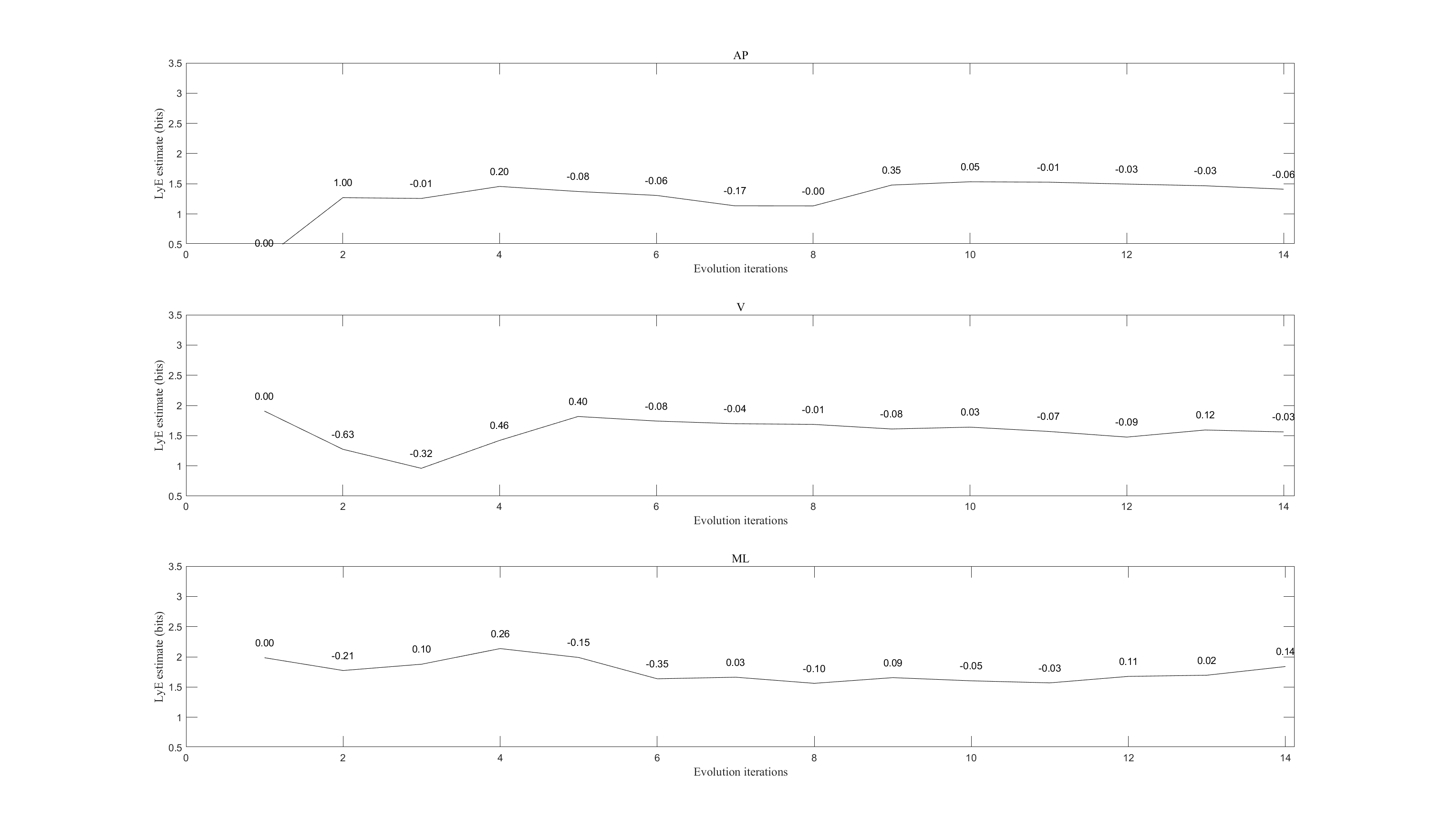

Supplement: Supplementary file 2 — Supplementary Information. [file 41598_2020_79584_MOESM2_ESM.zip › Participant19_trial4.png]

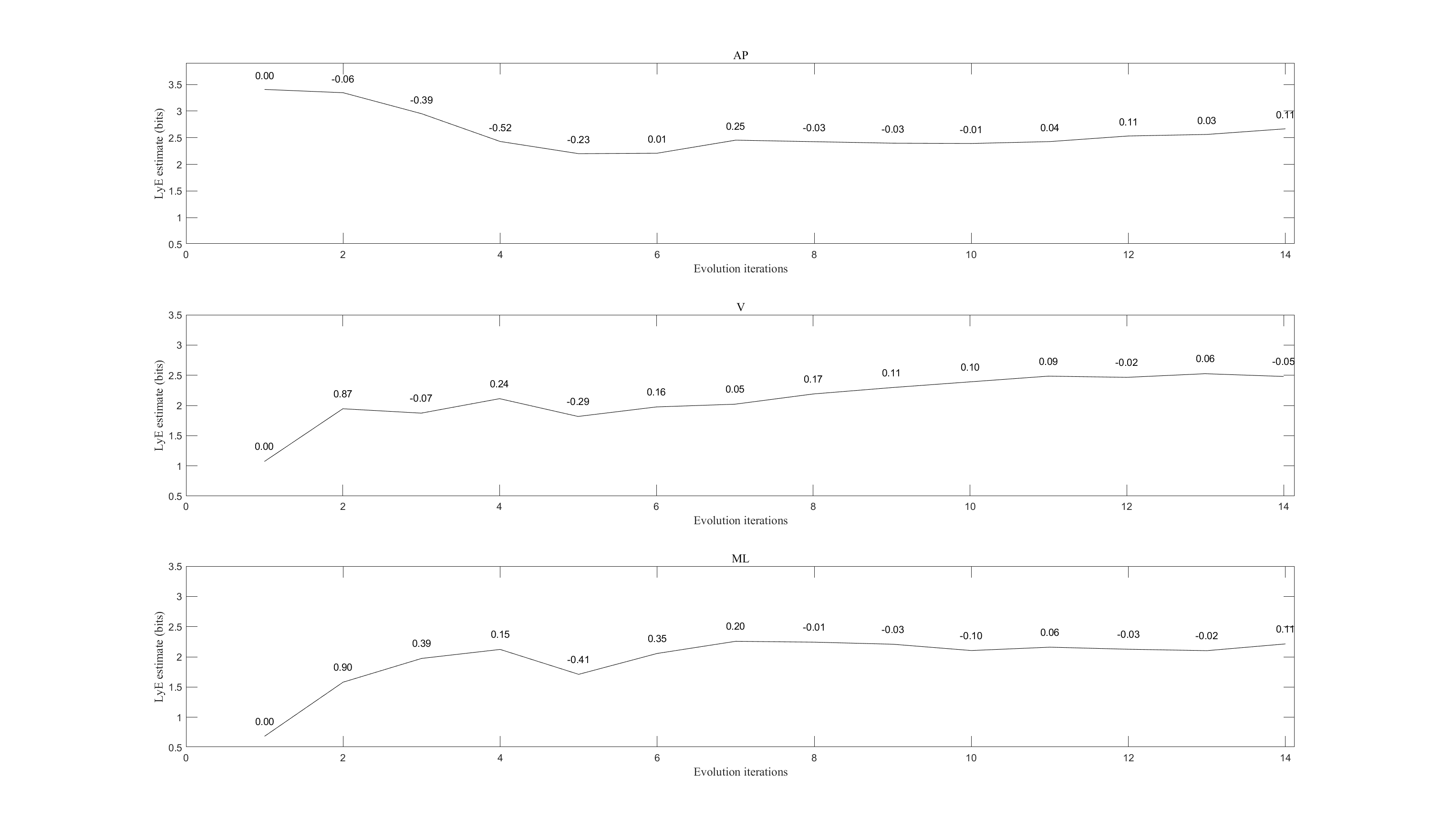

Supplement: Supplementary file 2 — Supplementary Information. [file 41598_2020_79584_MOESM2_ESM.zip › Participant19_trial5.png]

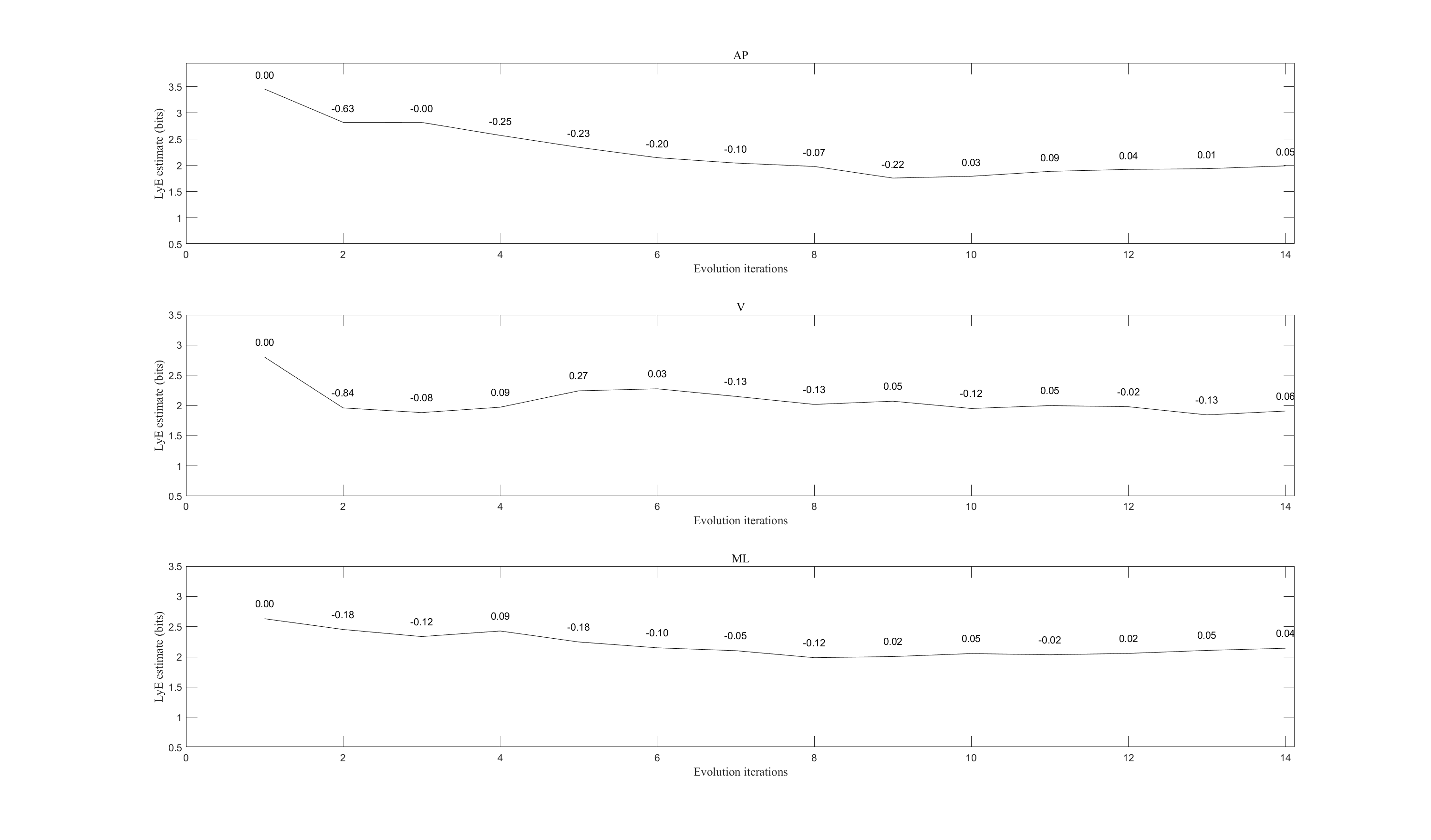

Supplement: Supplementary file 2 — Supplementary Information. [file 41598_2020_79584_MOESM2_ESM.zip › Participant19_trial6.png]

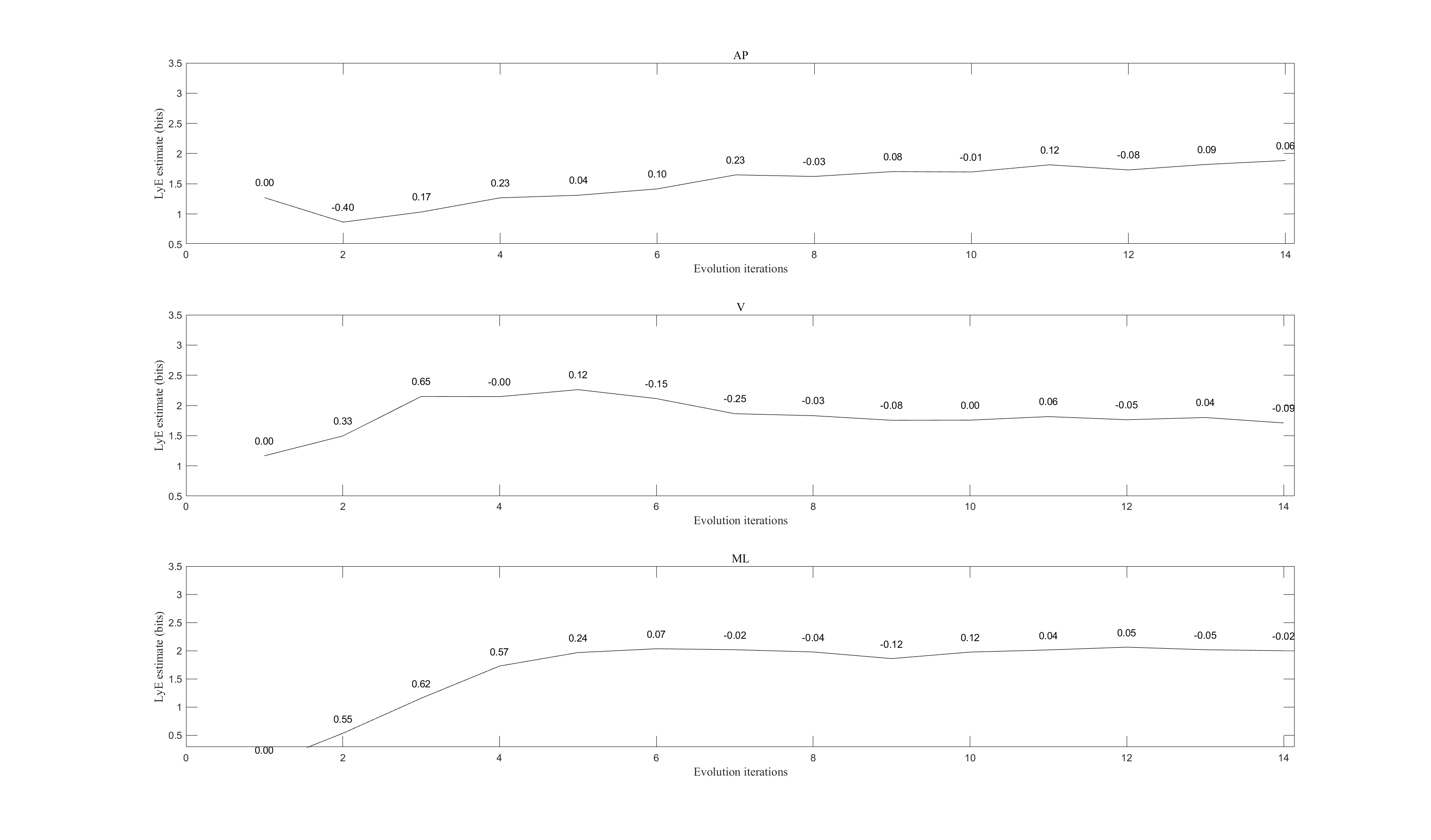

Supplement: Supplementary file 2 — Supplementary Information. [file 41598_2020_79584_MOESM2_ESM.zip › Participant19_trial7.png]

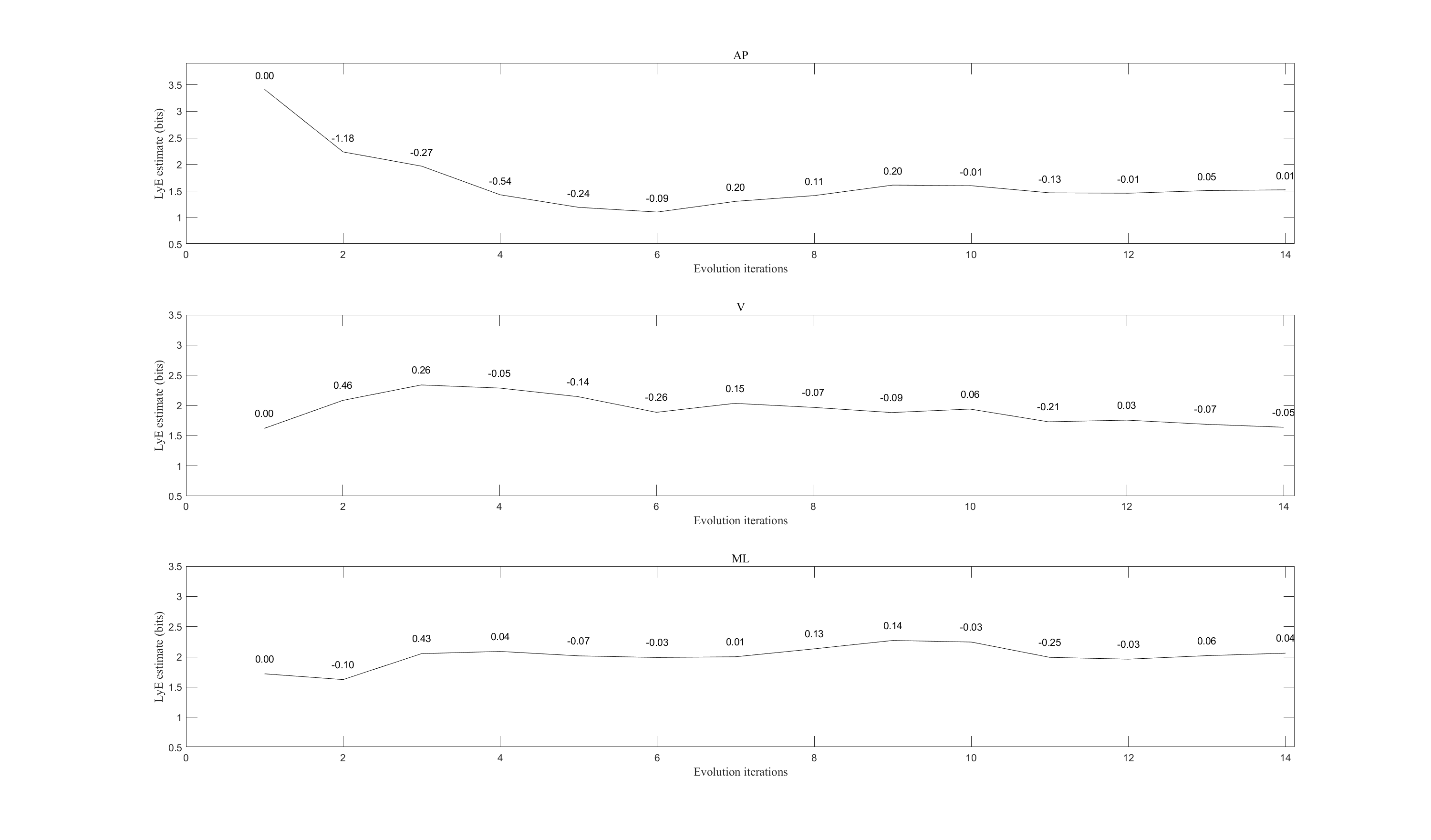

Supplement: Supplementary file 2 — Supplementary Information. [file 41598_2020_79584_MOESM2_ESM.zip › Participant19_trial8.png]

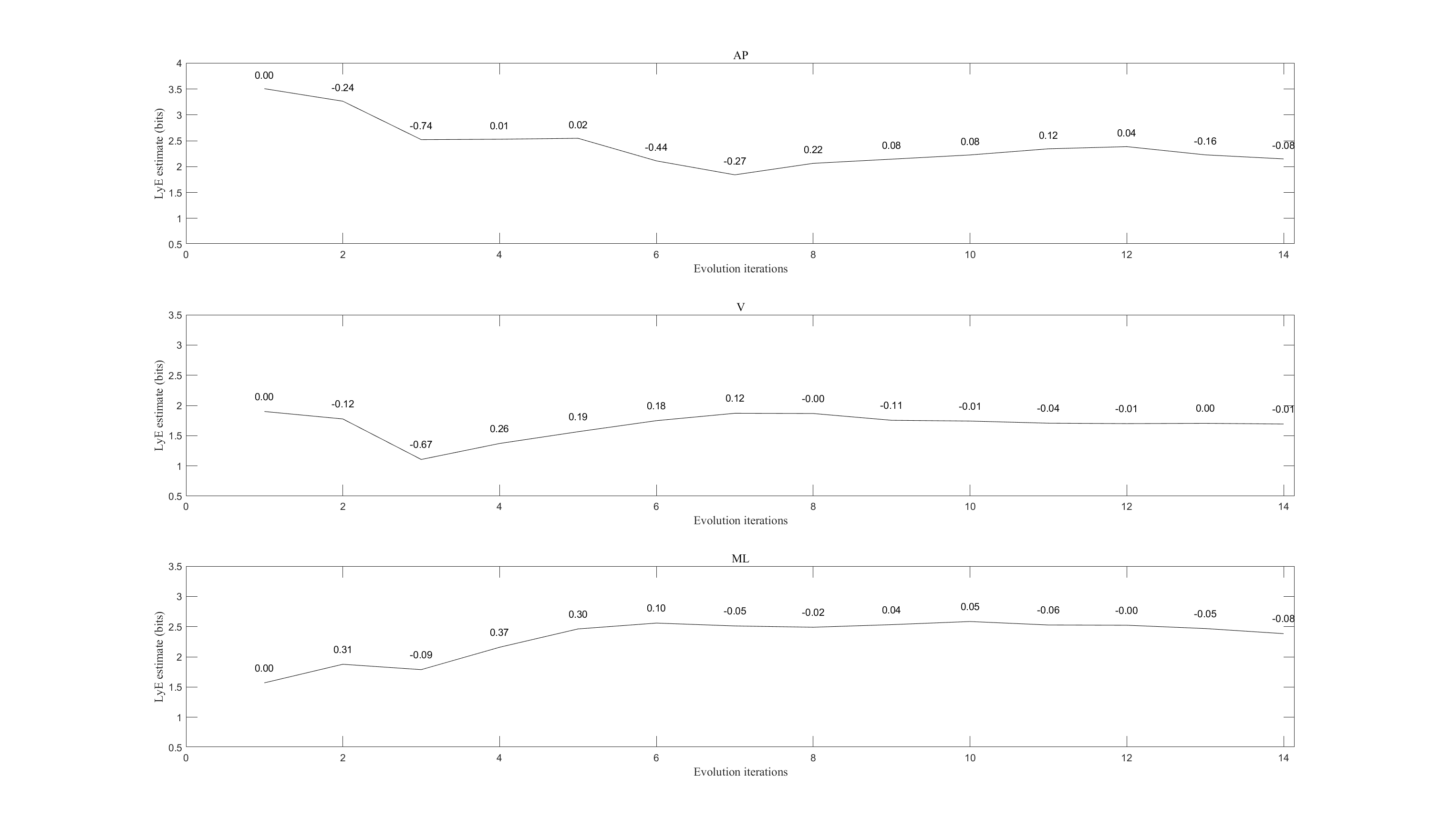

Supplement: Supplementary file 2 — Supplementary Information. [file 41598_2020_79584_MOESM2_ESM.zip › Participant19_trial9.png]

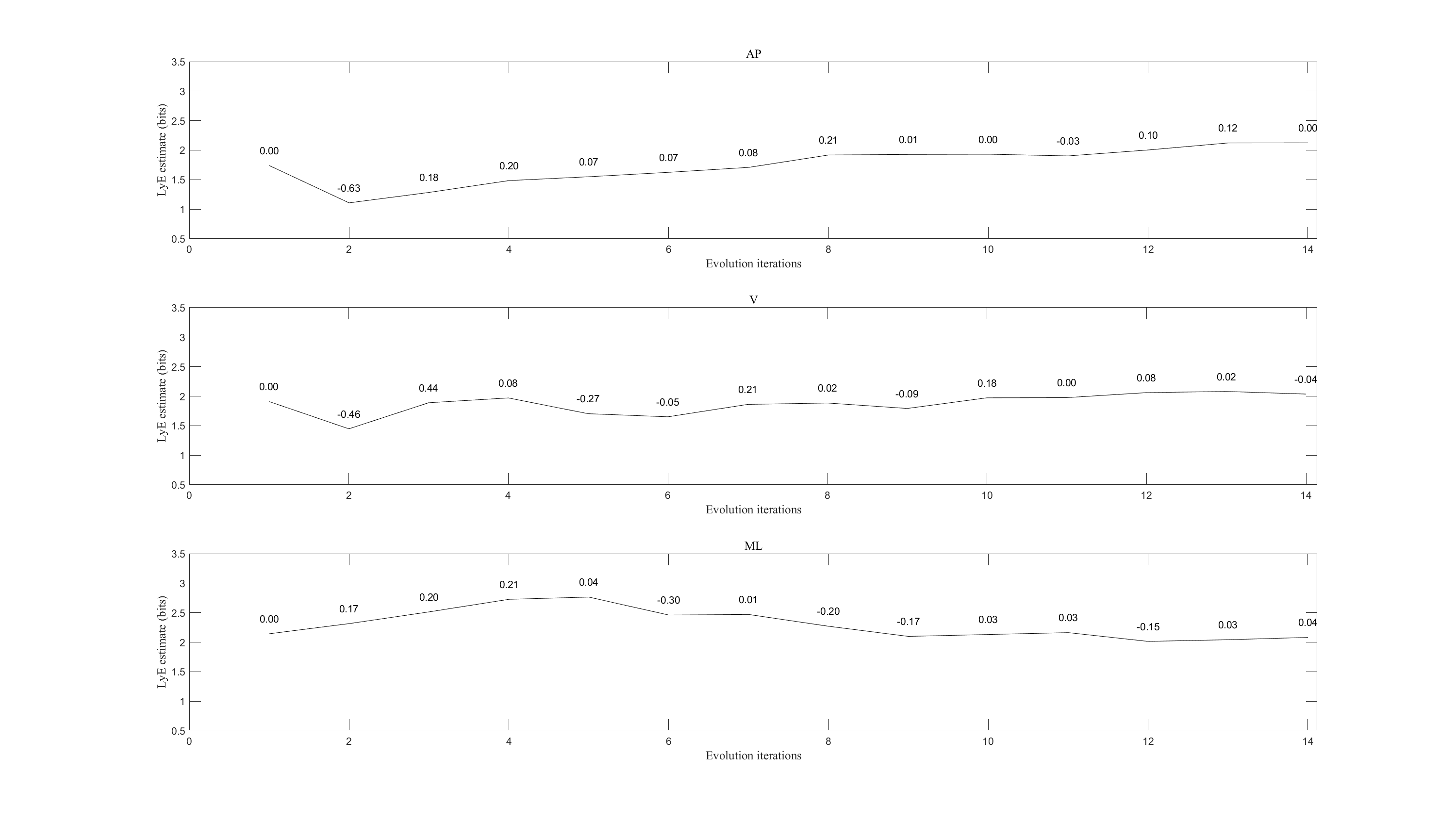

Supplement: Supplementary file 2 — Supplementary Information. [file 41598_2020_79584_MOESM2_ESM.zip › Participant2_trial1.png]

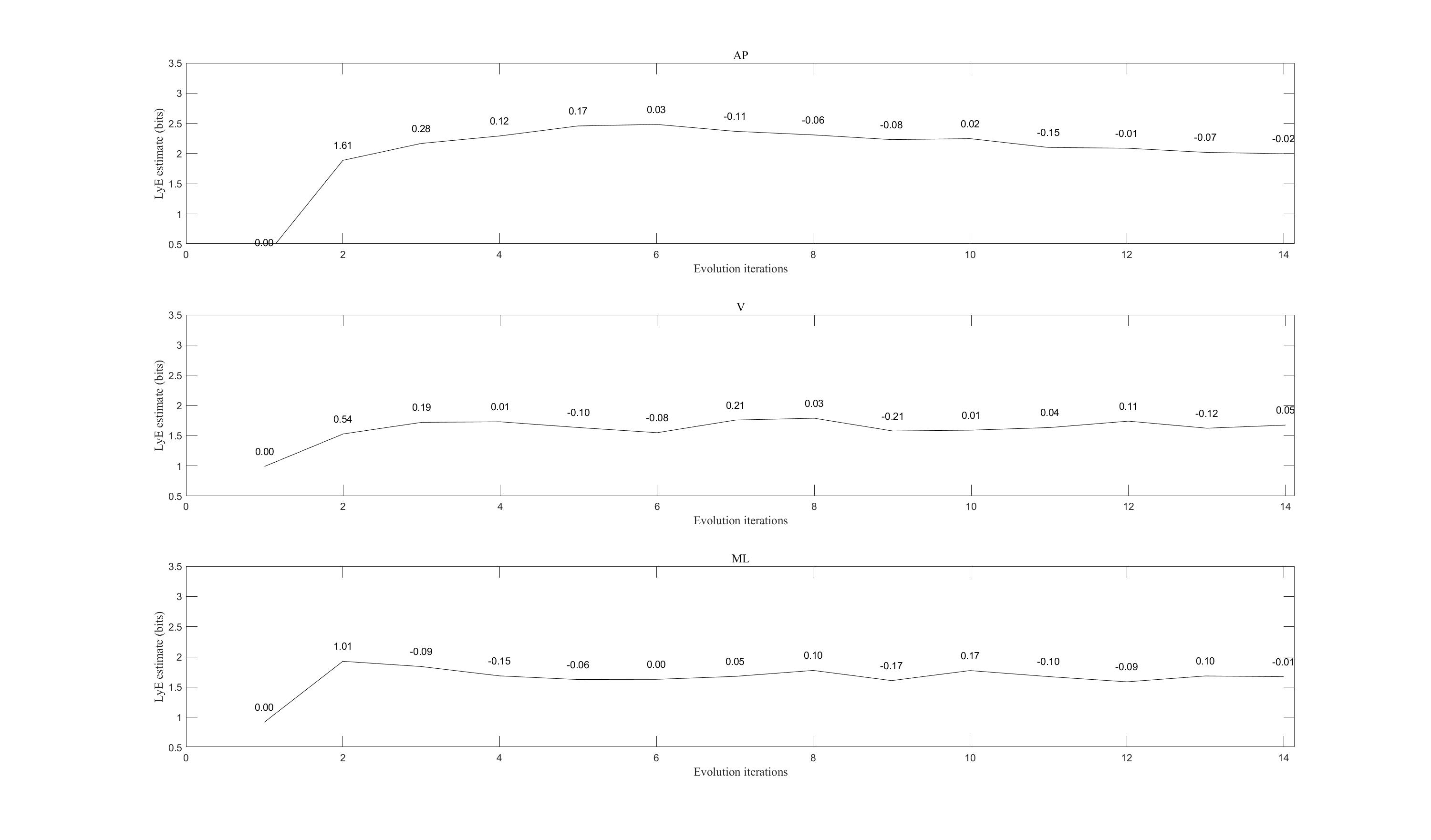

Supplement: Supplementary file 2 — Supplementary Information. [file 41598_2020_79584_MOESM2_ESM.zip › Participant2_trial10.png]

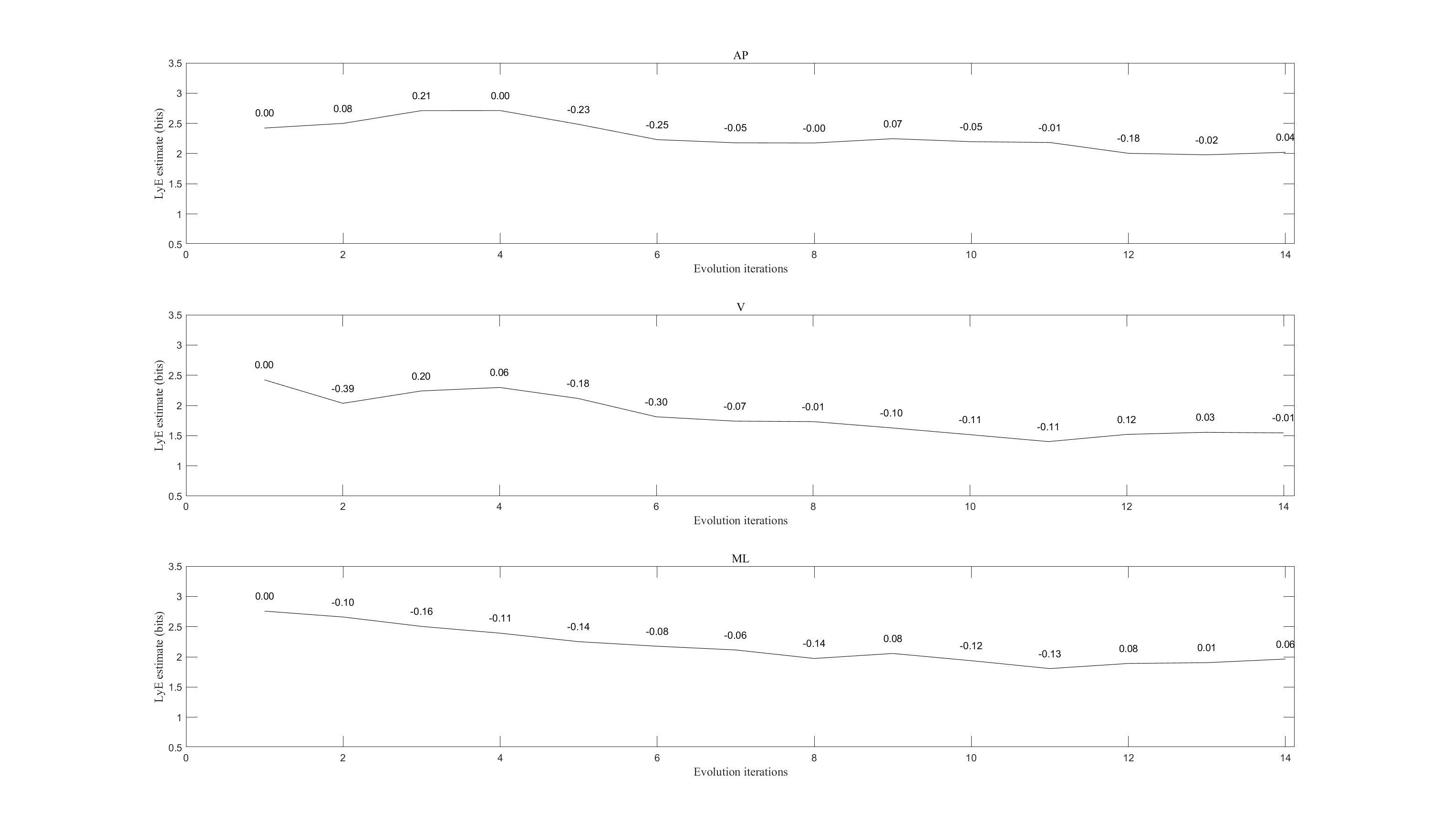

Supplement: Supplementary file 2 — Supplementary Information. [file 41598_2020_79584_MOESM2_ESM.zip › Participant2_trial11.png]

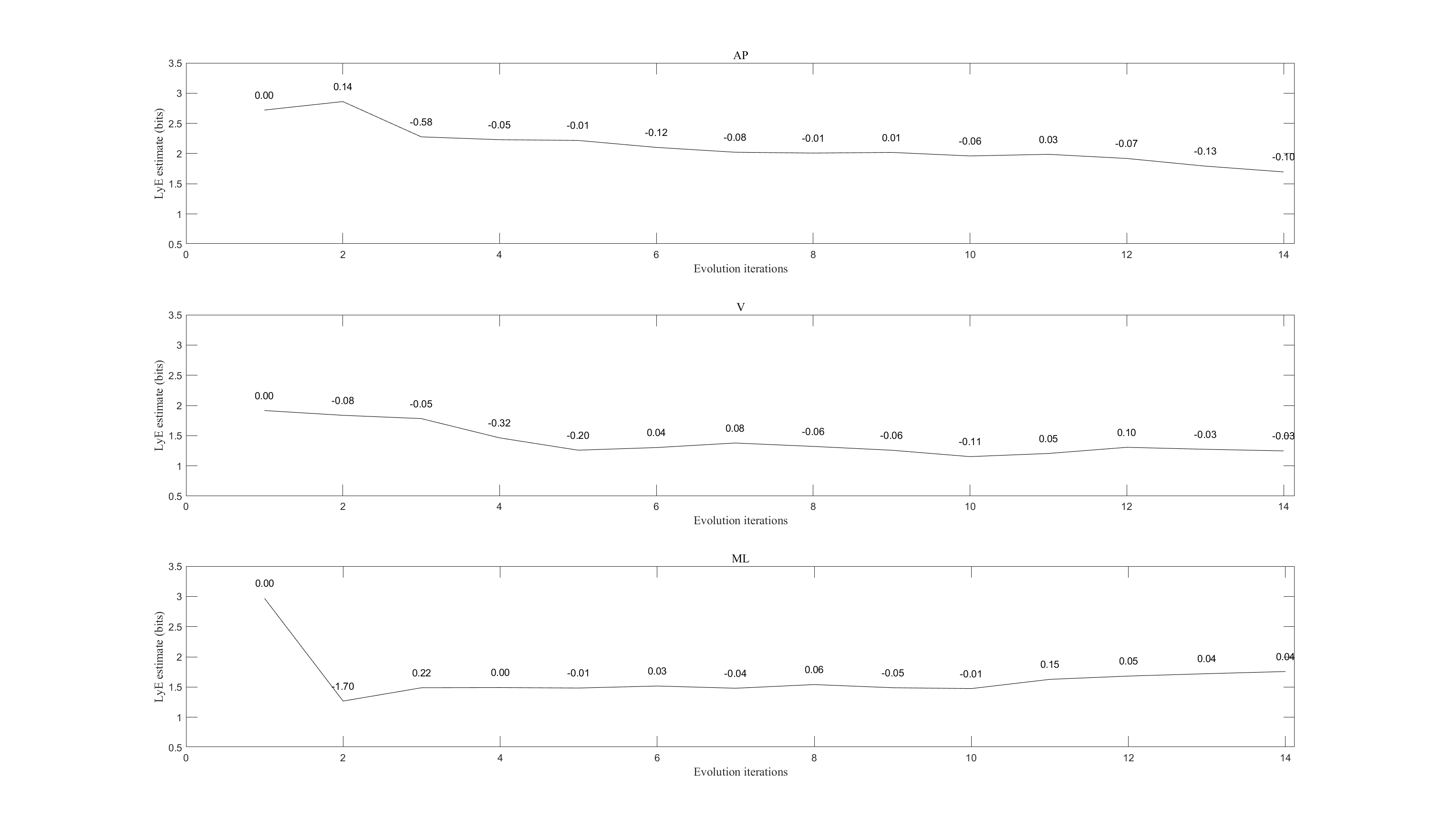

Supplement: Supplementary file 2 — Supplementary Information. [file 41598_2020_79584_MOESM2_ESM.zip › Participant2_trial12.png]

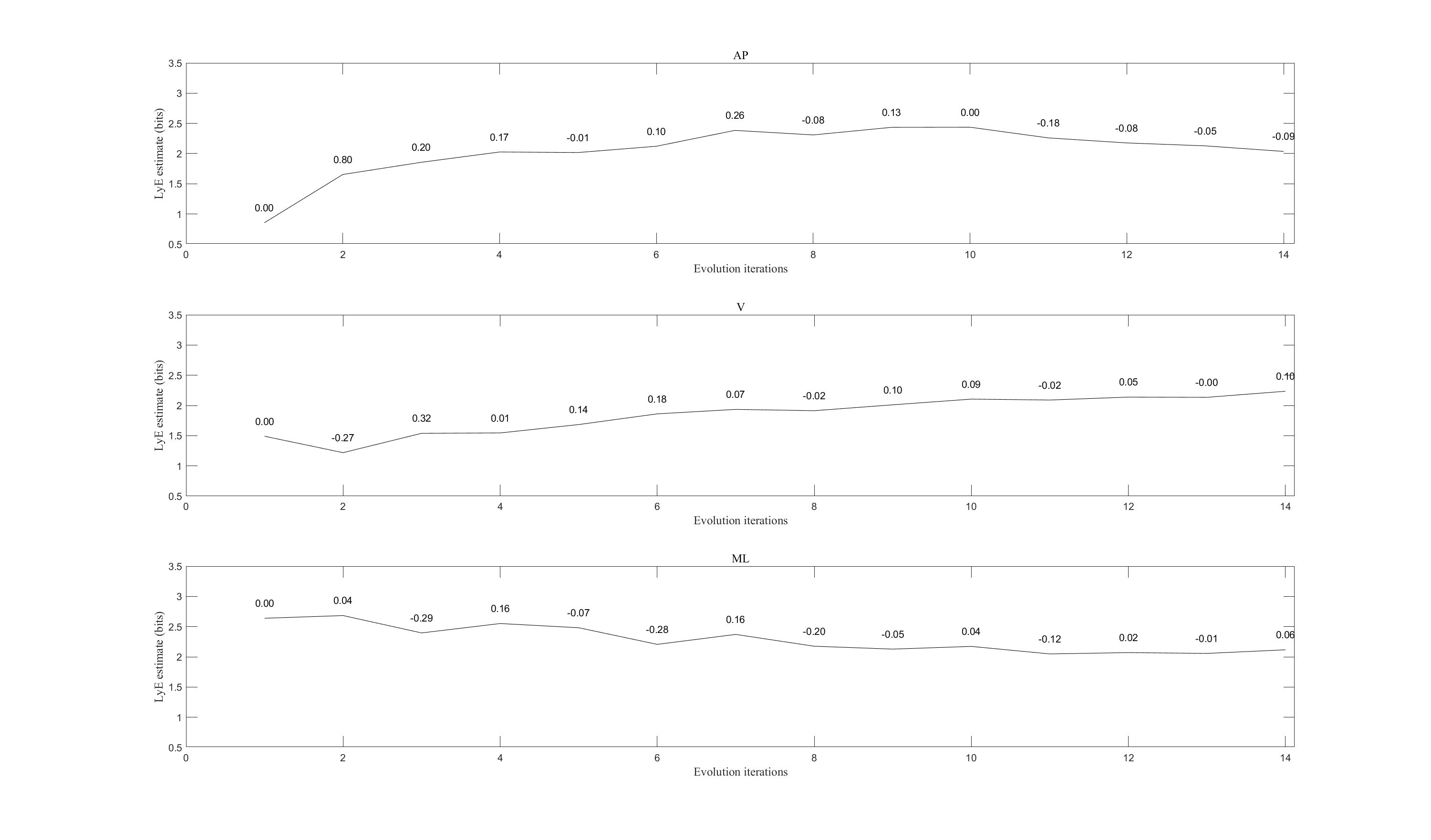

Supplement: Supplementary file 2 — Supplementary Information. [file 41598_2020_79584_MOESM2_ESM.zip › Participant2_trial2.png]

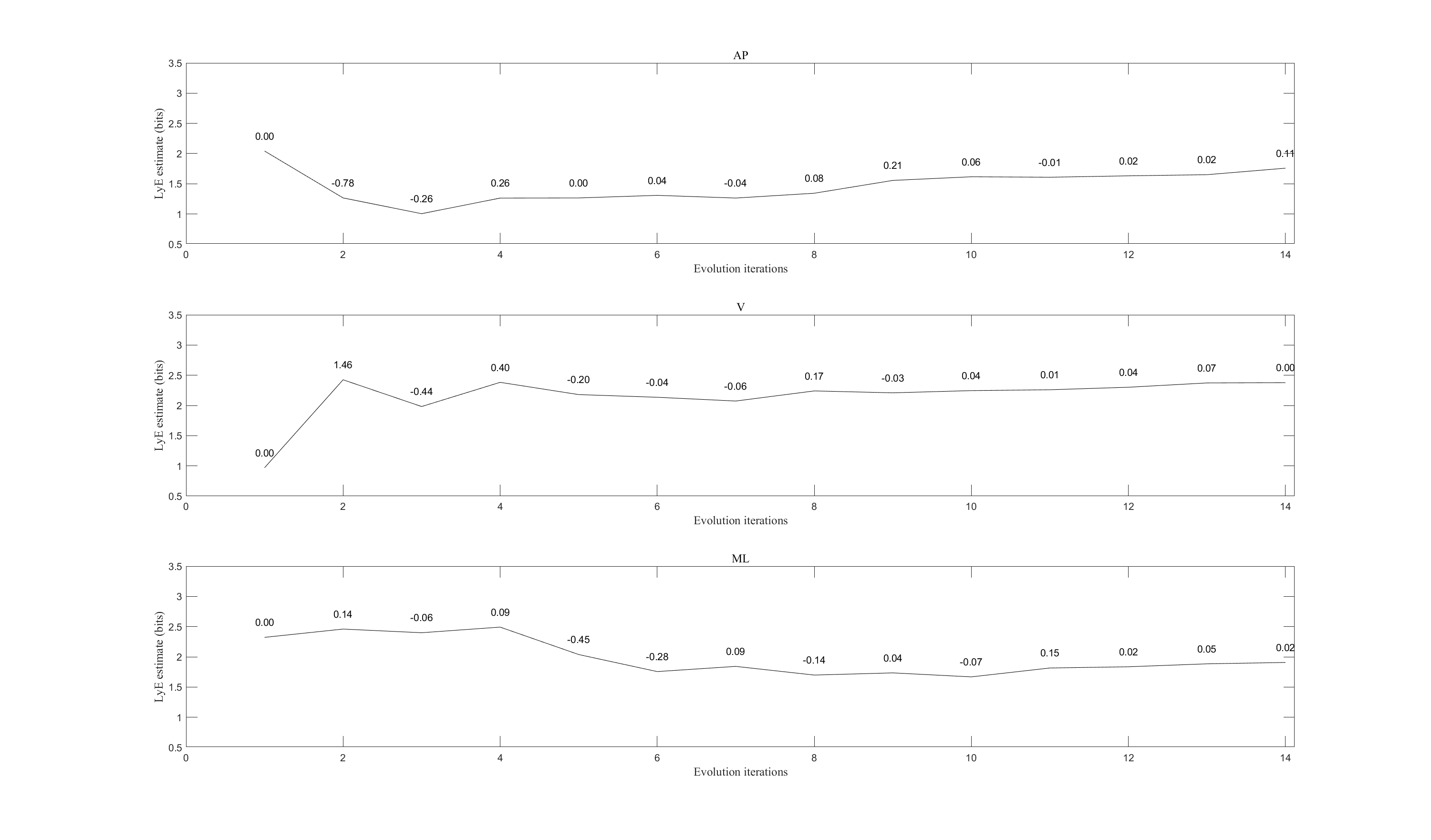

Supplement: Supplementary file 2 — Supplementary Information. [file 41598_2020_79584_MOESM2_ESM.zip › Participant2_trial3.png]

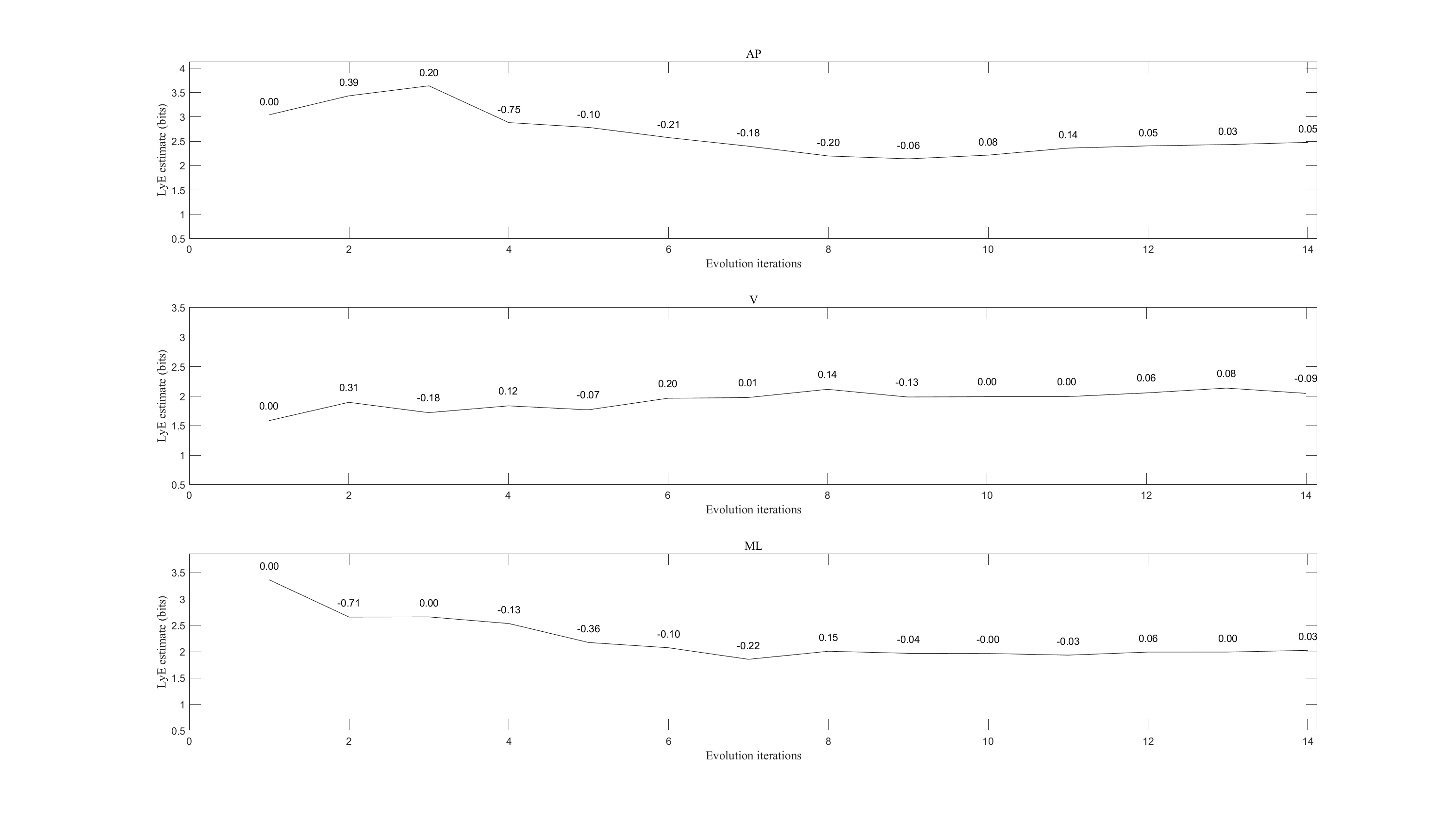

Supplement: Supplementary file 2 — Supplementary Information. [file 41598_2020_79584_MOESM2_ESM.zip › Participant2_trial4.png]

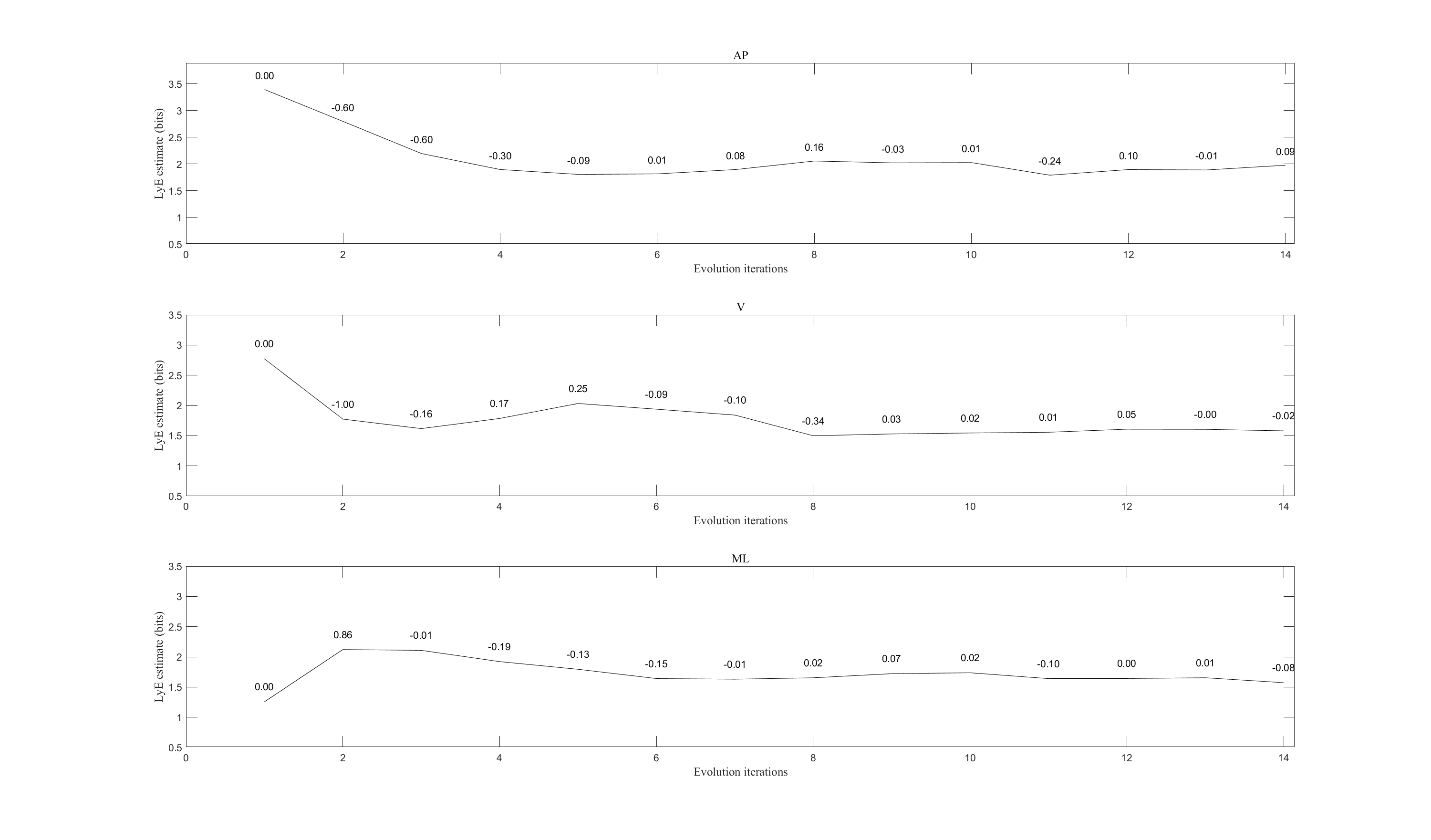

Supplement: Supplementary file 2 — Supplementary Information. [file 41598_2020_79584_MOESM2_ESM.zip › Participant2_trial5.png]

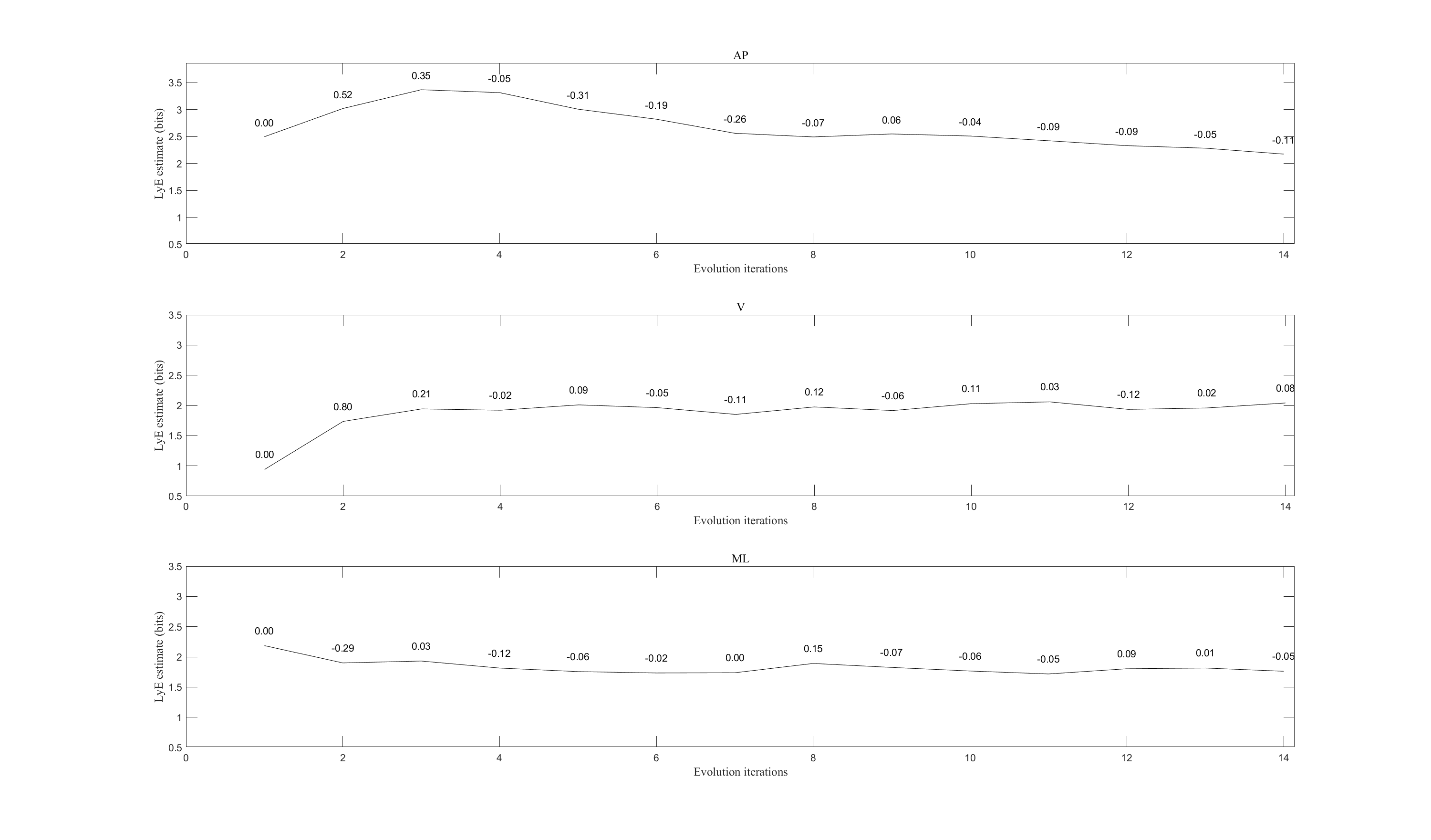

Supplement: Supplementary file 2 — Supplementary Information. [file 41598_2020_79584_MOESM2_ESM.zip › Participant2_trial6.png]

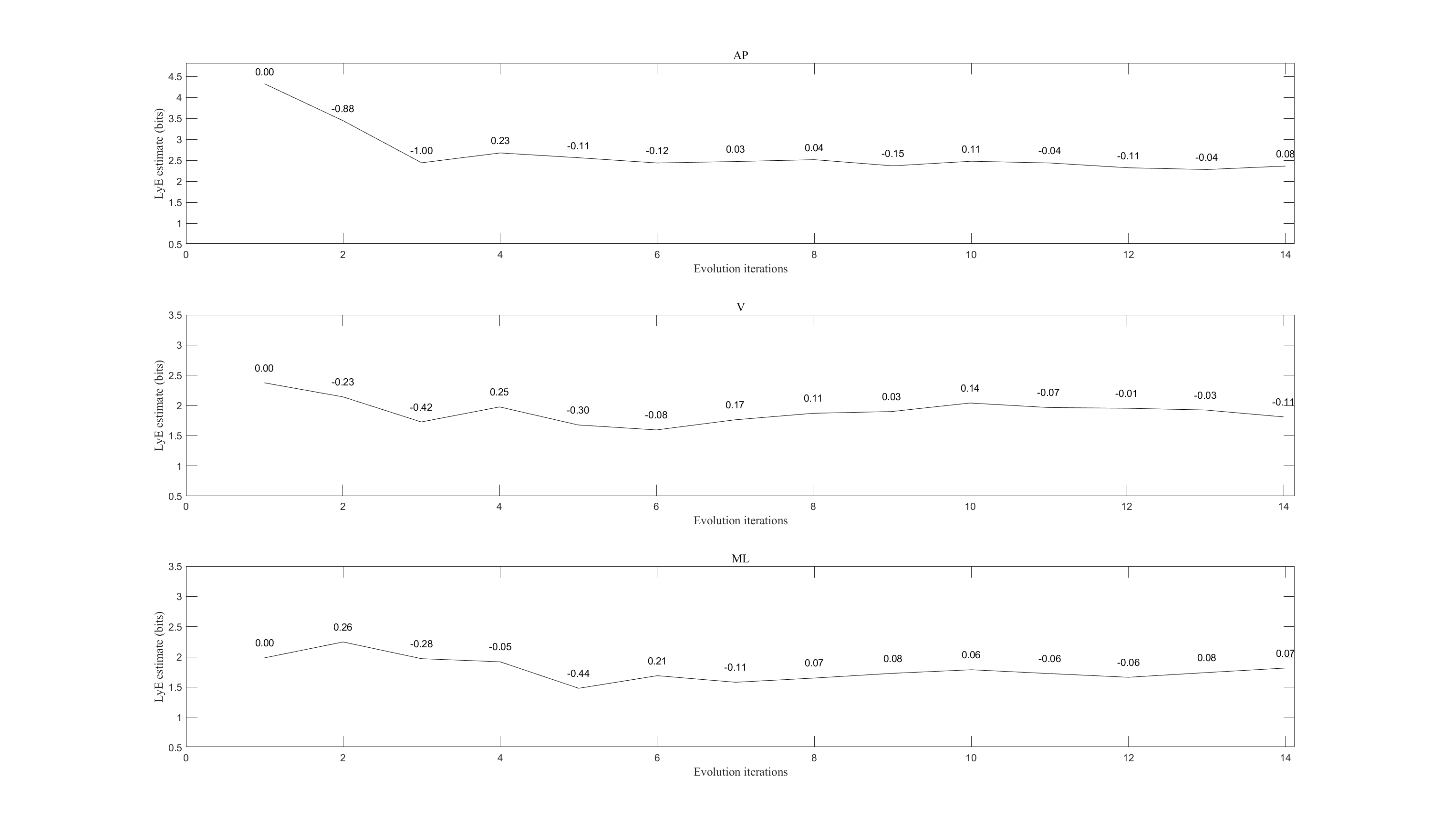

Supplement: Supplementary file 2 — Supplementary Information. [file 41598_2020_79584_MOESM2_ESM.zip › Participant2_trial7.png]

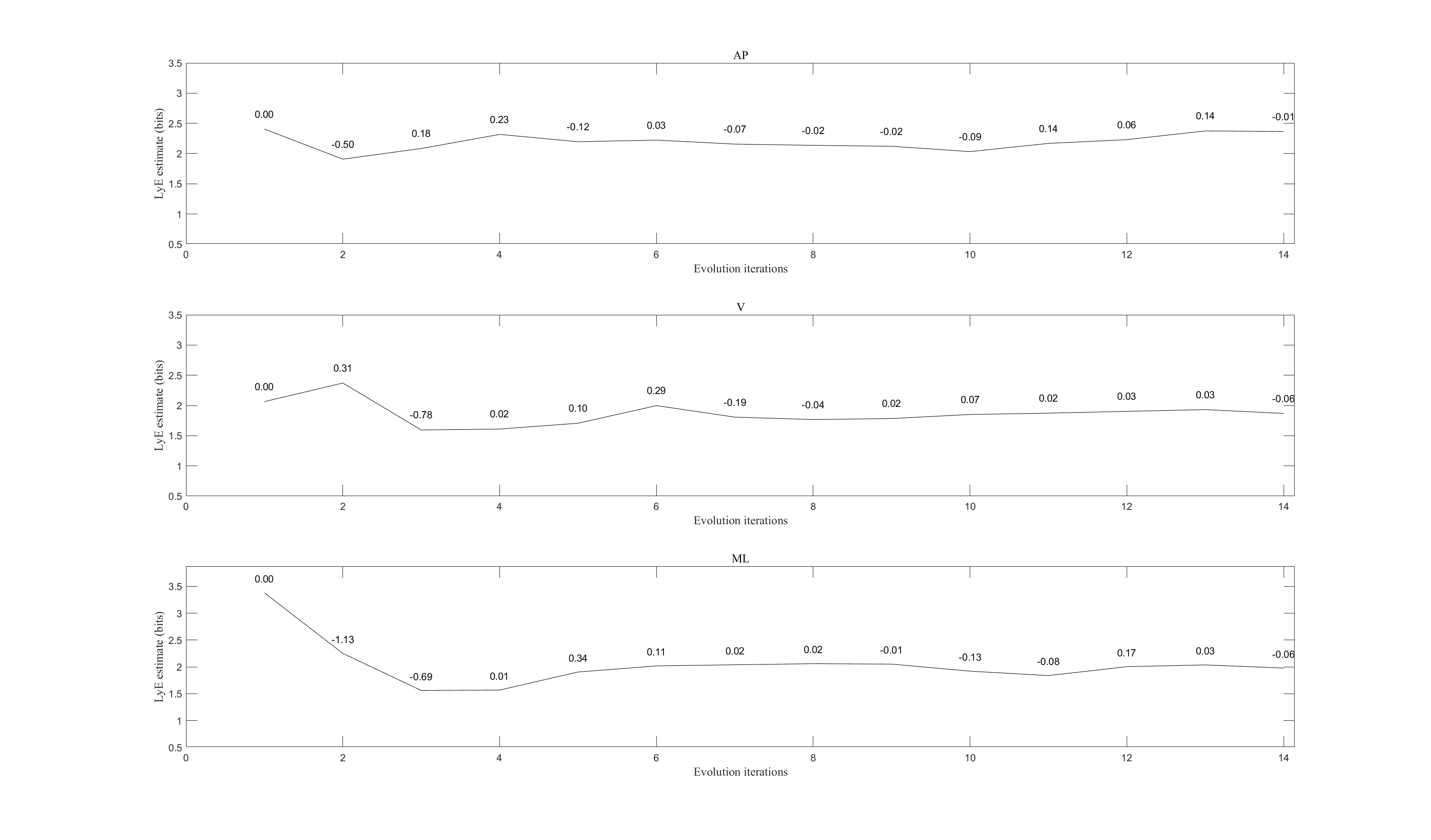

Supplement: Supplementary file 2 — Supplementary Information. [file 41598_2020_79584_MOESM2_ESM.zip › Participant2_trial8.png]

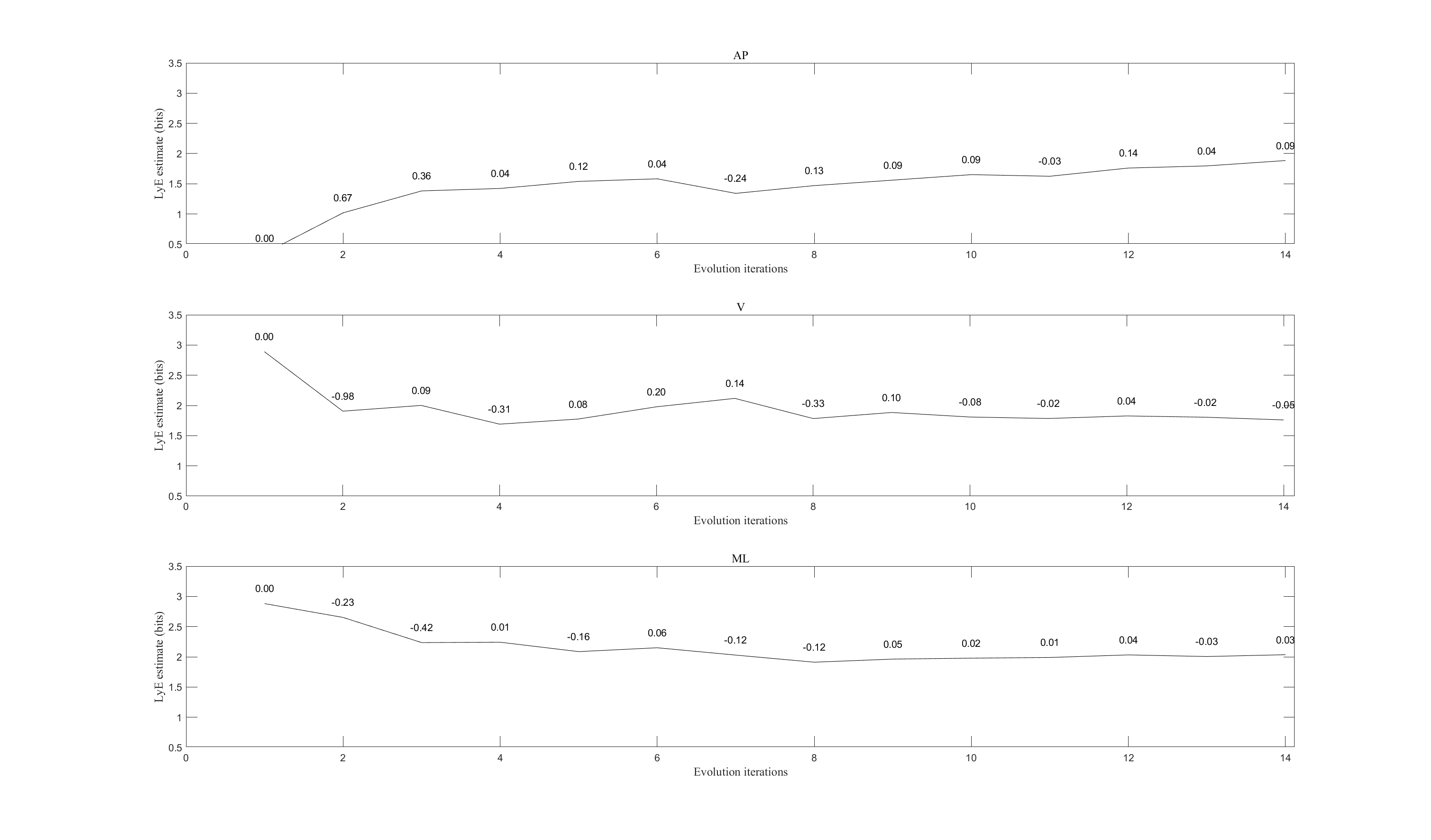

Supplement: Supplementary file 2 — Supplementary Information. [file 41598_2020_79584_MOESM2_ESM.zip › Participant2_trial9.png]

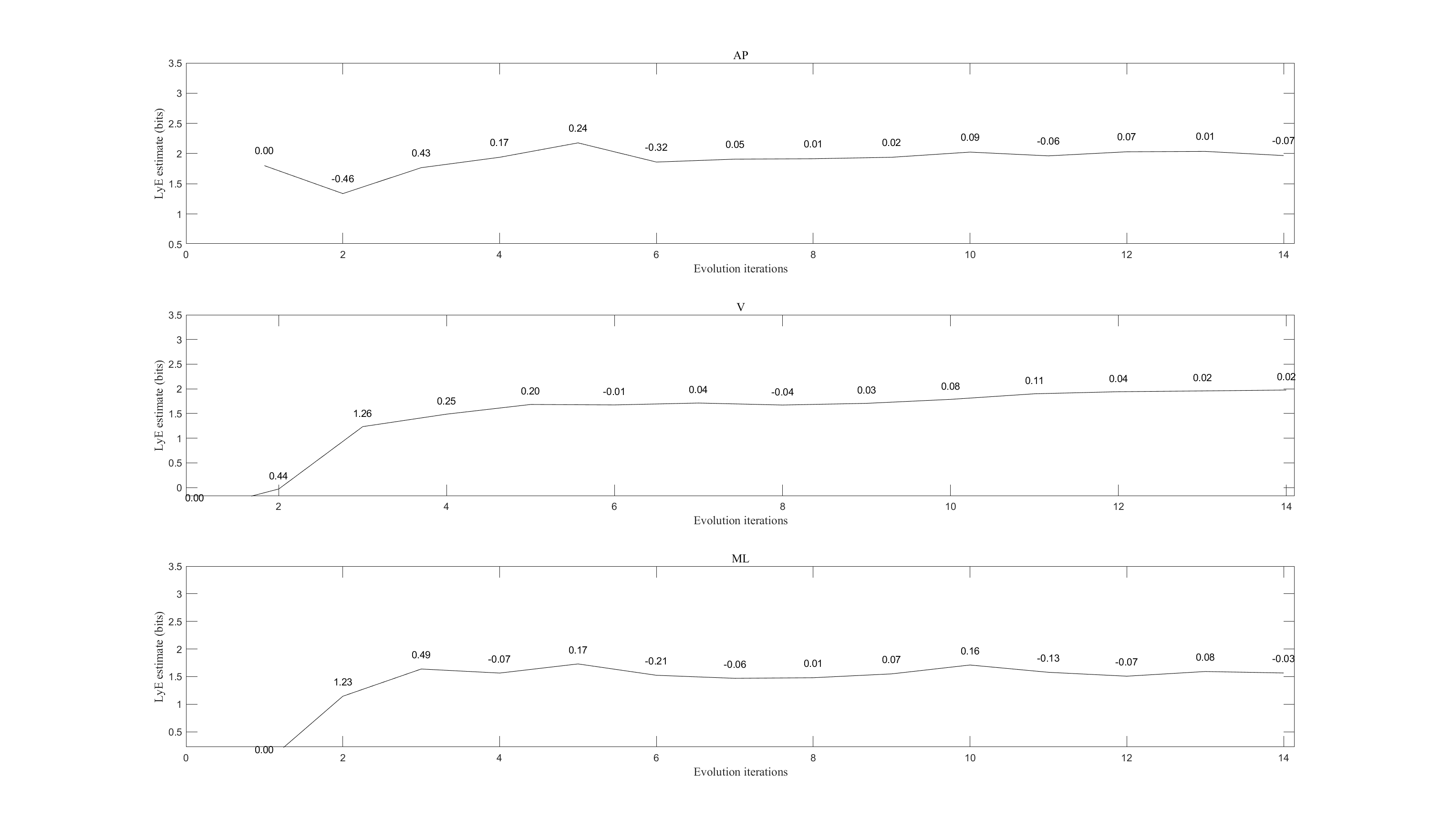

Supplement: Supplementary file 2 — Supplementary Information. [file 41598_2020_79584_MOESM2_ESM.zip › Participant20_trial1.png]

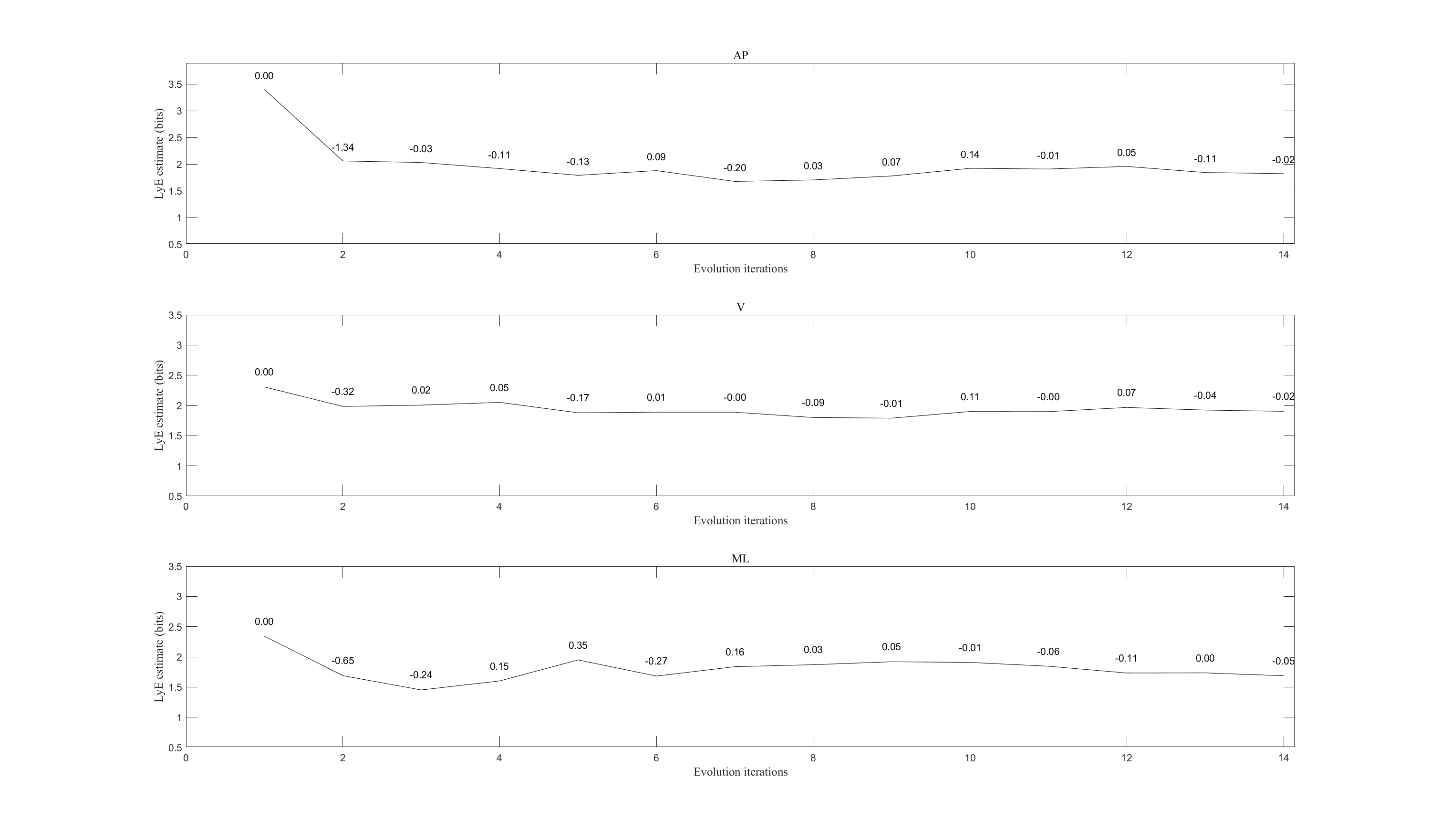

Supplement: Supplementary file 2 — Supplementary Information. [file 41598_2020_79584_MOESM2_ESM.zip › Participant20_trial10.png]

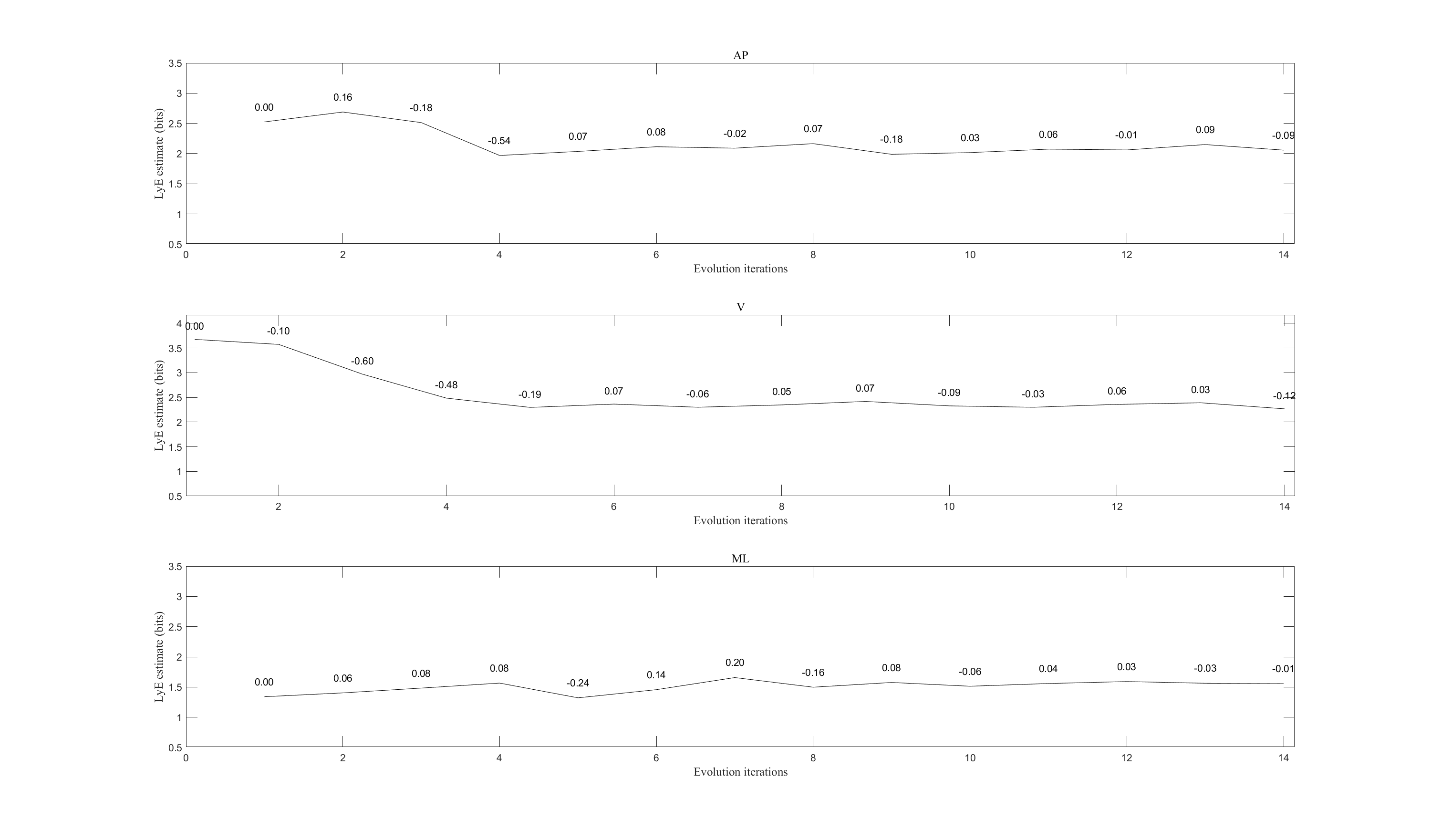

Supplement: Supplementary file 2 — Supplementary Information. [file 41598_2020_79584_MOESM2_ESM.zip › Participant20_trial11.png]

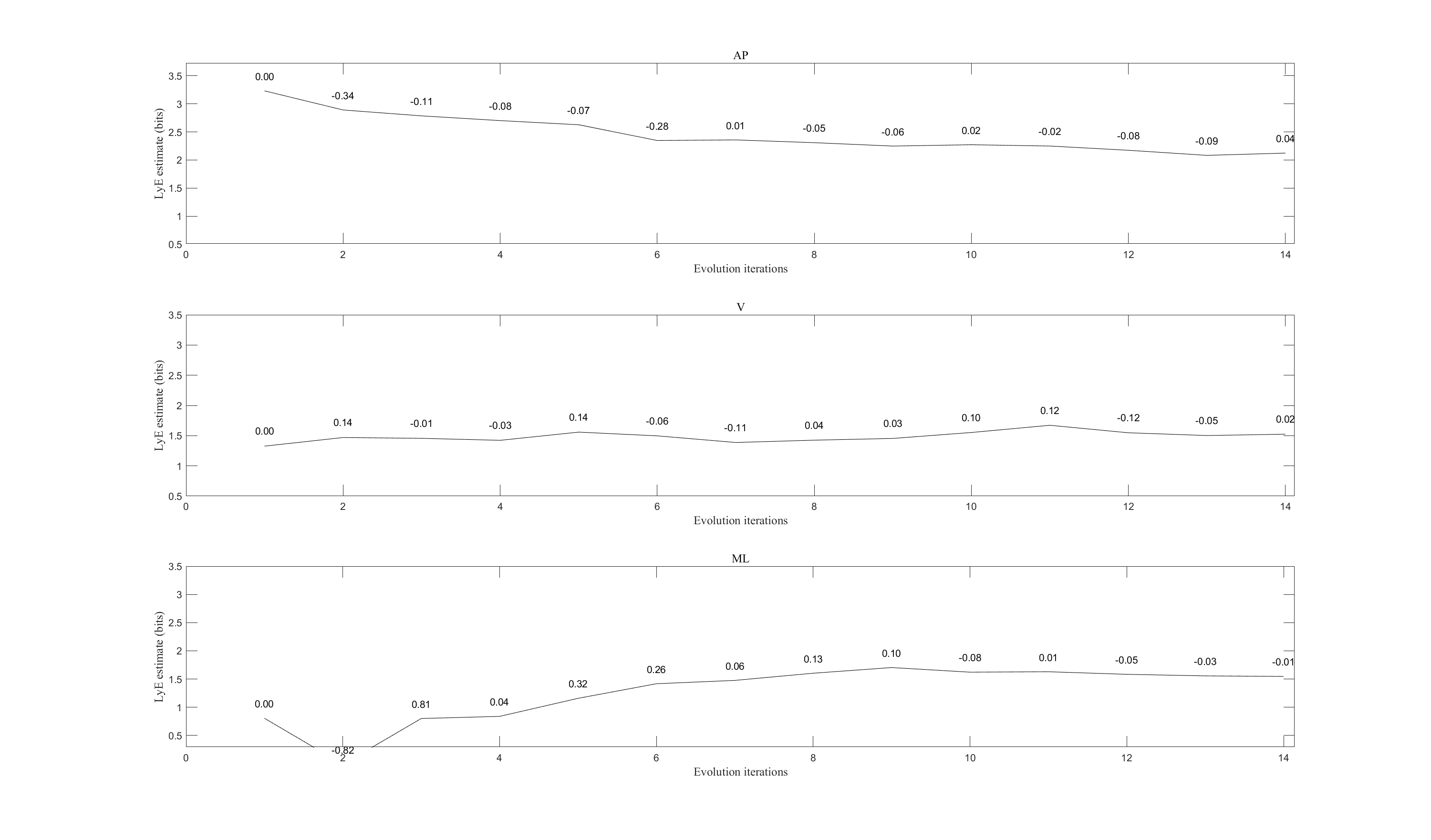

Supplement: Supplementary file 2 — Supplementary Information. [file 41598_2020_79584_MOESM2_ESM.zip › Participant20_trial12.png]

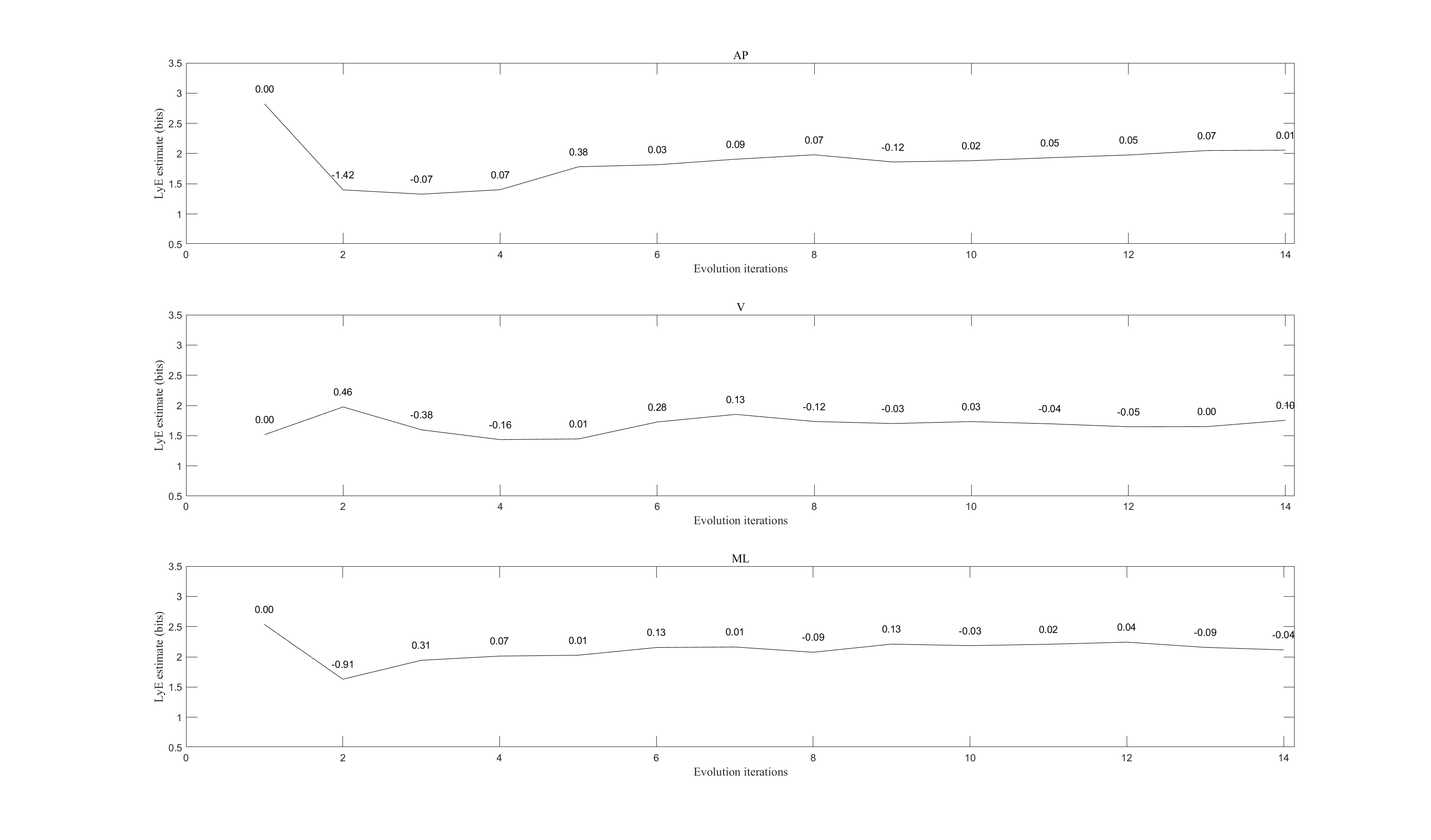

Supplement: Supplementary file 2 — Supplementary Information. [file 41598_2020_79584_MOESM2_ESM.zip › Participant20_trial2.png]

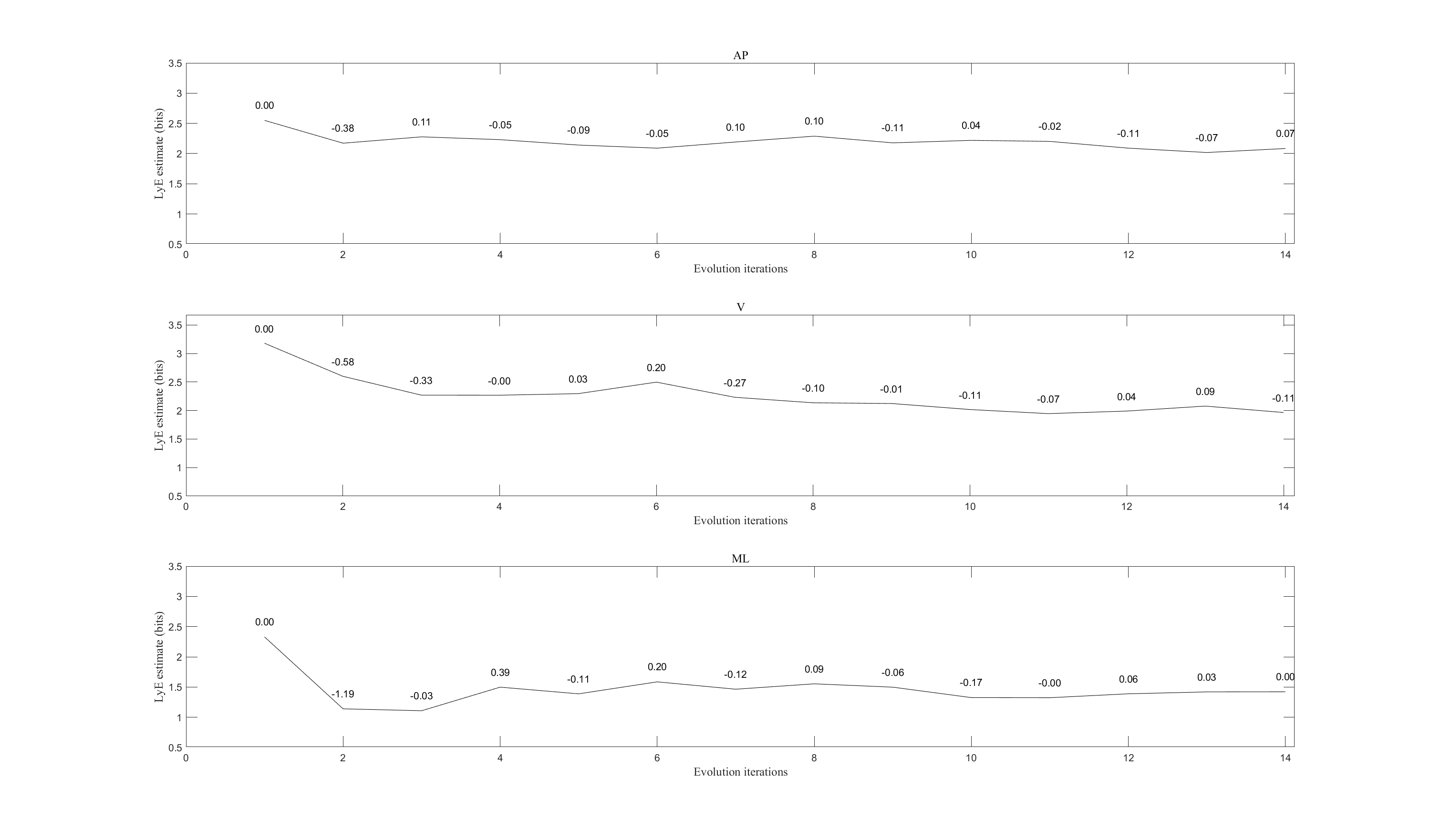

Supplement: Supplementary file 2 — Supplementary Information. [file 41598_2020_79584_MOESM2_ESM.zip › Participant20_trial3.png]

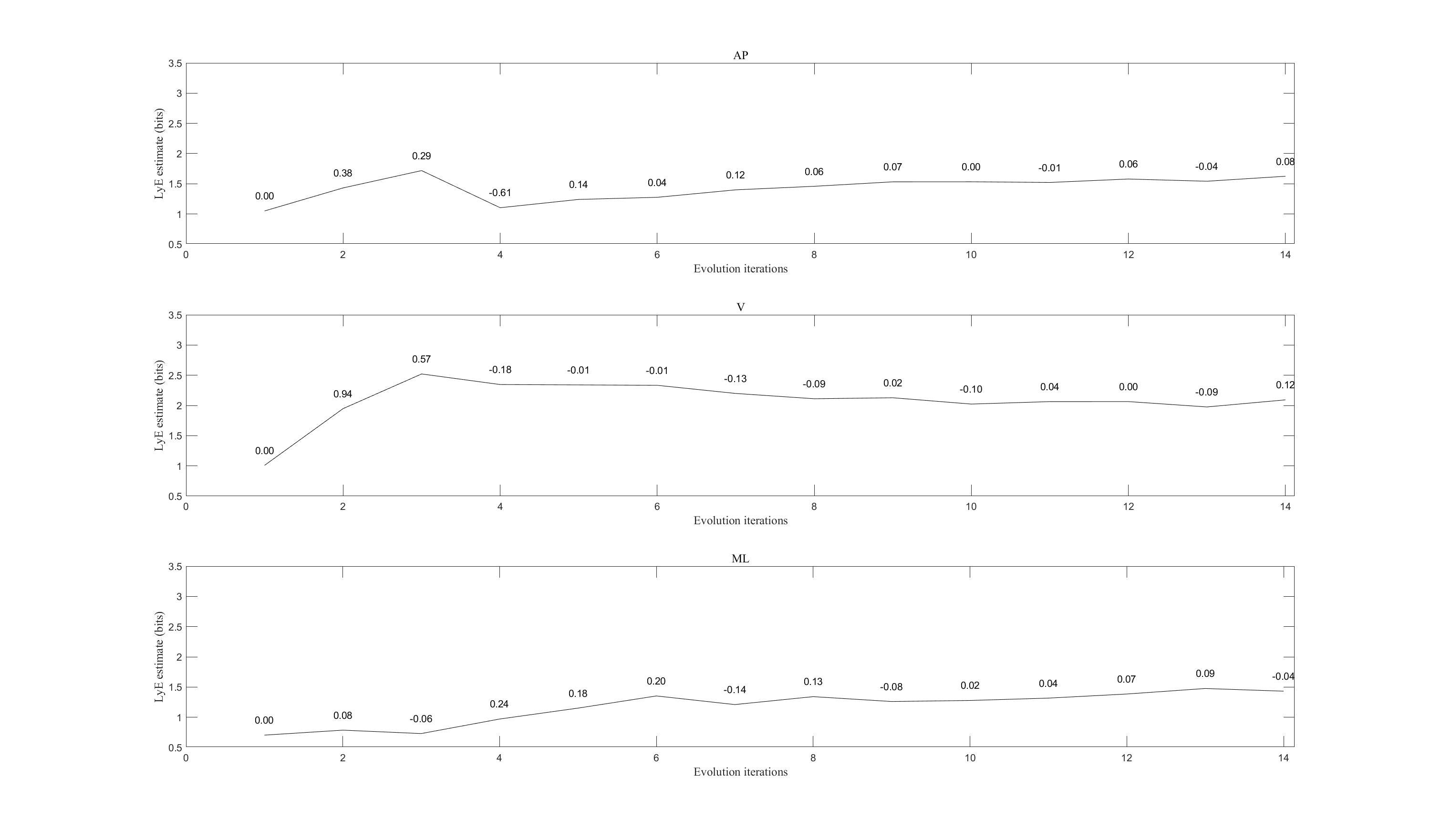

Supplement: Supplementary file 2 — Supplementary Information. [file 41598_2020_79584_MOESM2_ESM.zip › Participant20_trial4.png]

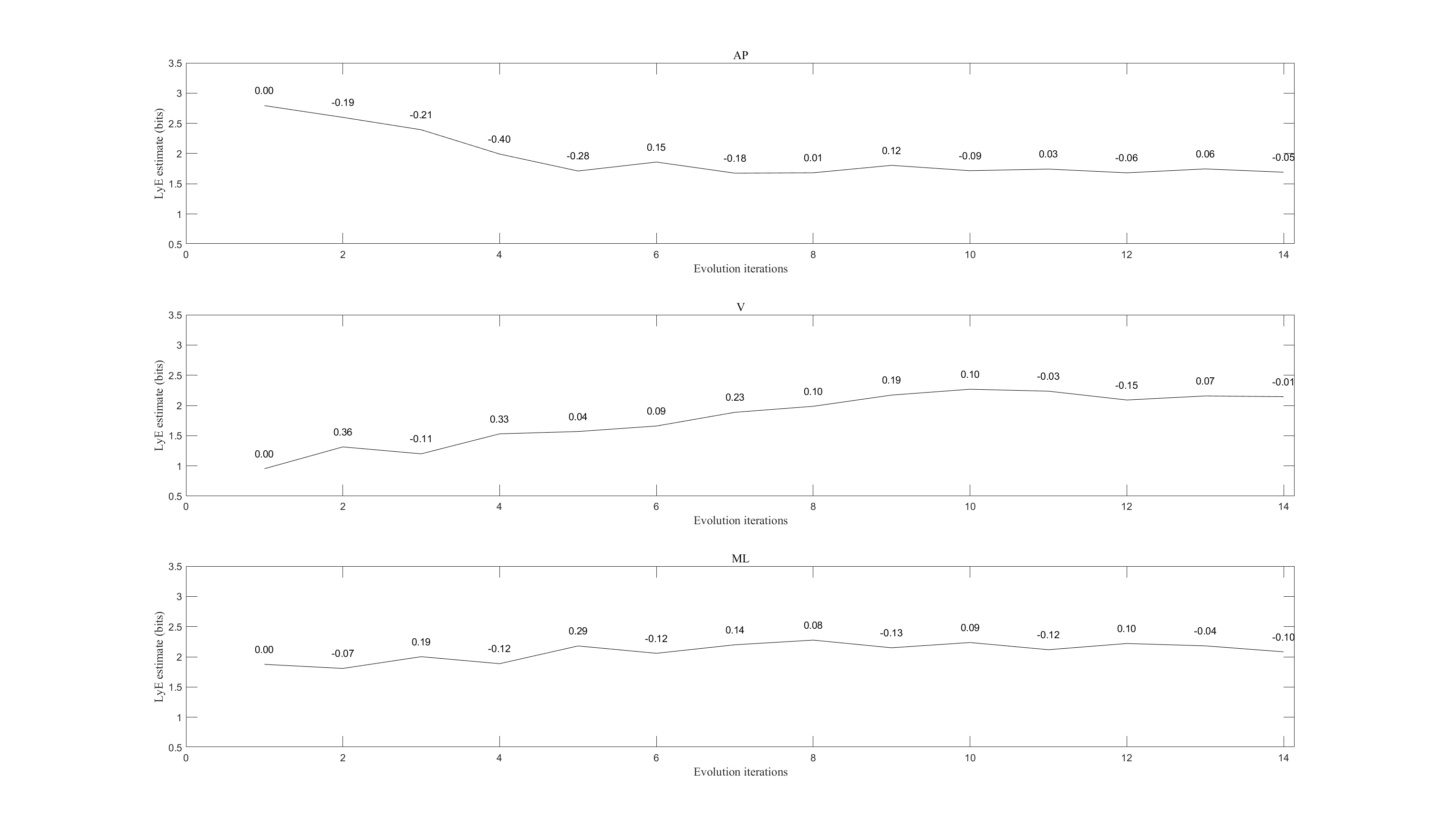

Supplement: Supplementary file 2 — Supplementary Information. [file 41598_2020_79584_MOESM2_ESM.zip › Participant20_trial5.png]

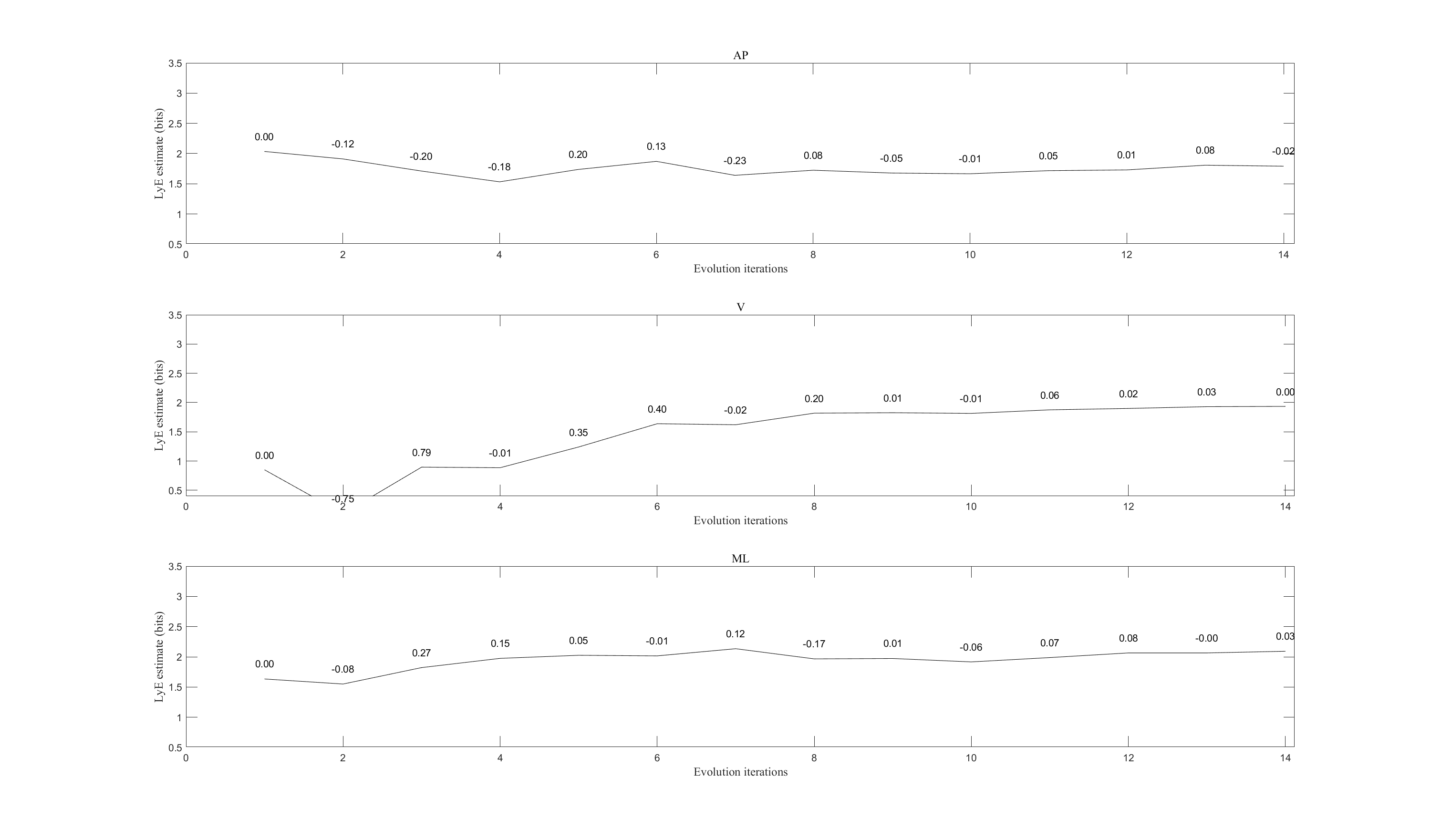

Supplement: Supplementary file 2 — Supplementary Information. [file 41598_2020_79584_MOESM2_ESM.zip › Participant20_trial6.png]

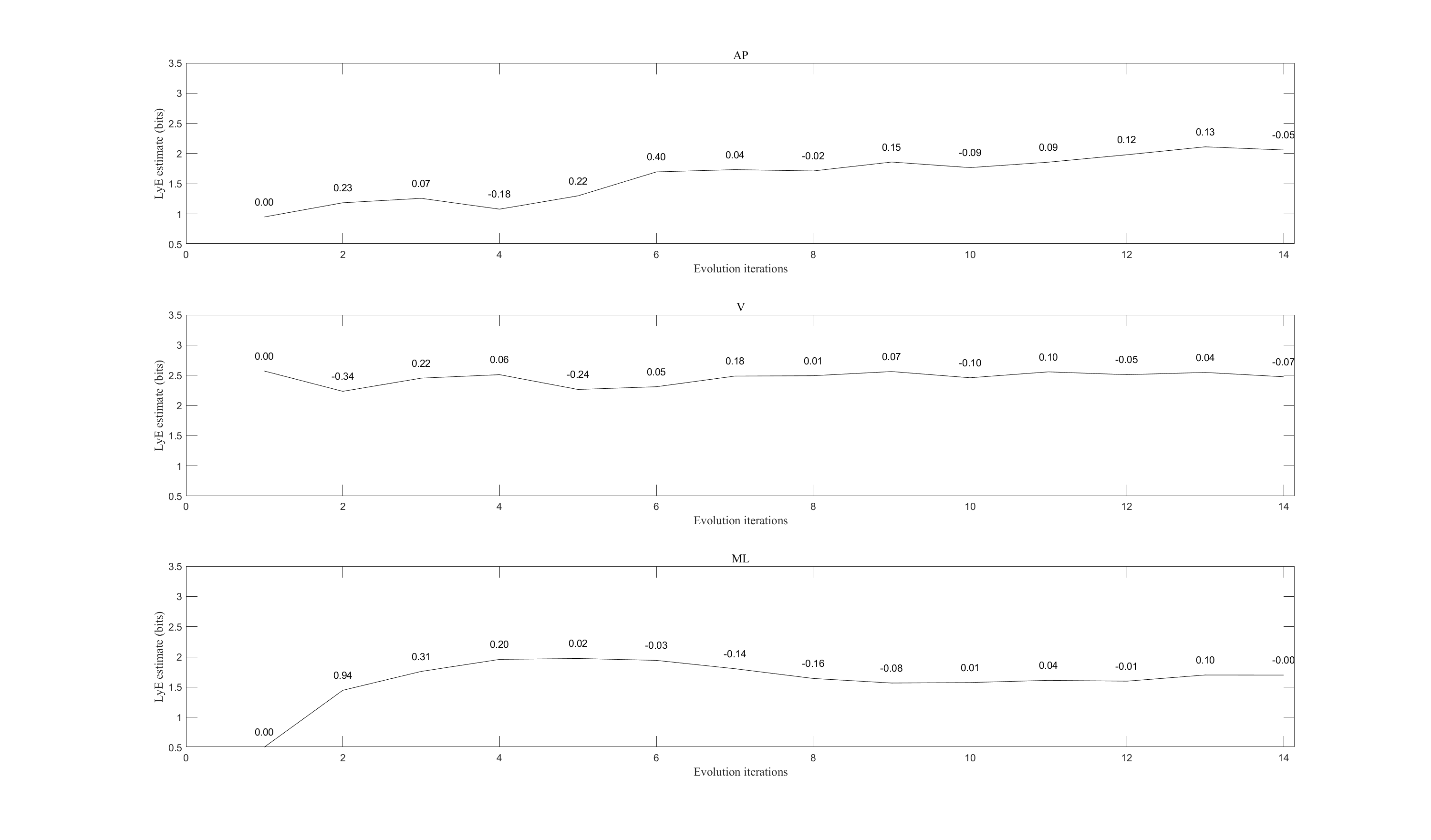

Supplement: Supplementary file 2 — Supplementary Information. [file 41598_2020_79584_MOESM2_ESM.zip › Participant20_trial7.png]

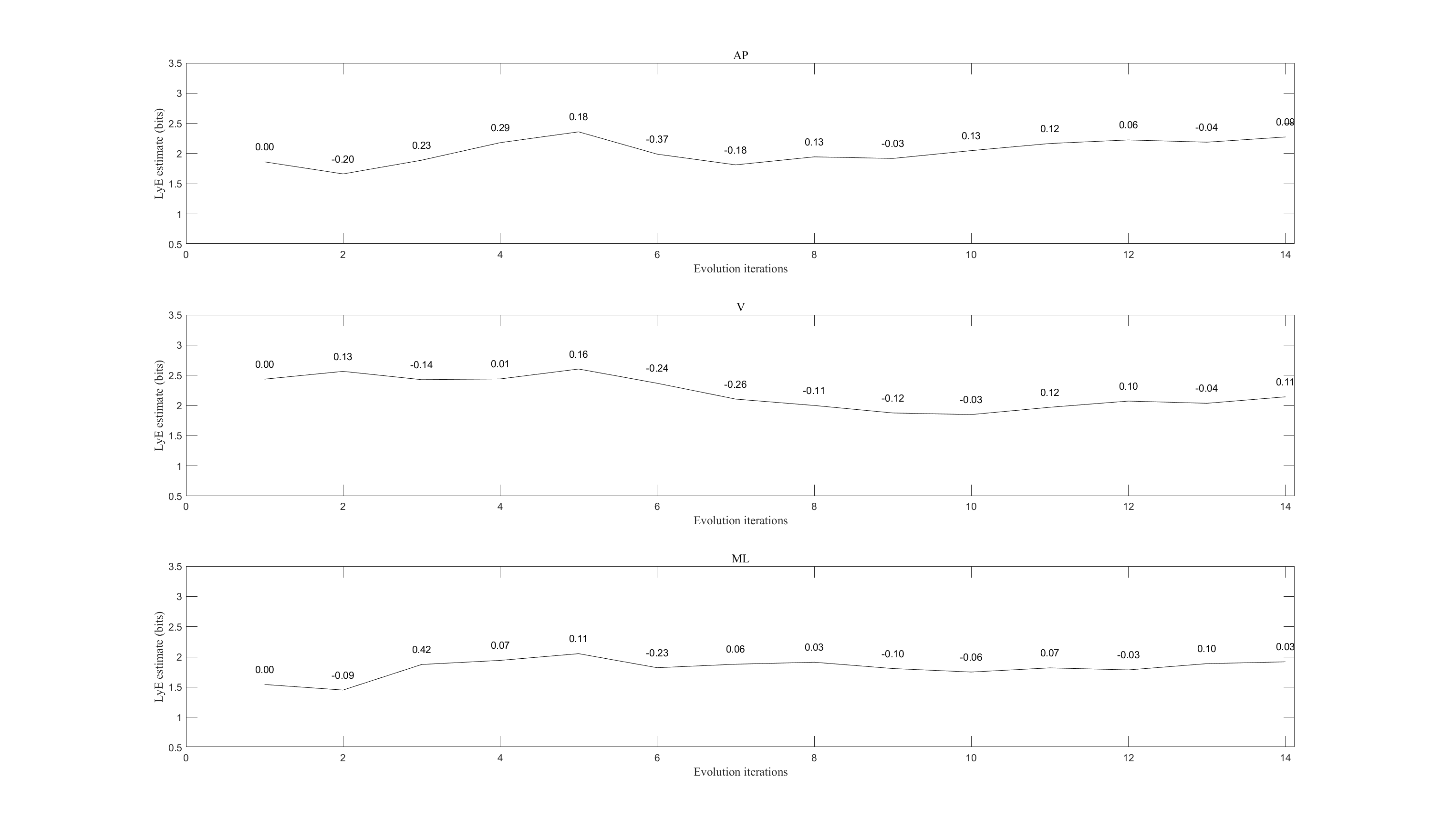

Supplement: Supplementary file 2 — Supplementary Information. [file 41598_2020_79584_MOESM2_ESM.zip › Participant20_trial8.png]

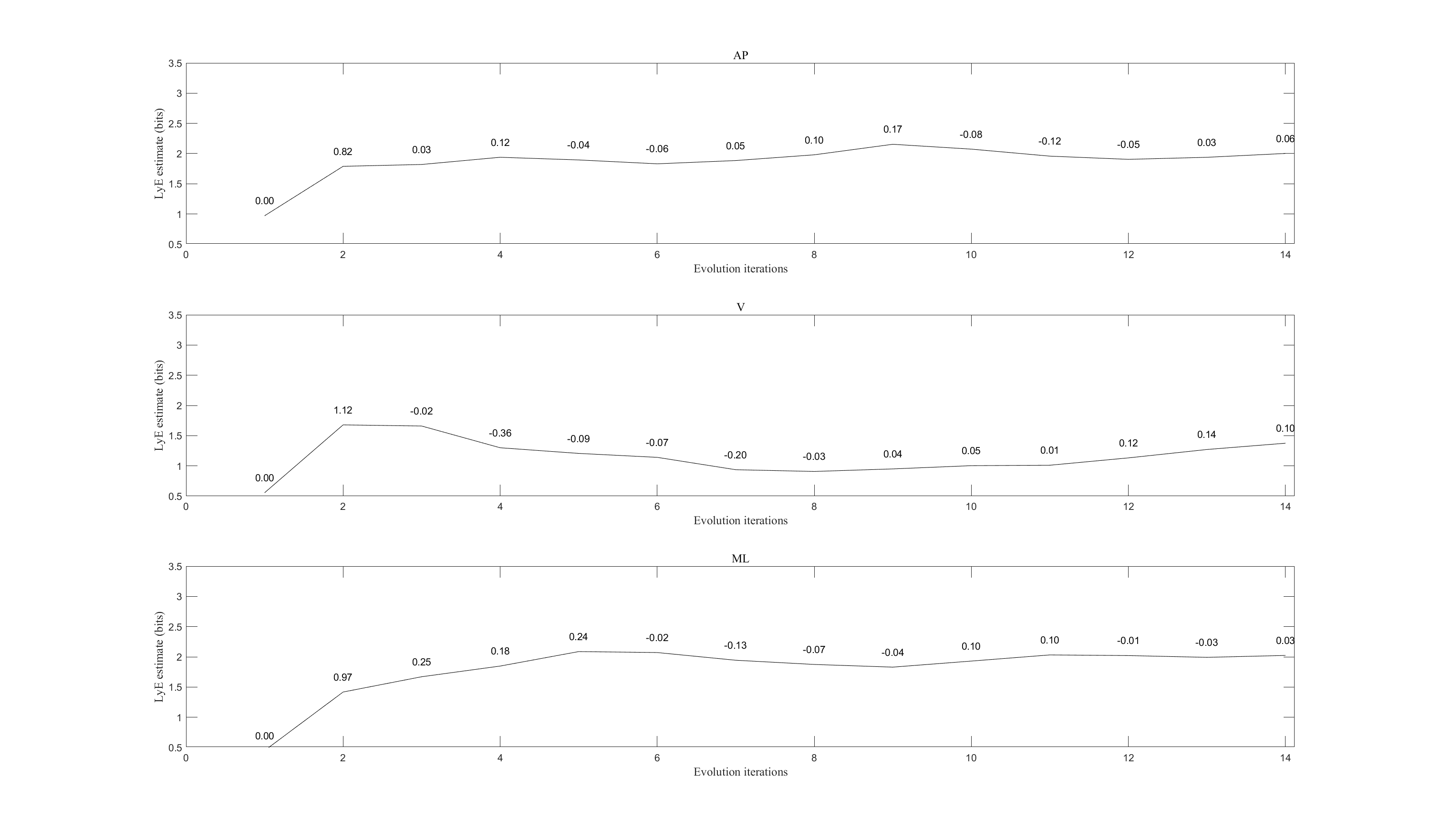

Supplement: Supplementary file 2 — Supplementary Information. [file 41598_2020_79584_MOESM2_ESM.zip › Participant20_trial9.png]

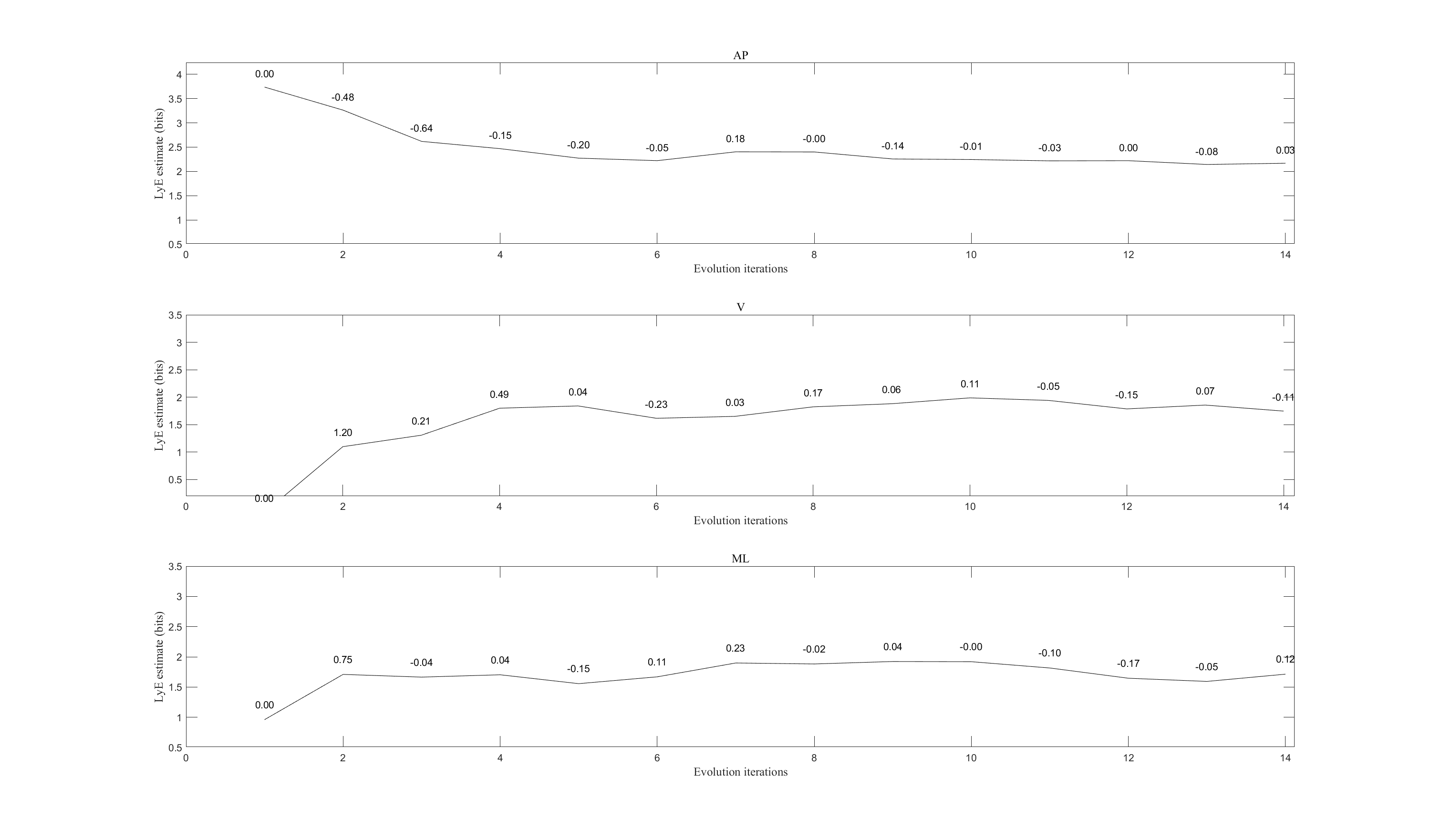

Supplement: Supplementary file 2 — Supplementary Information. [file 41598_2020_79584_MOESM2_ESM.zip › Participant3_trial1.png]

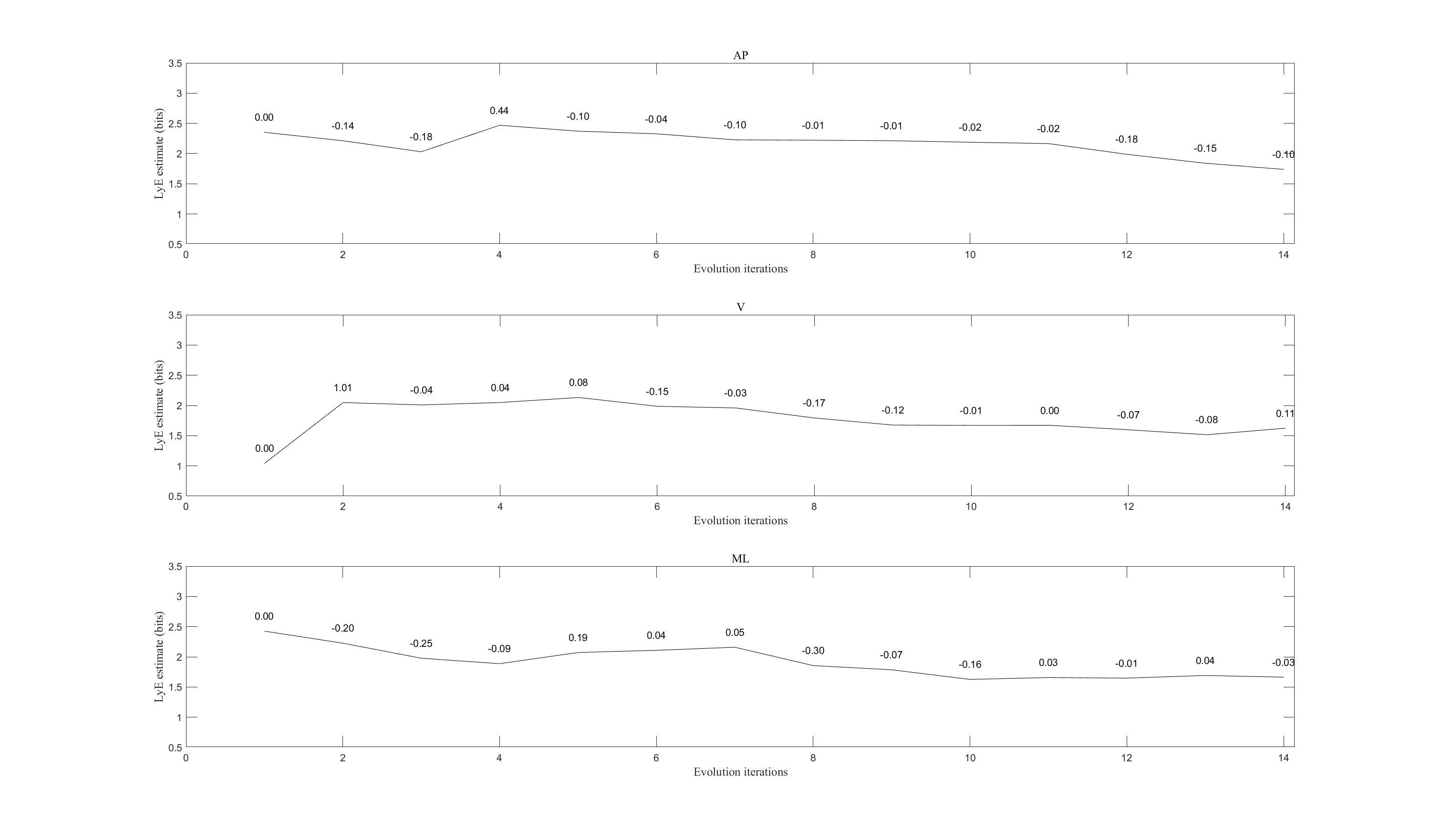

Supplement: Supplementary file 2 — Supplementary Information. [file 41598_2020_79584_MOESM2_ESM.zip › Participant3_trial10.png]

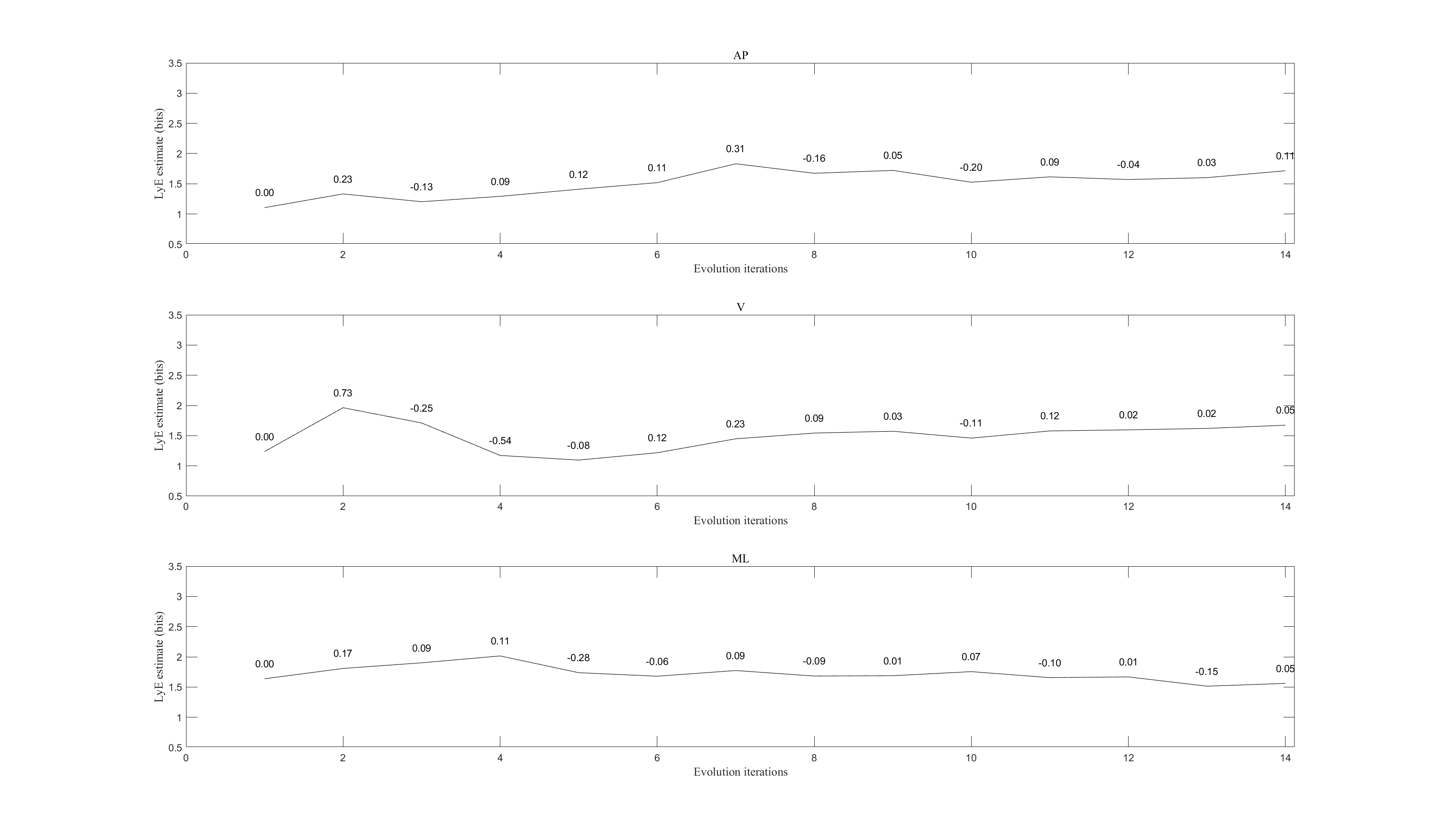

Supplement: Supplementary file 2 — Supplementary Information. [file 41598_2020_79584_MOESM2_ESM.zip › Participant3_trial11.png]

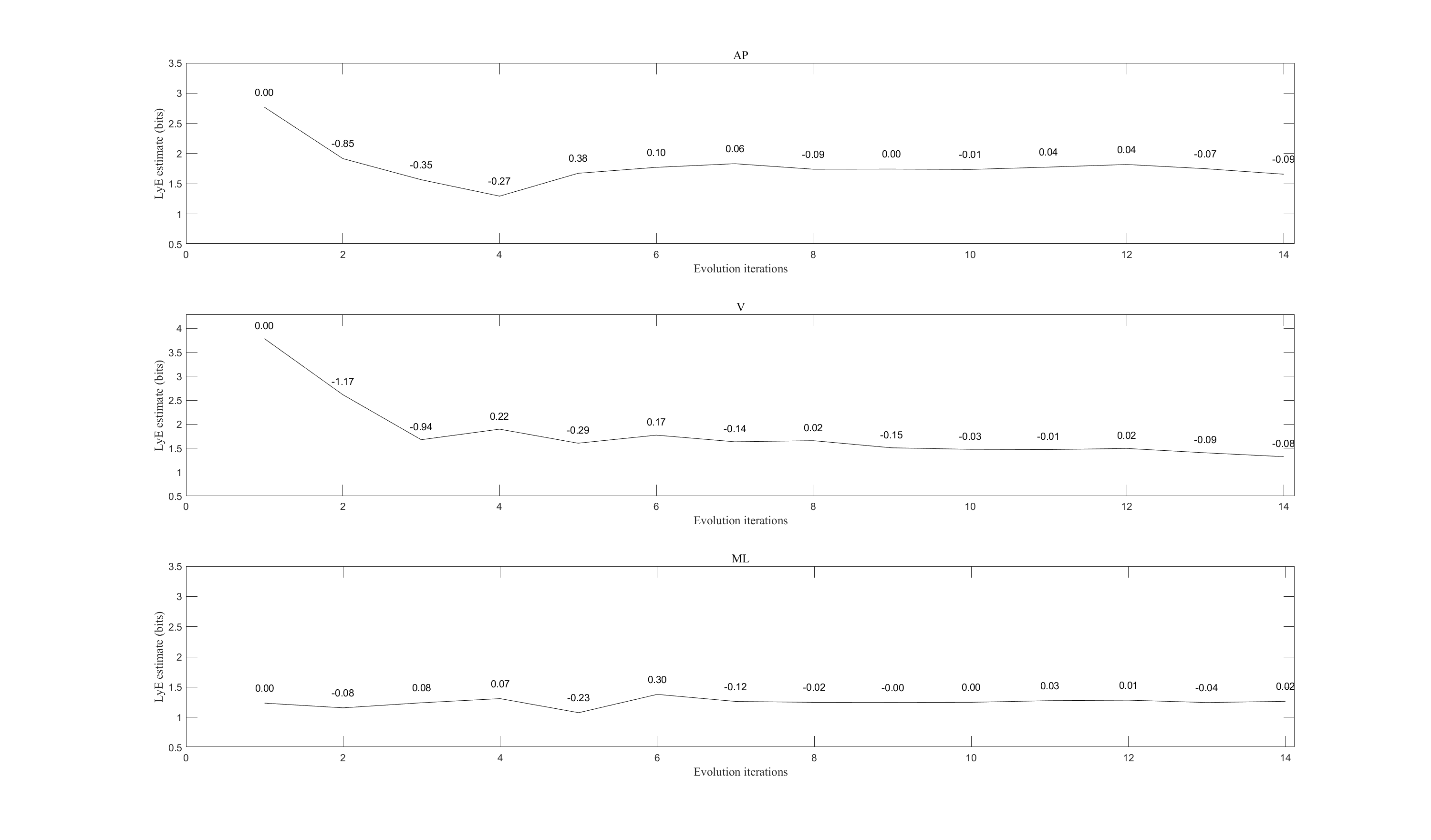

Supplement: Supplementary file 2 — Supplementary Information. [file 41598_2020_79584_MOESM2_ESM.zip › Participant3_trial12.png]

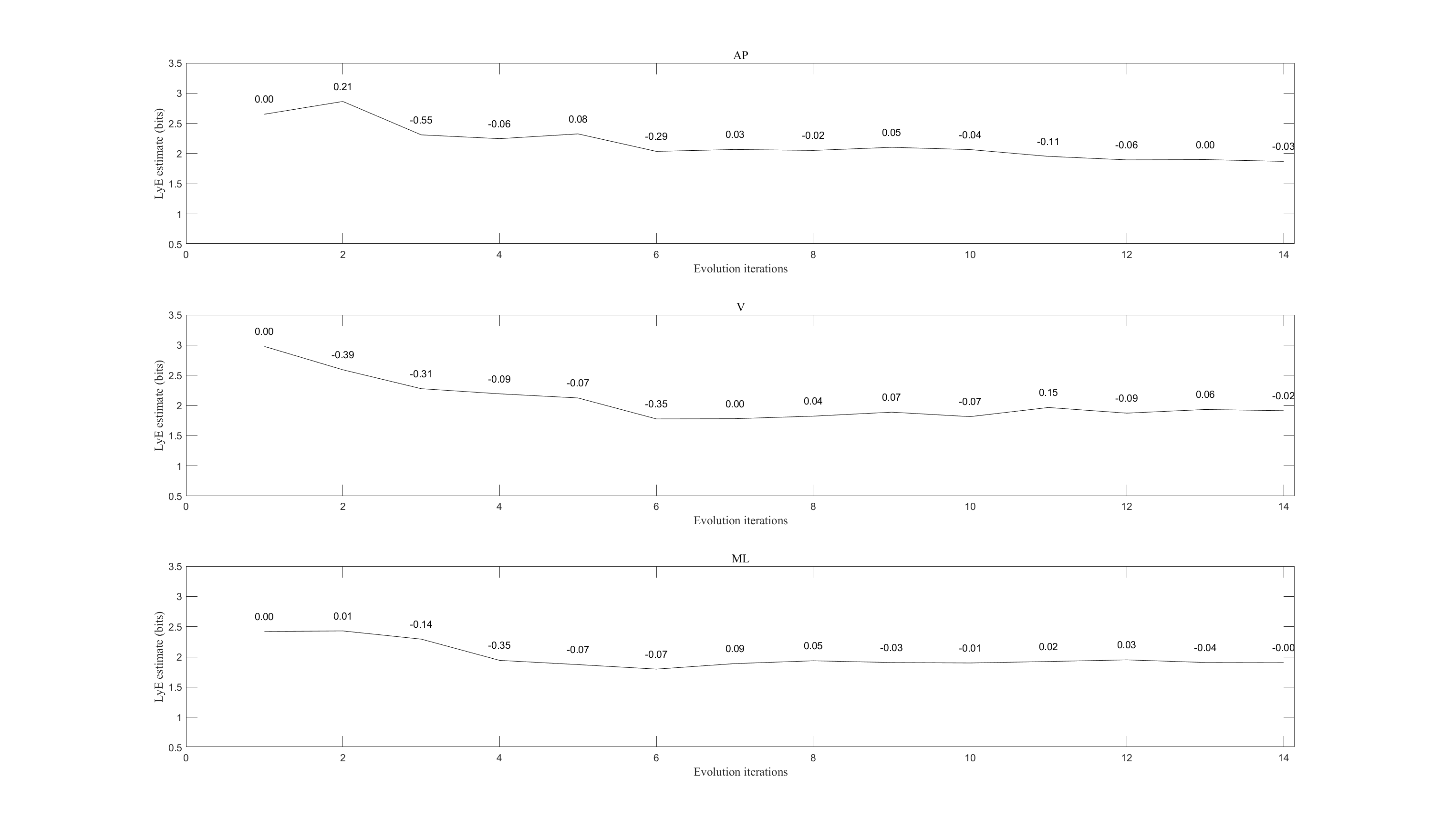

Supplement: Supplementary file 2 — Supplementary Information. [file 41598_2020_79584_MOESM2_ESM.zip › Participant3_trial2.png]

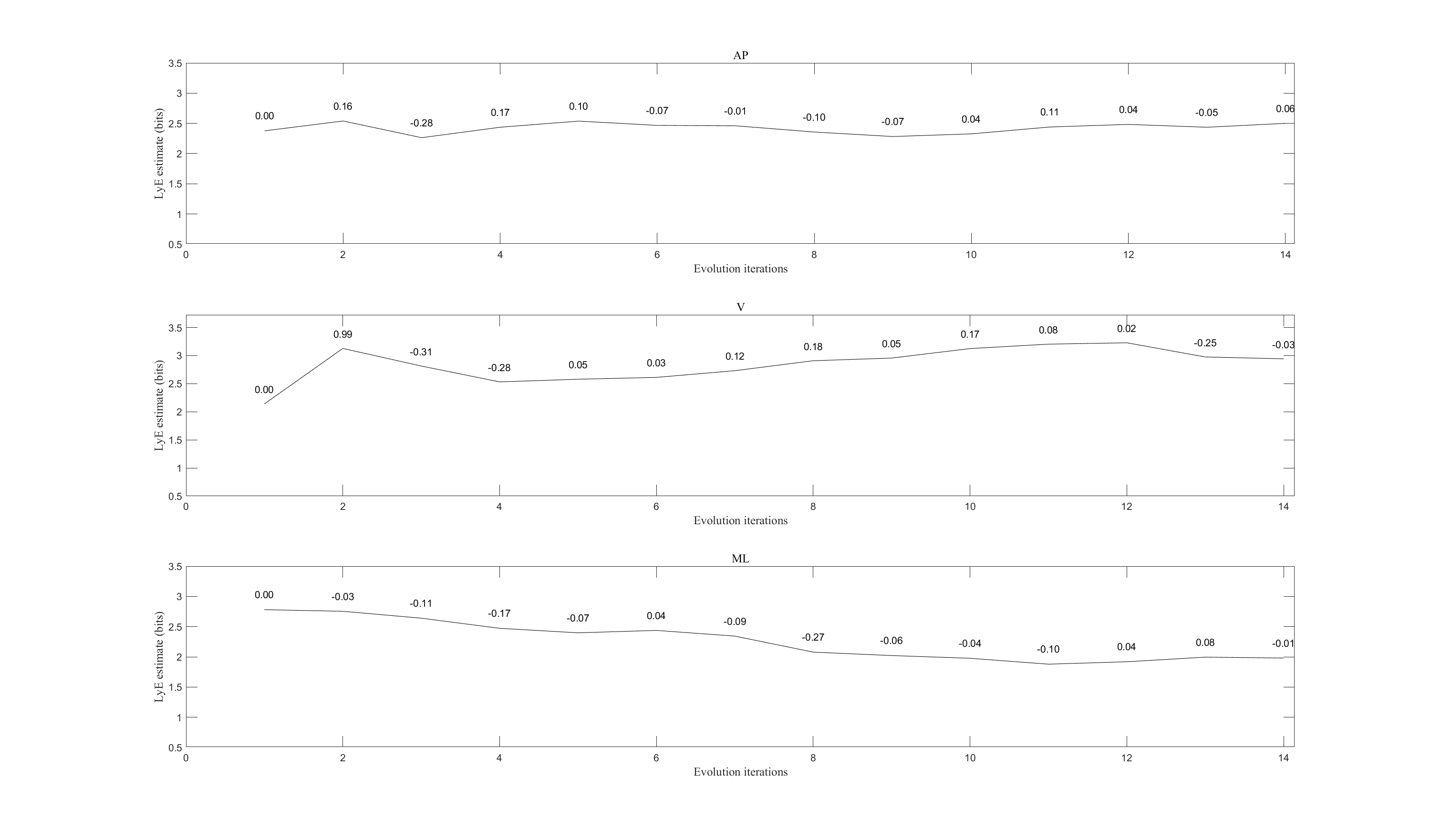

Supplement: Supplementary file 2 — Supplementary Information. [file 41598_2020_79584_MOESM2_ESM.zip › Participant3_trial3.png]

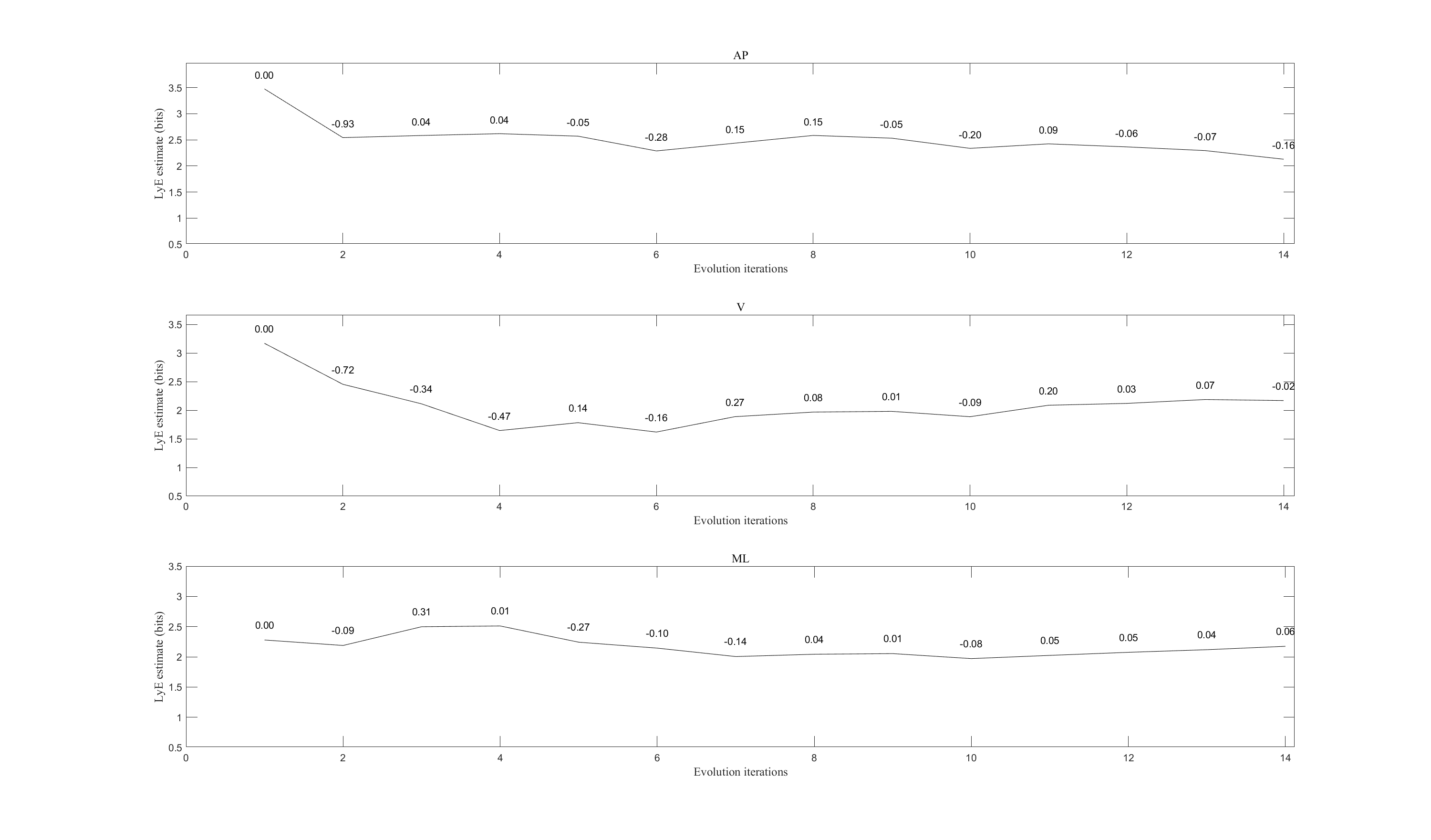

Supplement: Supplementary file 2 — Supplementary Information. [file 41598_2020_79584_MOESM2_ESM.zip › Participant3_trial4.png]

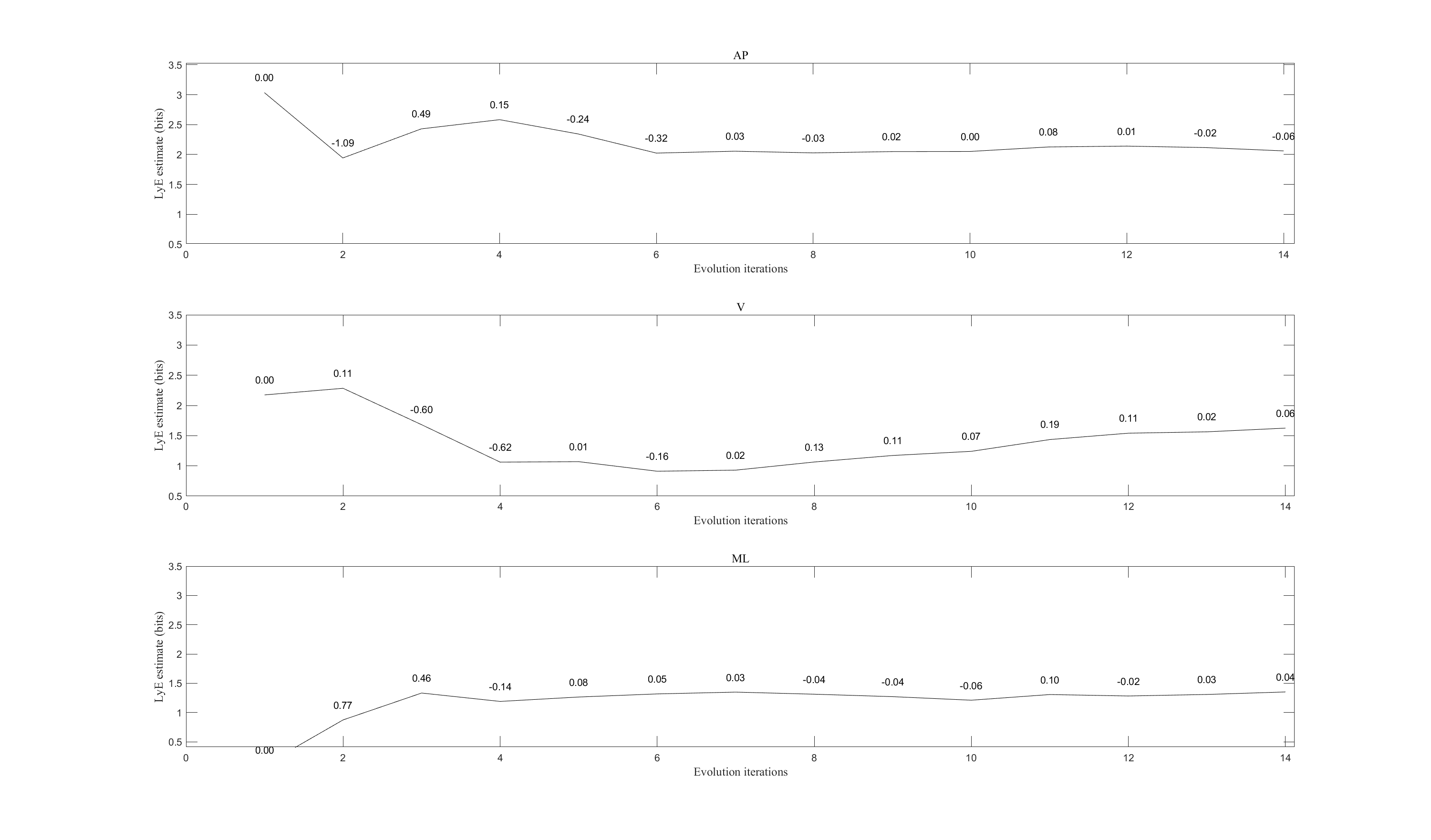

Supplement: Supplementary file 2 — Supplementary Information. [file 41598_2020_79584_MOESM2_ESM.zip › Participant3_trial5.png]

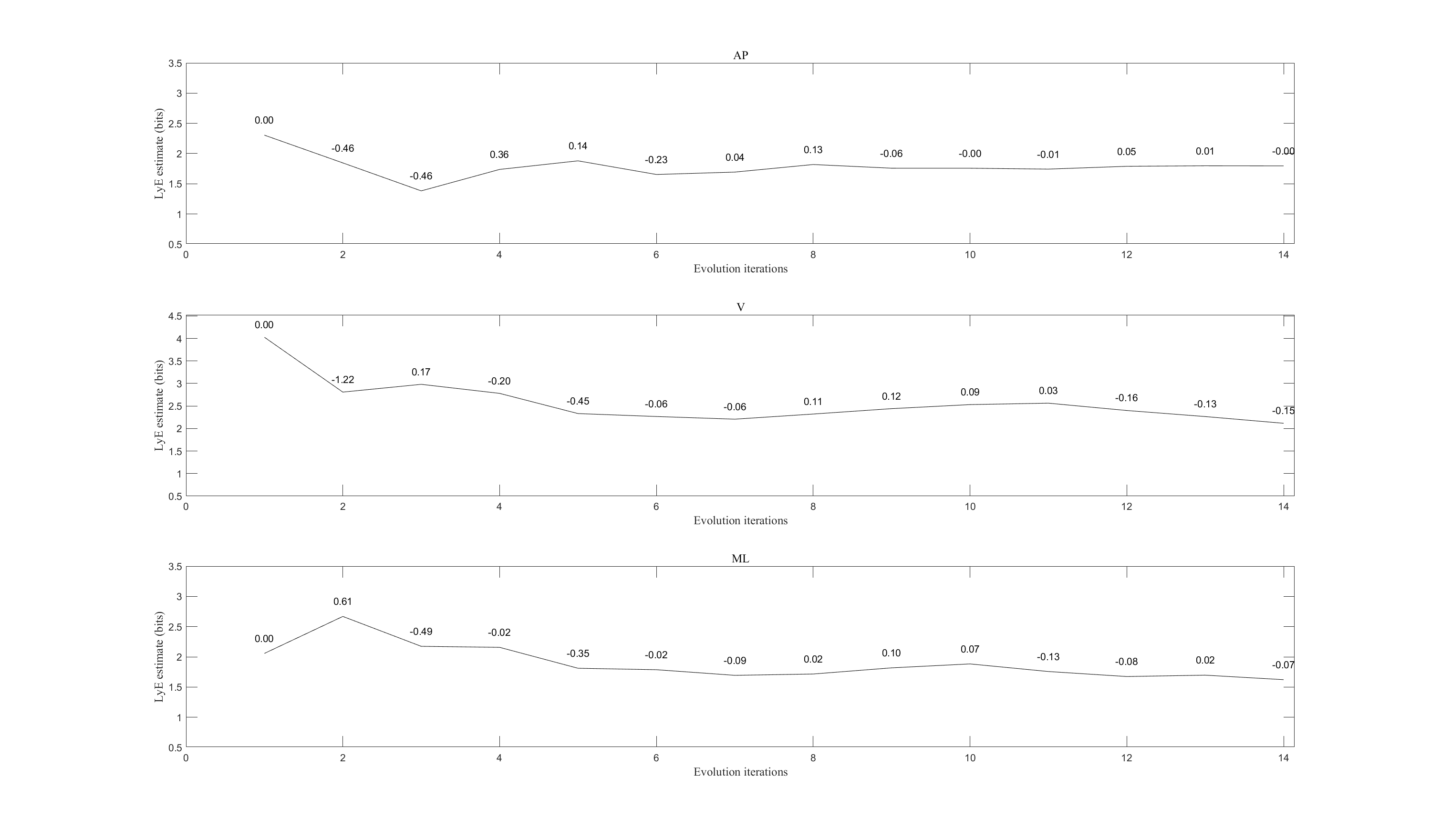

Supplement: Supplementary file 2 — Supplementary Information. [file 41598_2020_79584_MOESM2_ESM.zip › Participant3_trial6.png]

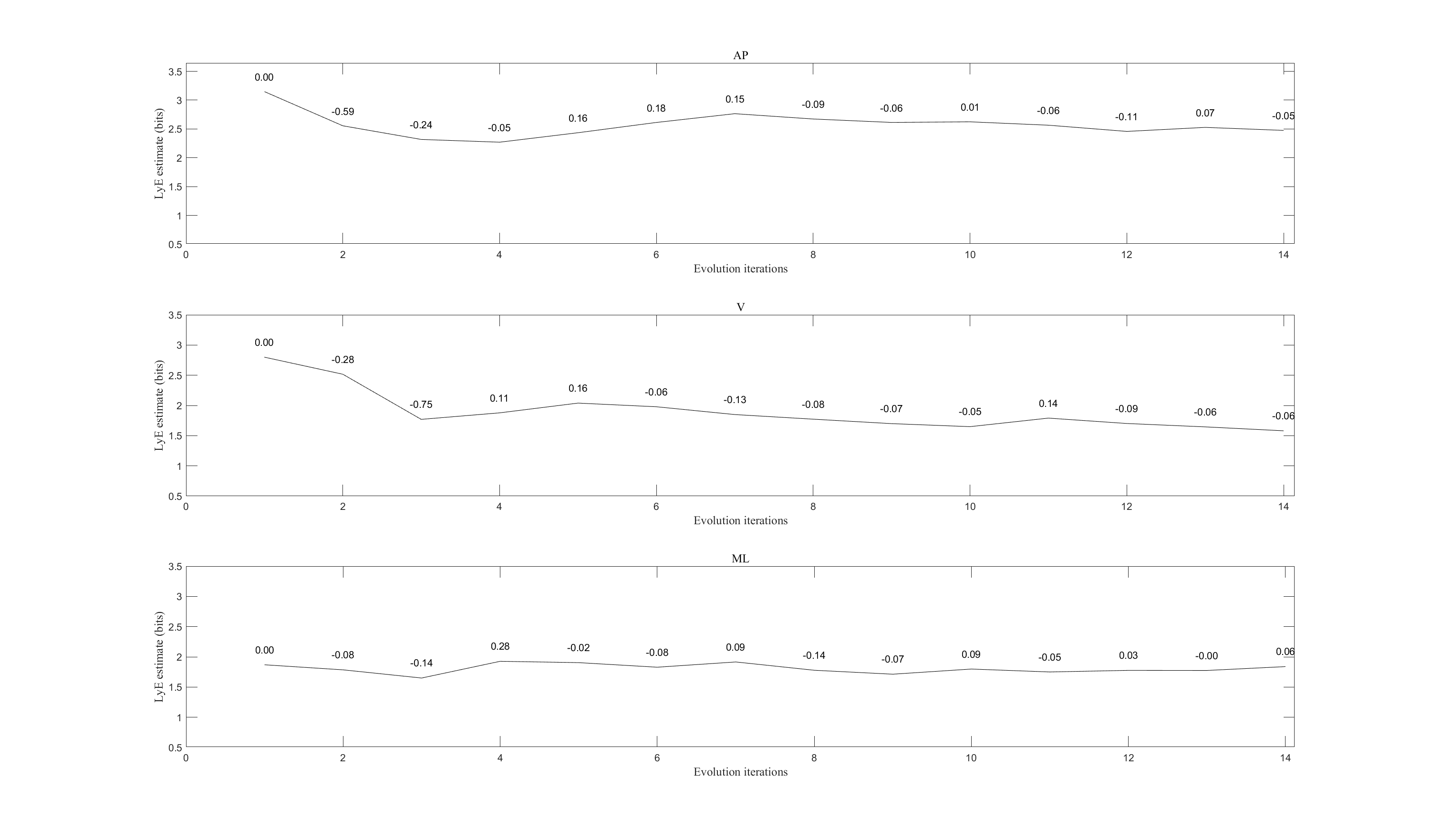

Supplement: Supplementary file 2 — Supplementary Information. [file 41598_2020_79584_MOESM2_ESM.zip › Participant3_trial7.png]

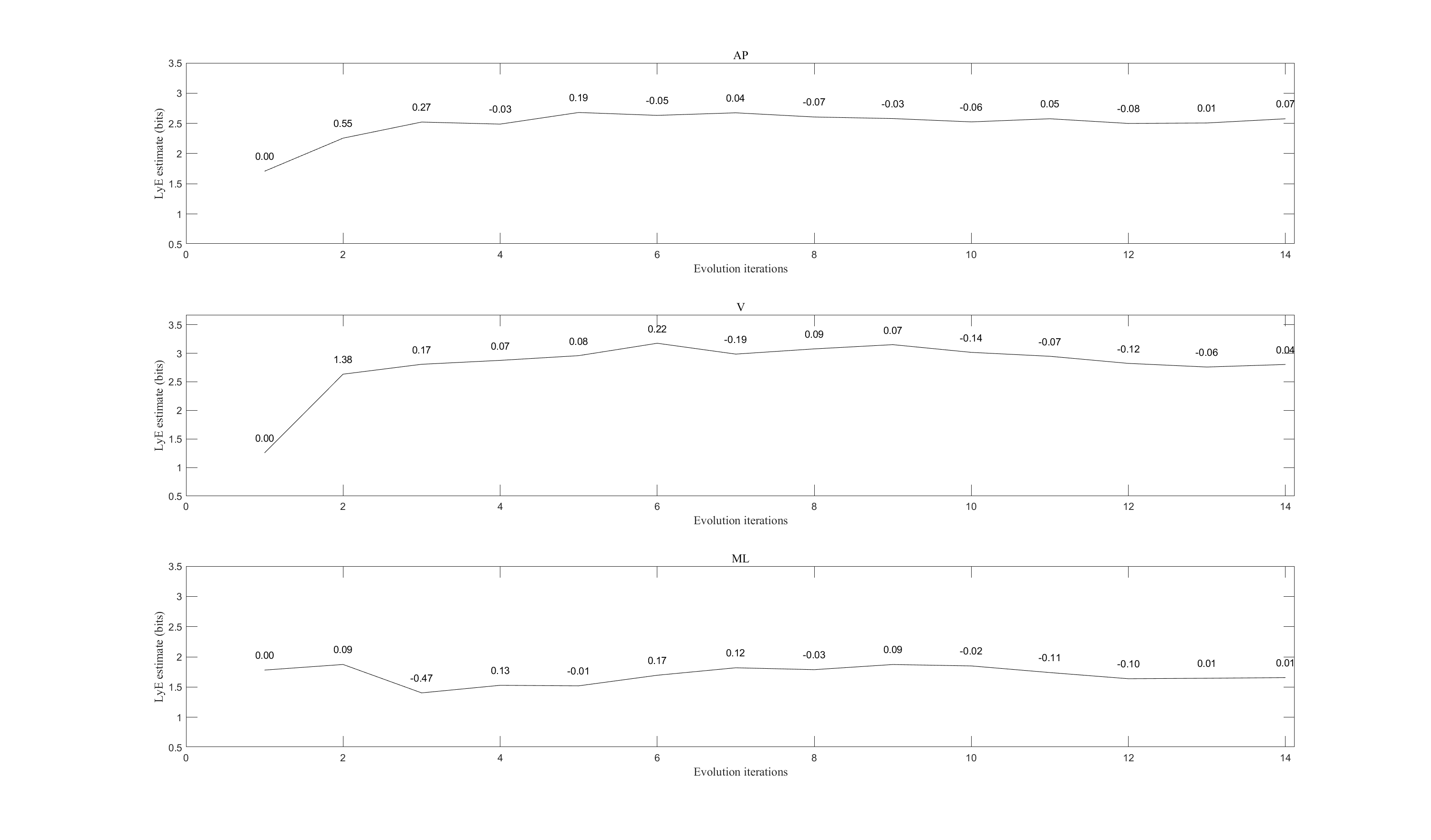

Supplement: Supplementary file 2 — Supplementary Information. [file 41598_2020_79584_MOESM2_ESM.zip › Participant3_trial8.png]

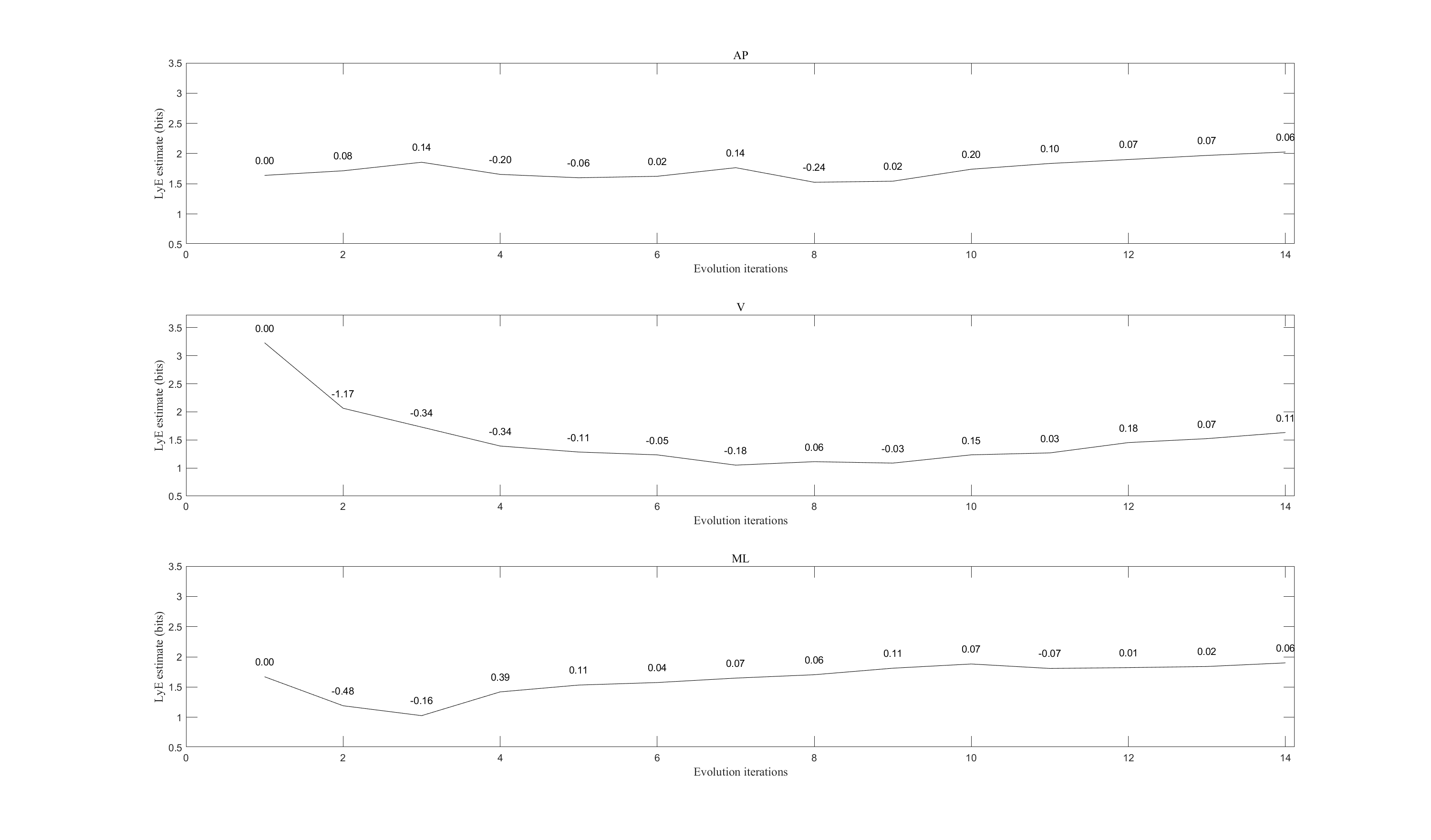

Supplement: Supplementary file 2 — Supplementary Information. [file 41598_2020_79584_MOESM2_ESM.zip › Participant3_trial9.png]

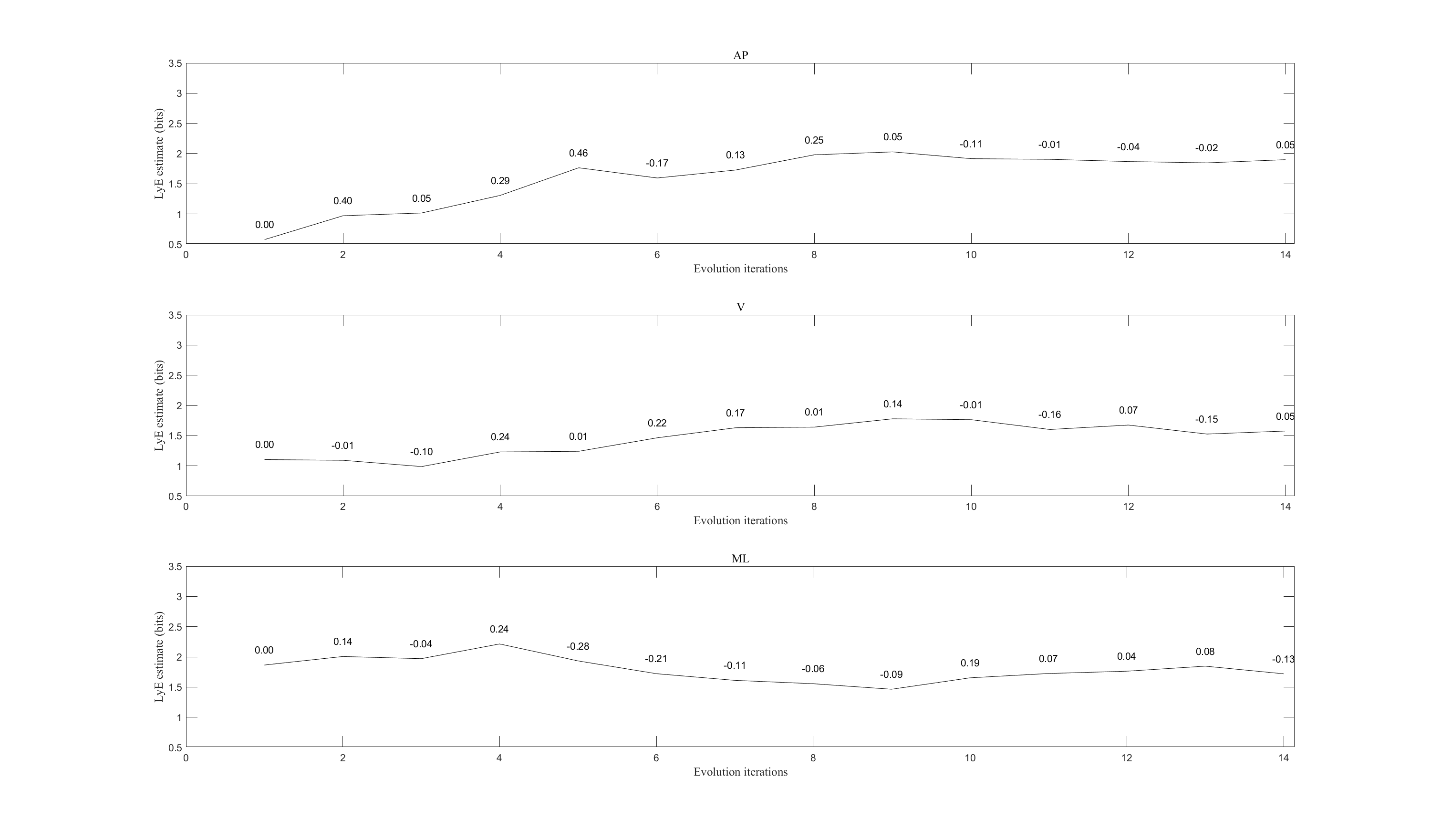

Supplement: Supplementary file 2 — Supplementary Information. [file 41598_2020_79584_MOESM2_ESM.zip › Participant4_trial1.png]

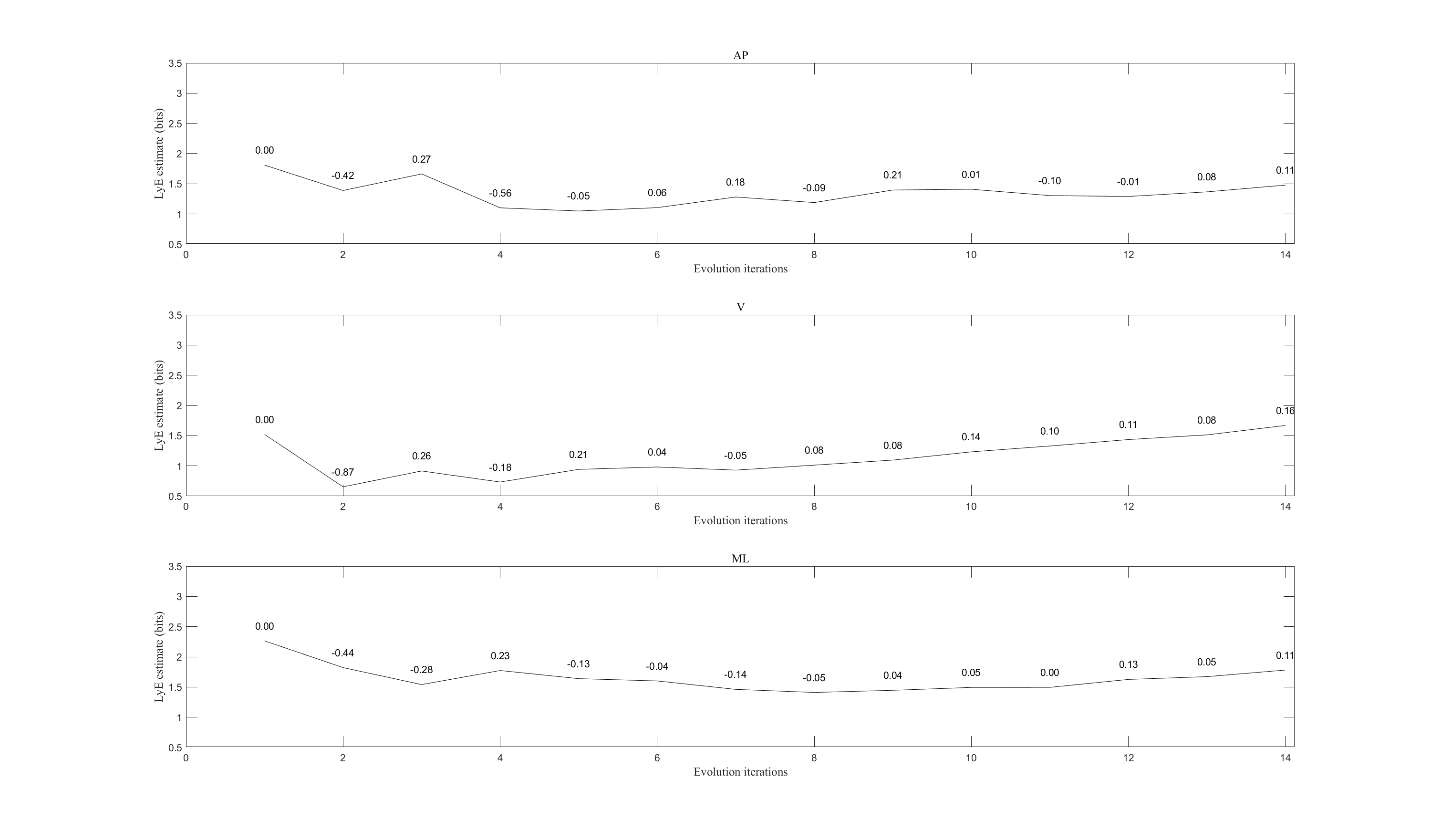

Supplement: Supplementary file 2 — Supplementary Information. [file 41598_2020_79584_MOESM2_ESM.zip › Participant4_trial10.png]

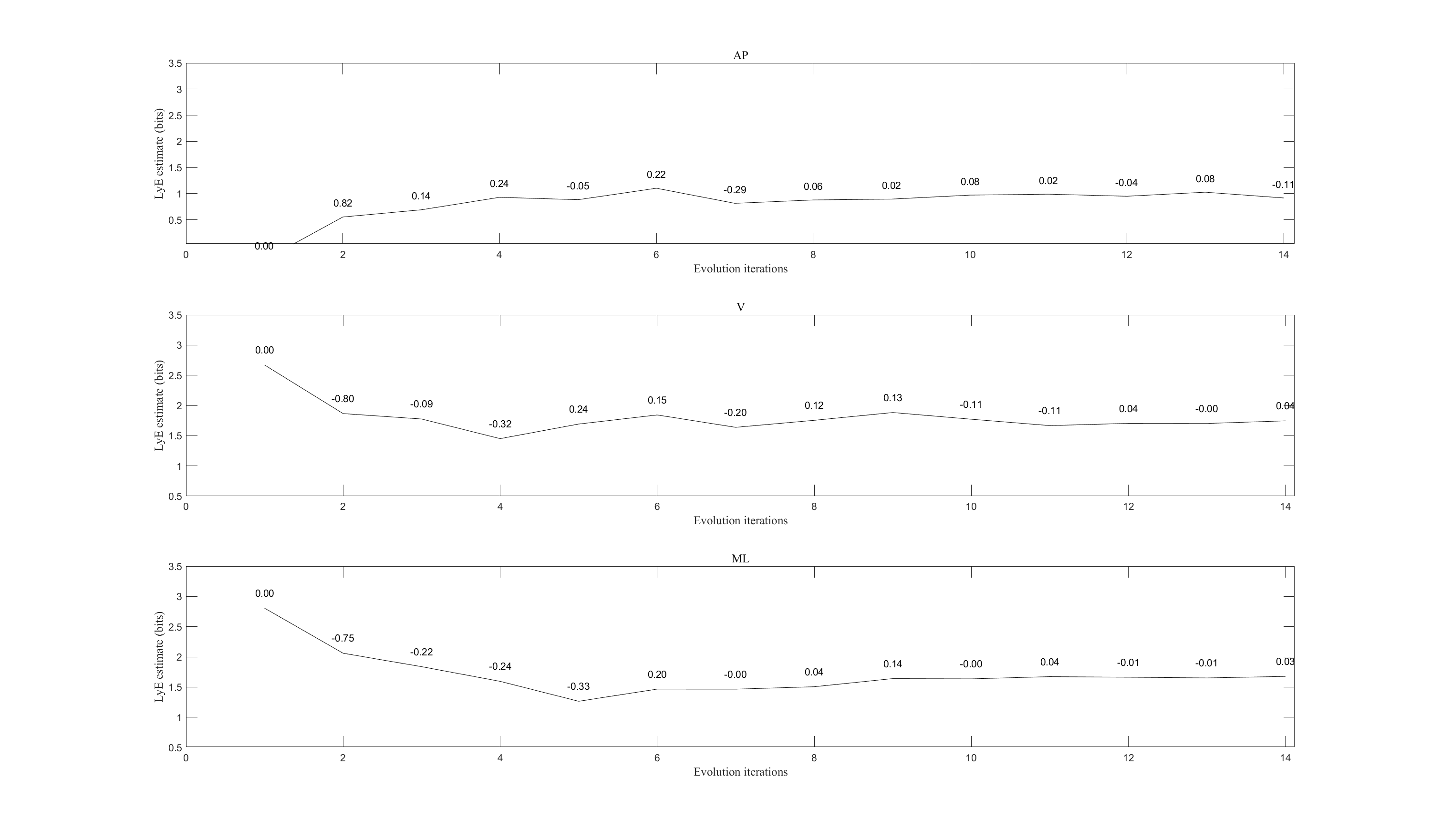

Supplement: Supplementary file 2 — Supplementary Information. [file 41598_2020_79584_MOESM2_ESM.zip › Participant4_trial11.png]

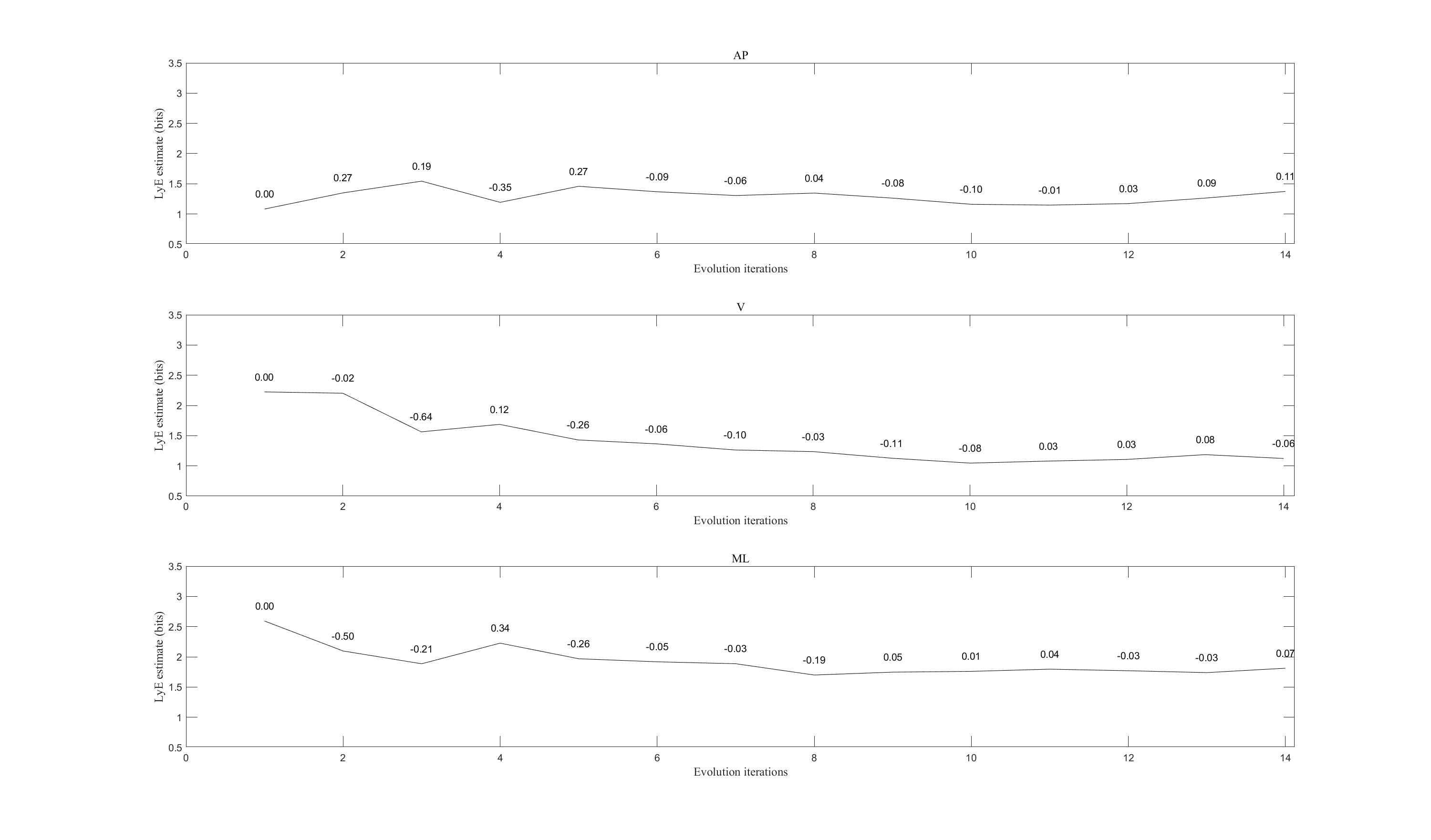

Supplement: Supplementary file 2 — Supplementary Information. [file 41598_2020_79584_MOESM2_ESM.zip › Participant4_trial12.png]

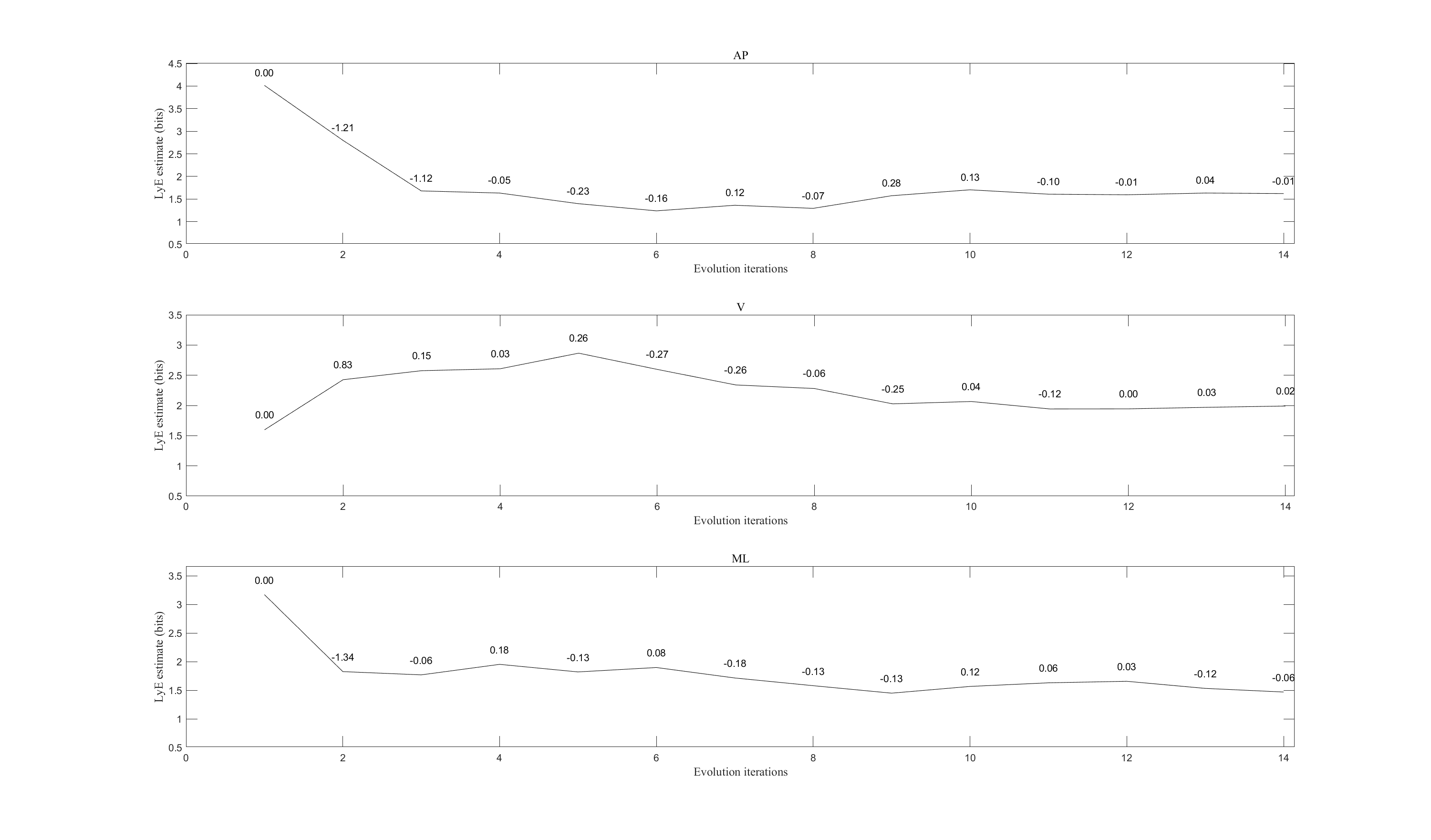

Supplement: Supplementary file 2 — Supplementary Information. [file 41598_2020_79584_MOESM2_ESM.zip › Participant4_trial2.png]

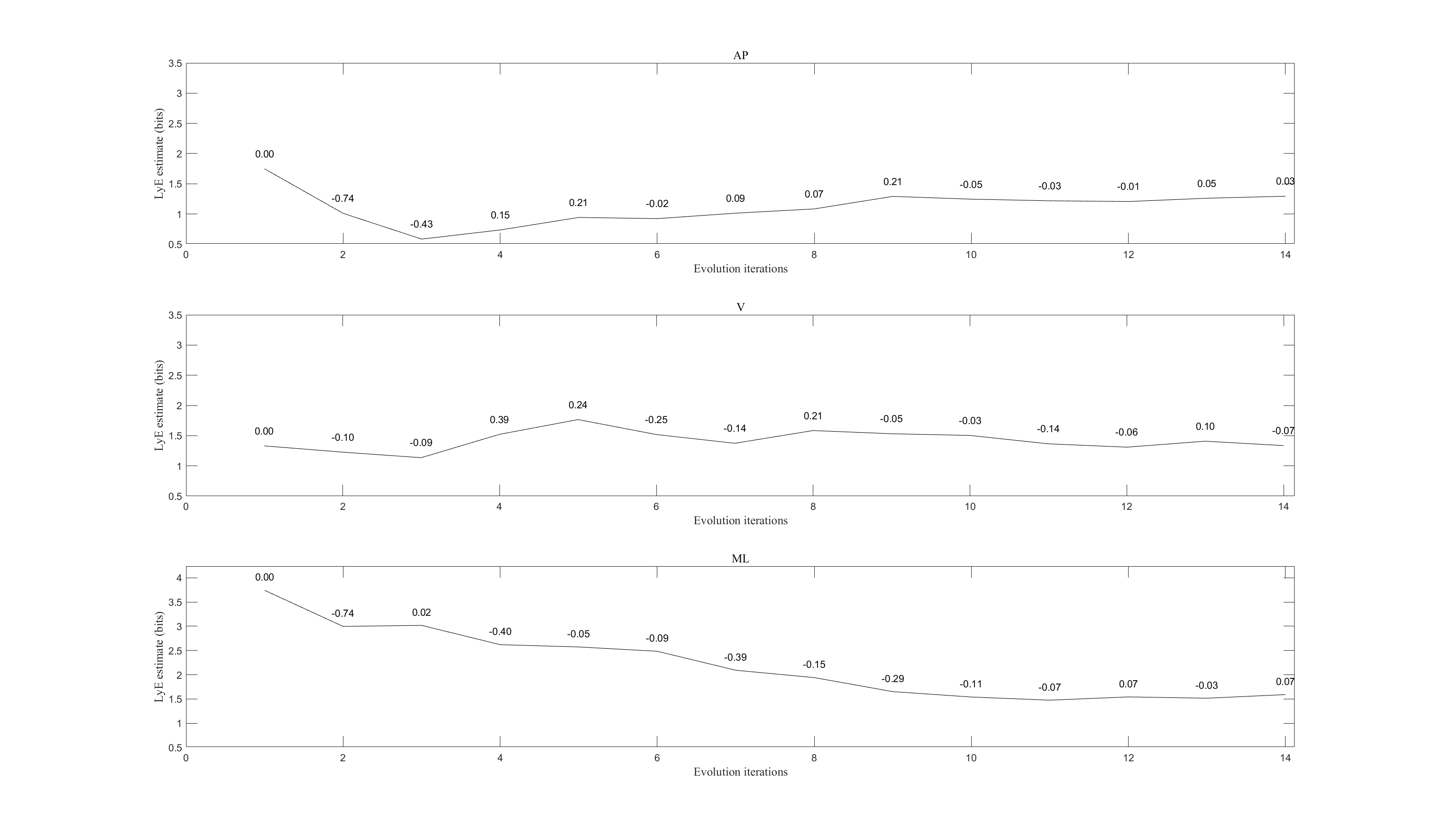

Supplement: Supplementary file 2 — Supplementary Information. [file 41598_2020_79584_MOESM2_ESM.zip › Participant4_trial3.png]

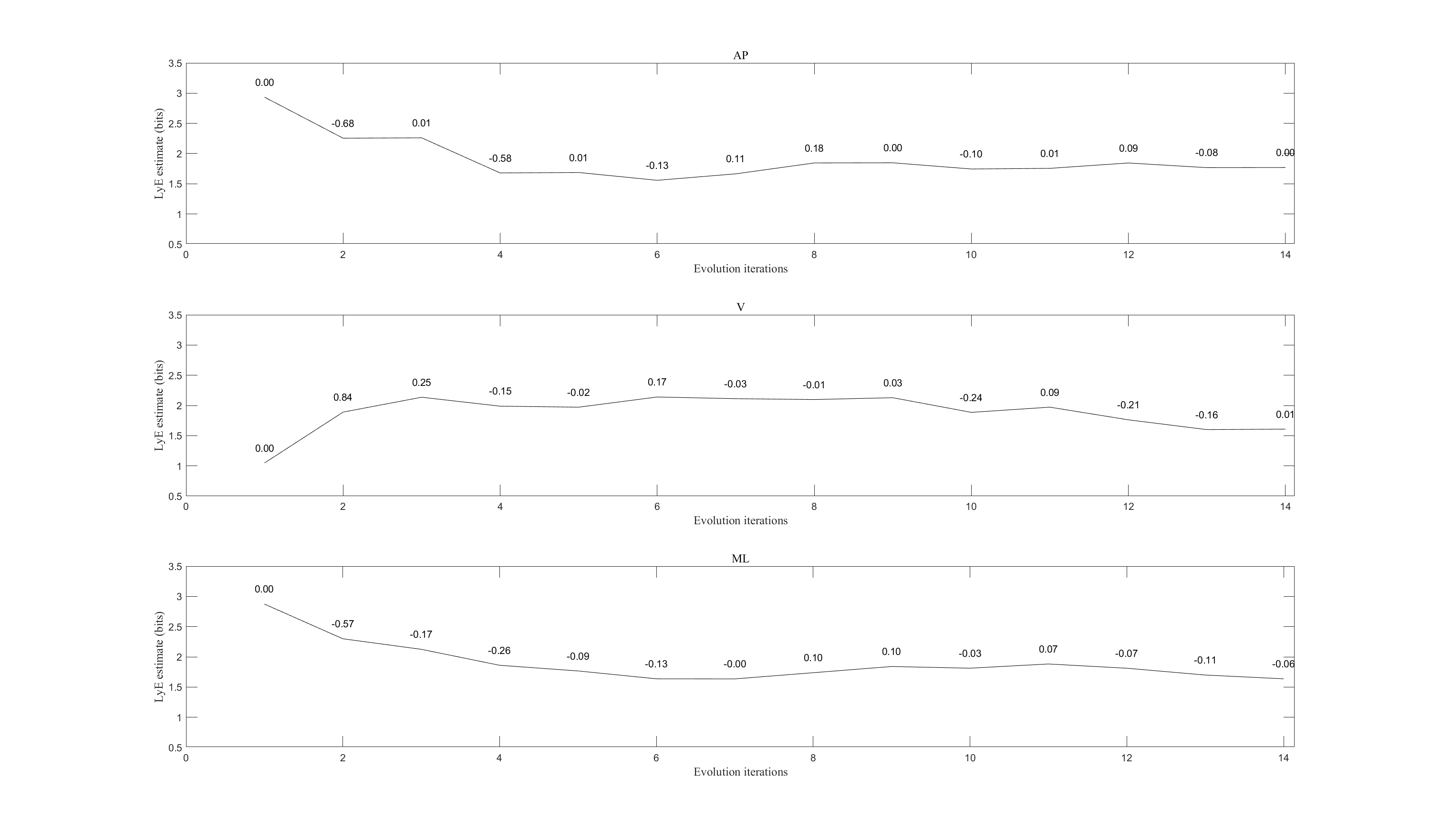

Supplement: Supplementary file 2 — Supplementary Information. [file 41598_2020_79584_MOESM2_ESM.zip › Participant4_trial4.png]

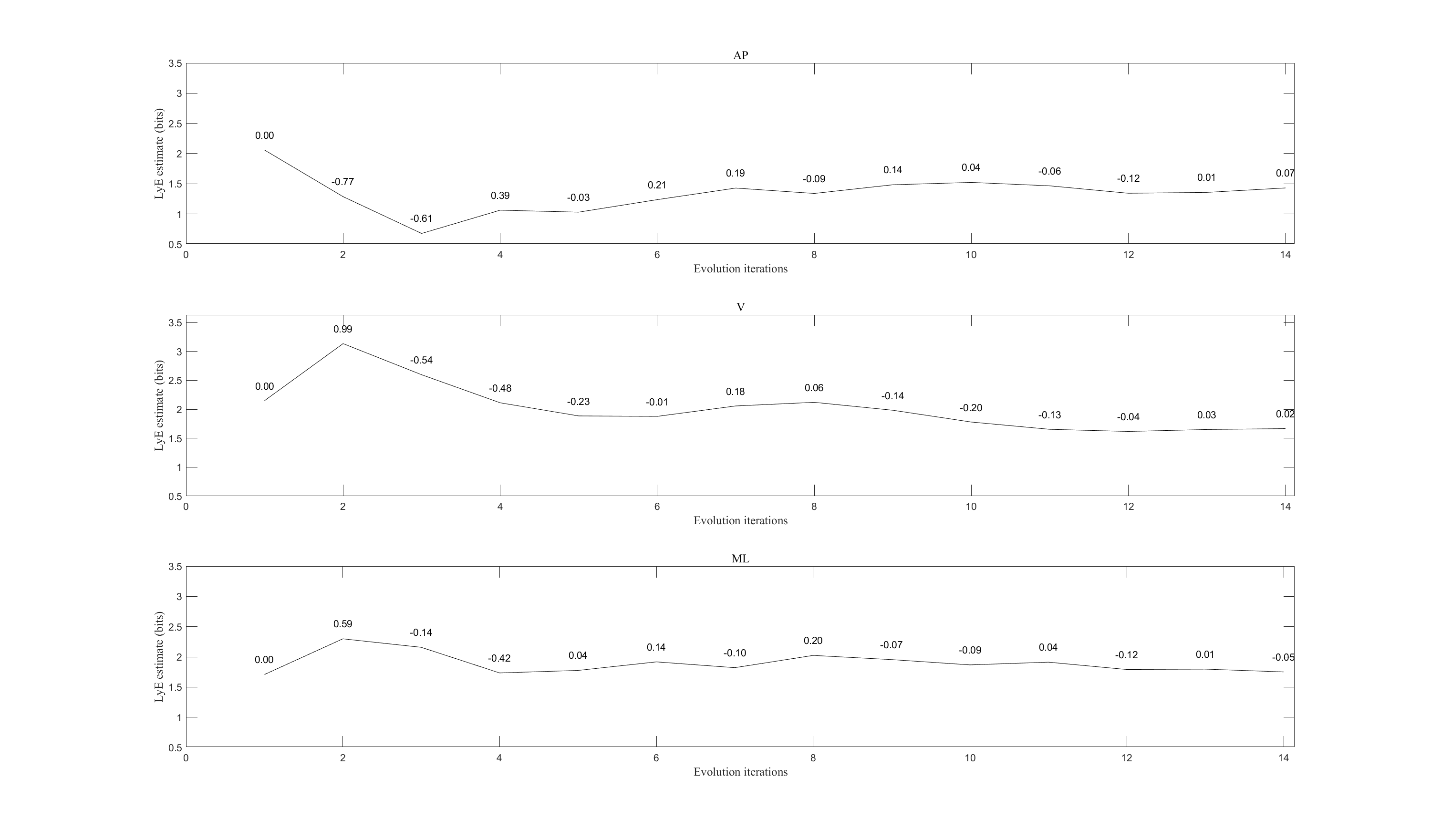

Supplement: Supplementary file 2 — Supplementary Information. [file 41598_2020_79584_MOESM2_ESM.zip › Participant4_trial5.png]

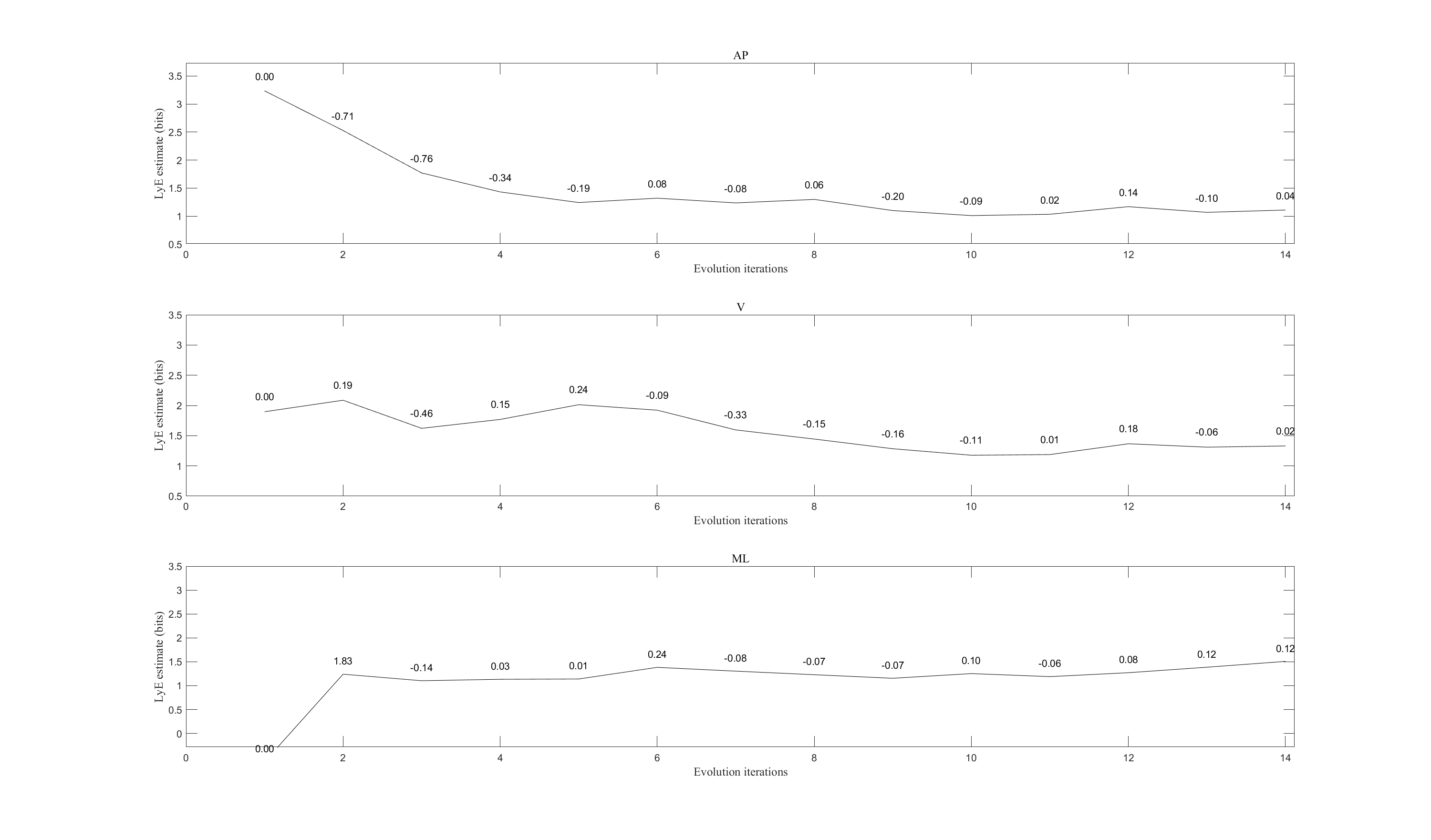

Supplement: Supplementary file 2 — Supplementary Information. [file 41598_2020_79584_MOESM2_ESM.zip › Participant4_trial6.png]

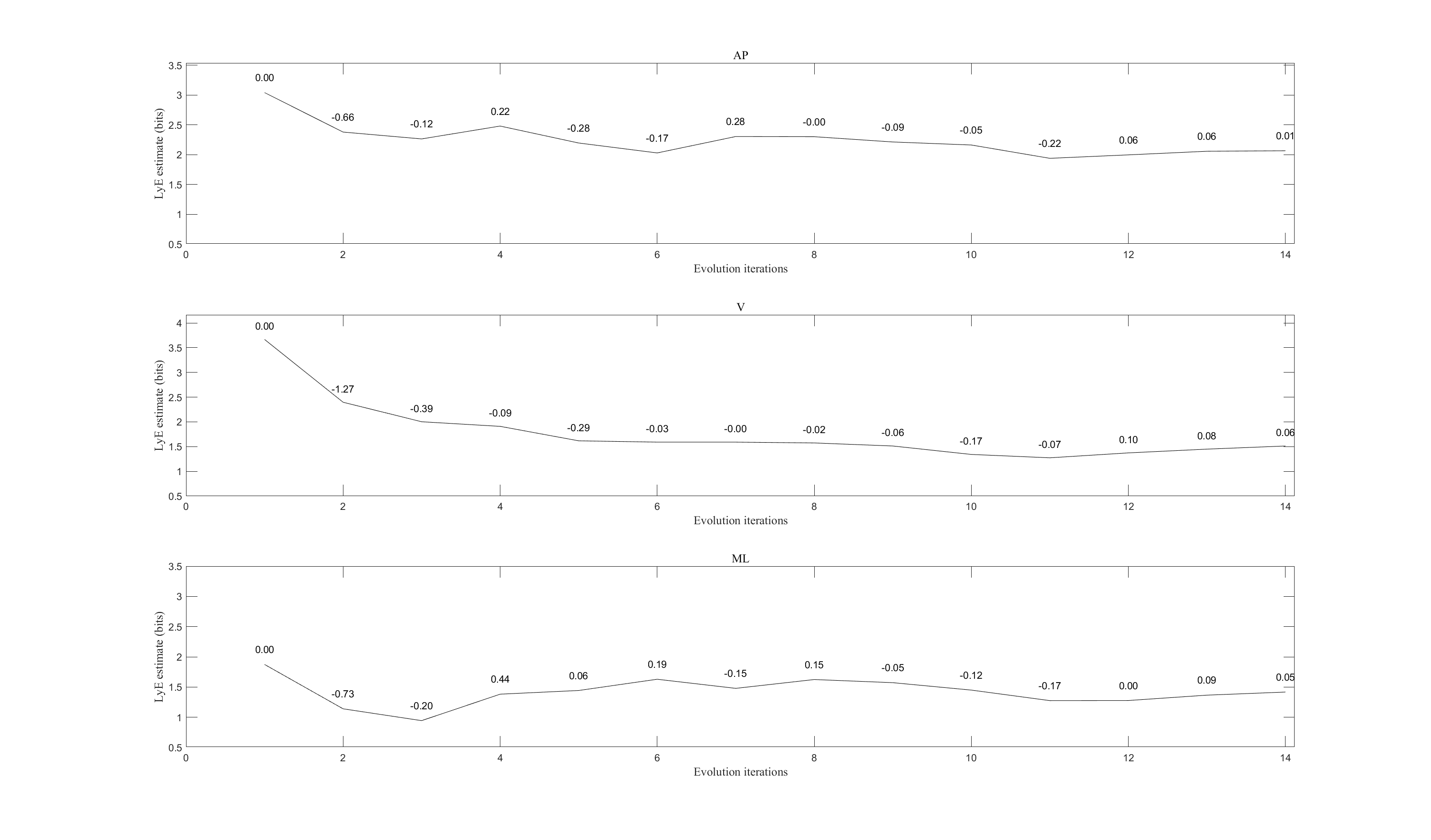

Supplement: Supplementary file 2 — Supplementary Information. [file 41598_2020_79584_MOESM2_ESM.zip › Participant4_trial7.png]

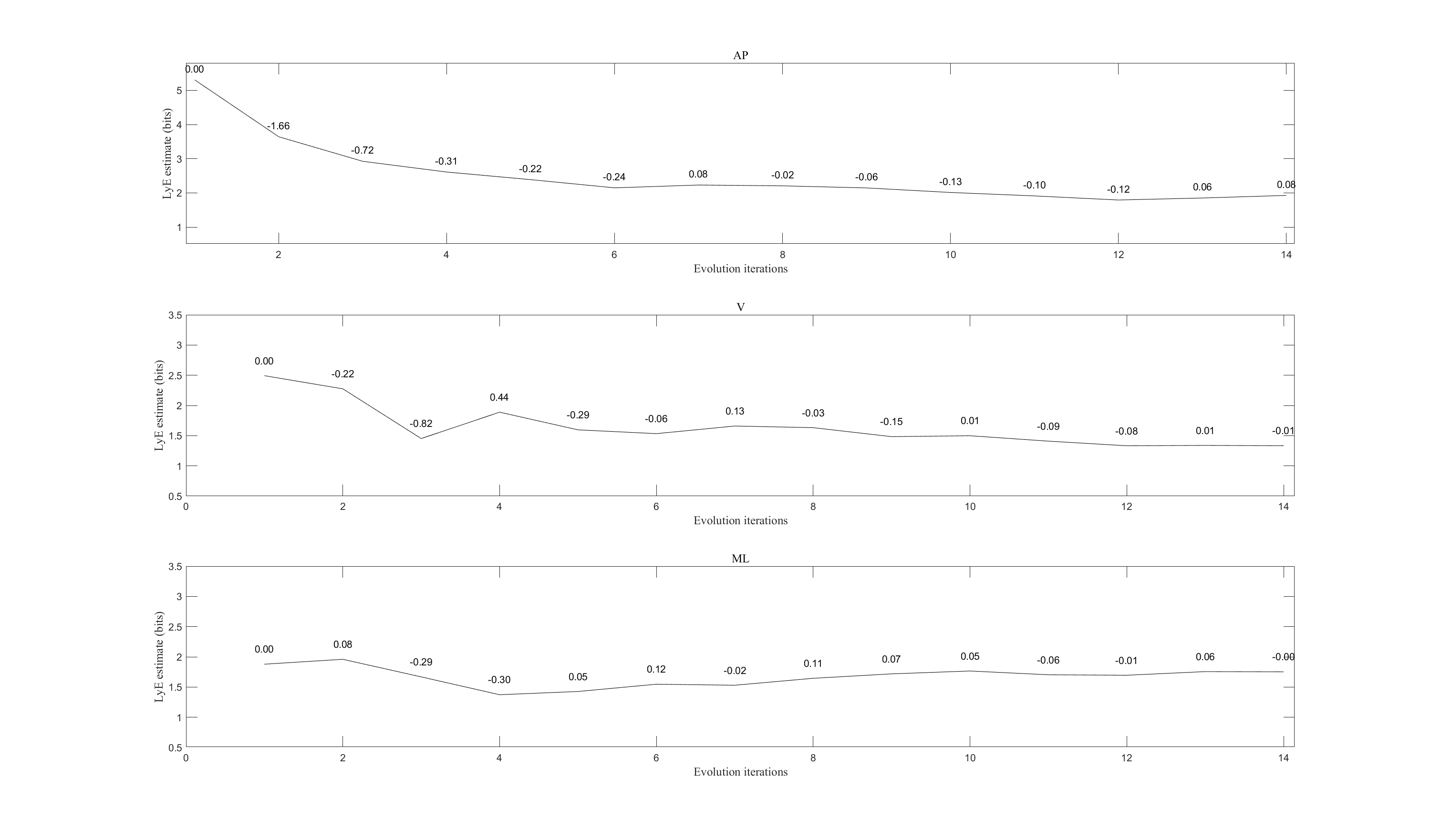

Supplement: Supplementary file 2 — Supplementary Information. [file 41598_2020_79584_MOESM2_ESM.zip › Participant4_trial8.png]

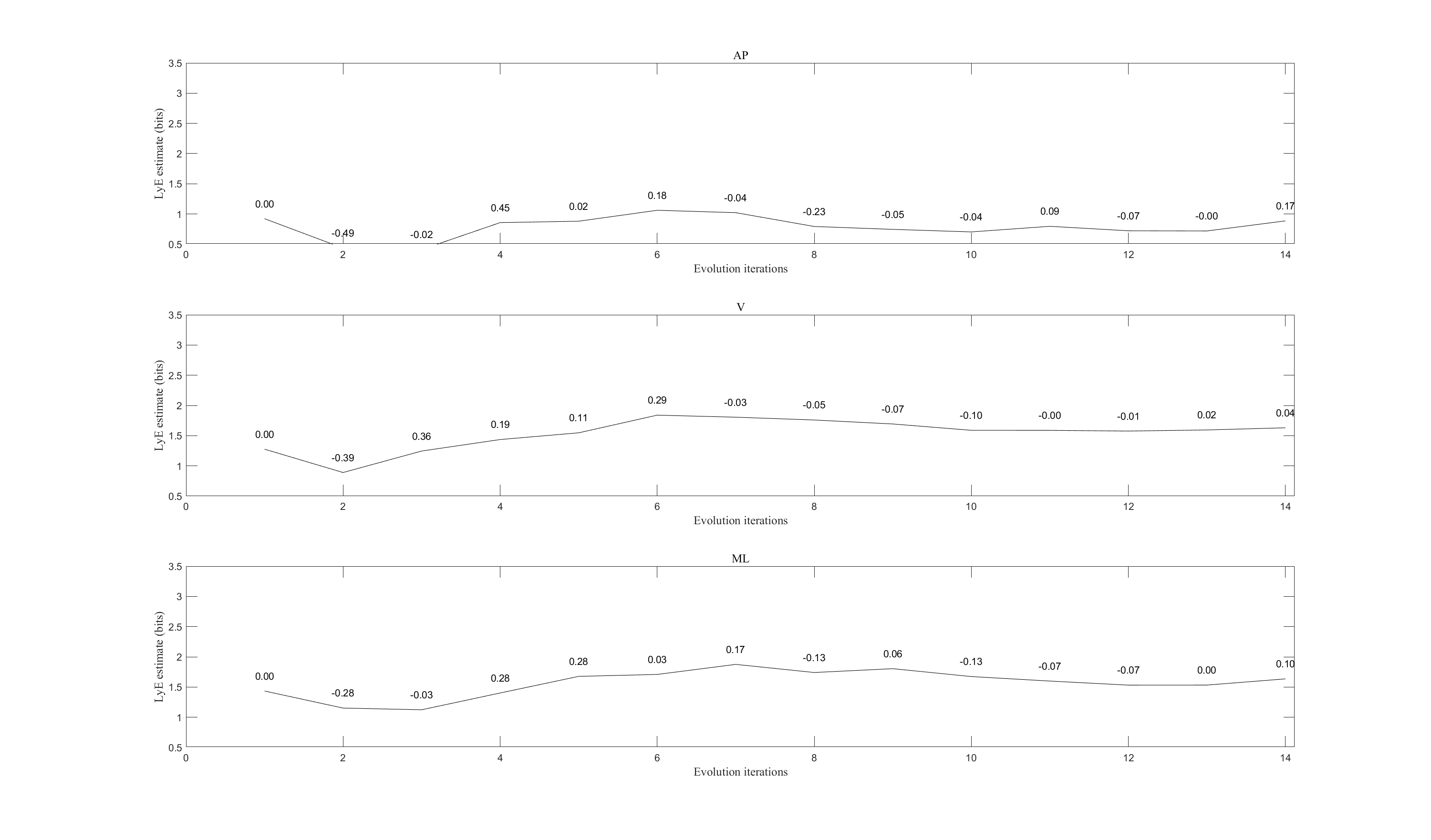

Supplement: Supplementary file 2 — Supplementary Information. [file 41598_2020_79584_MOESM2_ESM.zip › Participant4_trial9.png]

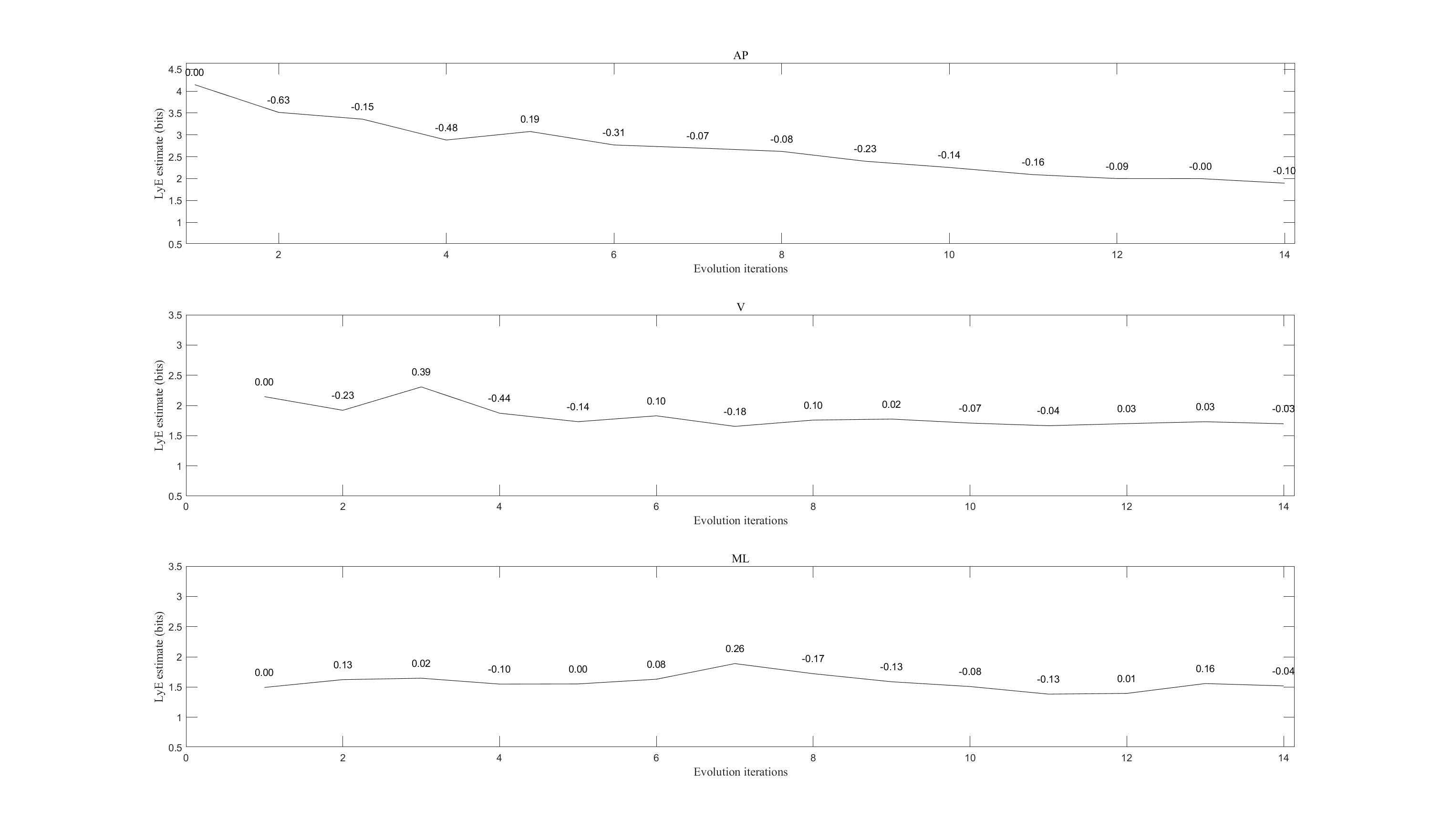

Supplement: Supplementary file 2 — Supplementary Information. [file 41598_2020_79584_MOESM2_ESM.zip › Participant5_trial1.png]

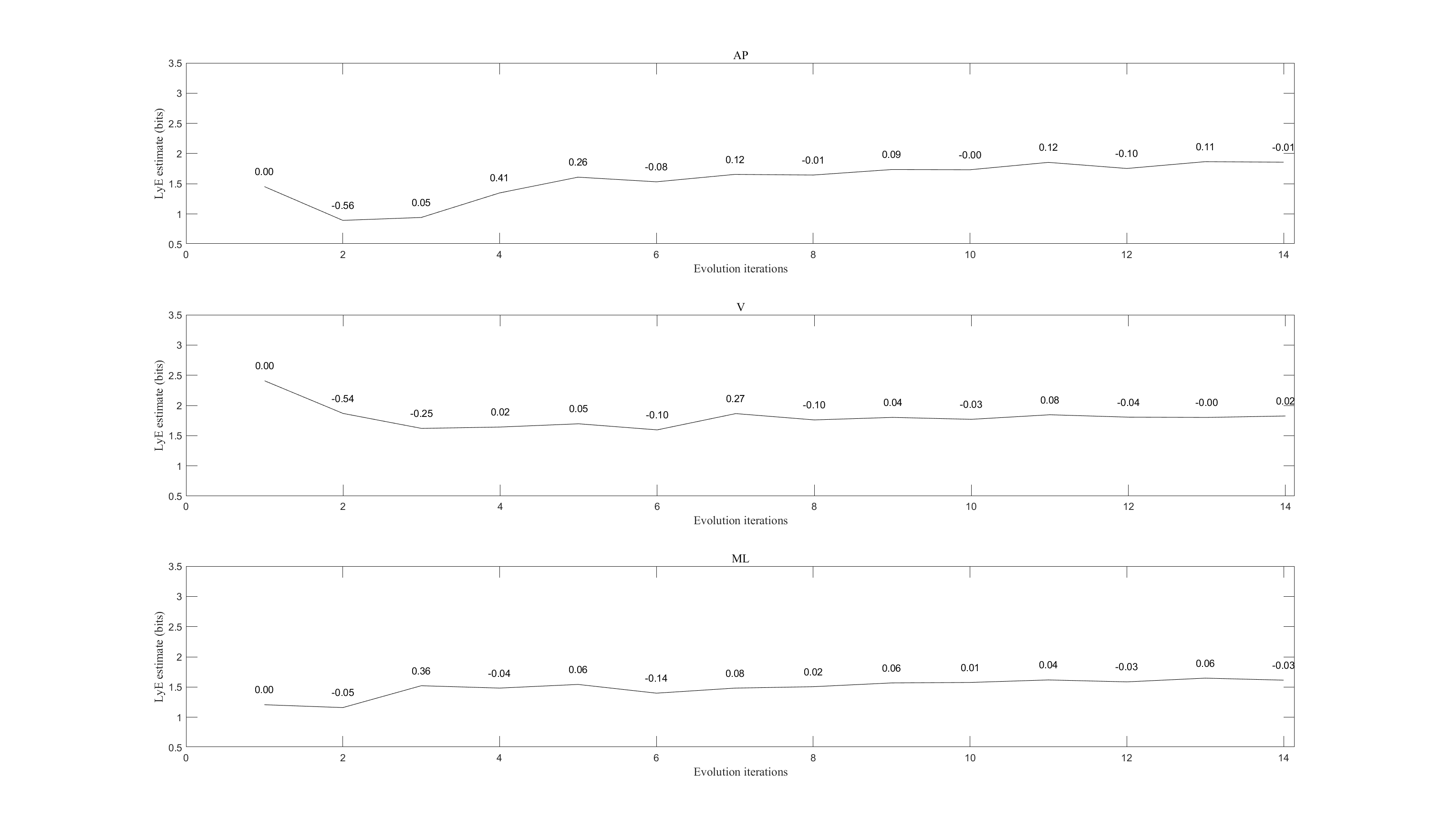

Supplement: Supplementary file 2 — Supplementary Information. [file 41598_2020_79584_MOESM2_ESM.zip › Participant5_trial10.png]

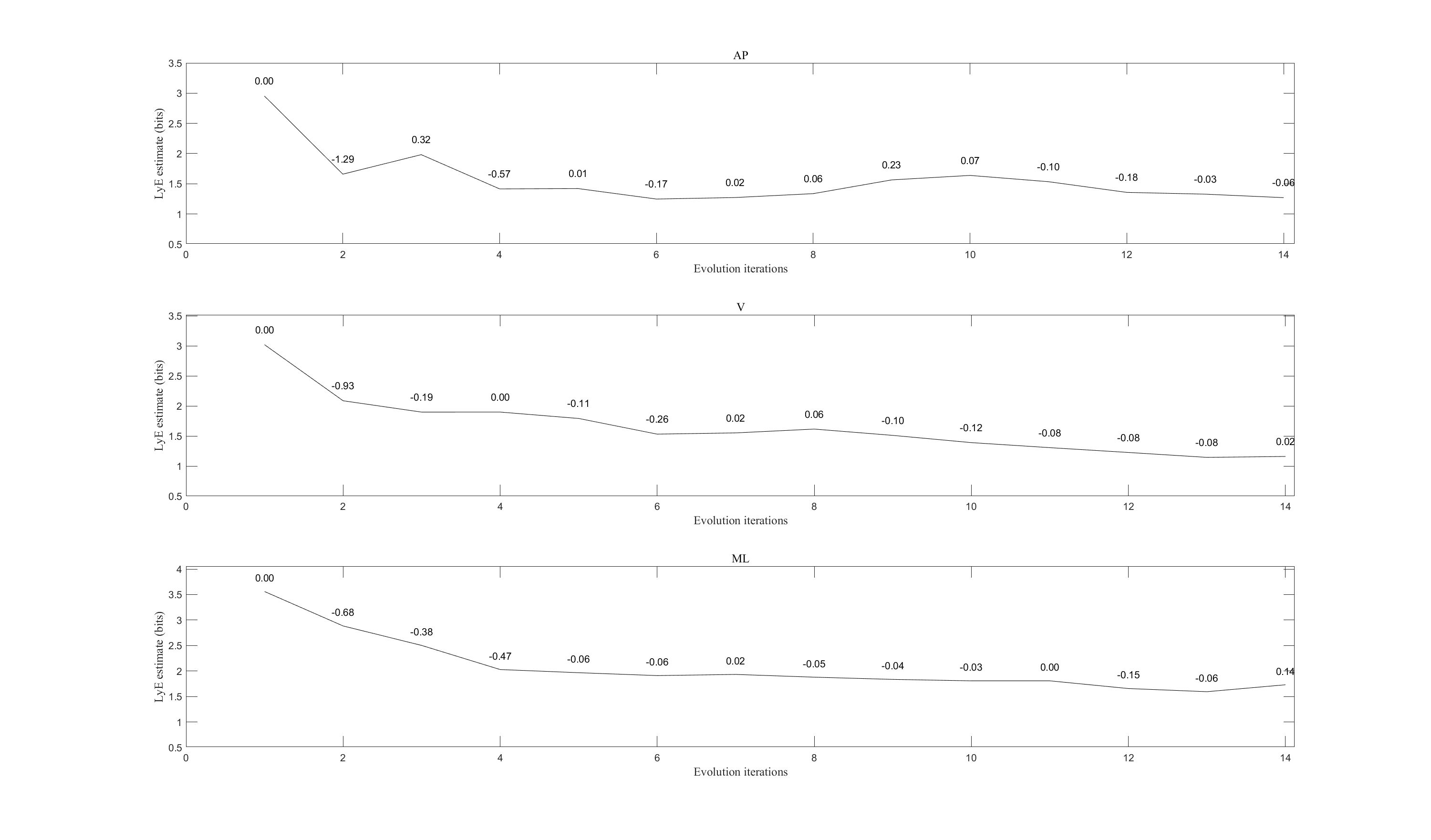

Supplement: Supplementary file 2 — Supplementary Information. [file 41598_2020_79584_MOESM2_ESM.zip › Participant5_trial11.png]

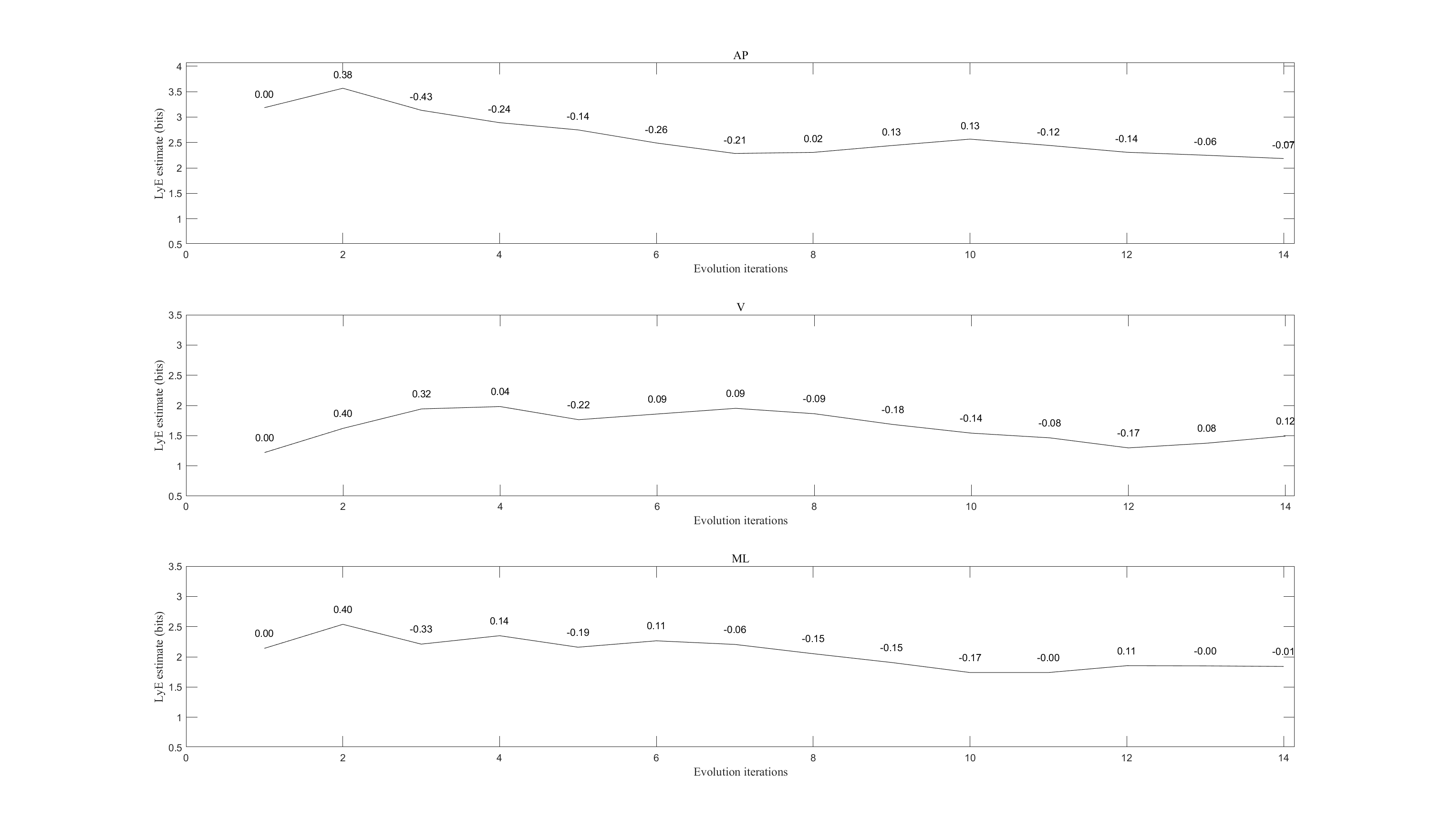

Supplement: Supplementary file 2 — Supplementary Information. [file 41598_2020_79584_MOESM2_ESM.zip › Participant5_trial12.png]

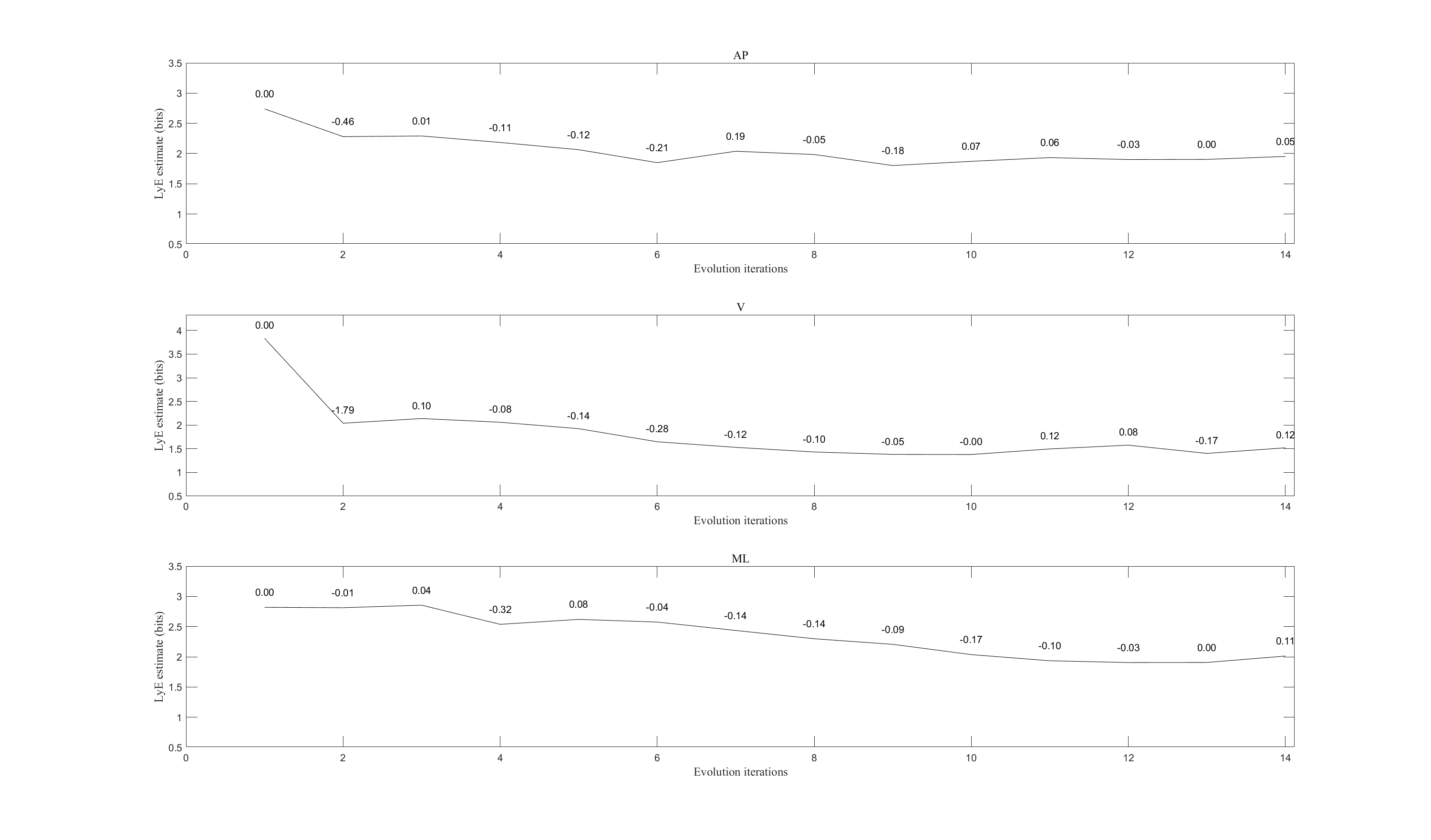

Supplement: Supplementary file 2 — Supplementary Information. [file 41598_2020_79584_MOESM2_ESM.zip › Participant5_trial2.png]

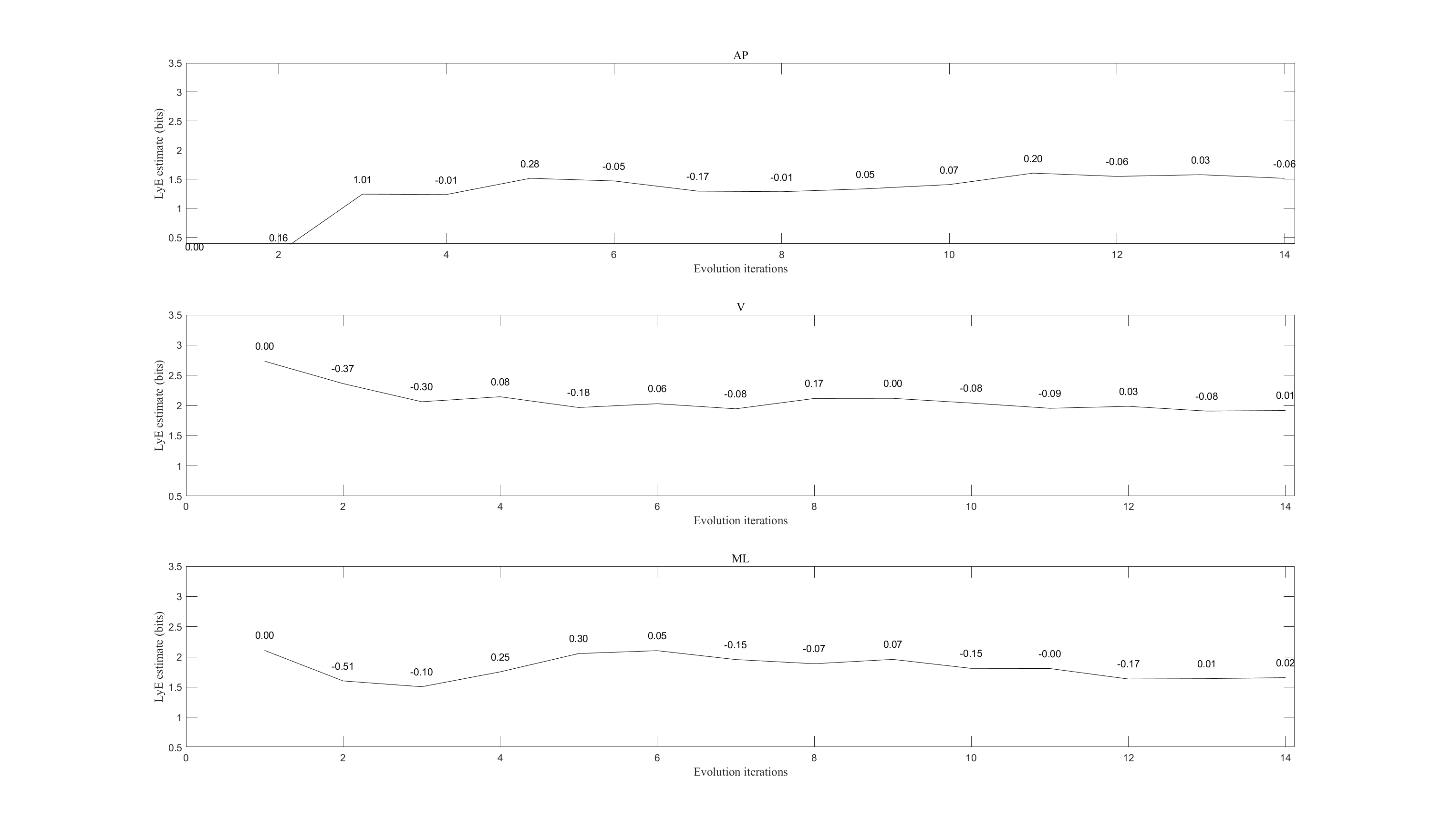

Supplement: Supplementary file 2 — Supplementary Information. [file 41598_2020_79584_MOESM2_ESM.zip › Participant5_trial3.png]

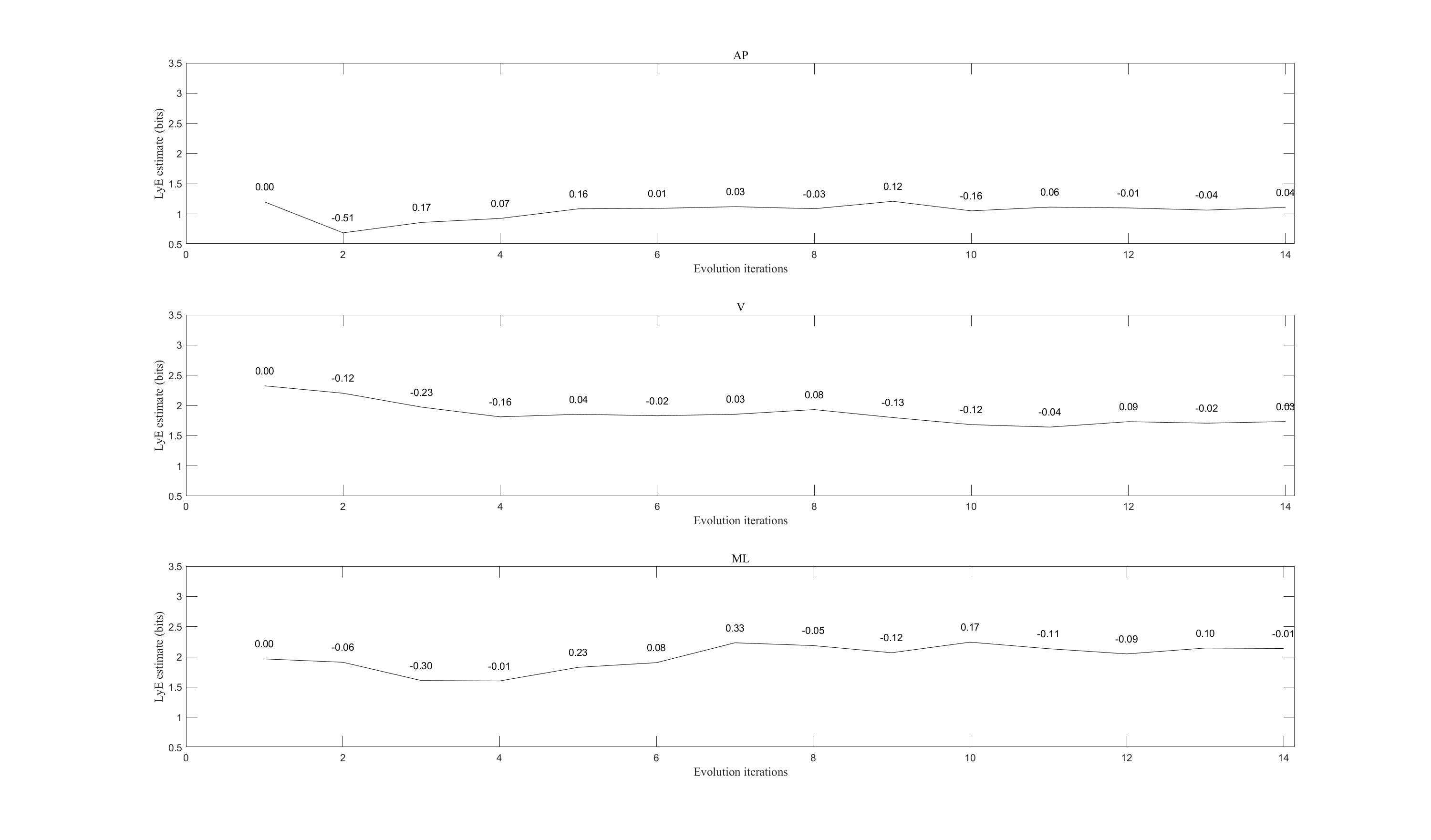

Supplement: Supplementary file 2 — Supplementary Information. [file 41598_2020_79584_MOESM2_ESM.zip › Participant5_trial4.png]

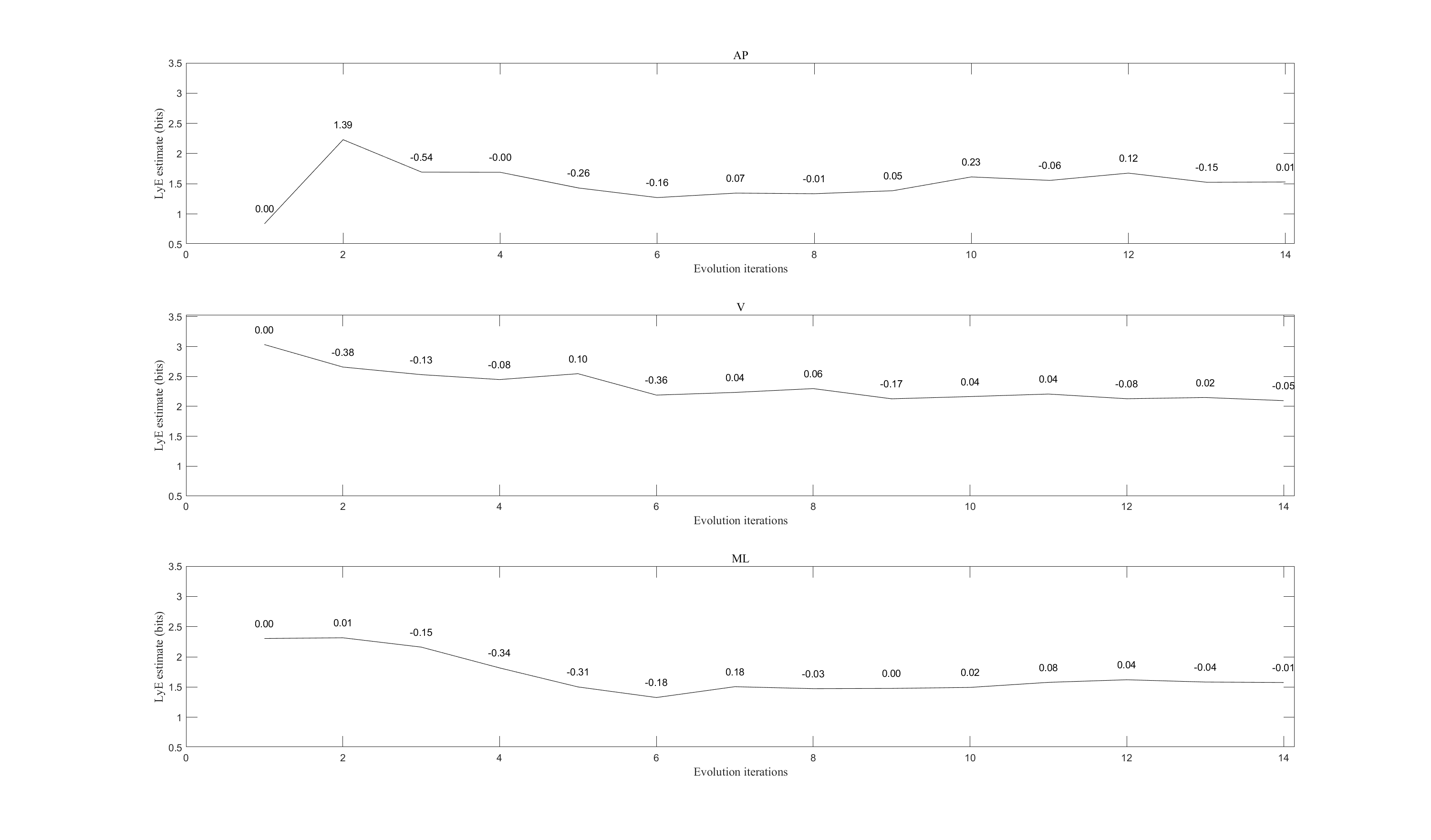

Supplement: Supplementary file 2 — Supplementary Information. [file 41598_2020_79584_MOESM2_ESM.zip › Participant5_trial5.png]

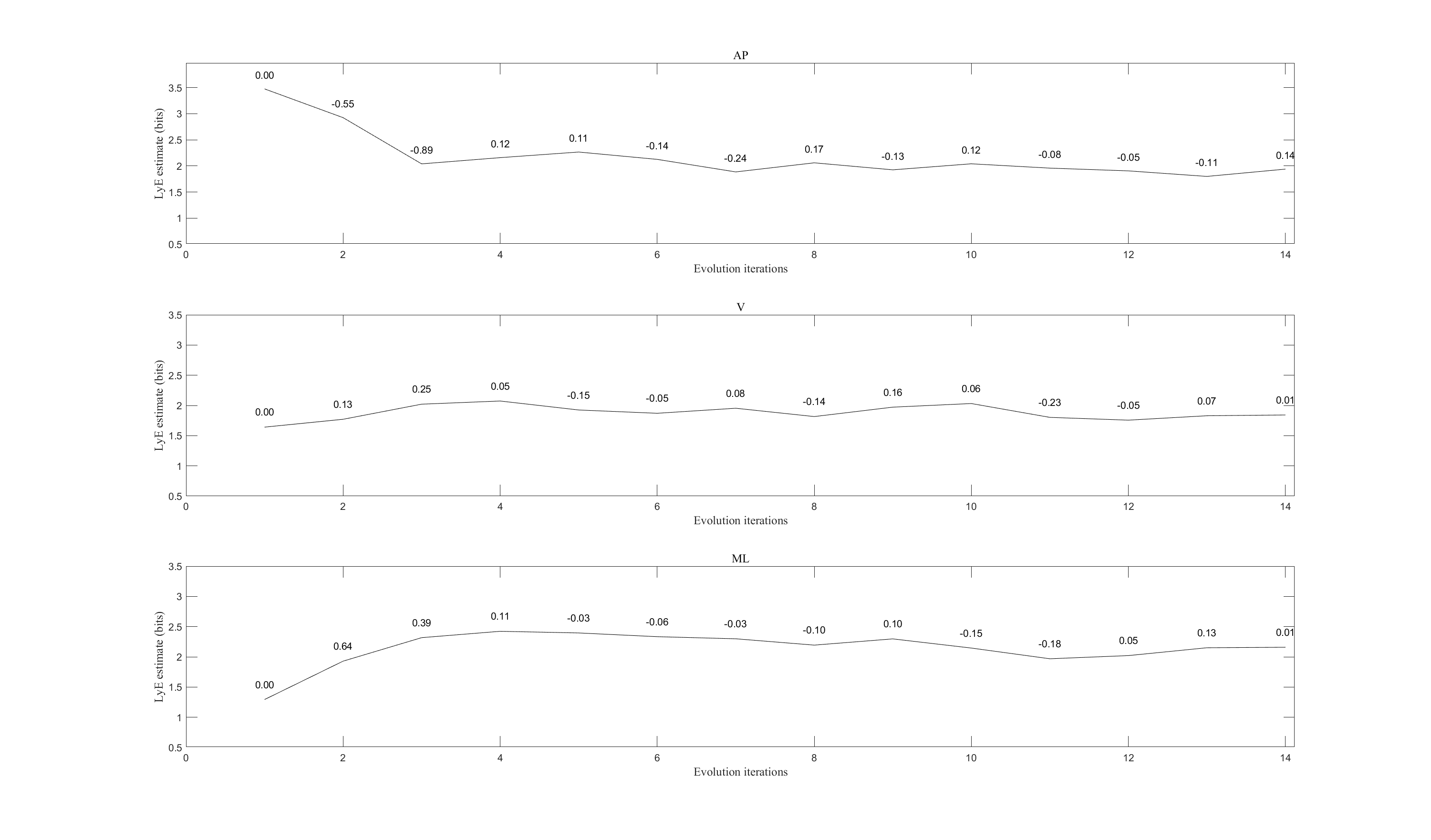

Supplement: Supplementary file 2 — Supplementary Information. [file 41598_2020_79584_MOESM2_ESM.zip › Participant5_trial6.png]

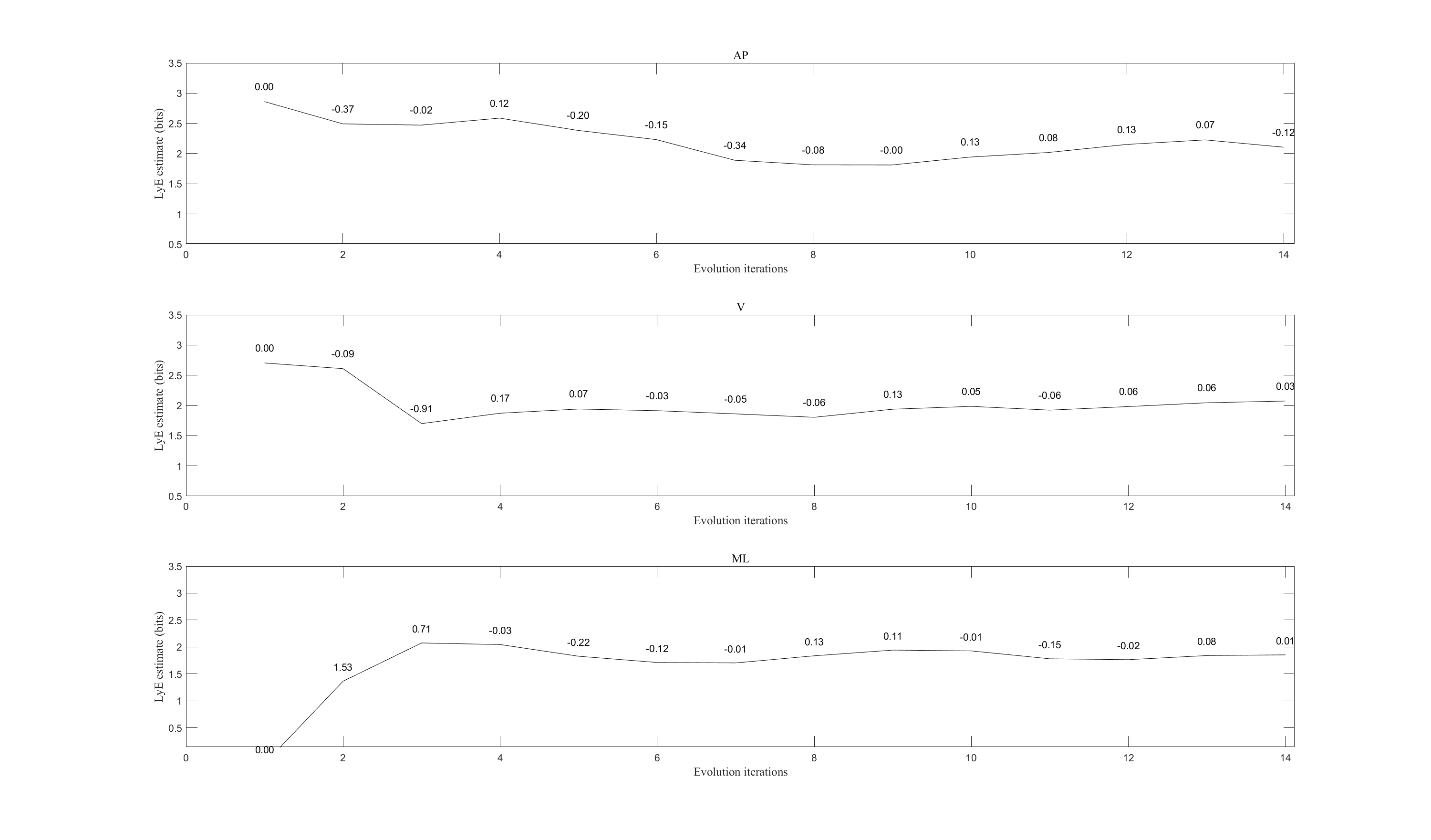

Supplement: Supplementary file 2 — Supplementary Information. [file 41598_2020_79584_MOESM2_ESM.zip › Participant5_trial7.png]

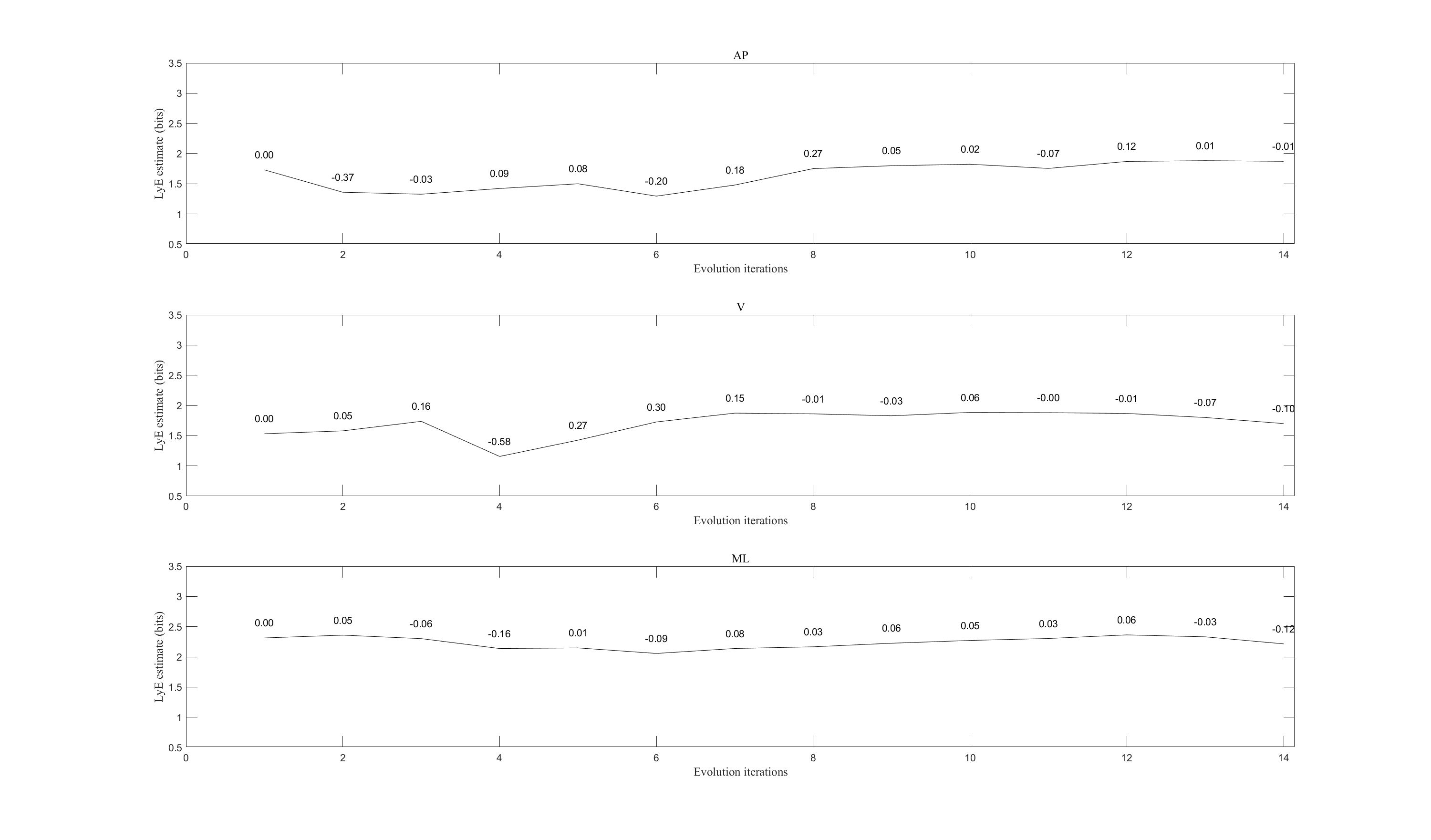

Supplement: Supplementary file 2 — Supplementary Information. [file 41598_2020_79584_MOESM2_ESM.zip › Participant5_trial8.png]

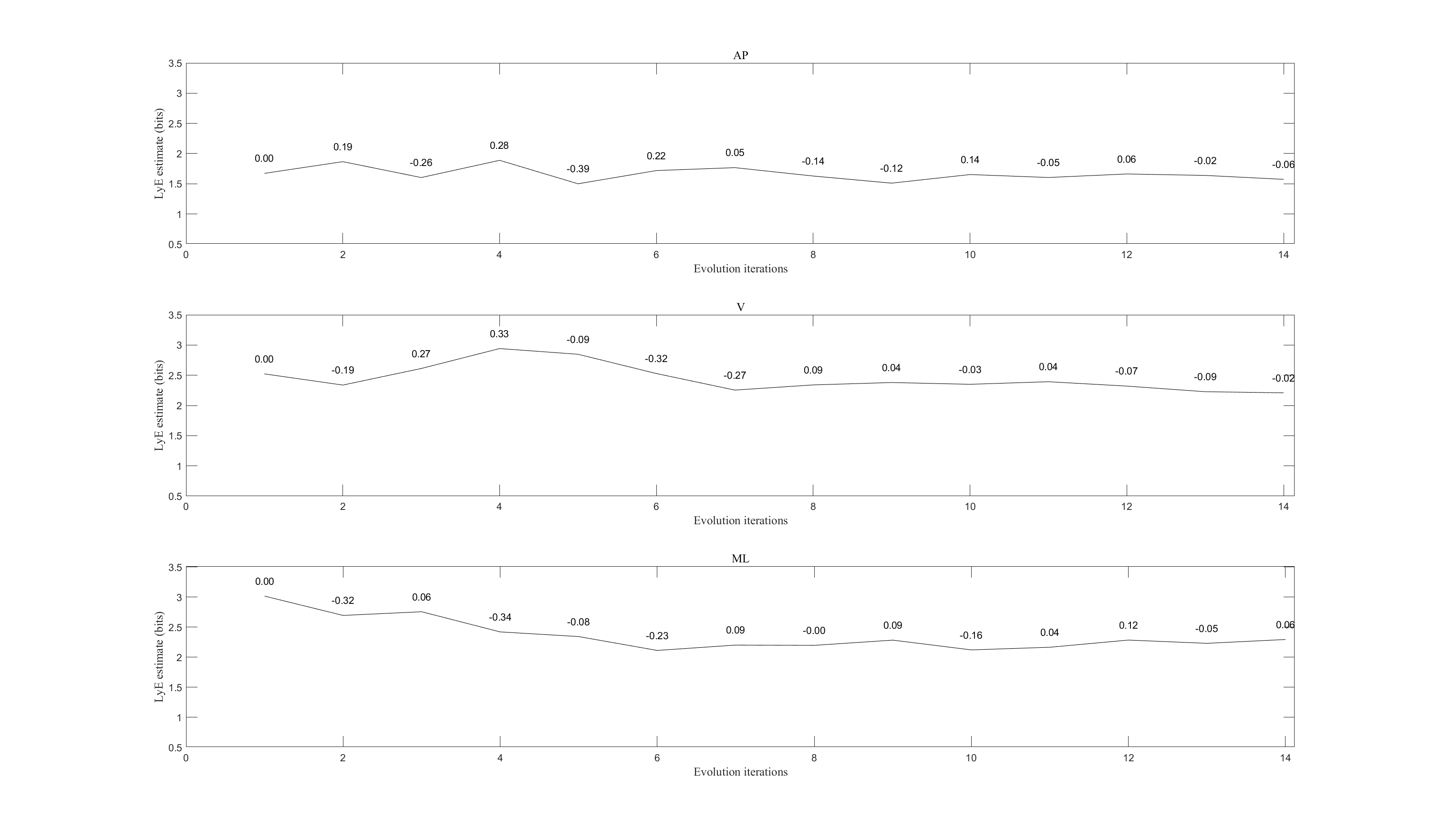

Supplement: Supplementary file 2 — Supplementary Information. [file 41598_2020_79584_MOESM2_ESM.zip › Participant5_trial9.png]

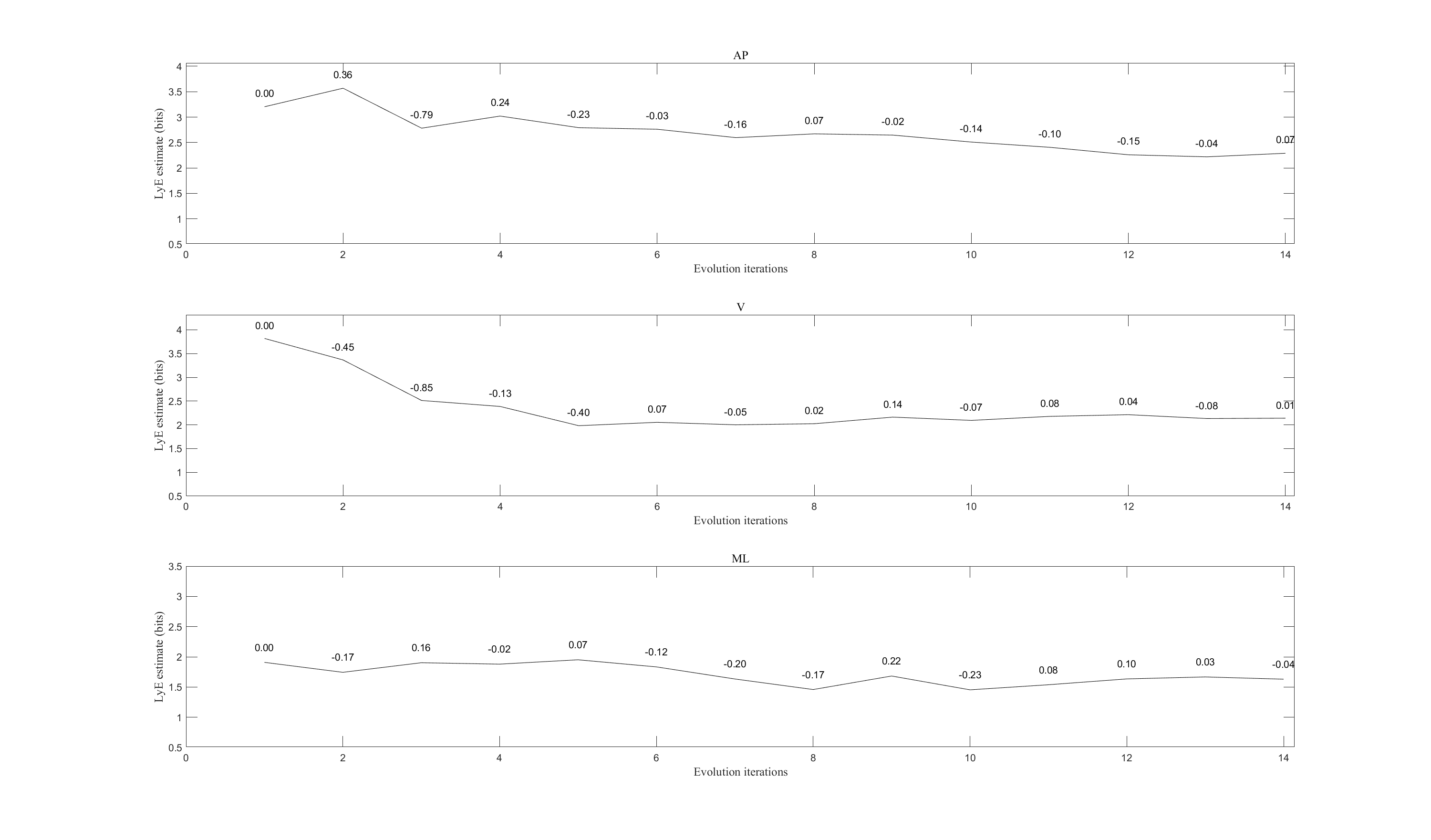

Supplement: Supplementary file 2 — Supplementary Information. [file 41598_2020_79584_MOESM2_ESM.zip › Participant6_trial1.png]

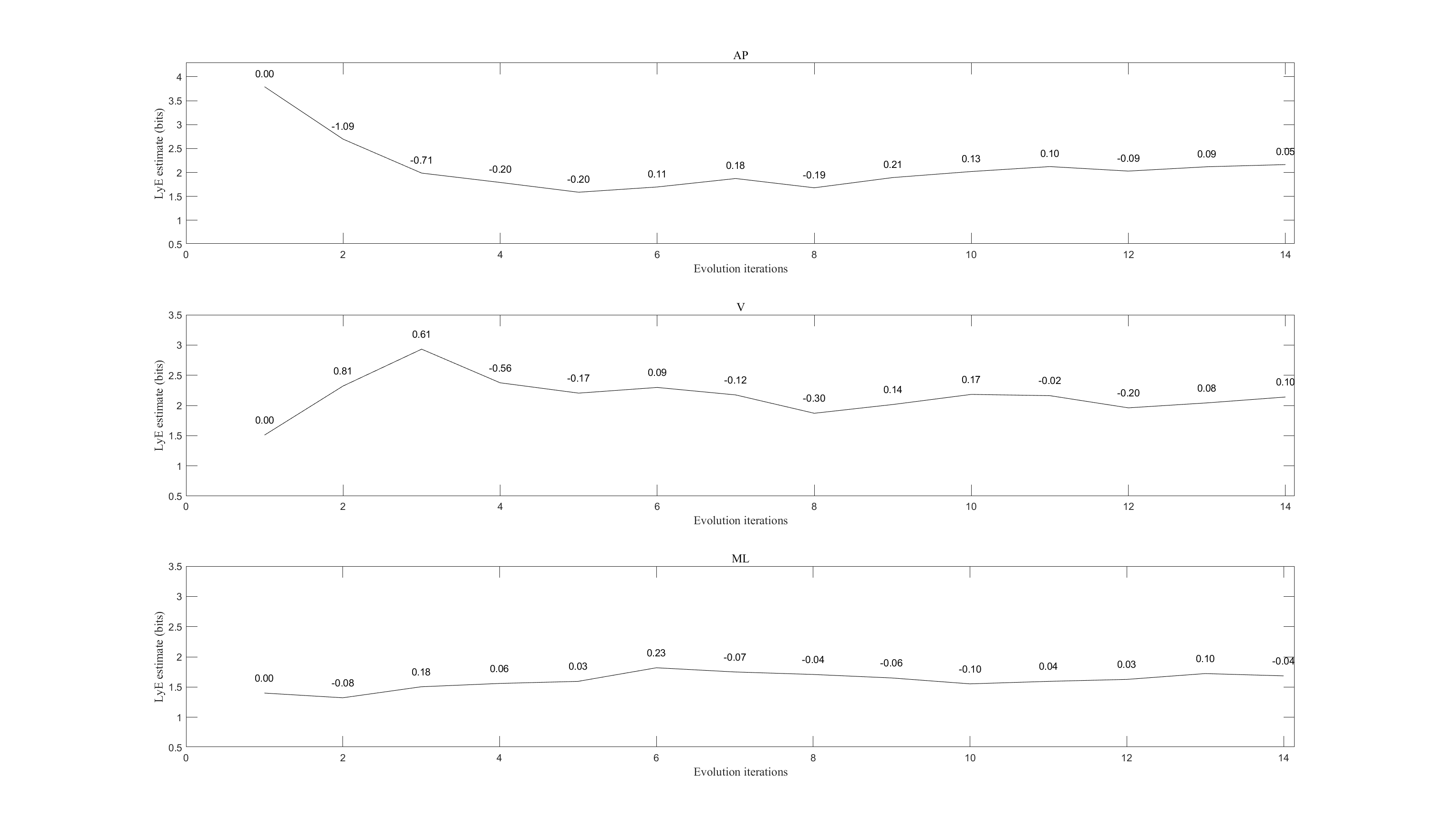

Supplement: Supplementary file 2 — Supplementary Information. [file 41598_2020_79584_MOESM2_ESM.zip › Participant6_trial10.png]

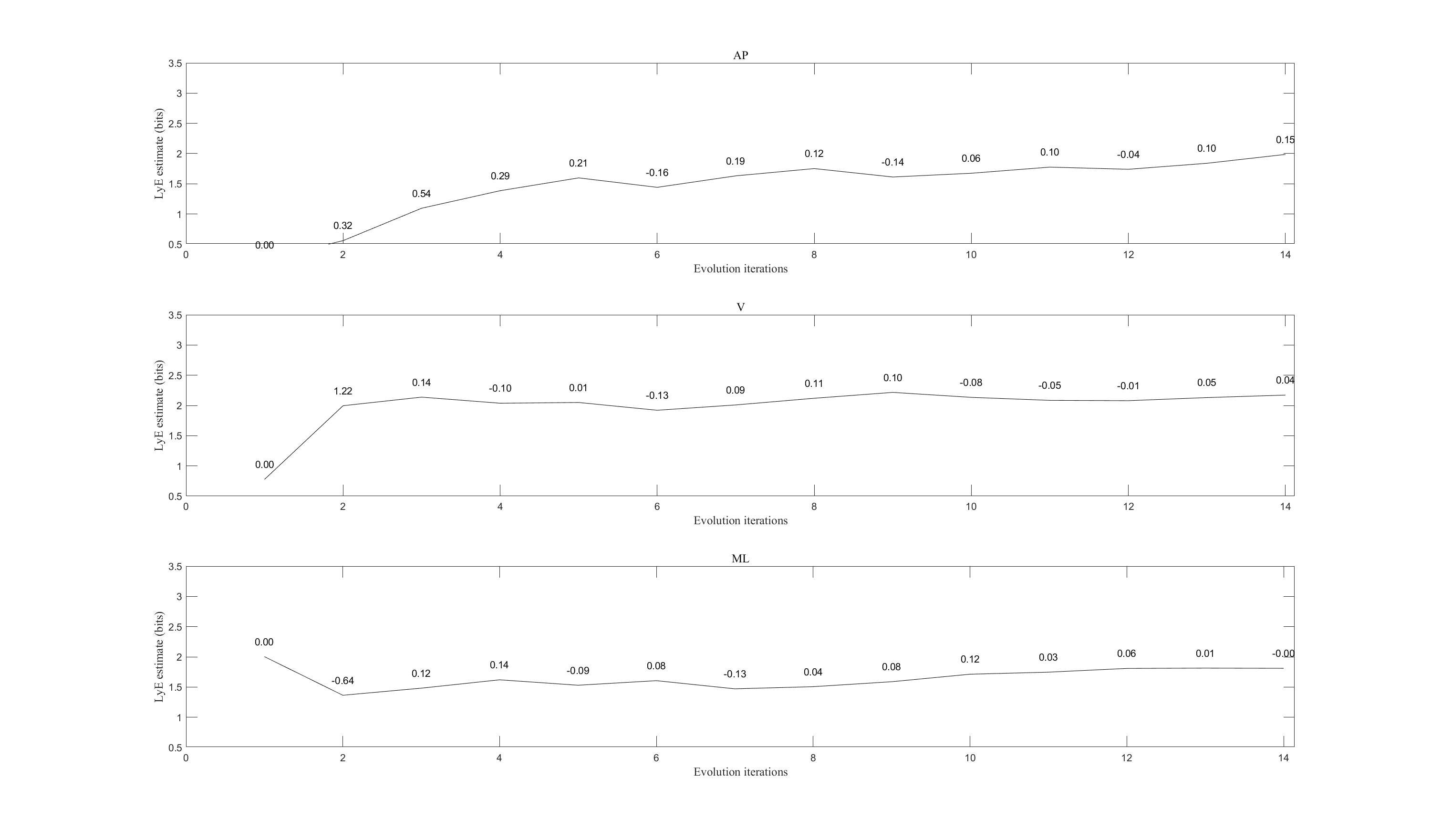

Supplement: Supplementary file 2 — Supplementary Information. [file 41598_2020_79584_MOESM2_ESM.zip › Participant6_trial11.png]

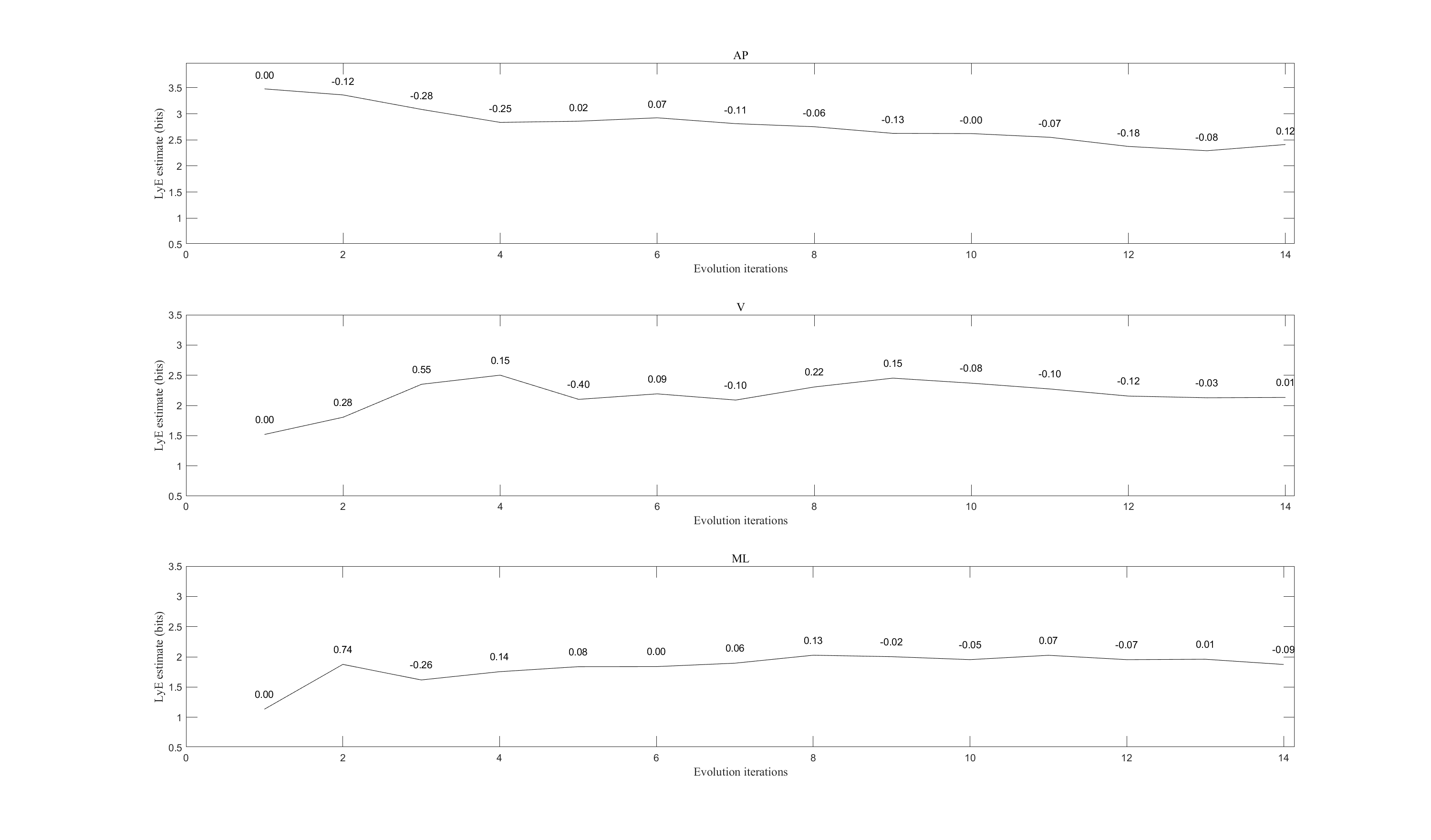

Supplement: Supplementary file 2 — Supplementary Information. [file 41598_2020_79584_MOESM2_ESM.zip › Participant6_trial12.png]

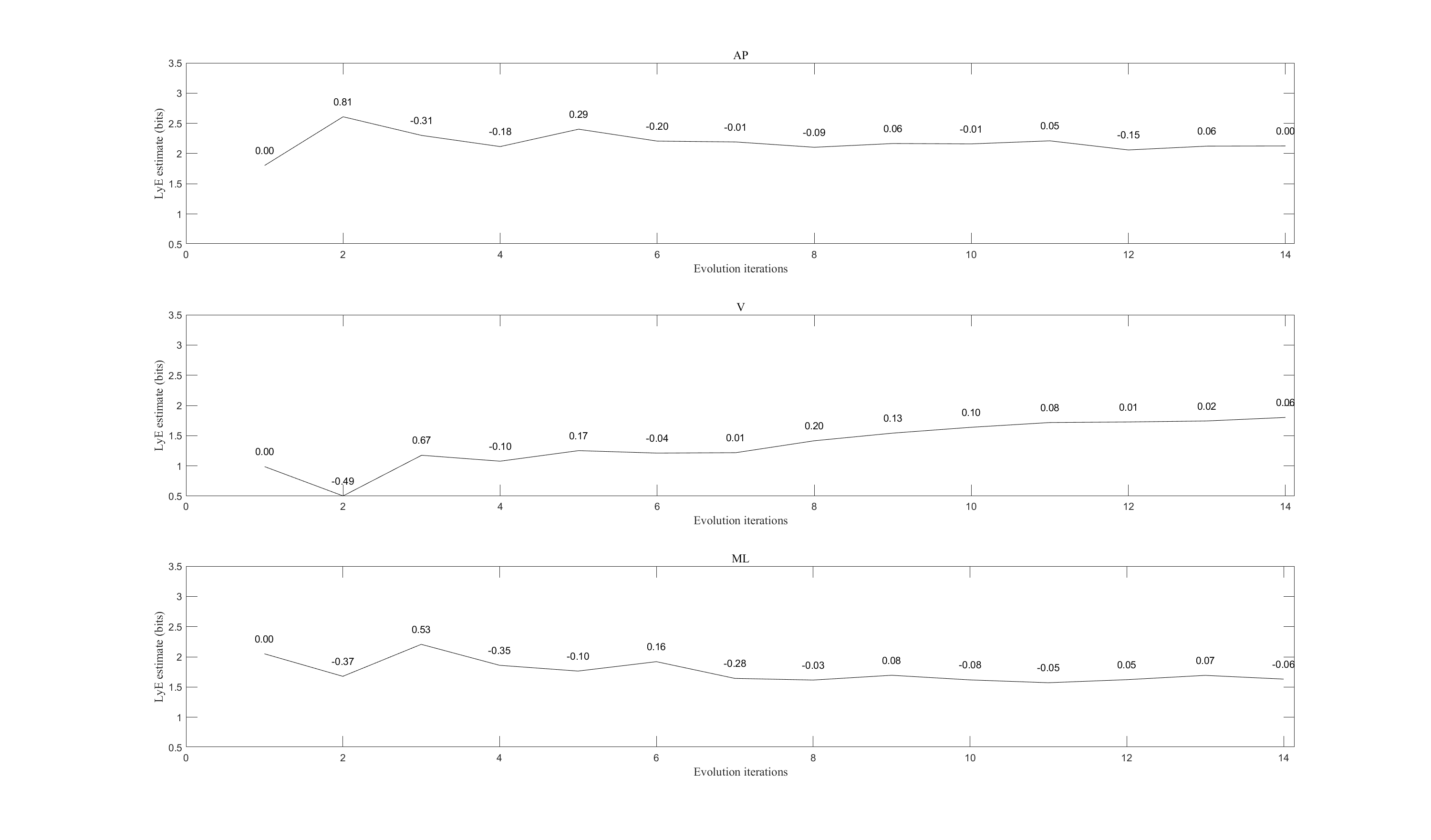

Supplement: Supplementary file 2 — Supplementary Information. [file 41598_2020_79584_MOESM2_ESM.zip › Participant6_trial2.png]

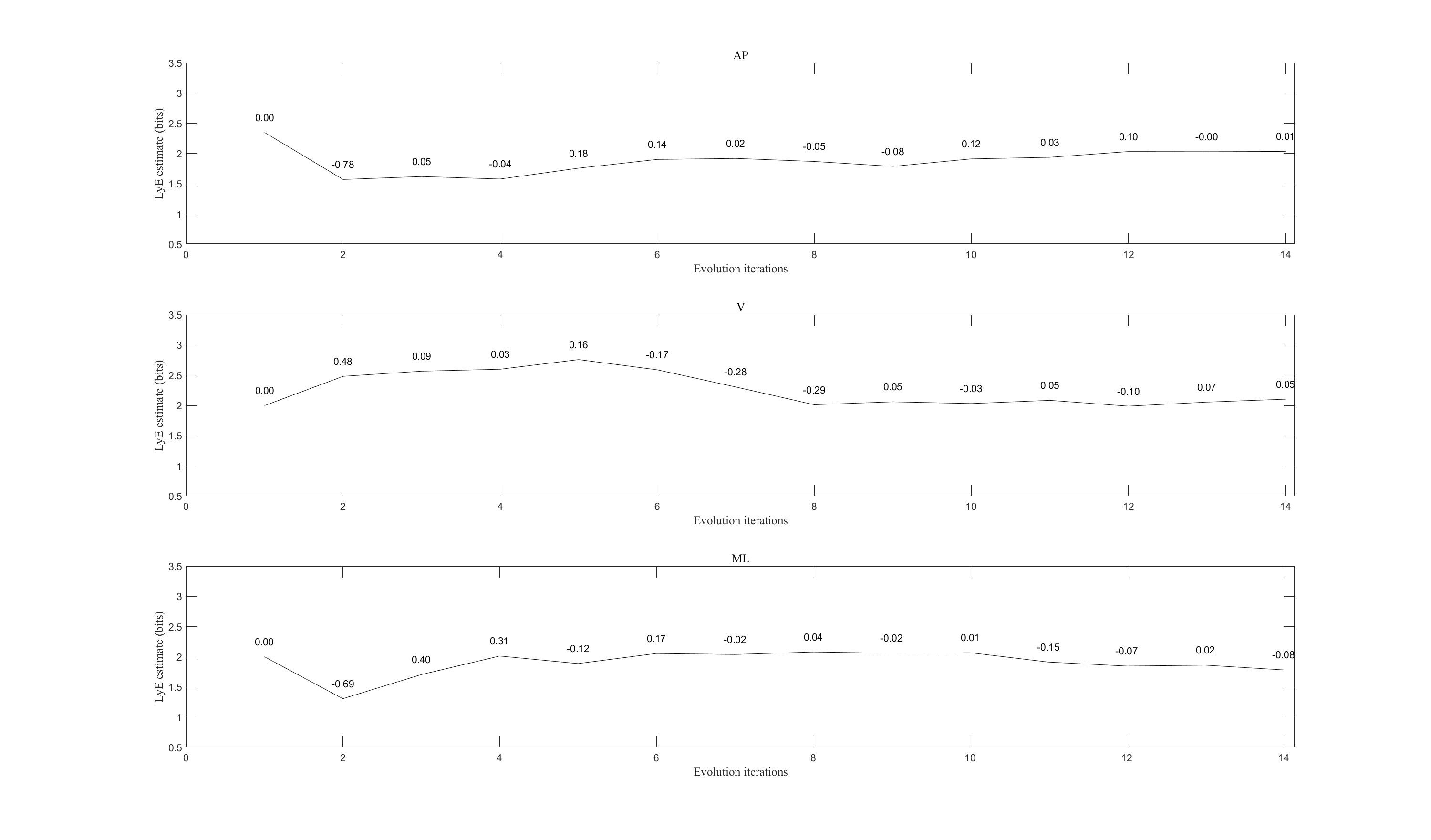

Supplement: Supplementary file 2 — Supplementary Information. [file 41598_2020_79584_MOESM2_ESM.zip › Participant6_trial3.png]

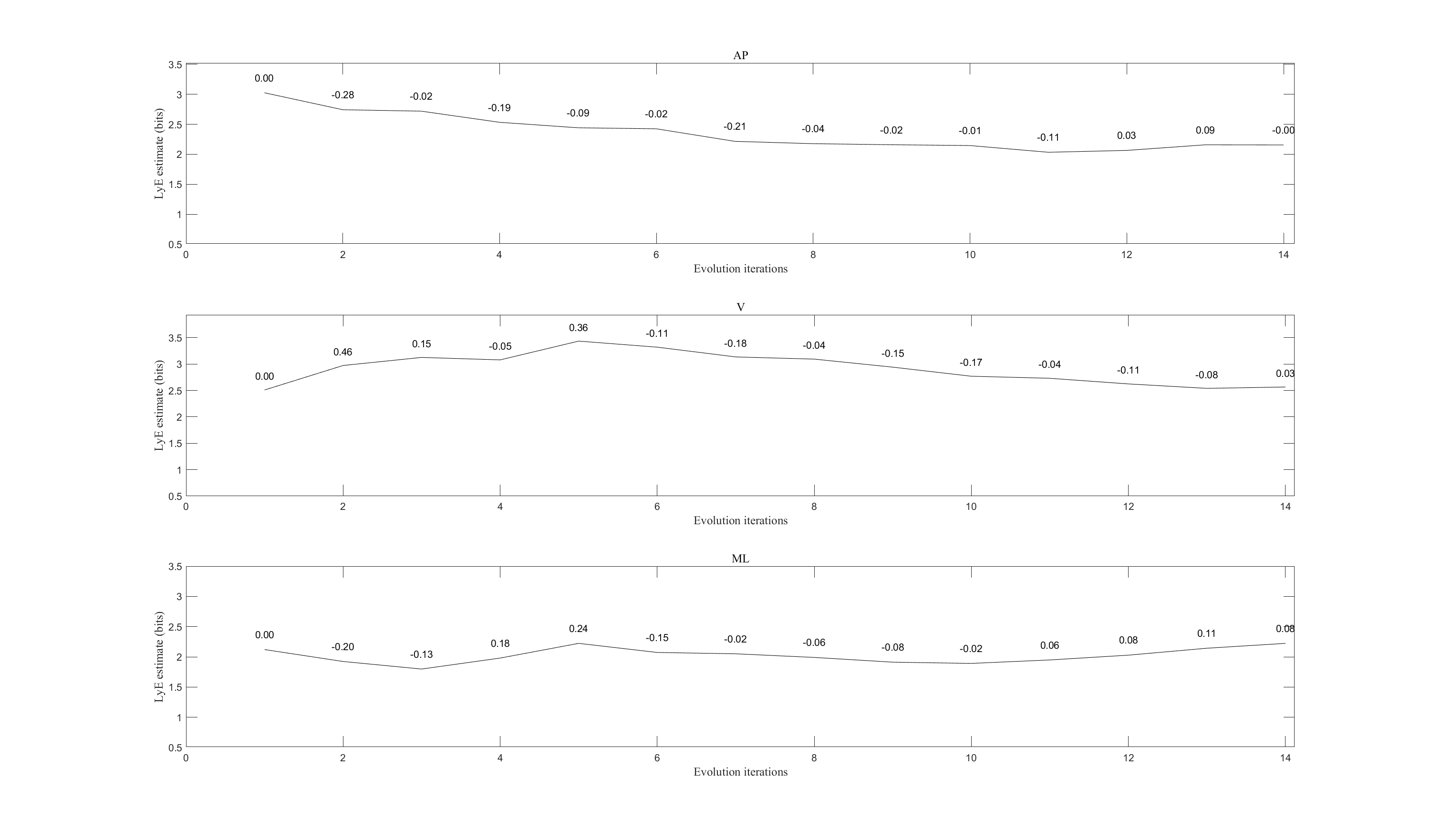

Supplement: Supplementary file 2 — Supplementary Information. [file 41598_2020_79584_MOESM2_ESM.zip › Participant6_trial4.png]

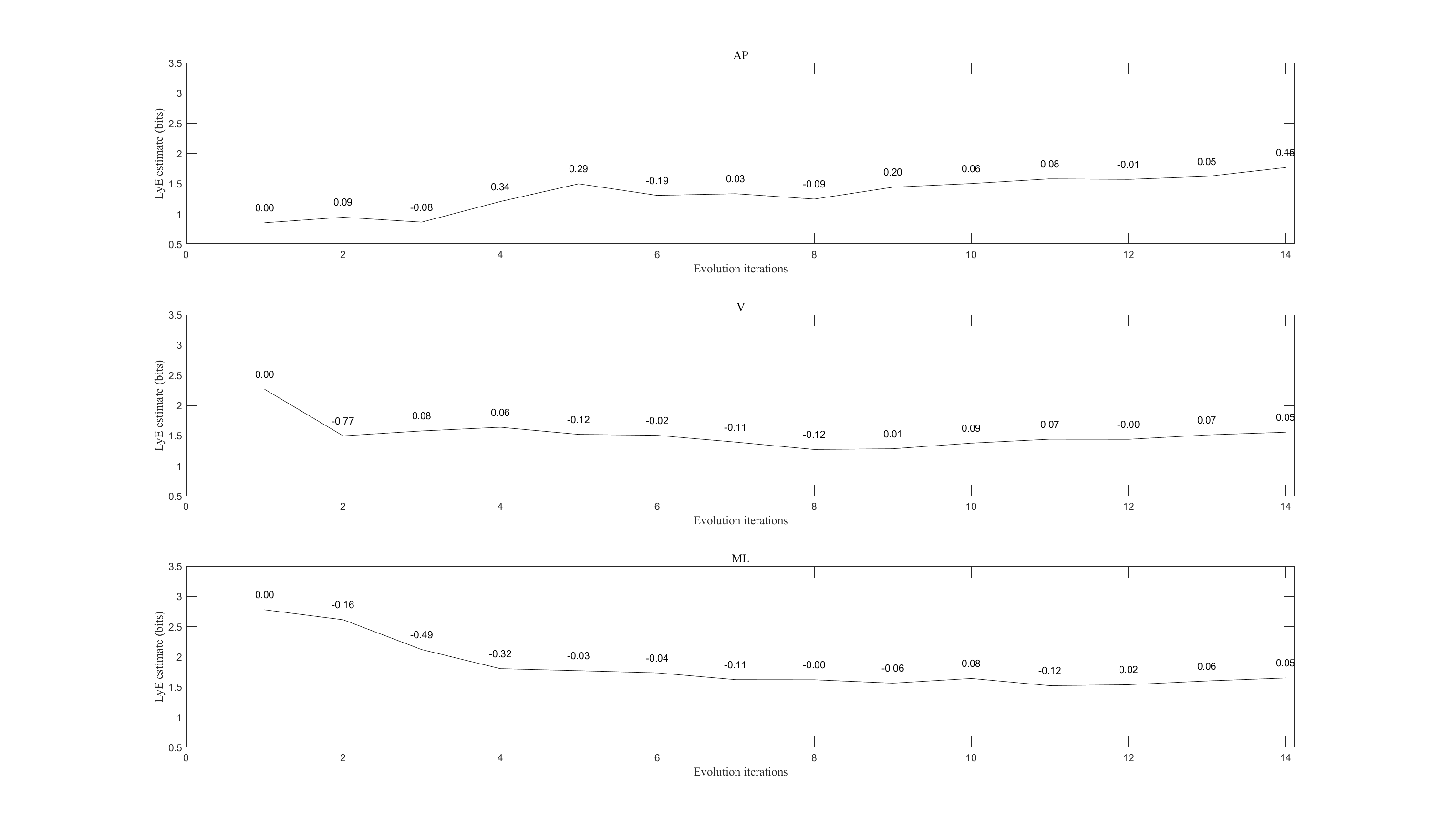

Supplement: Supplementary file 2 — Supplementary Information. [file 41598_2020_79584_MOESM2_ESM.zip › Participant6_trial5.png]
