# Supplementary figures and images for: The effects of walking speed and mobile phone use on the walking dynamics of young adults (part 1 of 2)
Source: Sci Rep. 2021 Jan 13;11:1237. doi: 10.1038/s41598-020-79584-5 (PMC7806980; doi:10.1038/s41598-020-79584-5)

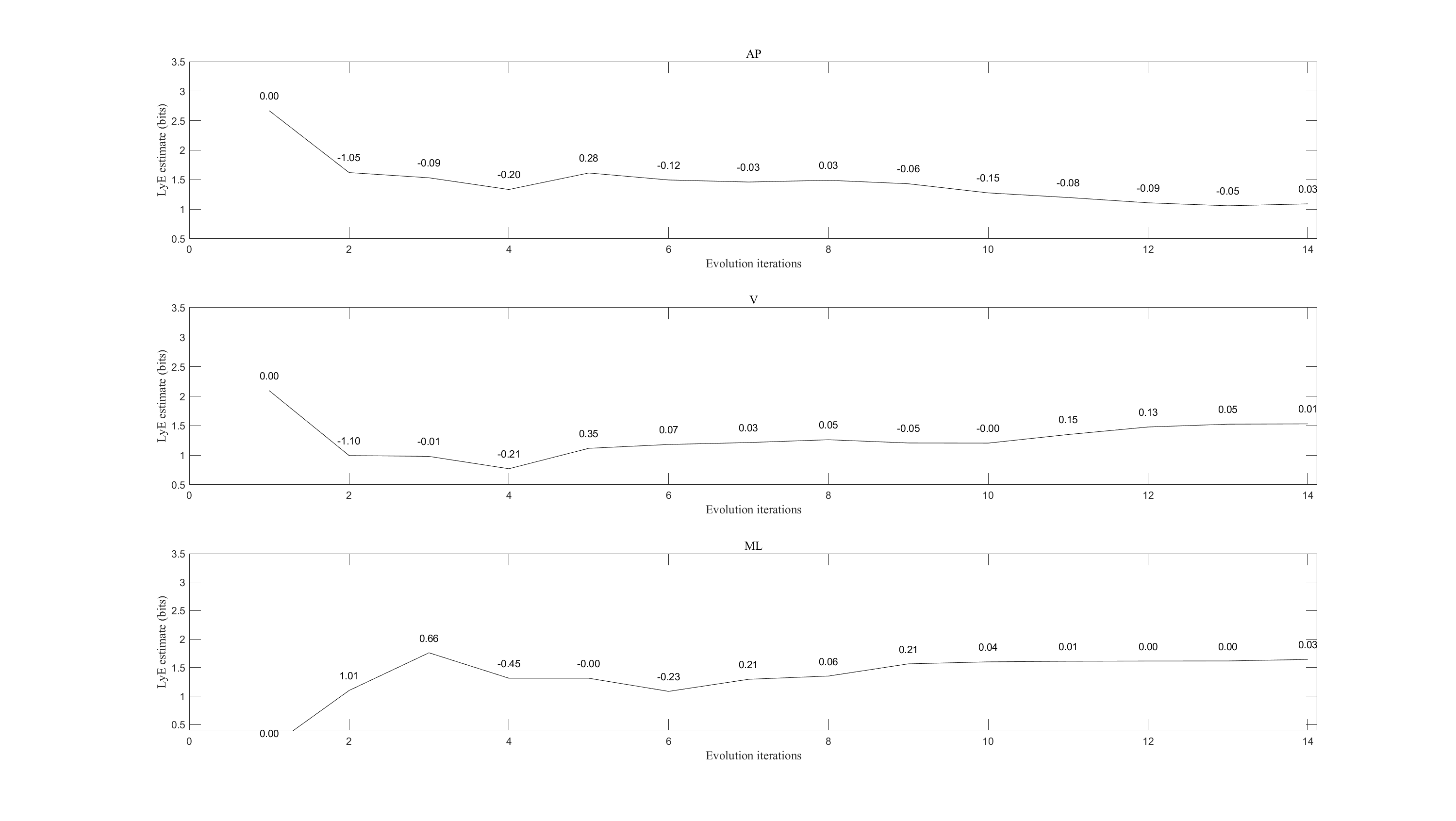

Supplement: Supplementary file 2 — Supplementary Information. [file 41598_2020_79584_MOESM2_ESM.zip › Participant1_trial1.png]

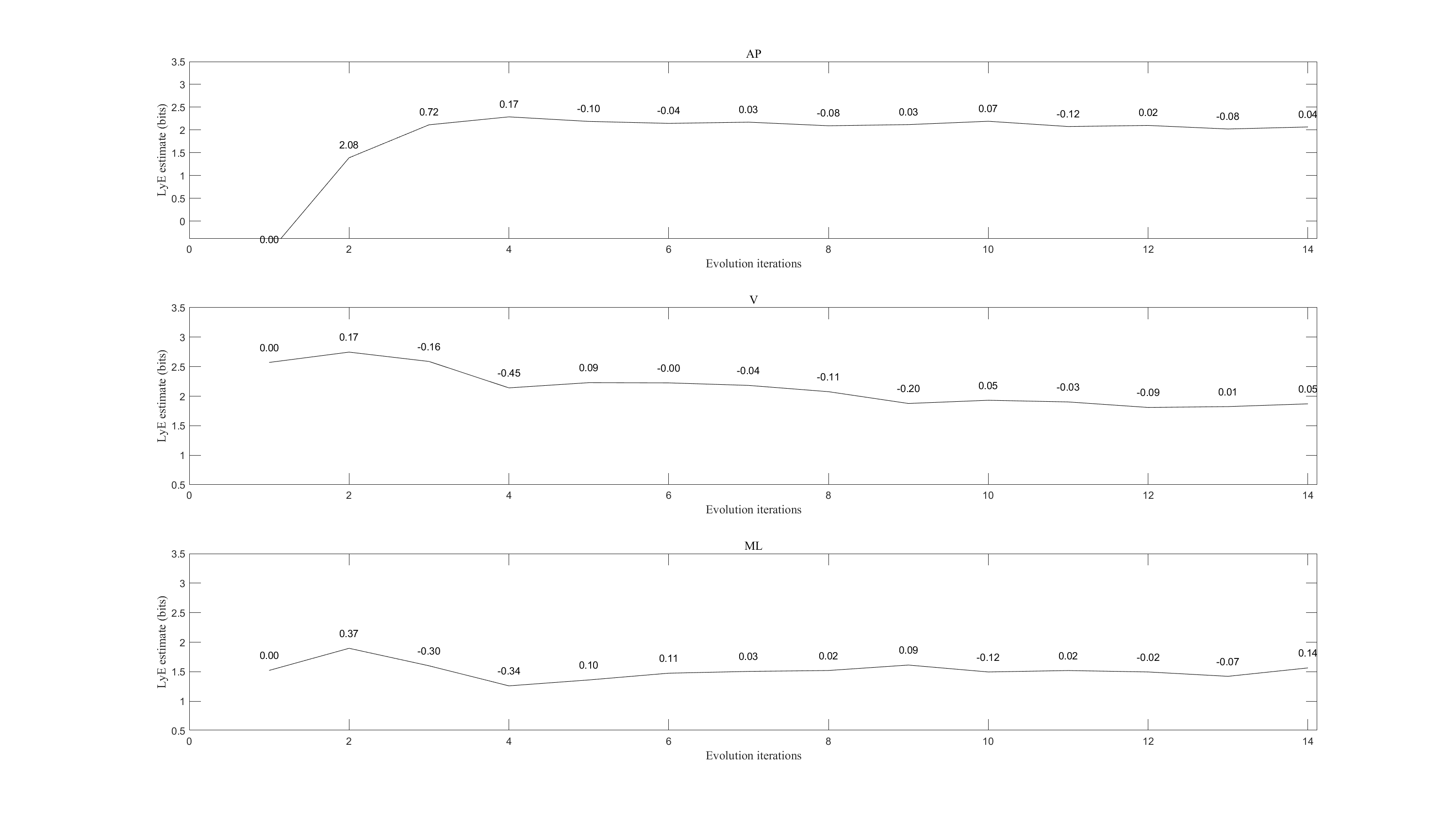

Supplement: Supplementary file 2 — Supplementary Information. [file 41598_2020_79584_MOESM2_ESM.zip › Participant1_trial10.png]

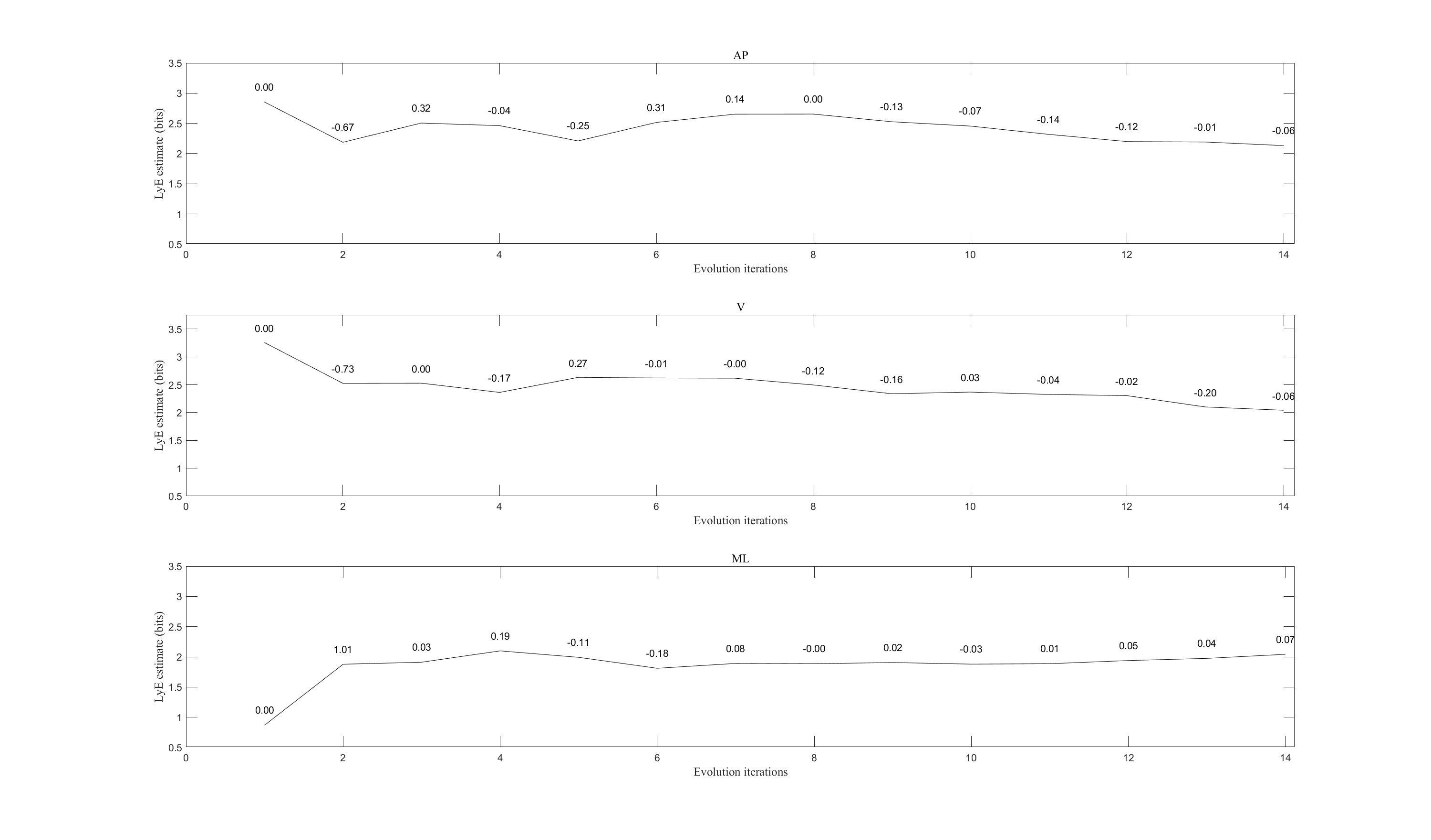

Supplement: Supplementary file 2 — Supplementary Information. [file 41598_2020_79584_MOESM2_ESM.zip › Participant1_trial11.png]

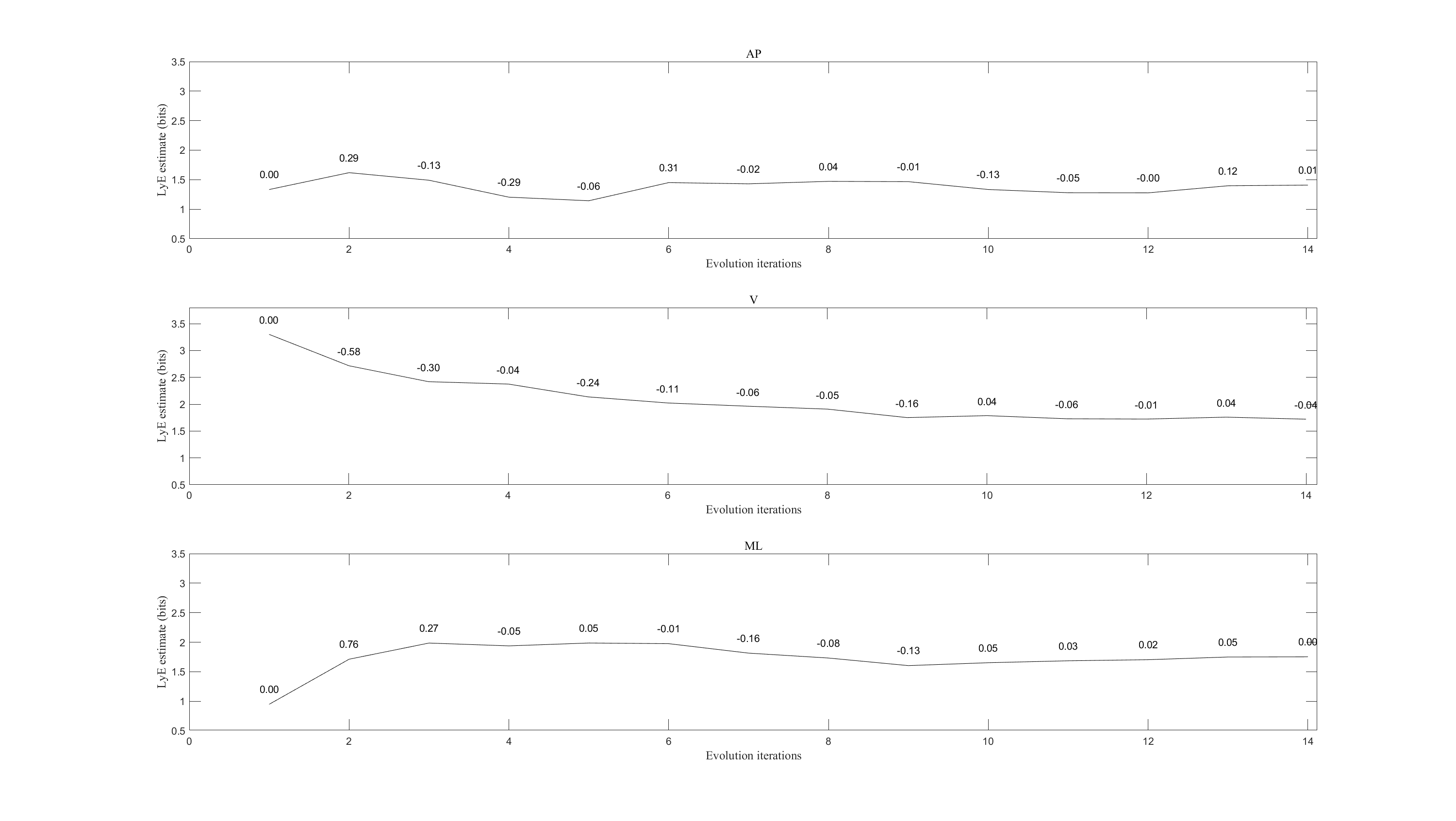

Supplement: Supplementary file 2 — Supplementary Information. [file 41598_2020_79584_MOESM2_ESM.zip › Participant1_trial12.png]

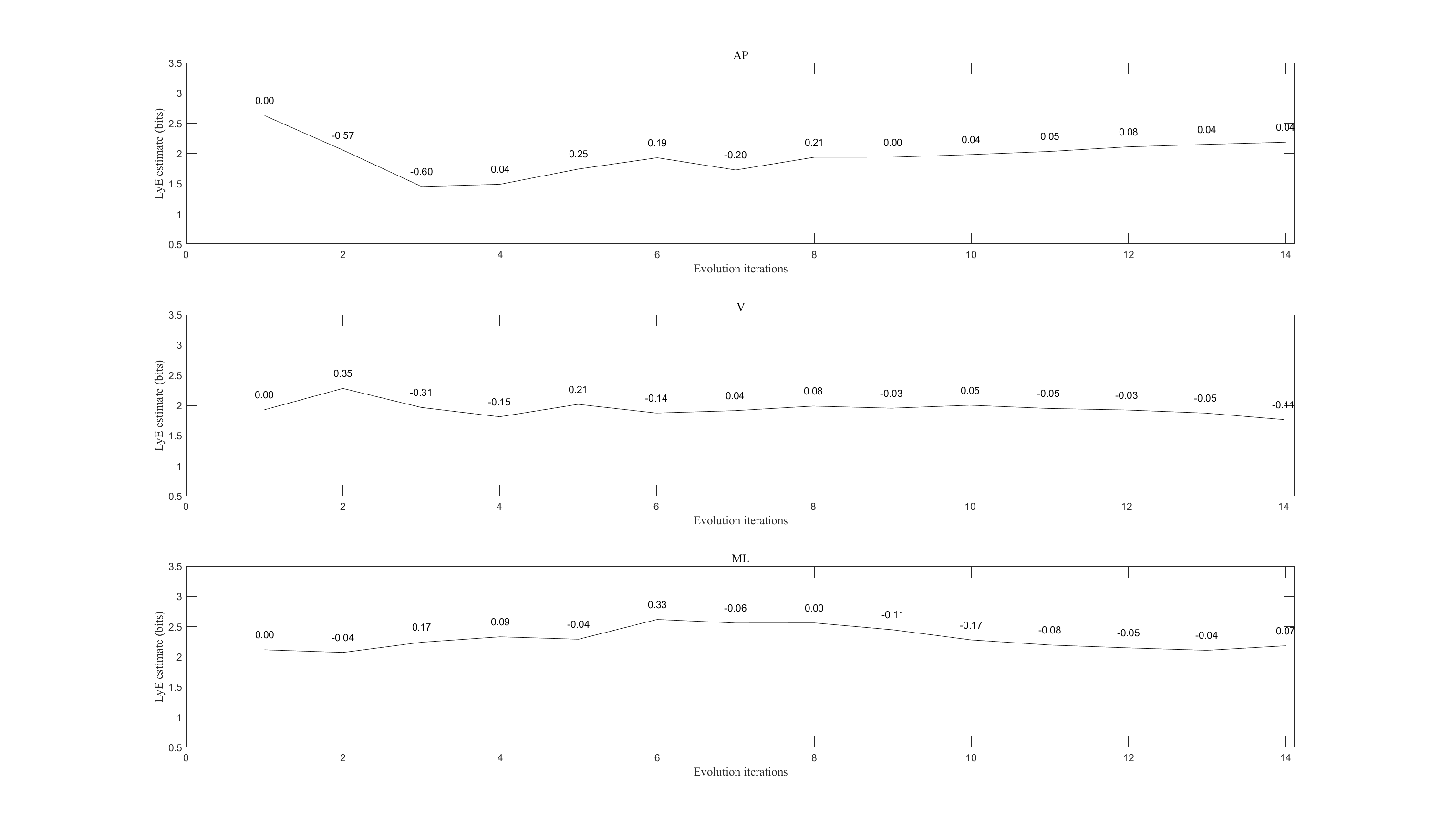

Supplement: Supplementary file 2 — Supplementary Information. [file 41598_2020_79584_MOESM2_ESM.zip › Participant1_trial2.png]

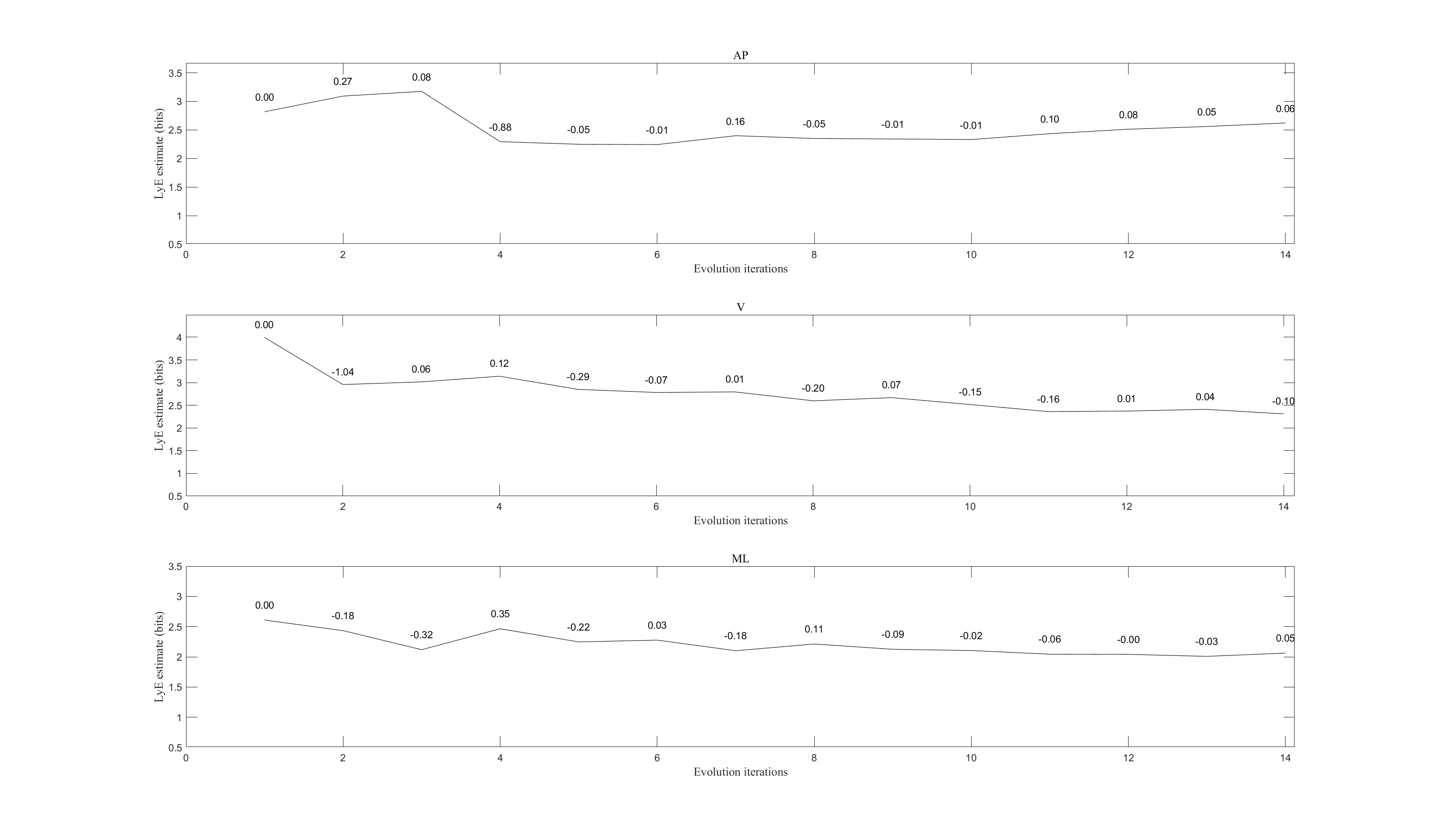

Supplement: Supplementary file 2 — Supplementary Information. [file 41598_2020_79584_MOESM2_ESM.zip › Participant1_trial3.png]

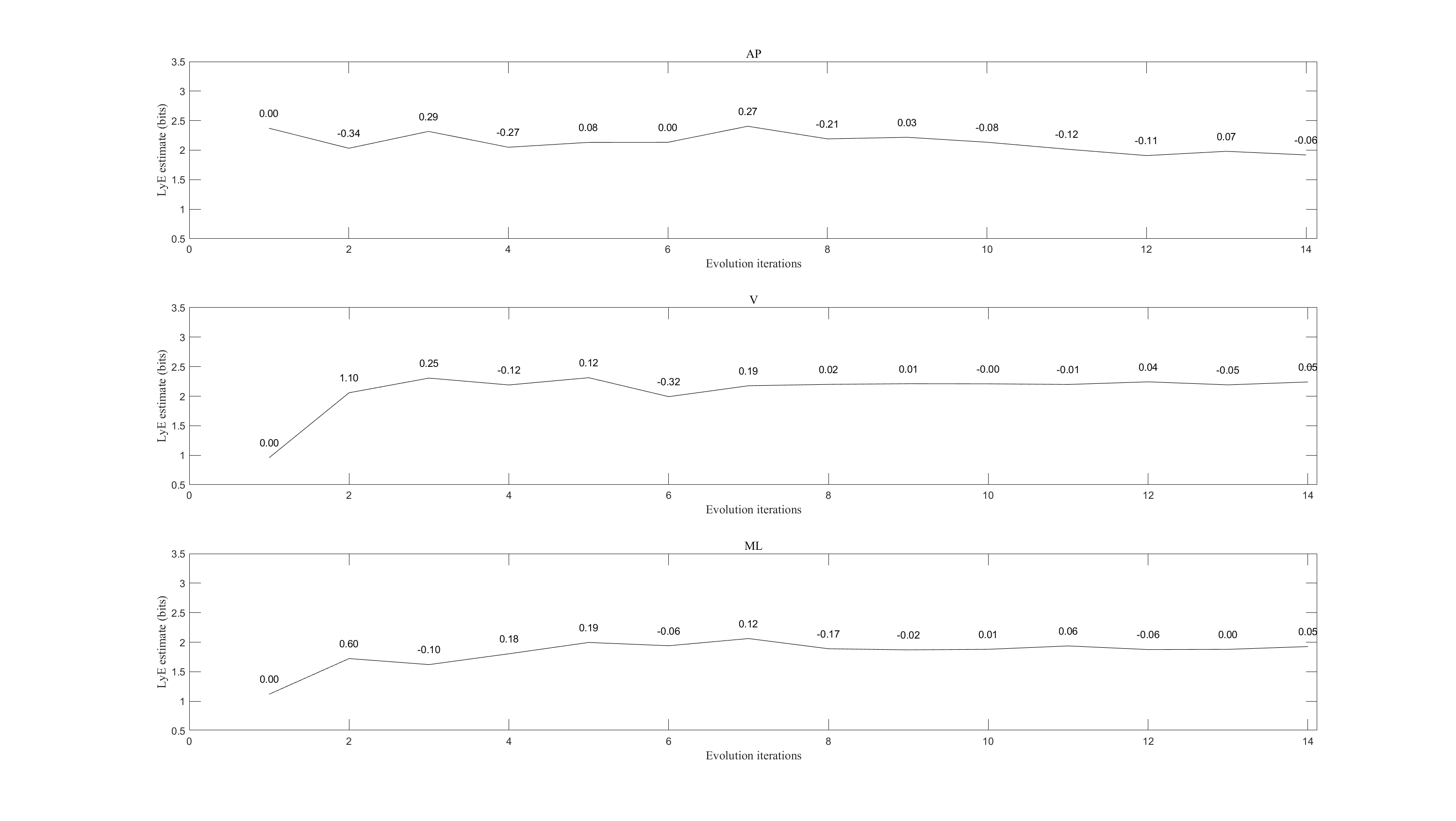

Supplement: Supplementary file 2 — Supplementary Information. [file 41598_2020_79584_MOESM2_ESM.zip › Participant1_trial4.png]

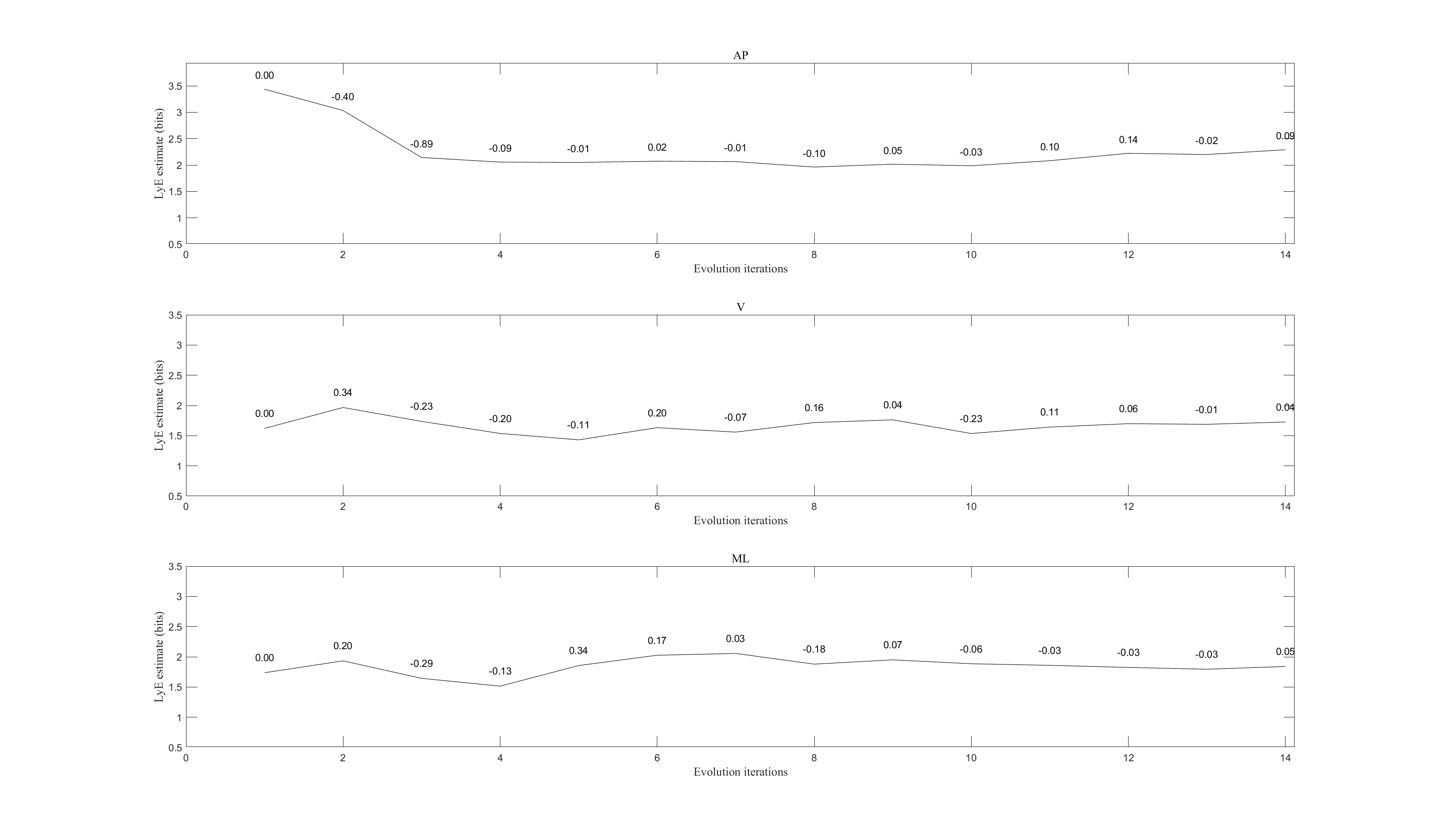

Supplement: Supplementary file 2 — Supplementary Information. [file 41598_2020_79584_MOESM2_ESM.zip › Participant1_trial5.png]

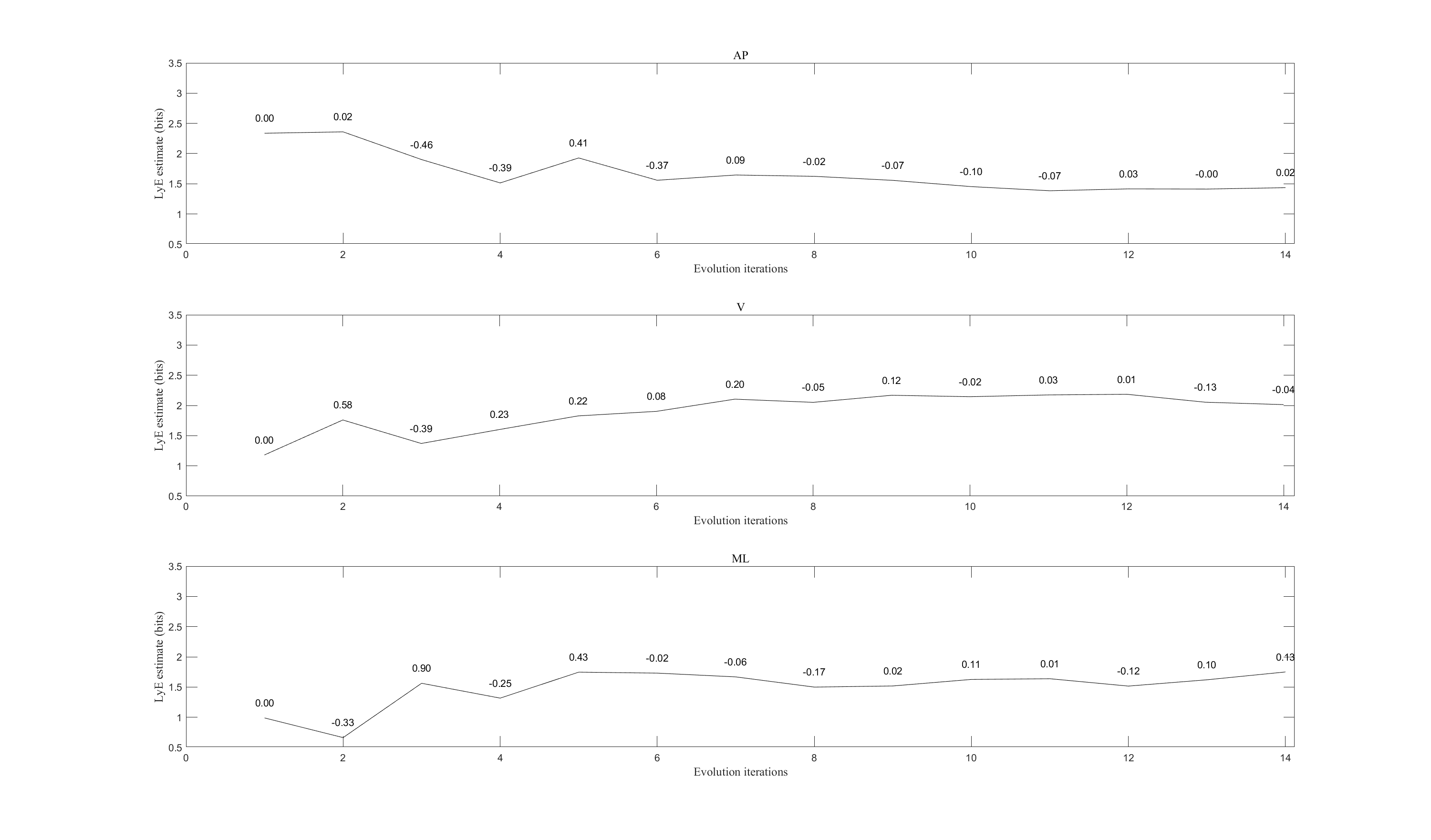

Supplement: Supplementary file 2 — Supplementary Information. [file 41598_2020_79584_MOESM2_ESM.zip › Participant1_trial6.png]

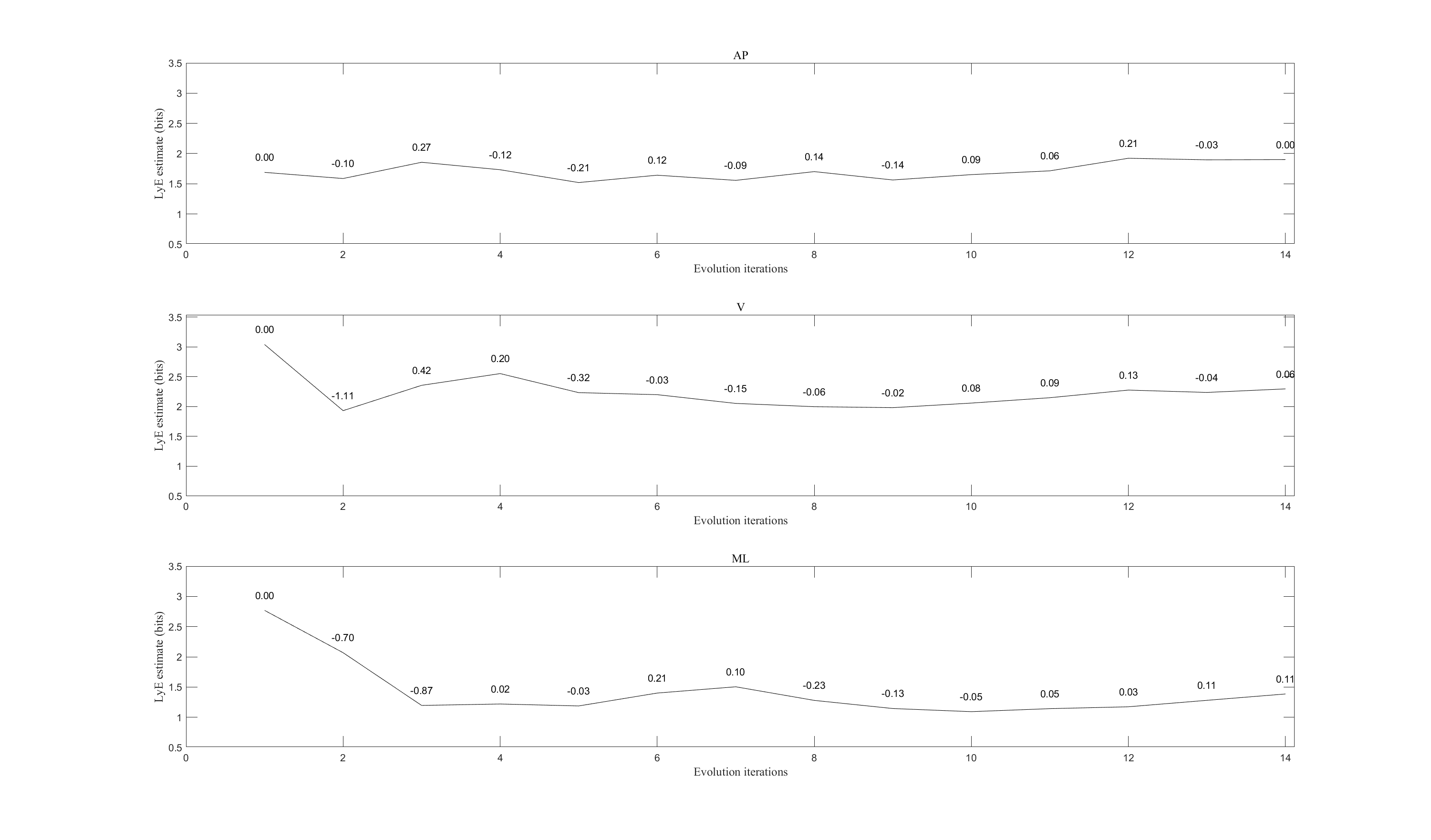

Supplement: Supplementary file 2 — Supplementary Information. [file 41598_2020_79584_MOESM2_ESM.zip › Participant1_trial7.png]

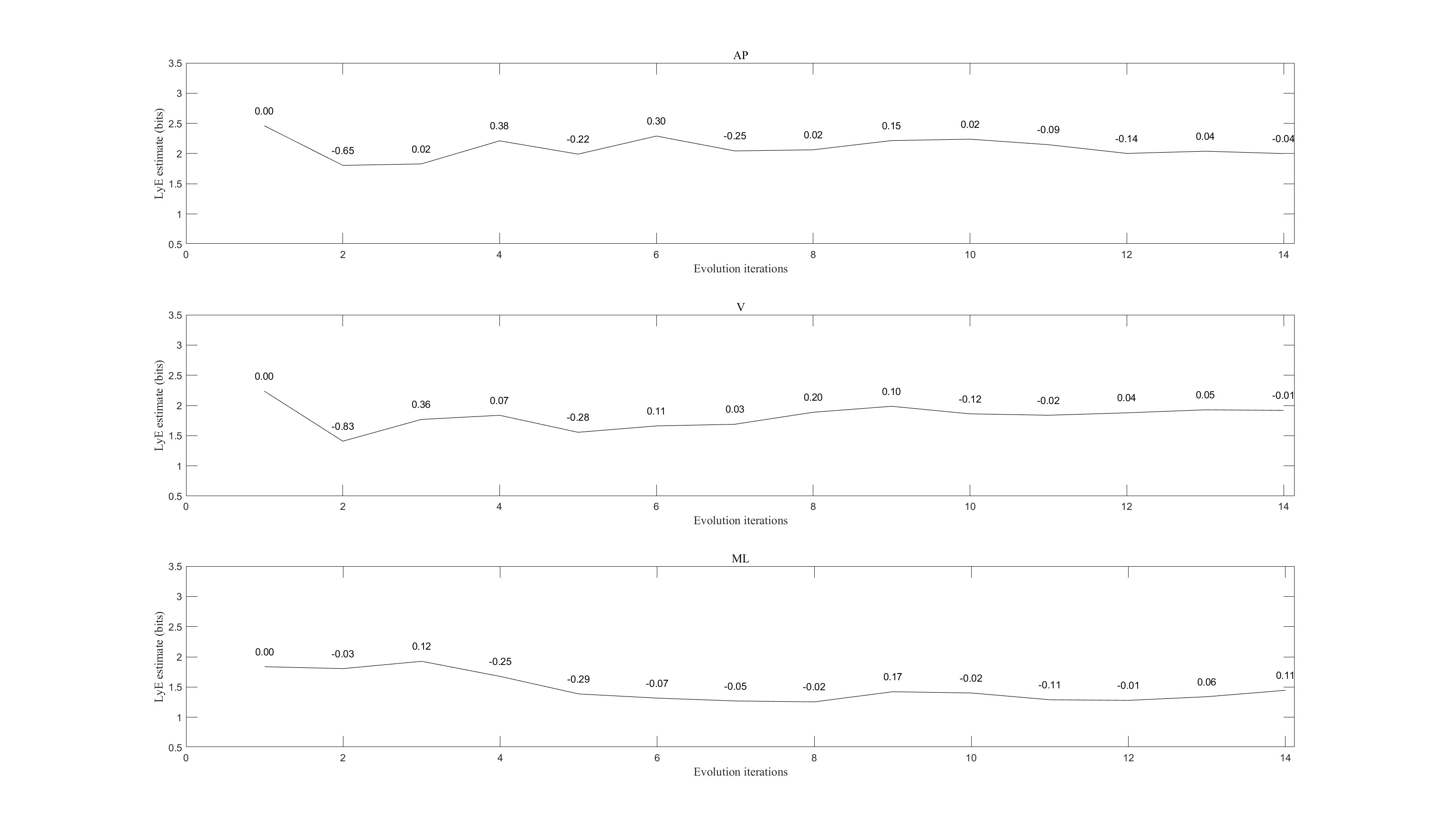

Supplement: Supplementary file 2 — Supplementary Information. [file 41598_2020_79584_MOESM2_ESM.zip › Participant1_trial8.png]

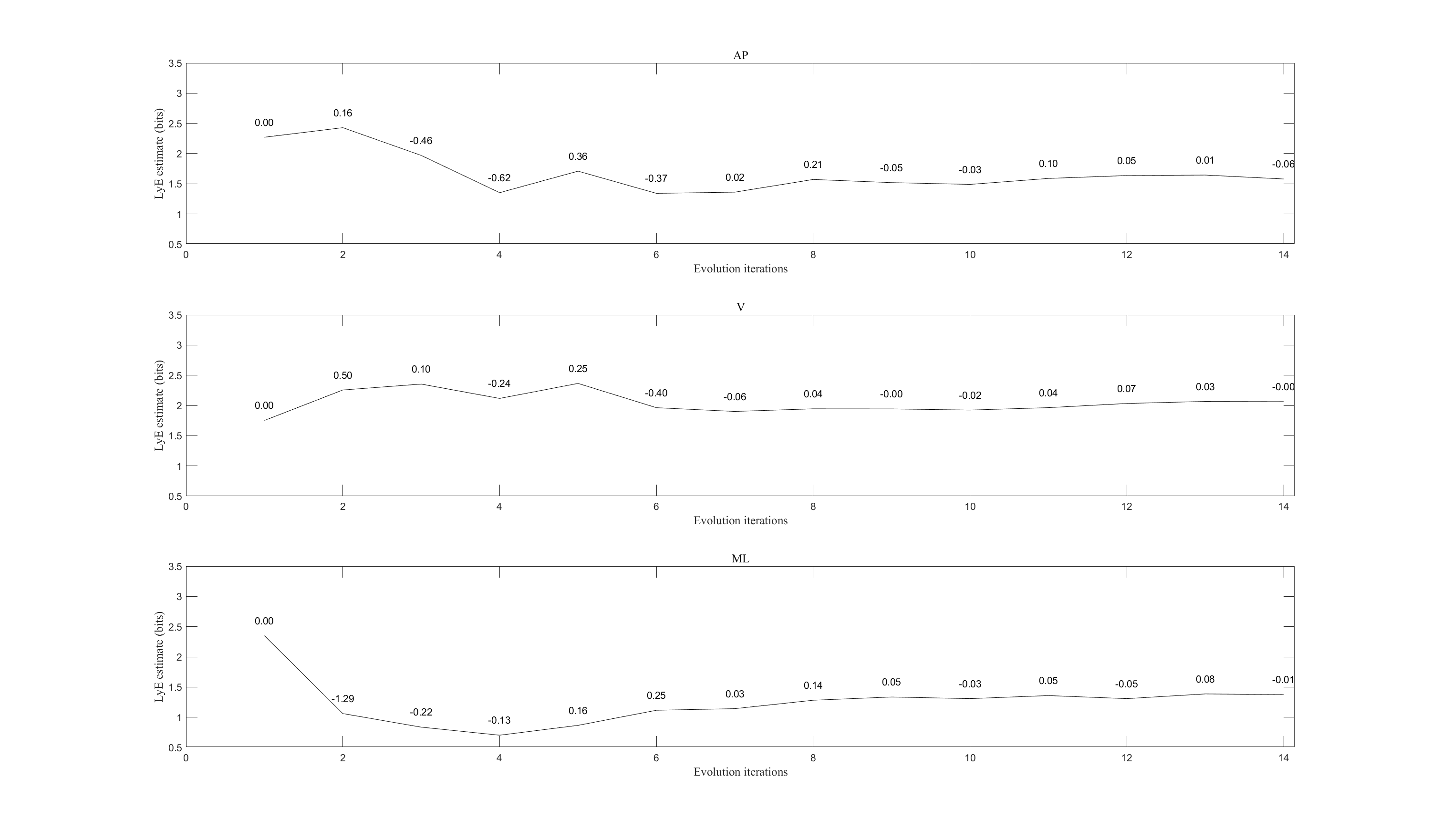

Supplement: Supplementary file 2 — Supplementary Information. [file 41598_2020_79584_MOESM2_ESM.zip › Participant1_trial9.png]

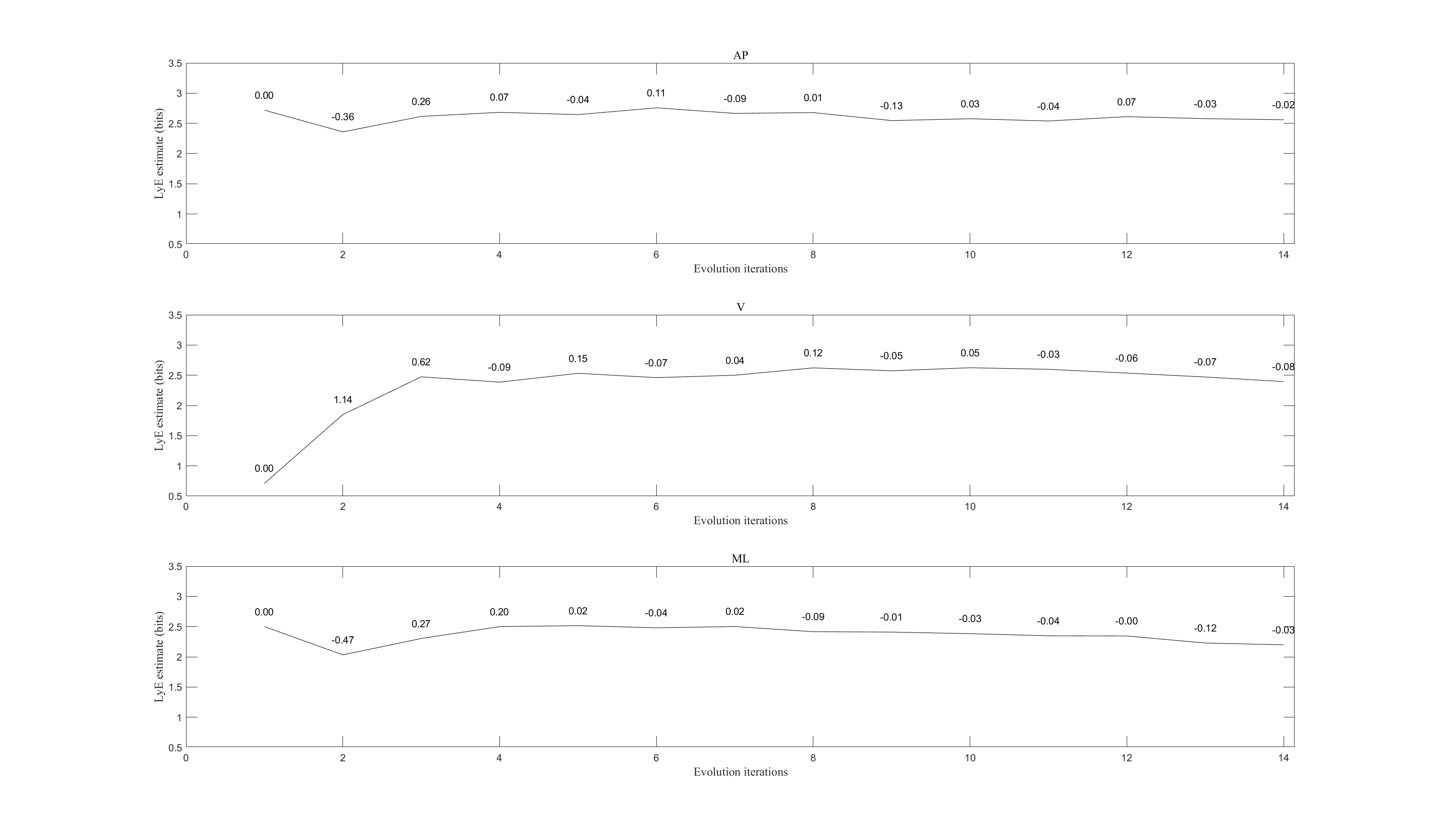

Supplement: Supplementary file 2 — Supplementary Information. [file 41598_2020_79584_MOESM2_ESM.zip › Participant10_trial1.png]

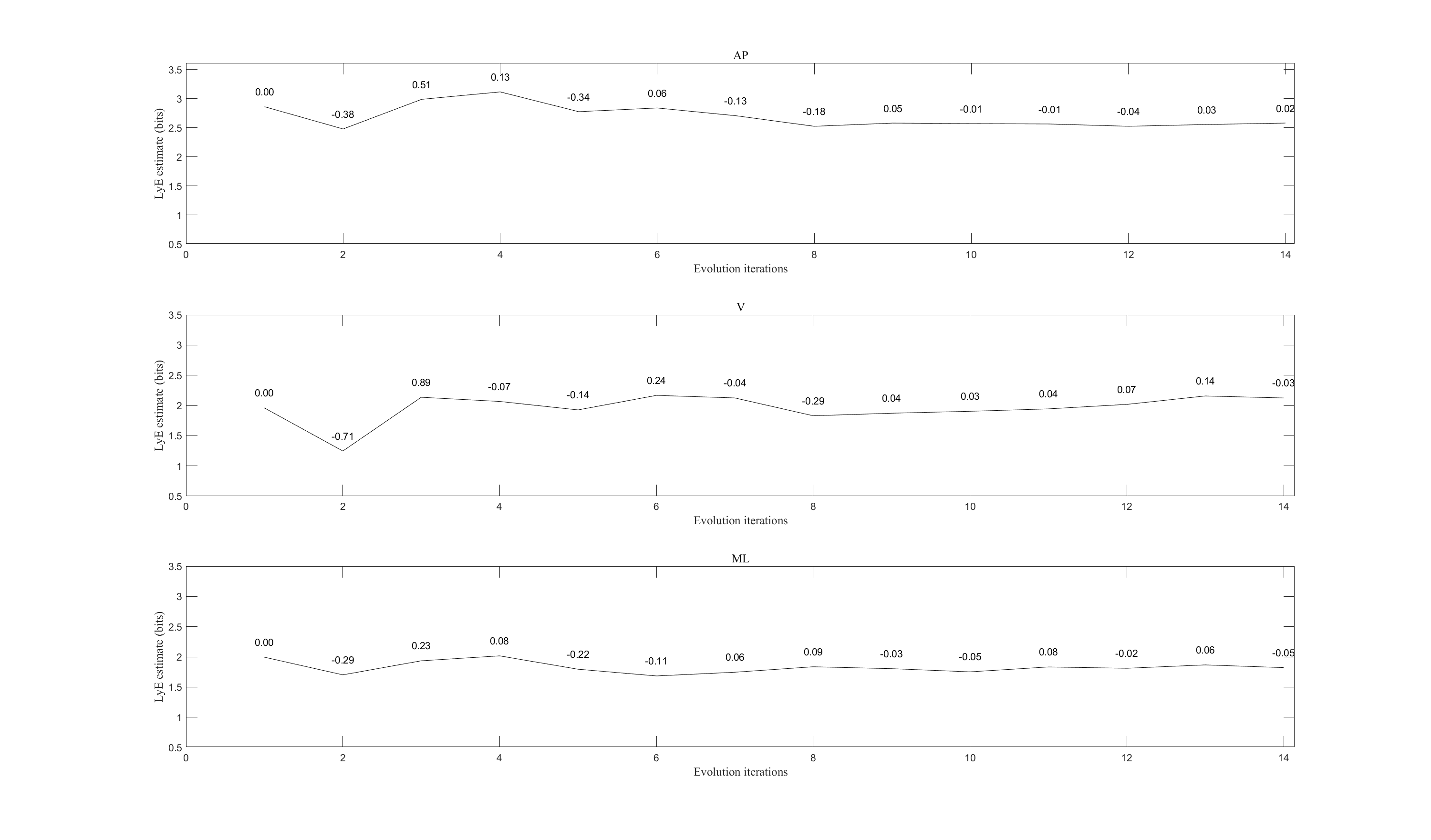

Supplement: Supplementary file 2 — Supplementary Information. [file 41598_2020_79584_MOESM2_ESM.zip › Participant10_trial10.png]

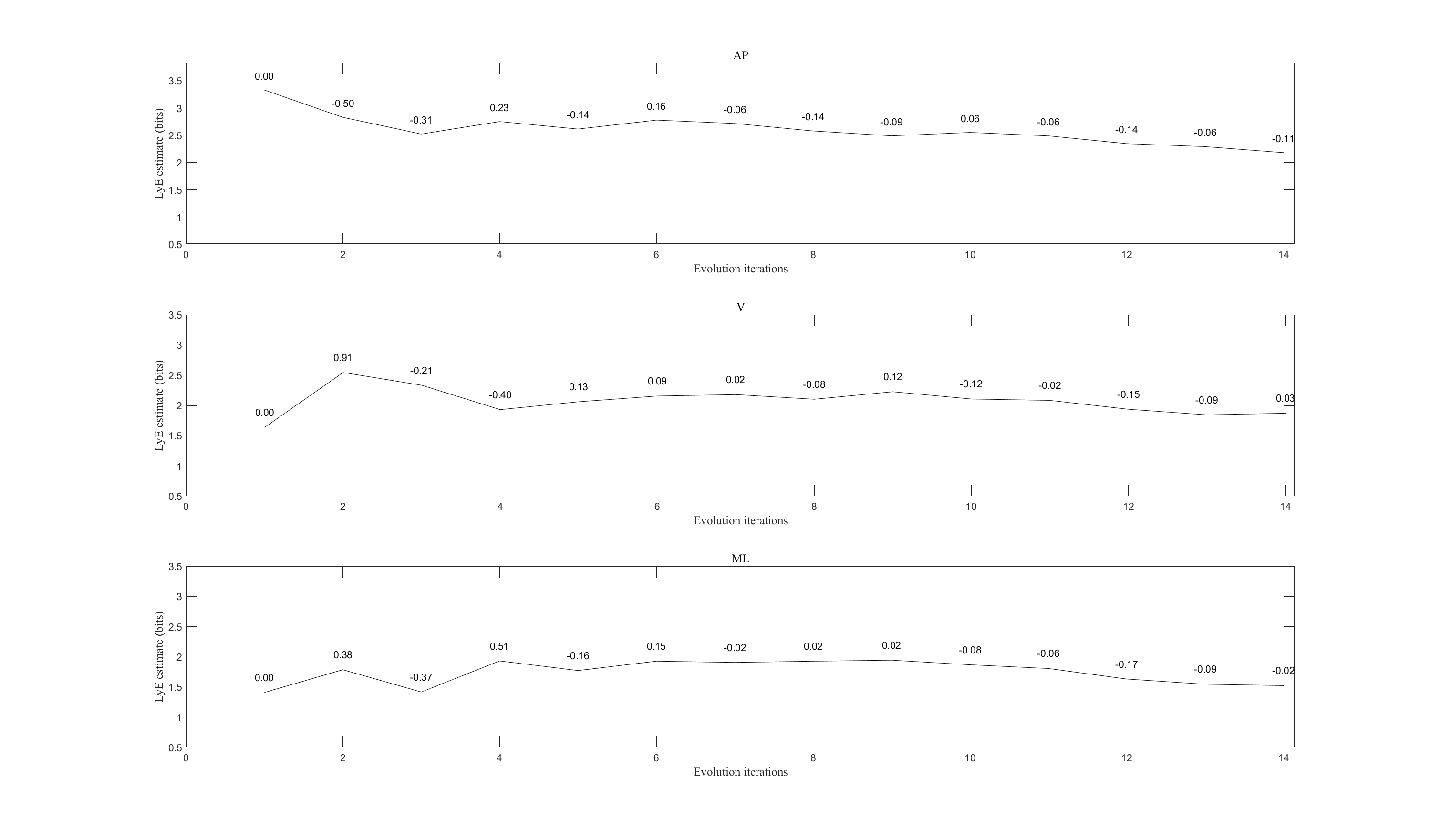

Supplement: Supplementary file 2 — Supplementary Information. [file 41598_2020_79584_MOESM2_ESM.zip › Participant10_trial11.png]

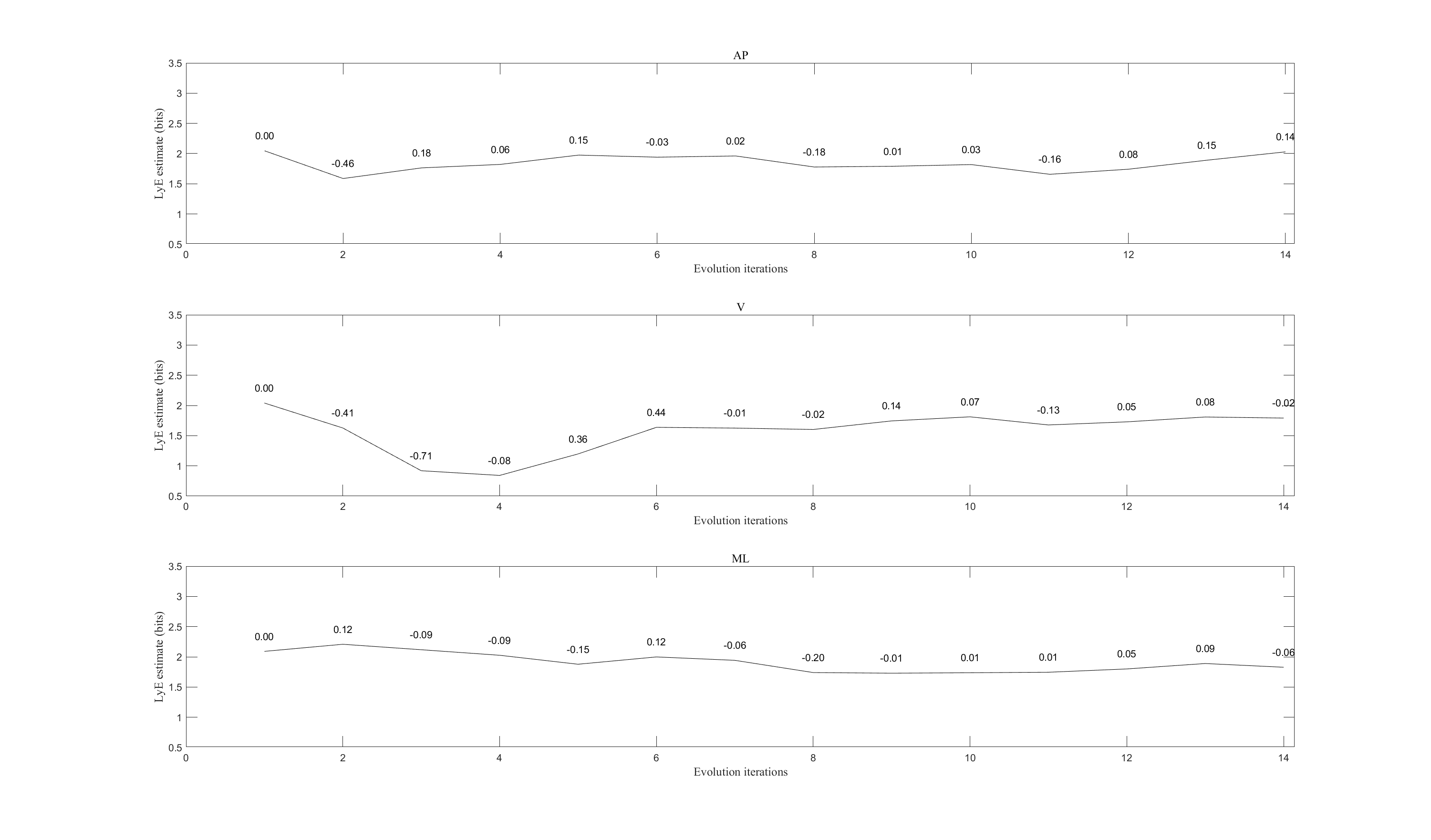

Supplement: Supplementary file 2 — Supplementary Information. [file 41598_2020_79584_MOESM2_ESM.zip › Participant10_trial12.png]

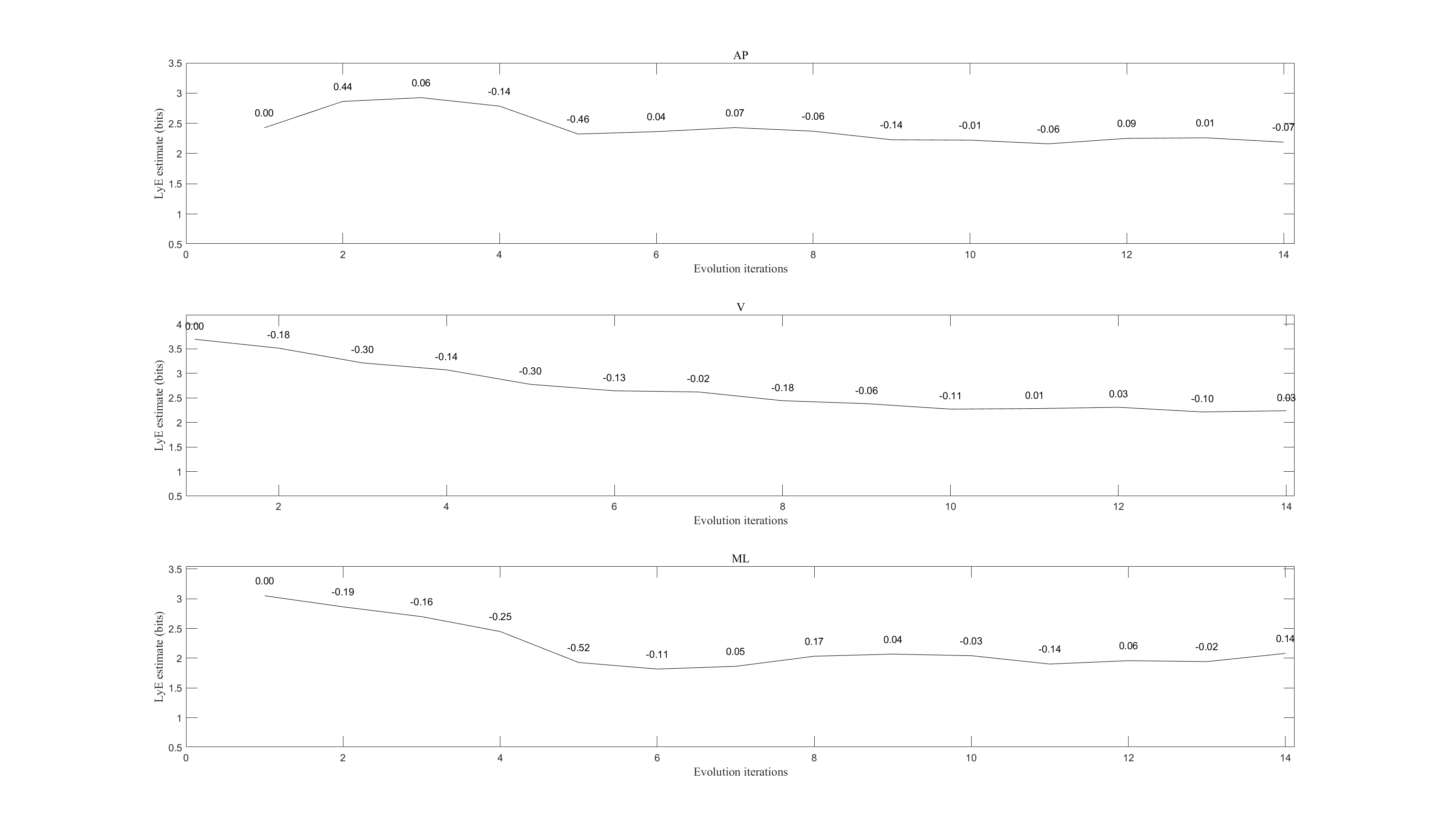

Supplement: Supplementary file 2 — Supplementary Information. [file 41598_2020_79584_MOESM2_ESM.zip › Participant10_trial2.png]

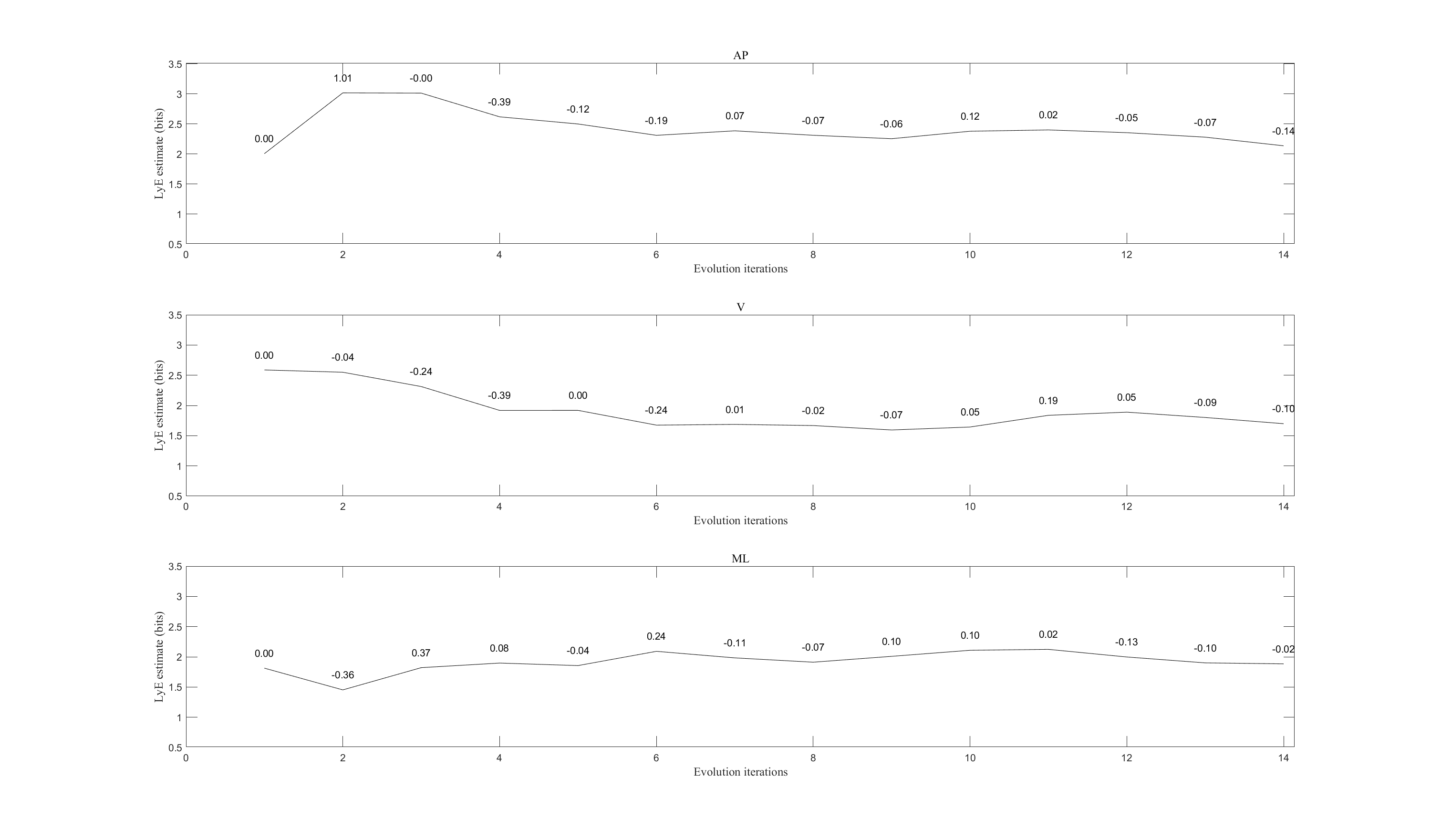

Supplement: Supplementary file 2 — Supplementary Information. [file 41598_2020_79584_MOESM2_ESM.zip › Participant10_trial3.png]

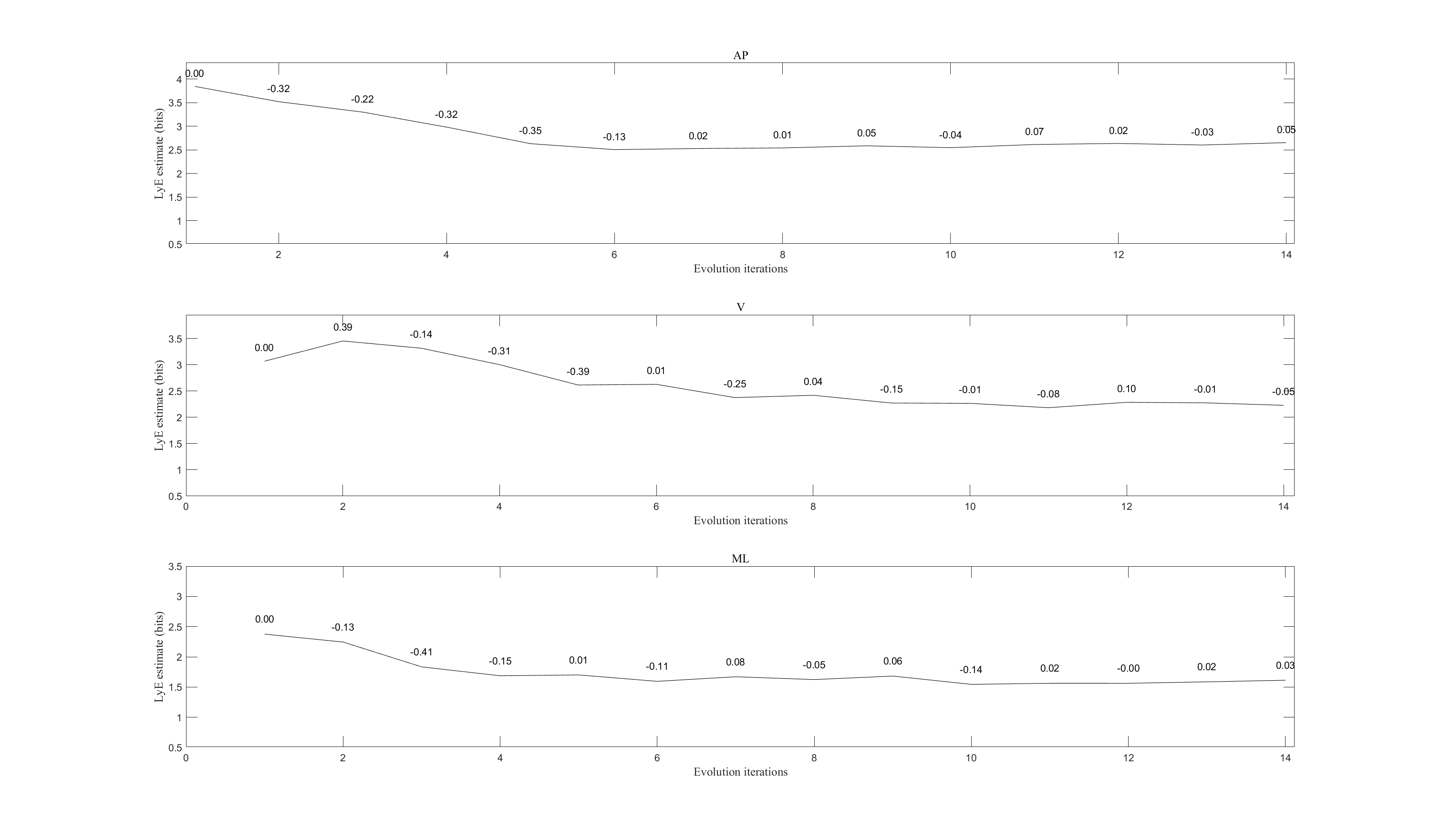

Supplement: Supplementary file 2 — Supplementary Information. [file 41598_2020_79584_MOESM2_ESM.zip › Participant10_trial4.png]

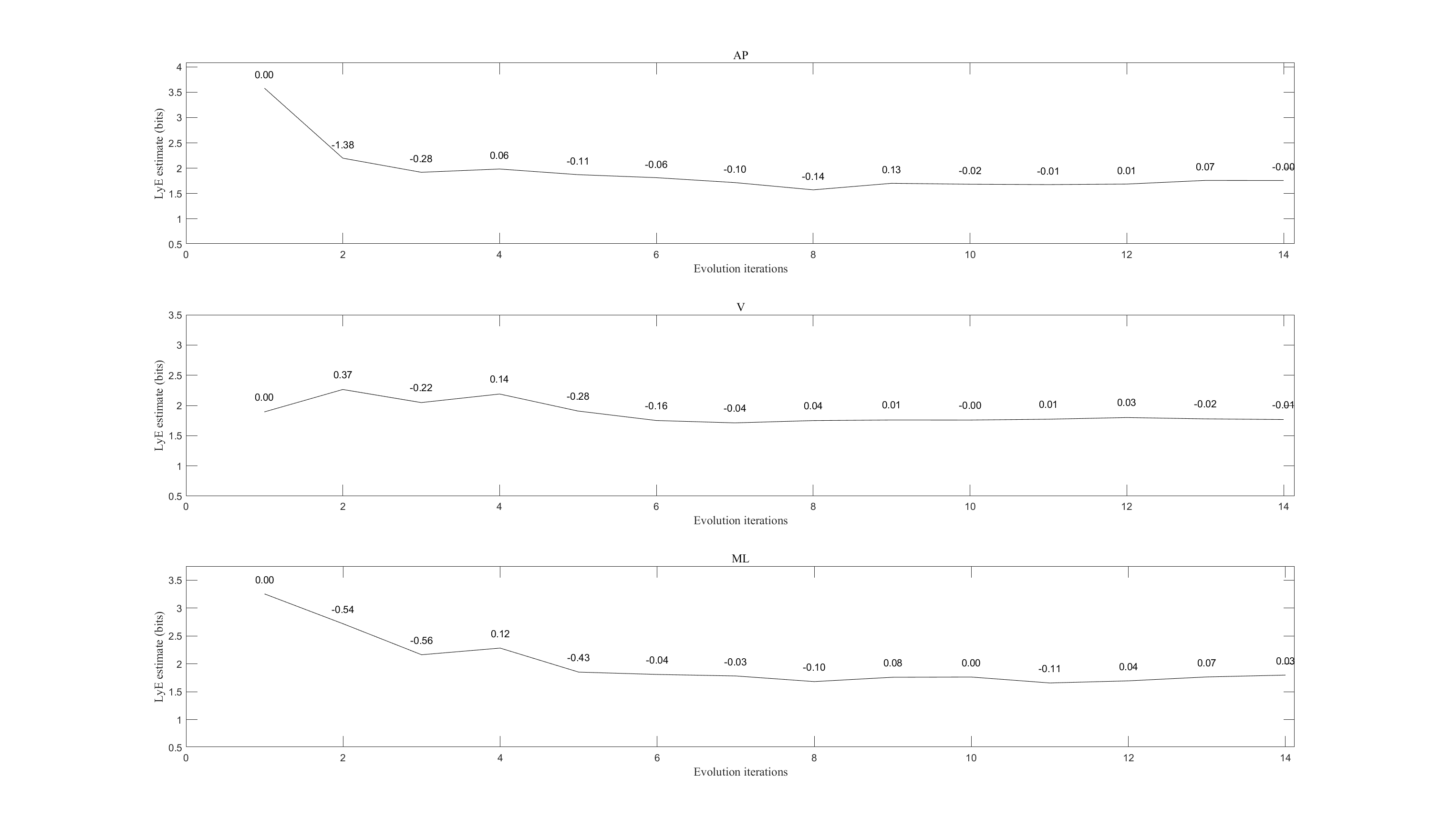

Supplement: Supplementary file 2 — Supplementary Information. [file 41598_2020_79584_MOESM2_ESM.zip › Participant10_trial5.png]

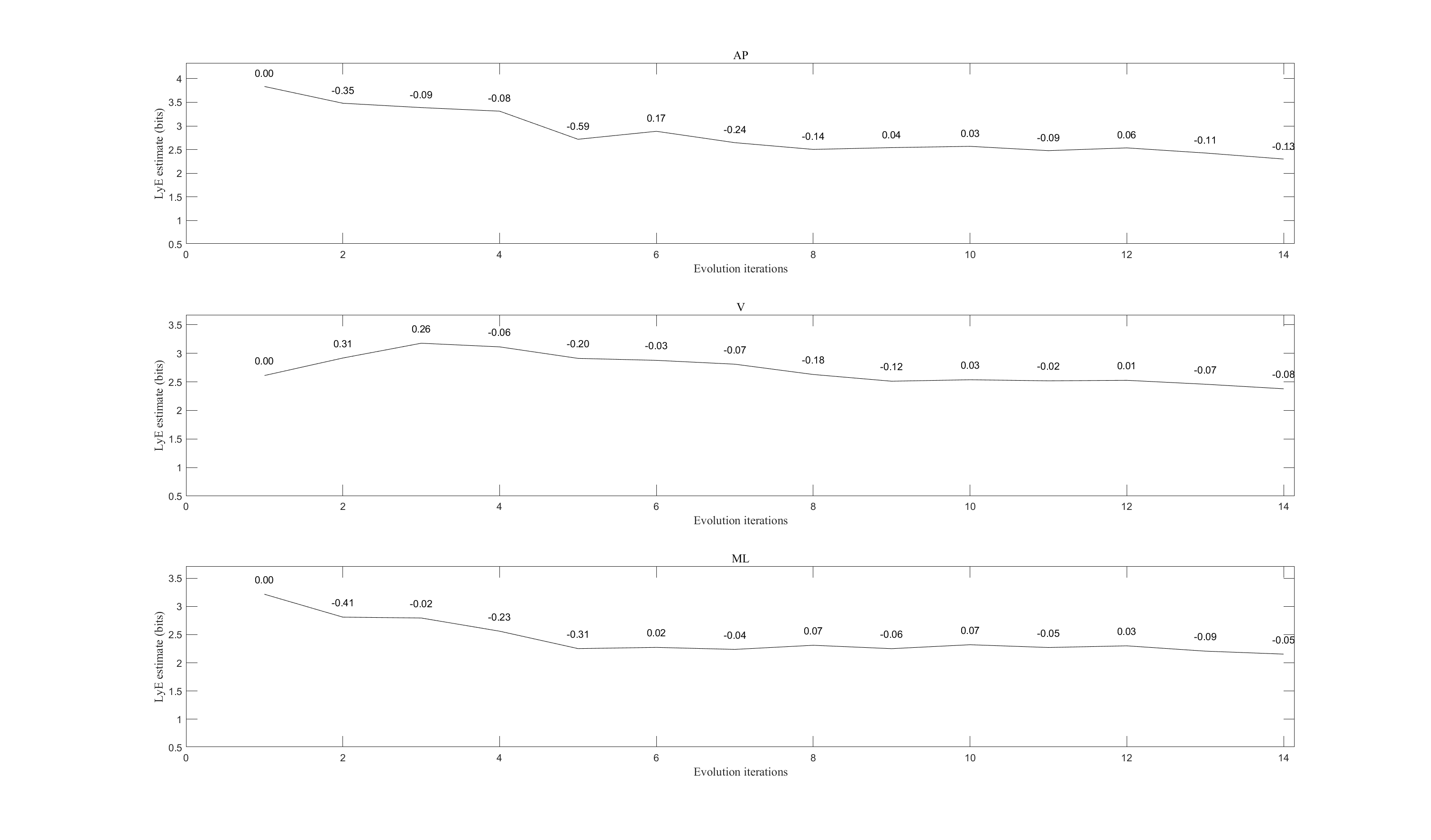

Supplement: Supplementary file 2 — Supplementary Information. [file 41598_2020_79584_MOESM2_ESM.zip › Participant10_trial6.png]

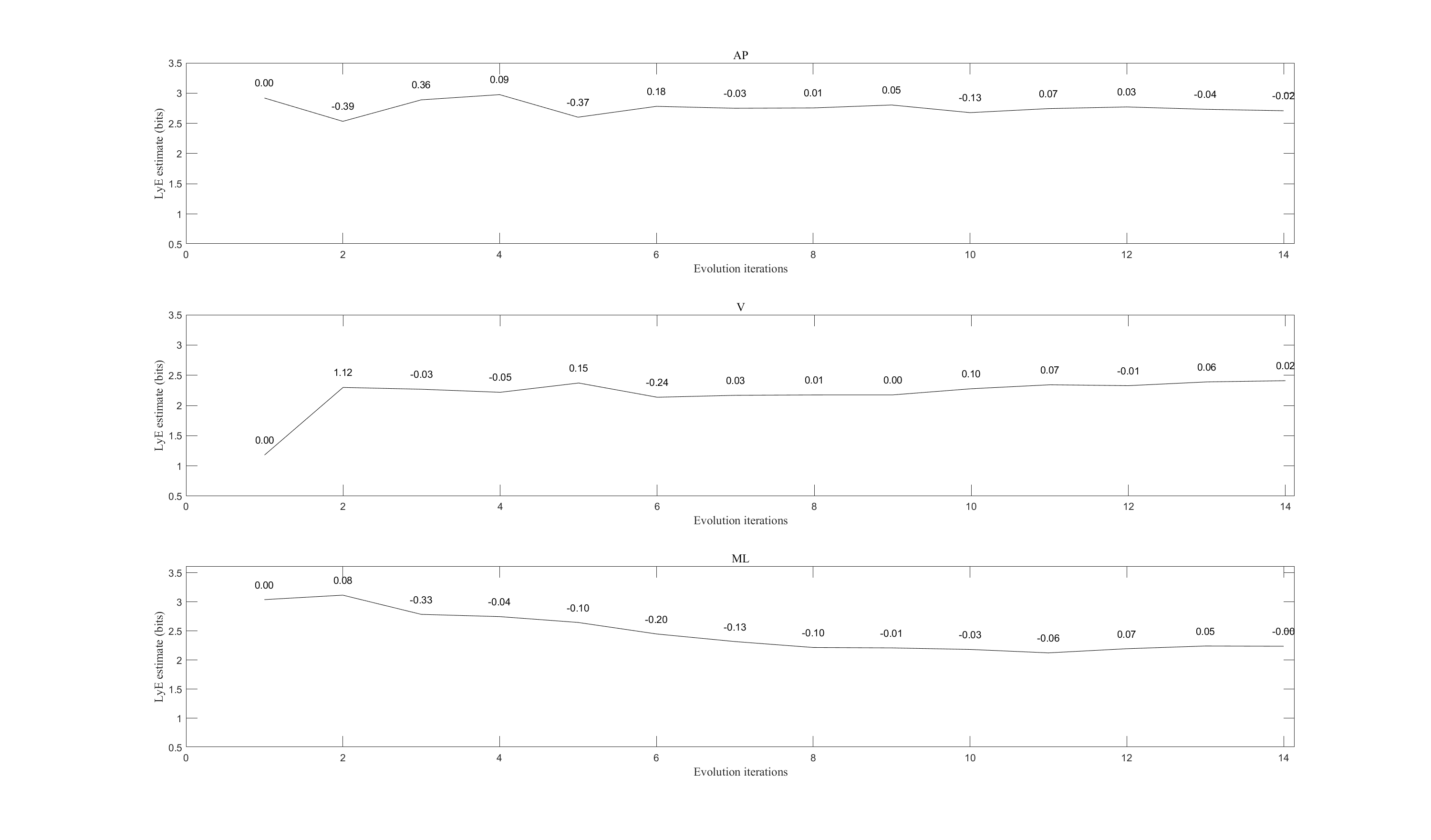

Supplement: Supplementary file 2 — Supplementary Information. [file 41598_2020_79584_MOESM2_ESM.zip › Participant10_trial7.png]

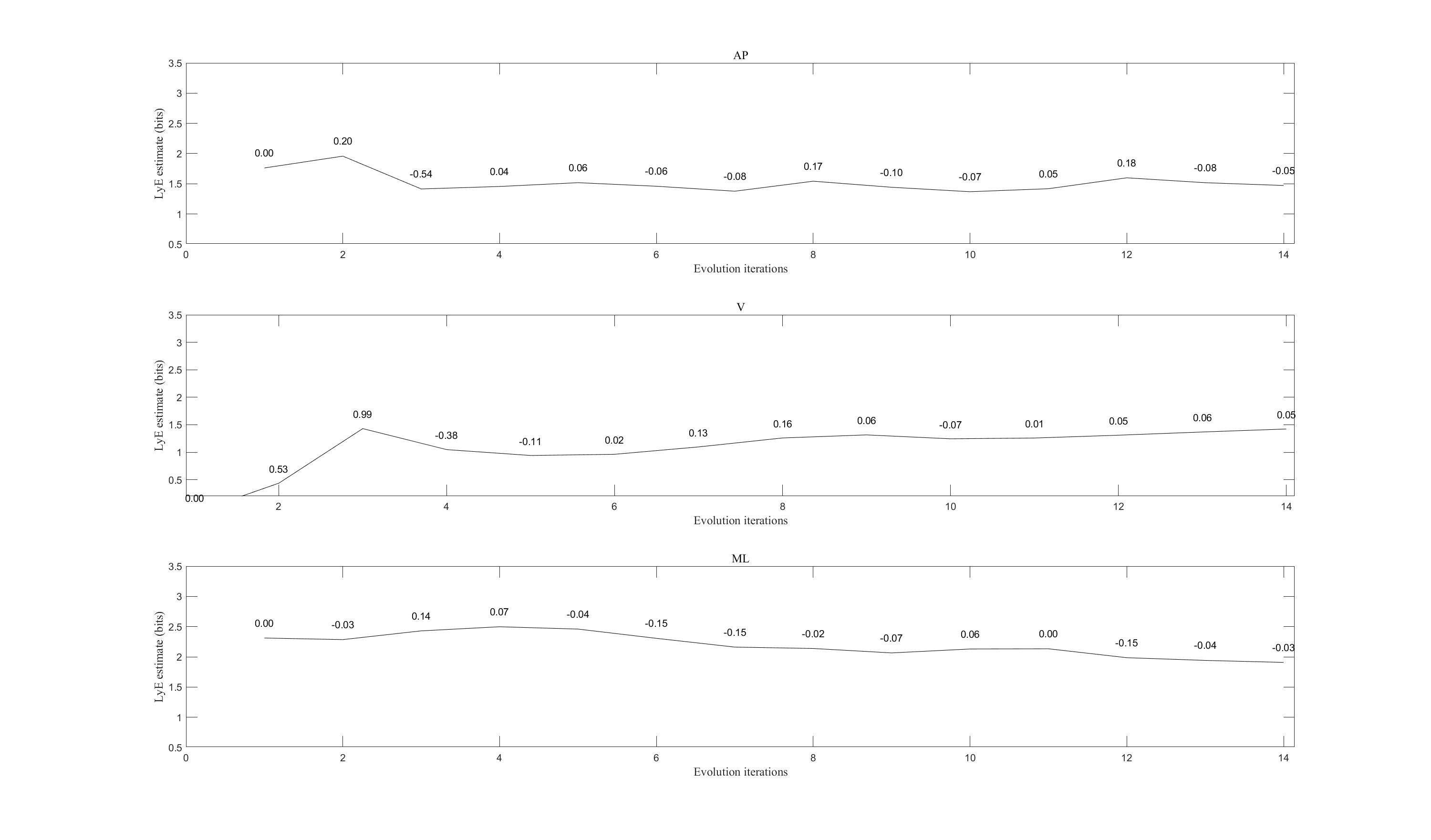

Supplement: Supplementary file 2 — Supplementary Information. [file 41598_2020_79584_MOESM2_ESM.zip › Participant10_trial8.png]

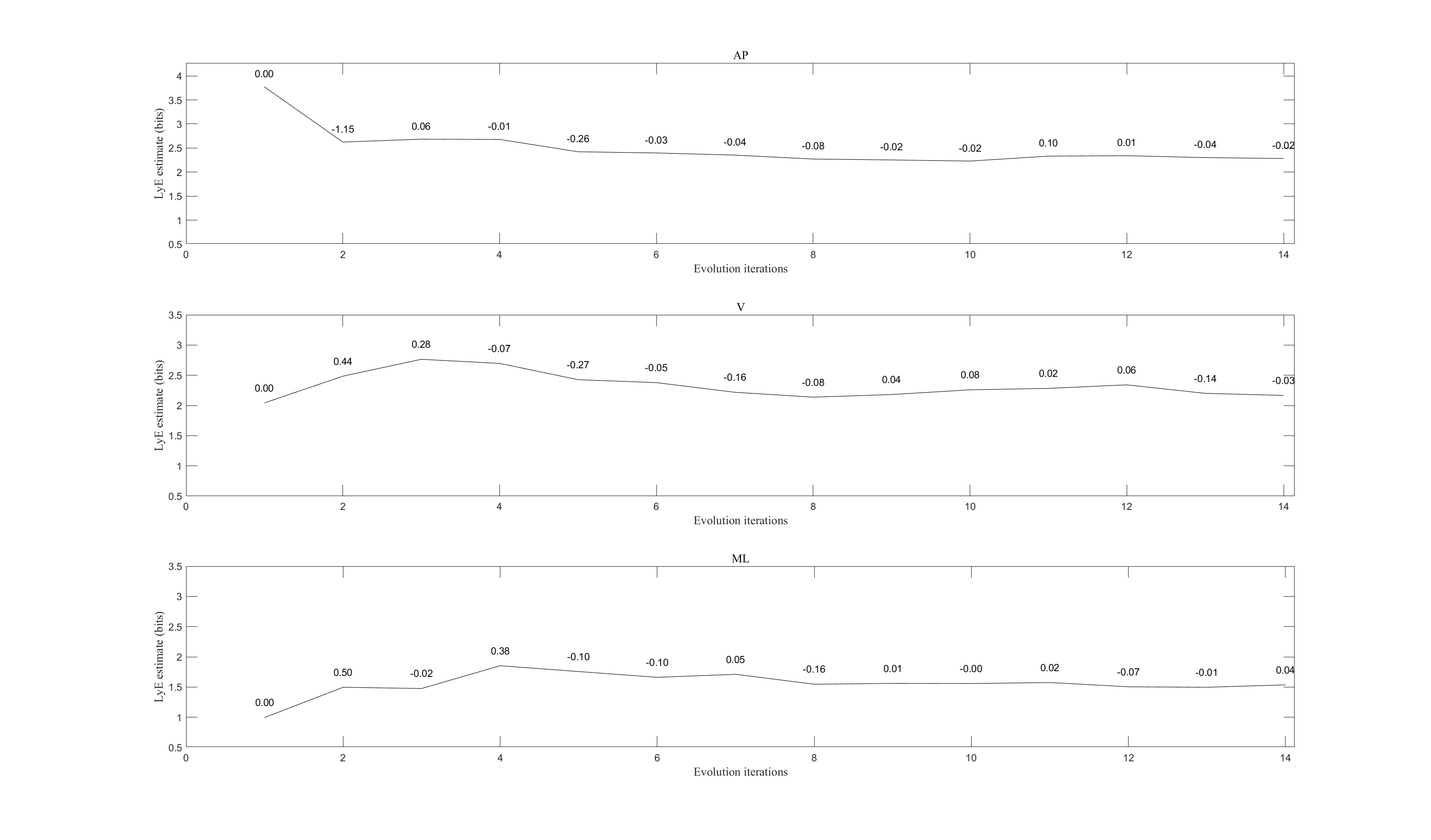

Supplement: Supplementary file 2 — Supplementary Information. [file 41598_2020_79584_MOESM2_ESM.zip › Participant10_trial9.png]

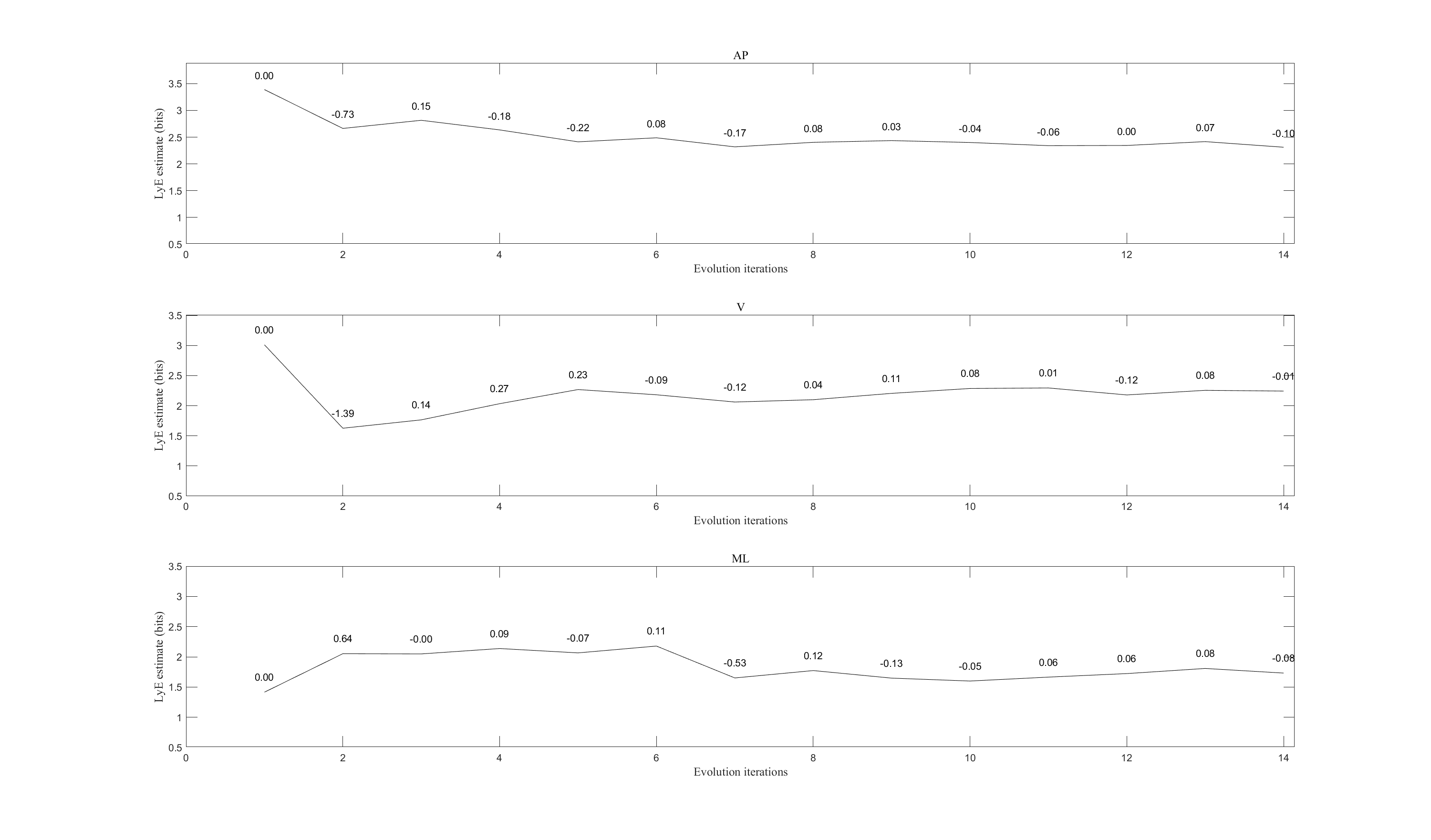

Supplement: Supplementary file 2 — Supplementary Information. [file 41598_2020_79584_MOESM2_ESM.zip › Participant11_trial1.png]

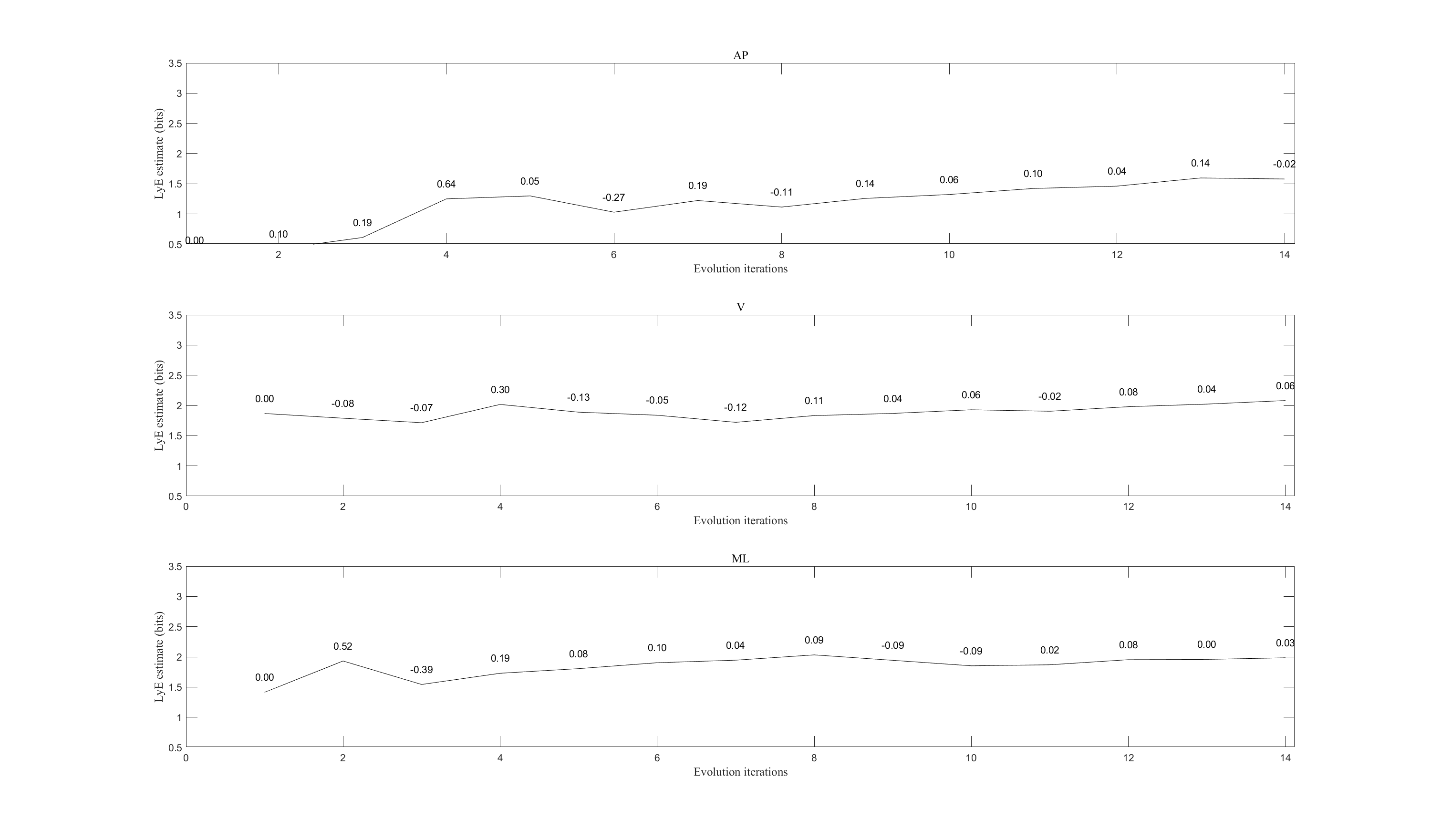

Supplement: Supplementary file 2 — Supplementary Information. [file 41598_2020_79584_MOESM2_ESM.zip › Participant11_trial10.png]

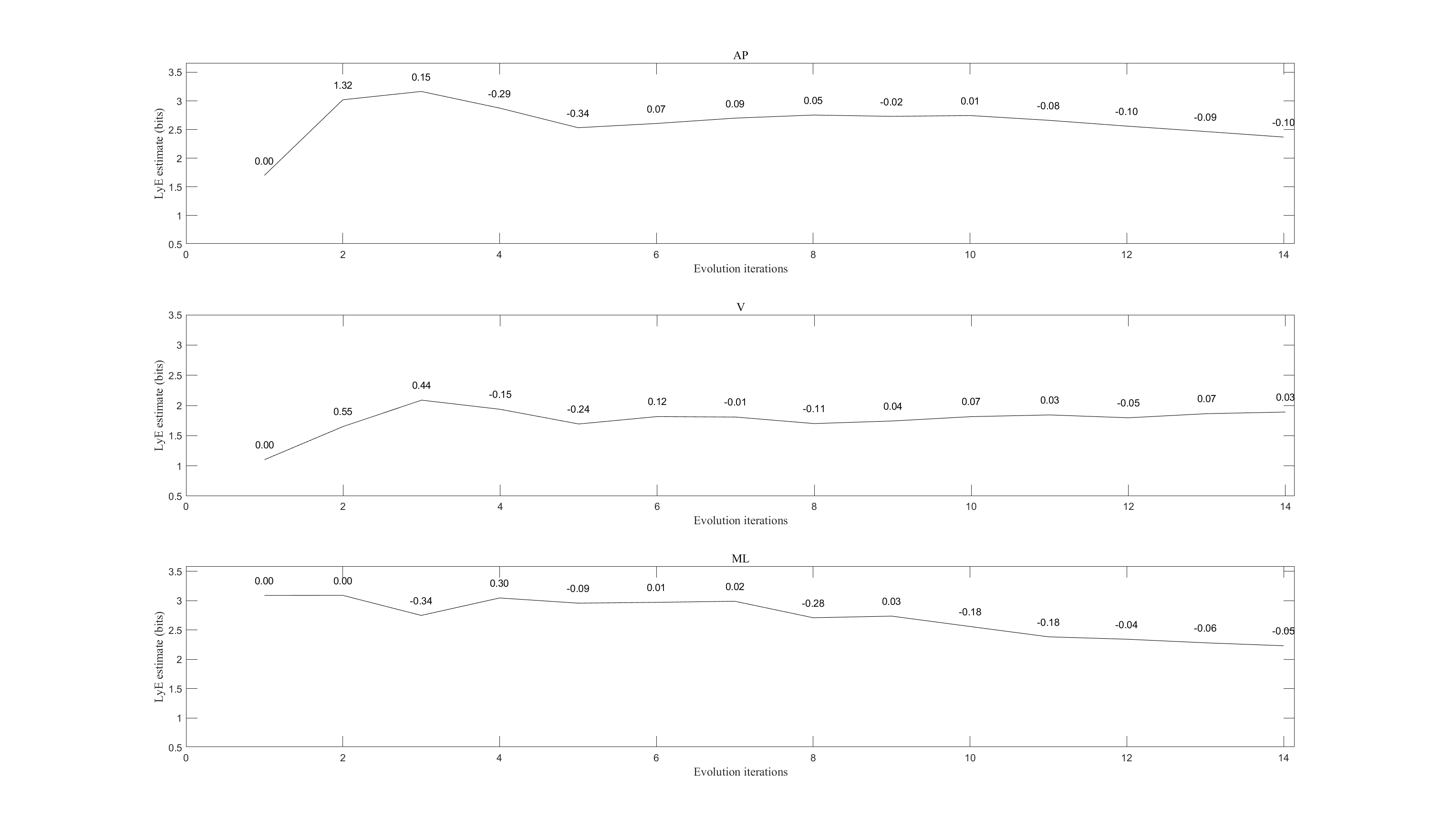

Supplement: Supplementary file 2 — Supplementary Information. [file 41598_2020_79584_MOESM2_ESM.zip › Participant11_trial11.png]

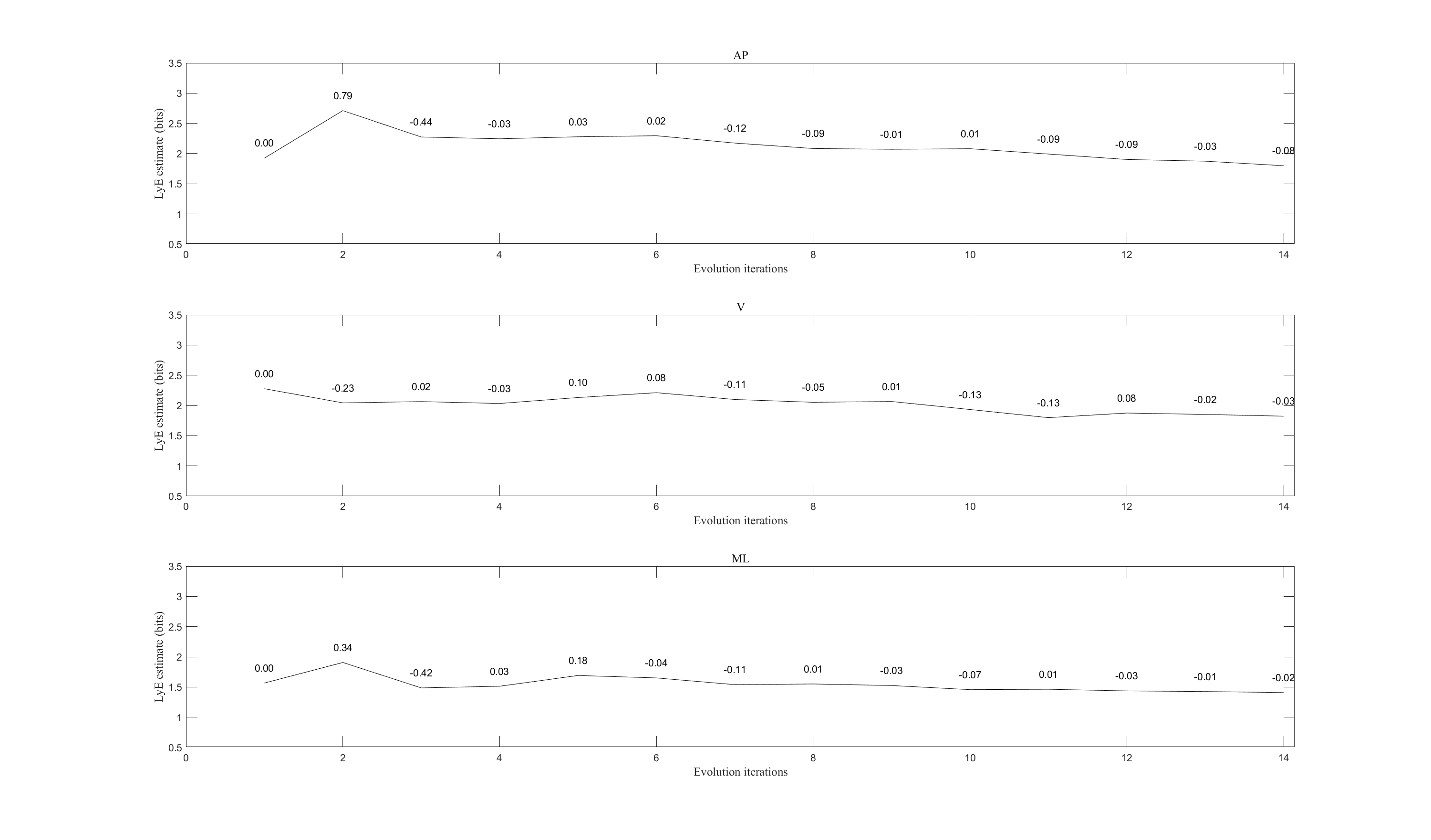

Supplement: Supplementary file 2 — Supplementary Information. [file 41598_2020_79584_MOESM2_ESM.zip › Participant11_trial12.png]

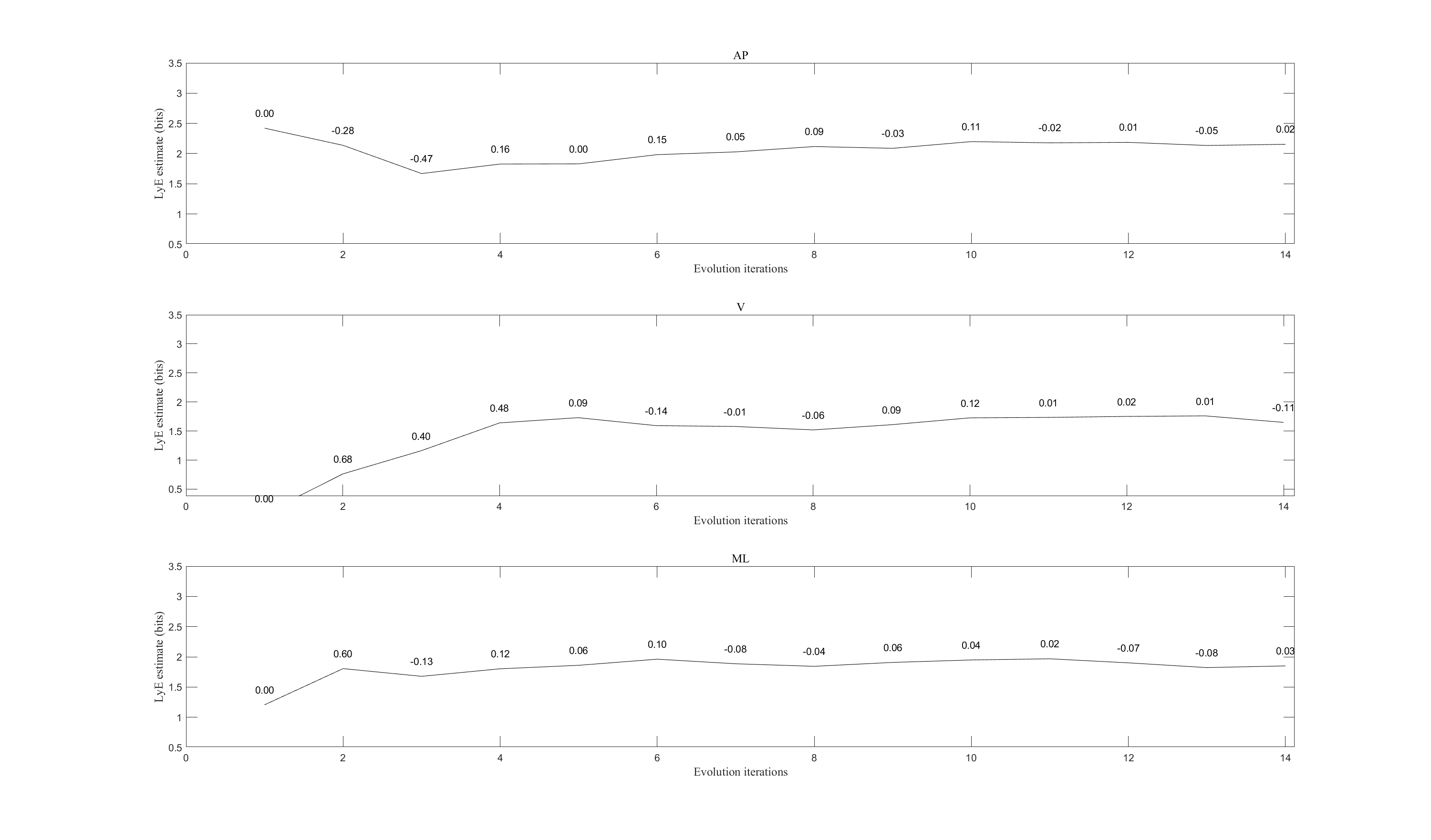

Supplement: Supplementary file 2 — Supplementary Information. [file 41598_2020_79584_MOESM2_ESM.zip › Participant11_trial2.png]

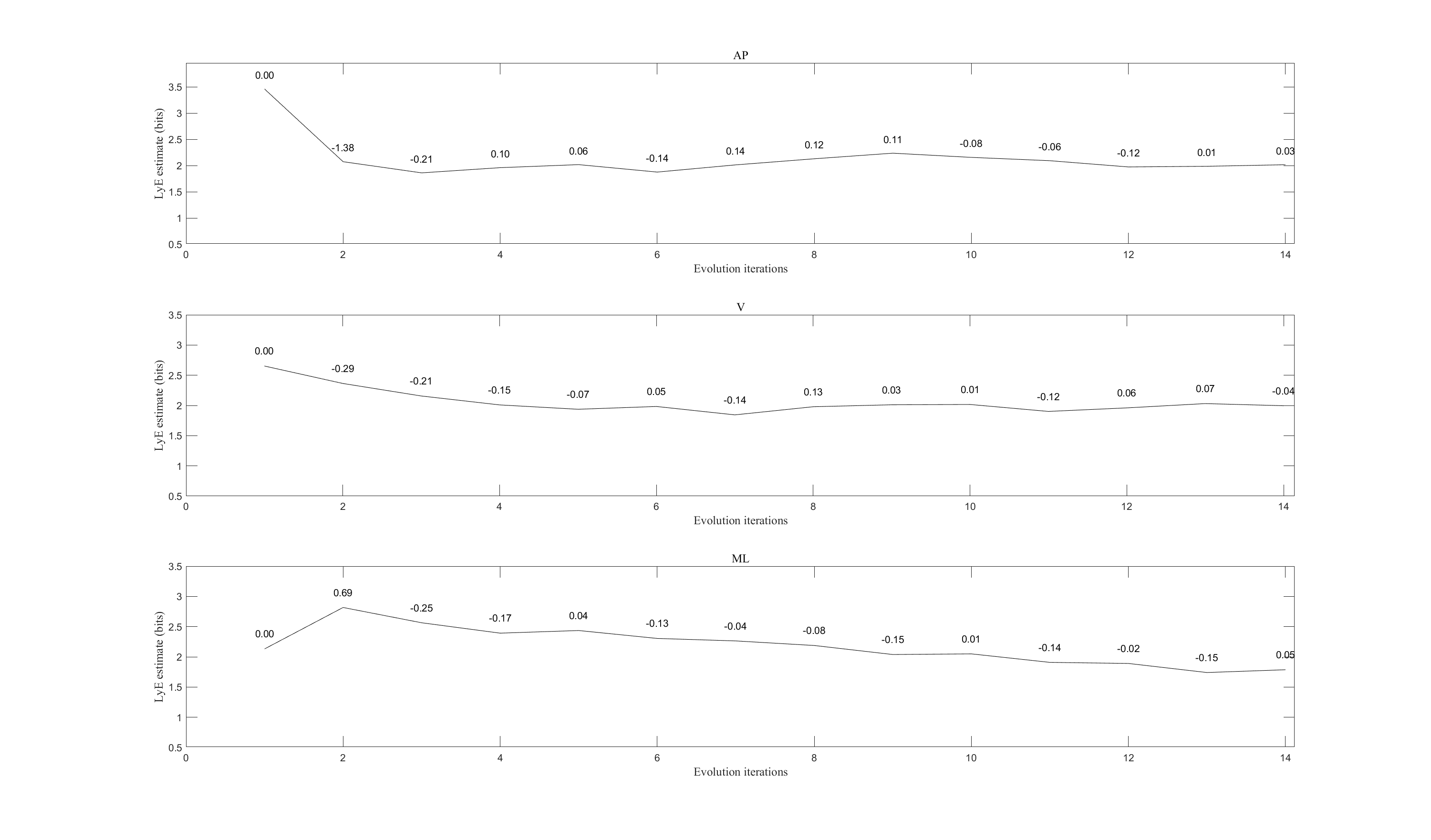

Supplement: Supplementary file 2 — Supplementary Information. [file 41598_2020_79584_MOESM2_ESM.zip › Participant11_trial3.png]

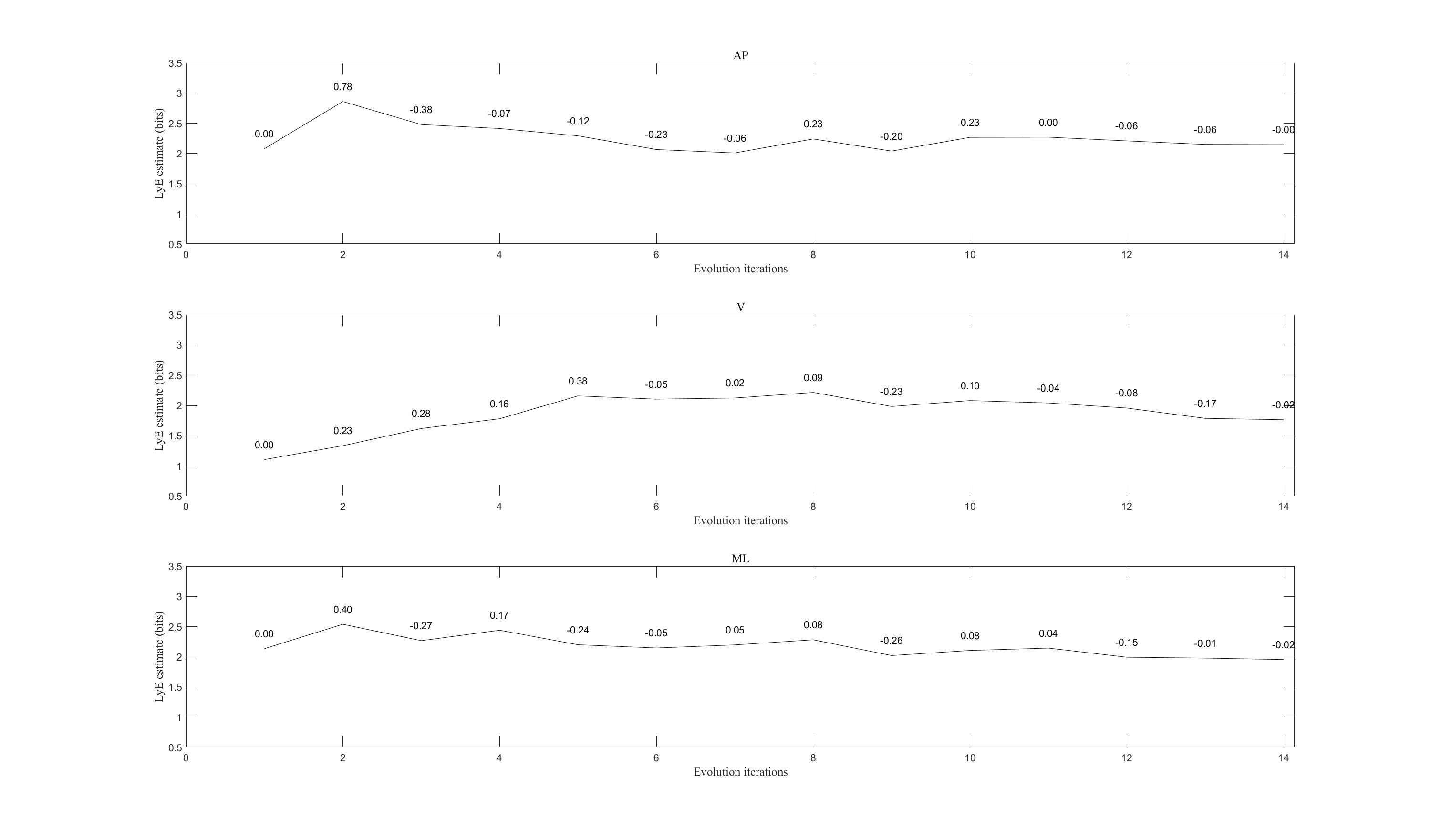

Supplement: Supplementary file 2 — Supplementary Information. [file 41598_2020_79584_MOESM2_ESM.zip › Participant11_trial4.png]

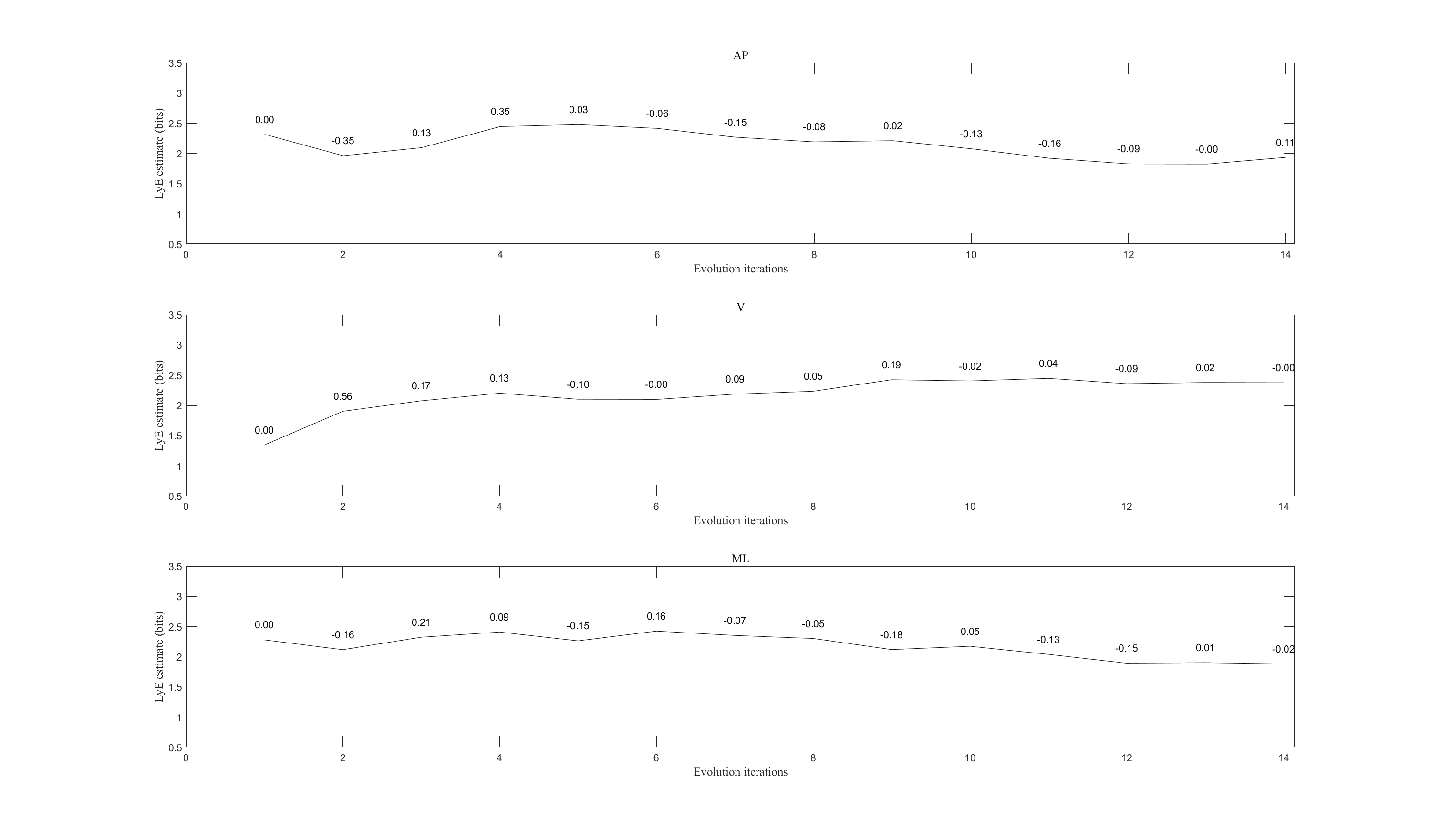

Supplement: Supplementary file 2 — Supplementary Information. [file 41598_2020_79584_MOESM2_ESM.zip › Participant11_trial5.png]

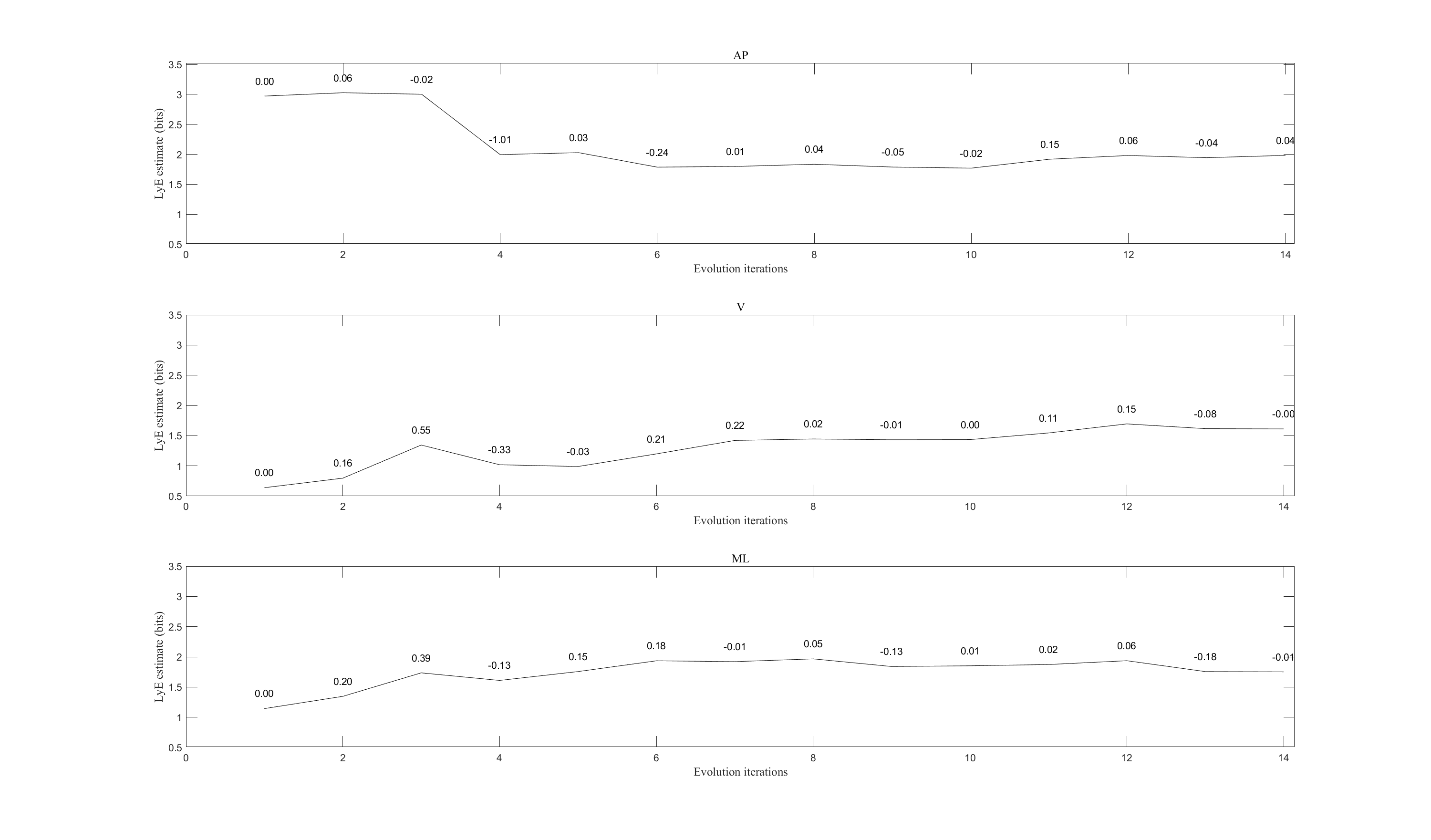

Supplement: Supplementary file 2 — Supplementary Information. [file 41598_2020_79584_MOESM2_ESM.zip › Participant11_trial6.png]

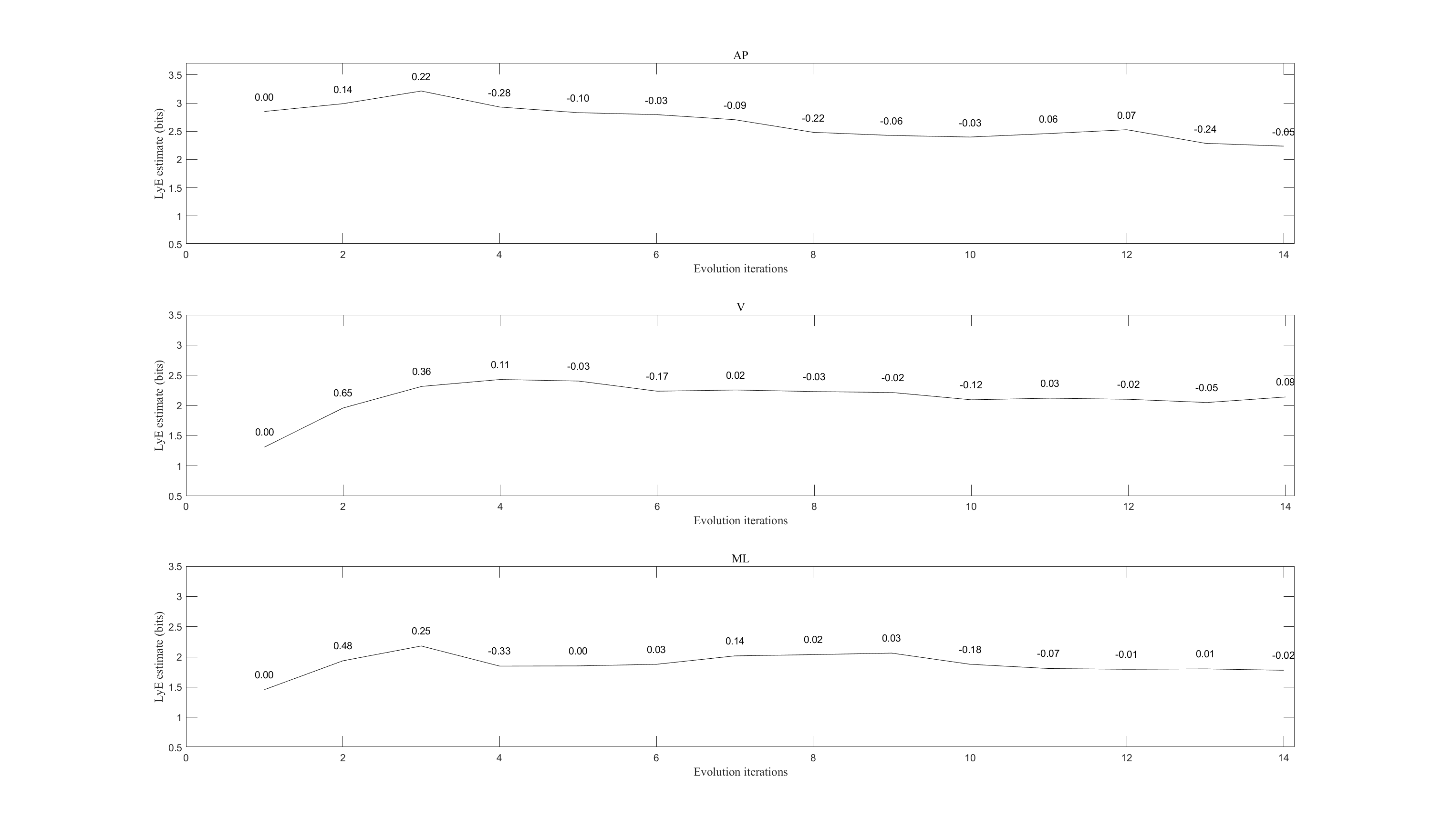

Supplement: Supplementary file 2 — Supplementary Information. [file 41598_2020_79584_MOESM2_ESM.zip › Participant11_trial7.png]

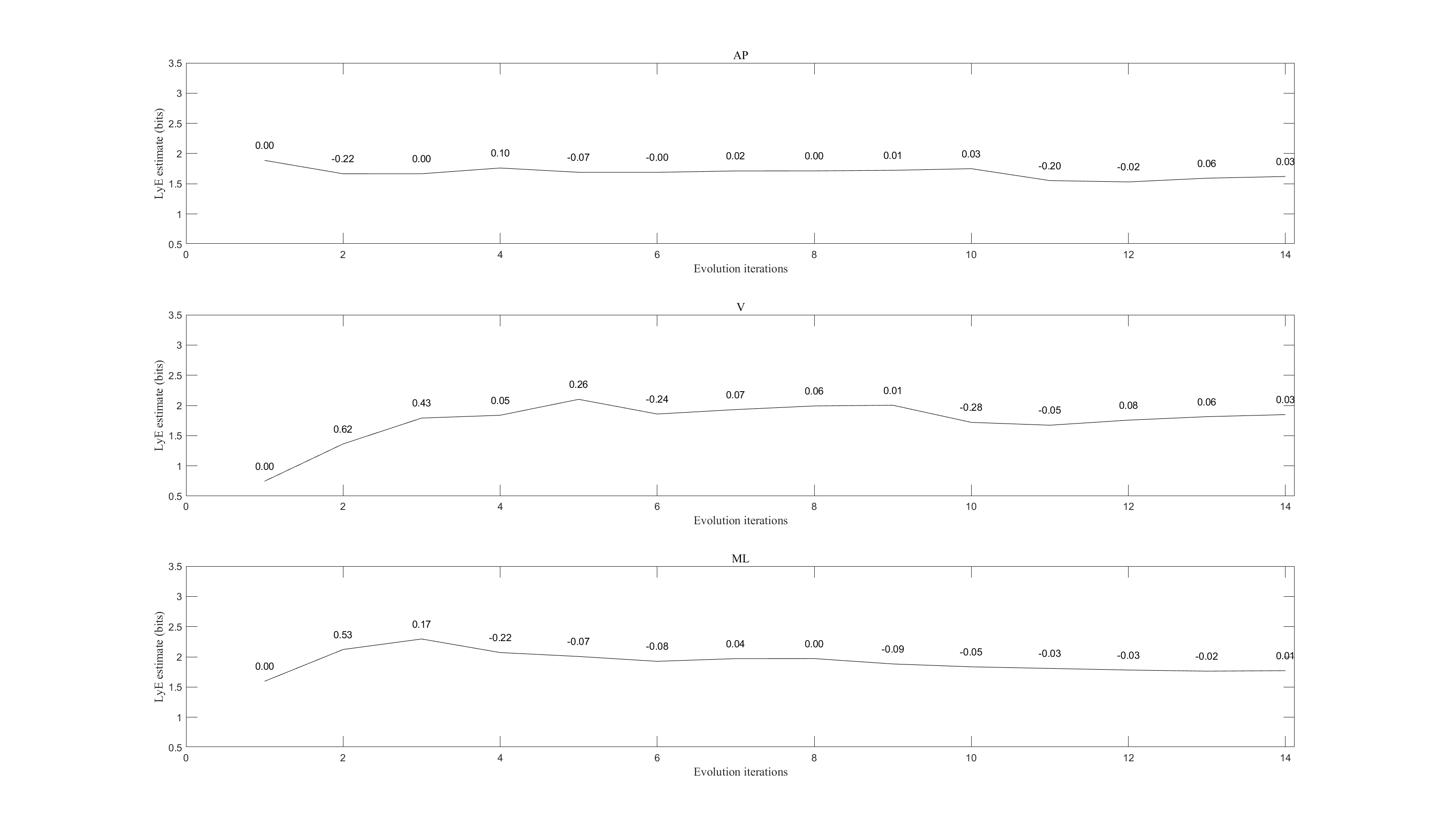

Supplement: Supplementary file 2 — Supplementary Information. [file 41598_2020_79584_MOESM2_ESM.zip › Participant11_trial8.png]

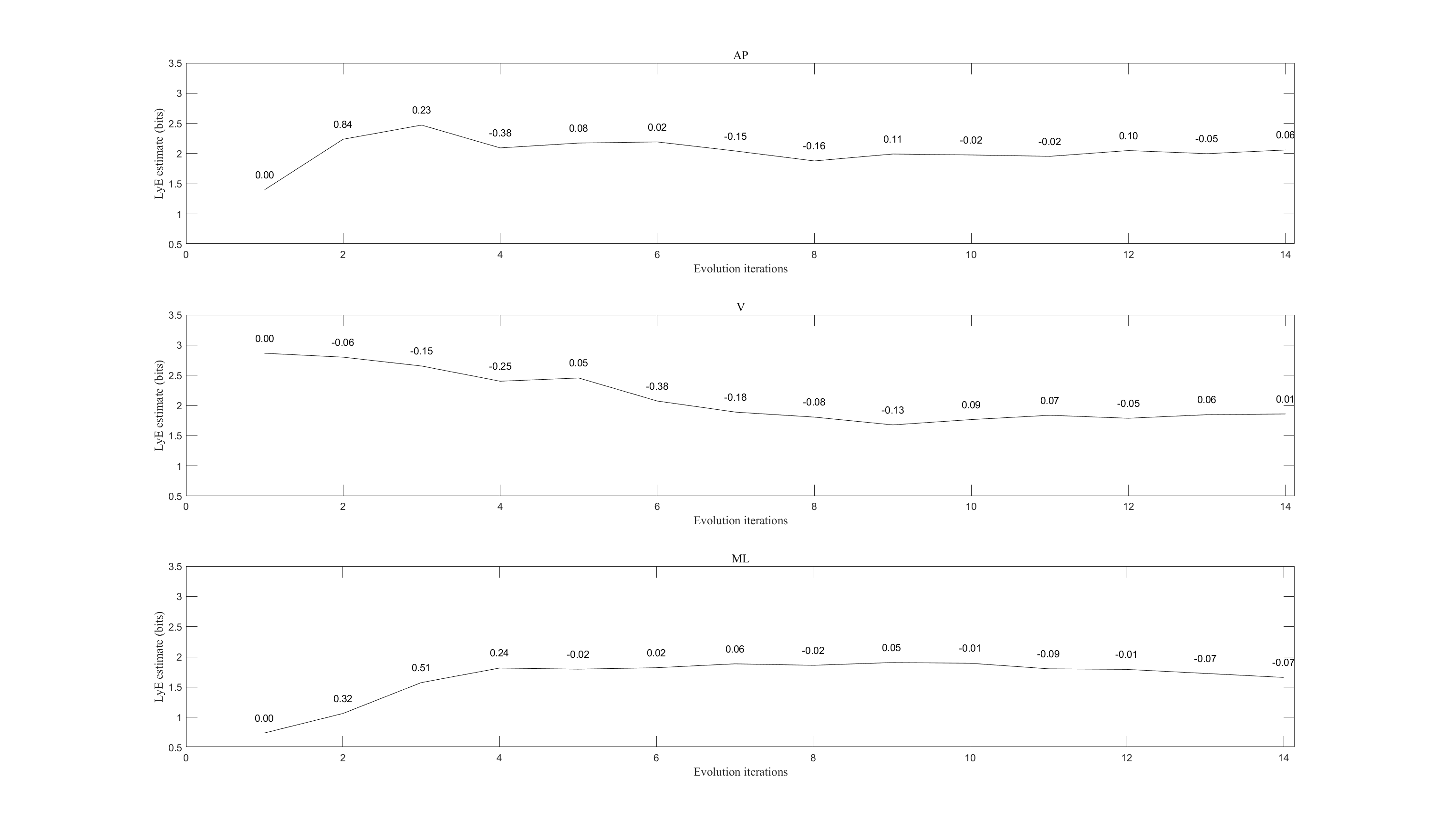

Supplement: Supplementary file 2 — Supplementary Information. [file 41598_2020_79584_MOESM2_ESM.zip › Participant11_trial9.png]

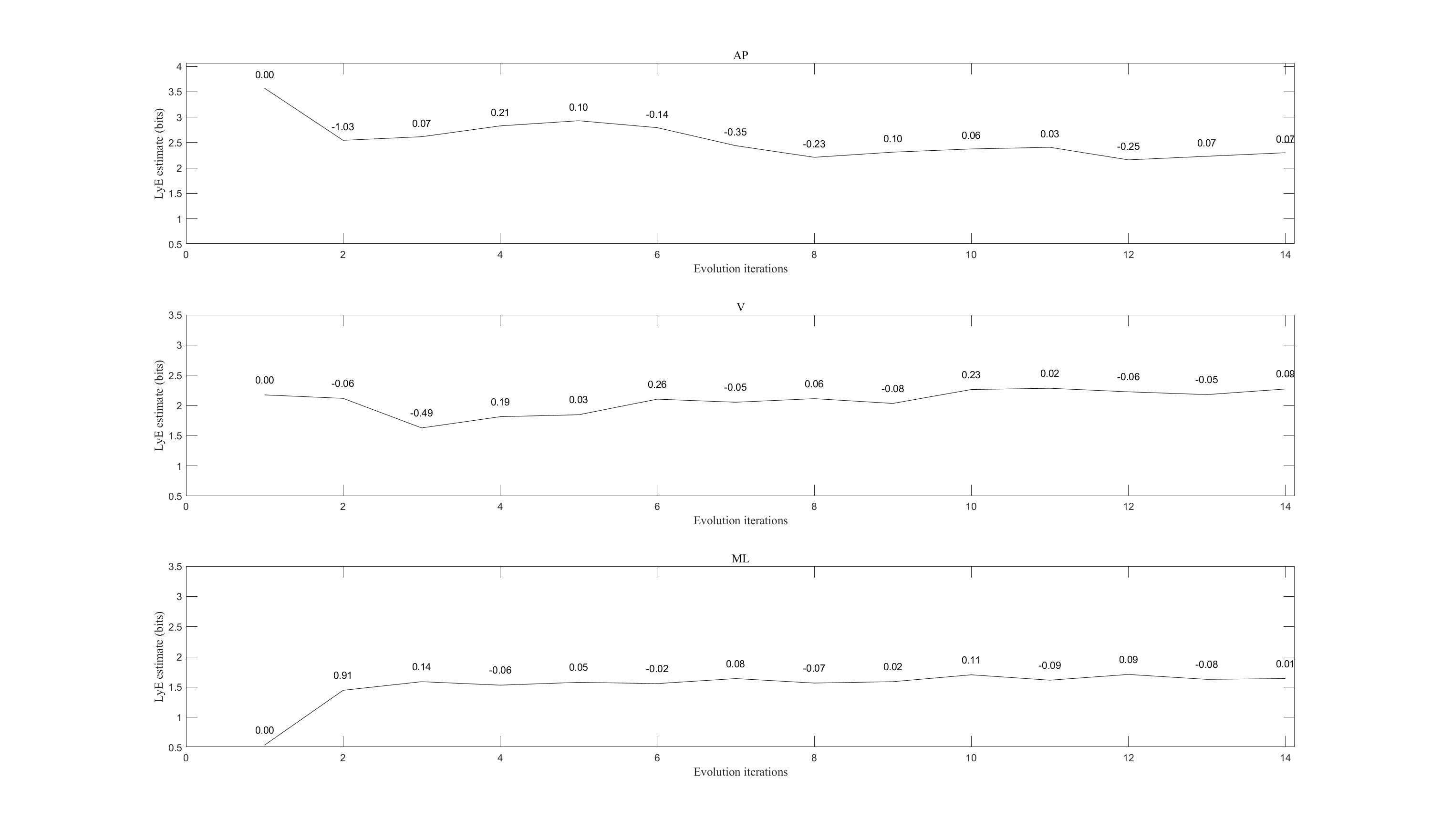

Supplement: Supplementary file 2 — Supplementary Information. [file 41598_2020_79584_MOESM2_ESM.zip › Participant12_trial1.png]

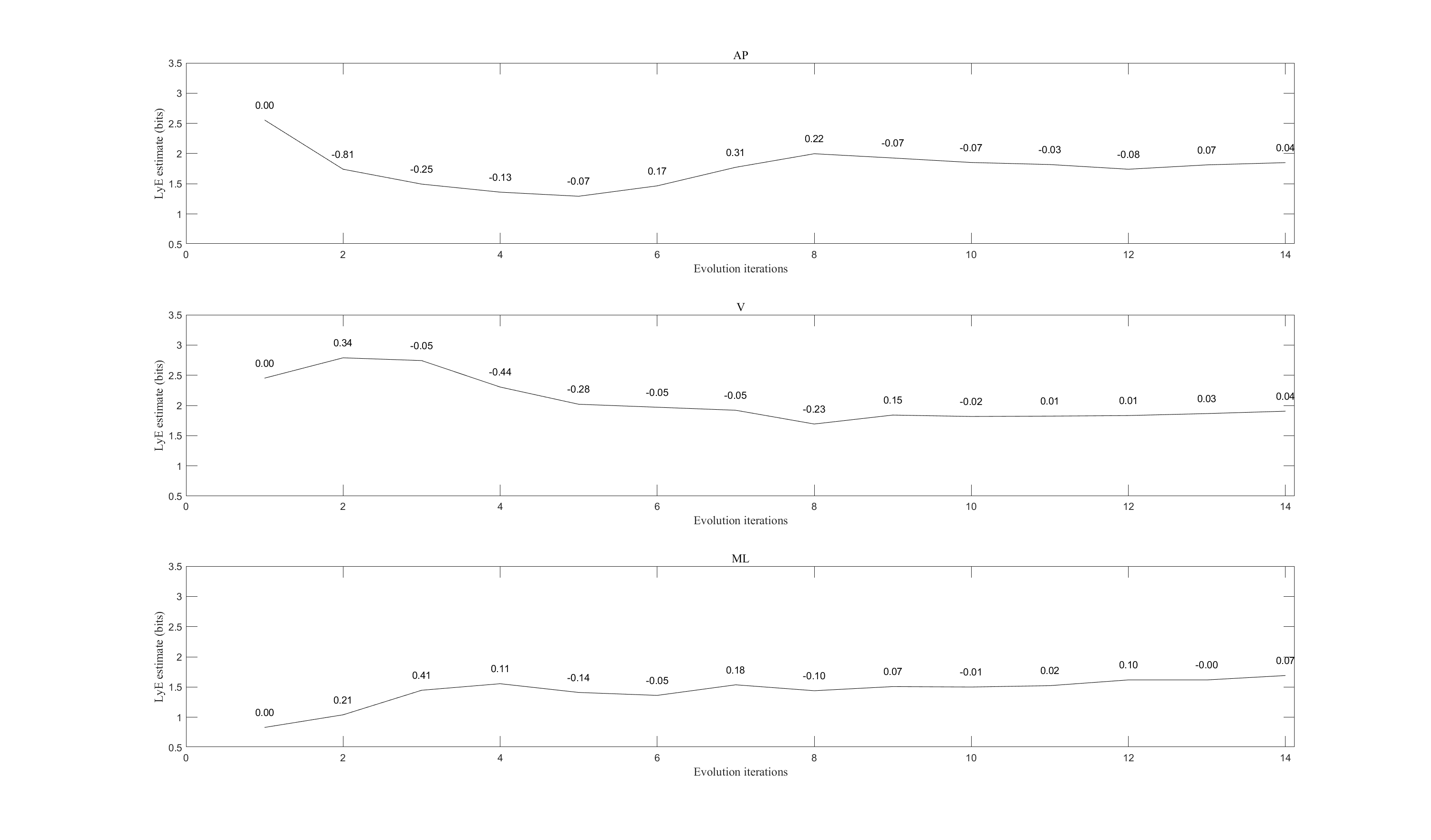

Supplement: Supplementary file 2 — Supplementary Information. [file 41598_2020_79584_MOESM2_ESM.zip › Participant12_trial10.png]

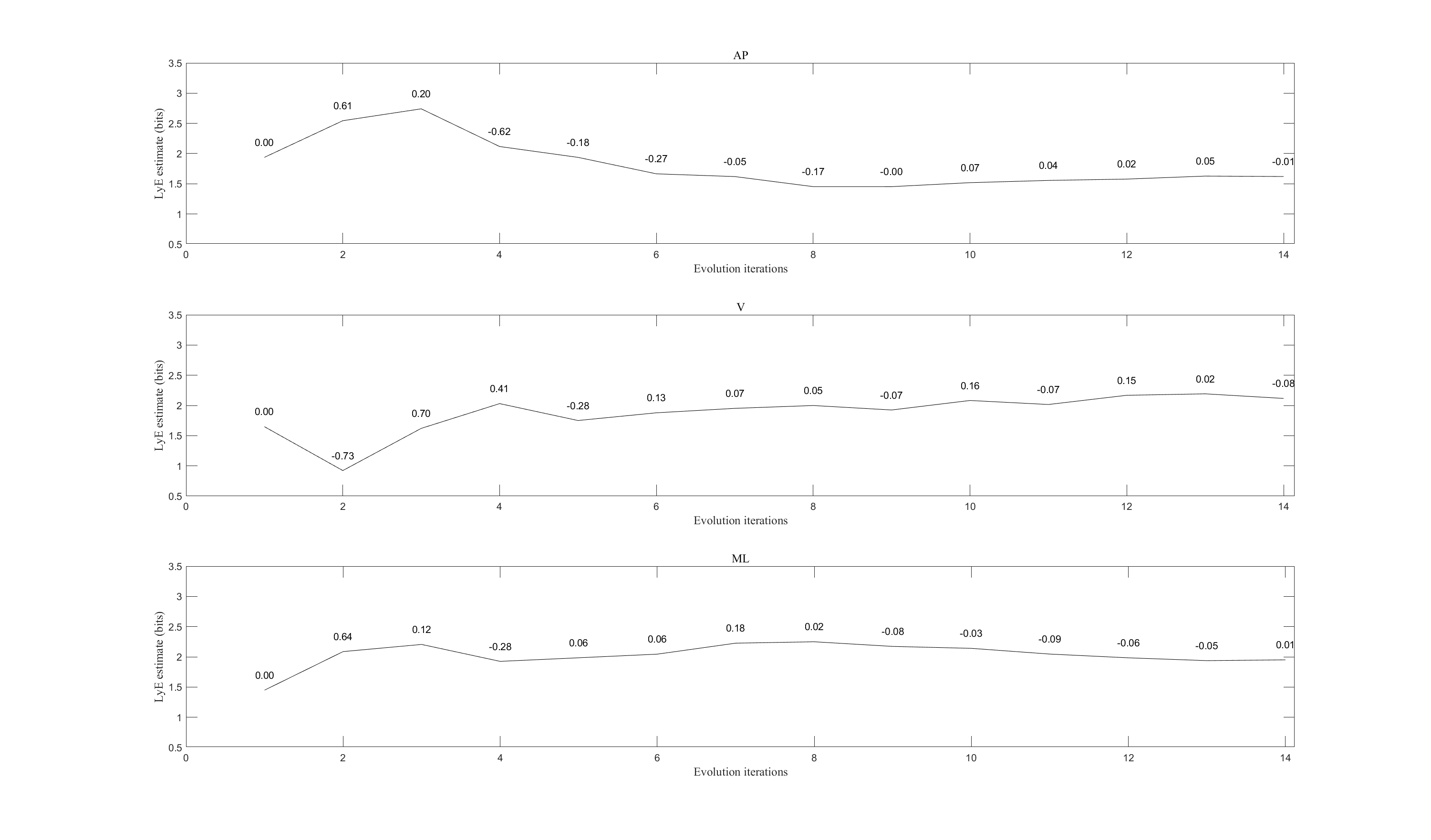

Supplement: Supplementary file 2 — Supplementary Information. [file 41598_2020_79584_MOESM2_ESM.zip › Participant12_trial11.png]

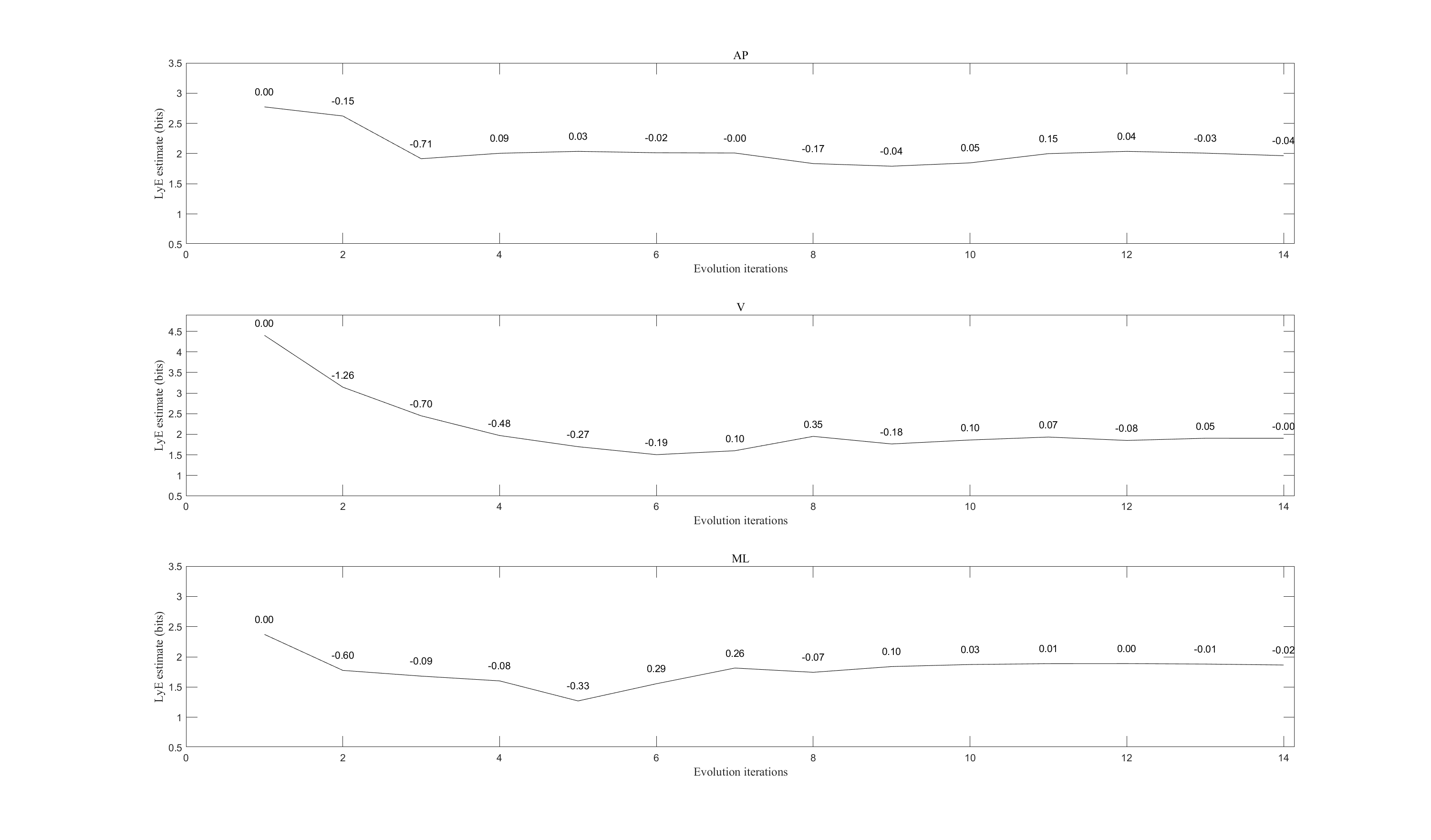

Supplement: Supplementary file 2 — Supplementary Information. [file 41598_2020_79584_MOESM2_ESM.zip › Participant12_trial12.png]

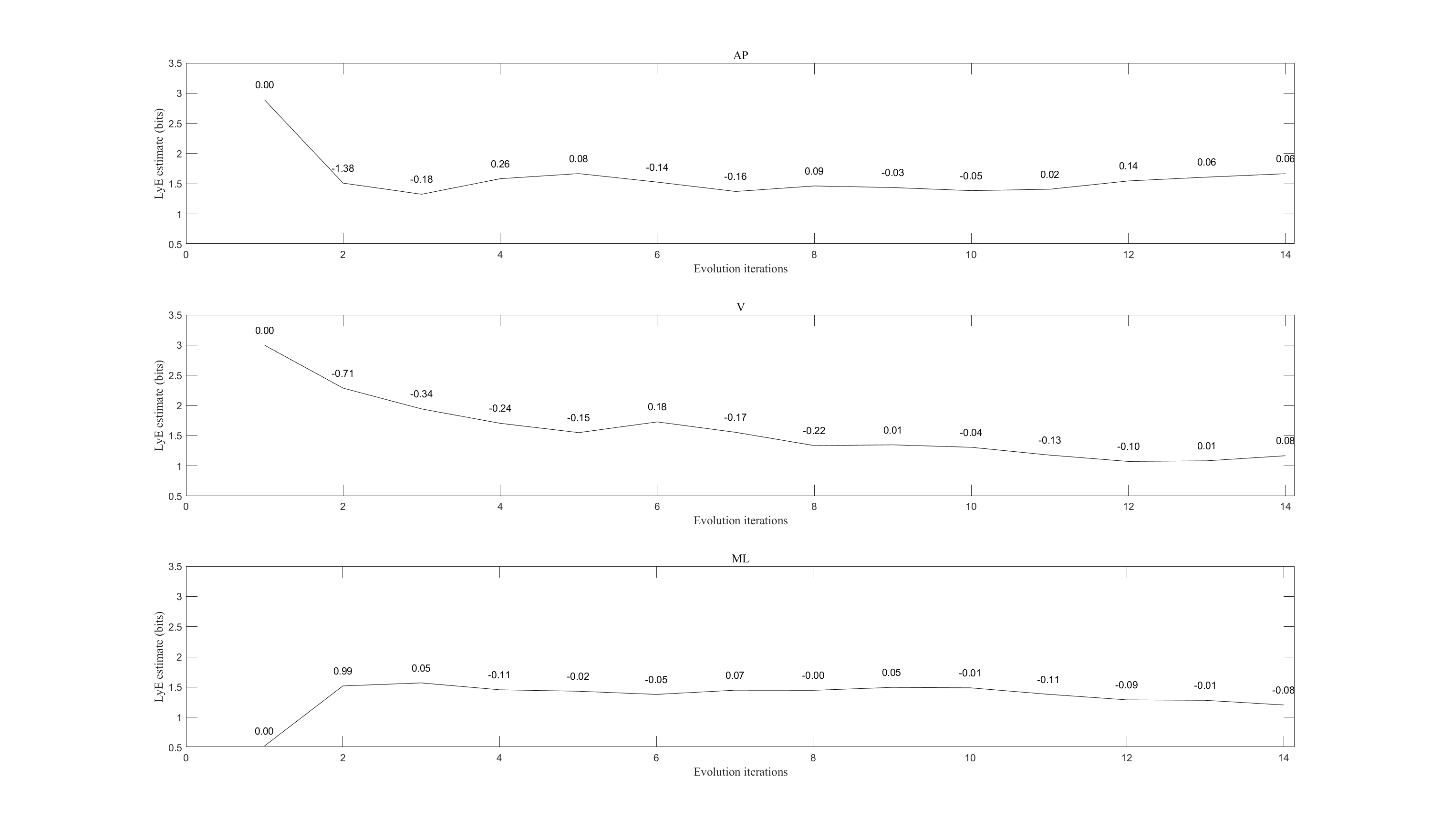

Supplement: Supplementary file 2 — Supplementary Information. [file 41598_2020_79584_MOESM2_ESM.zip › Participant12_trial2.png]

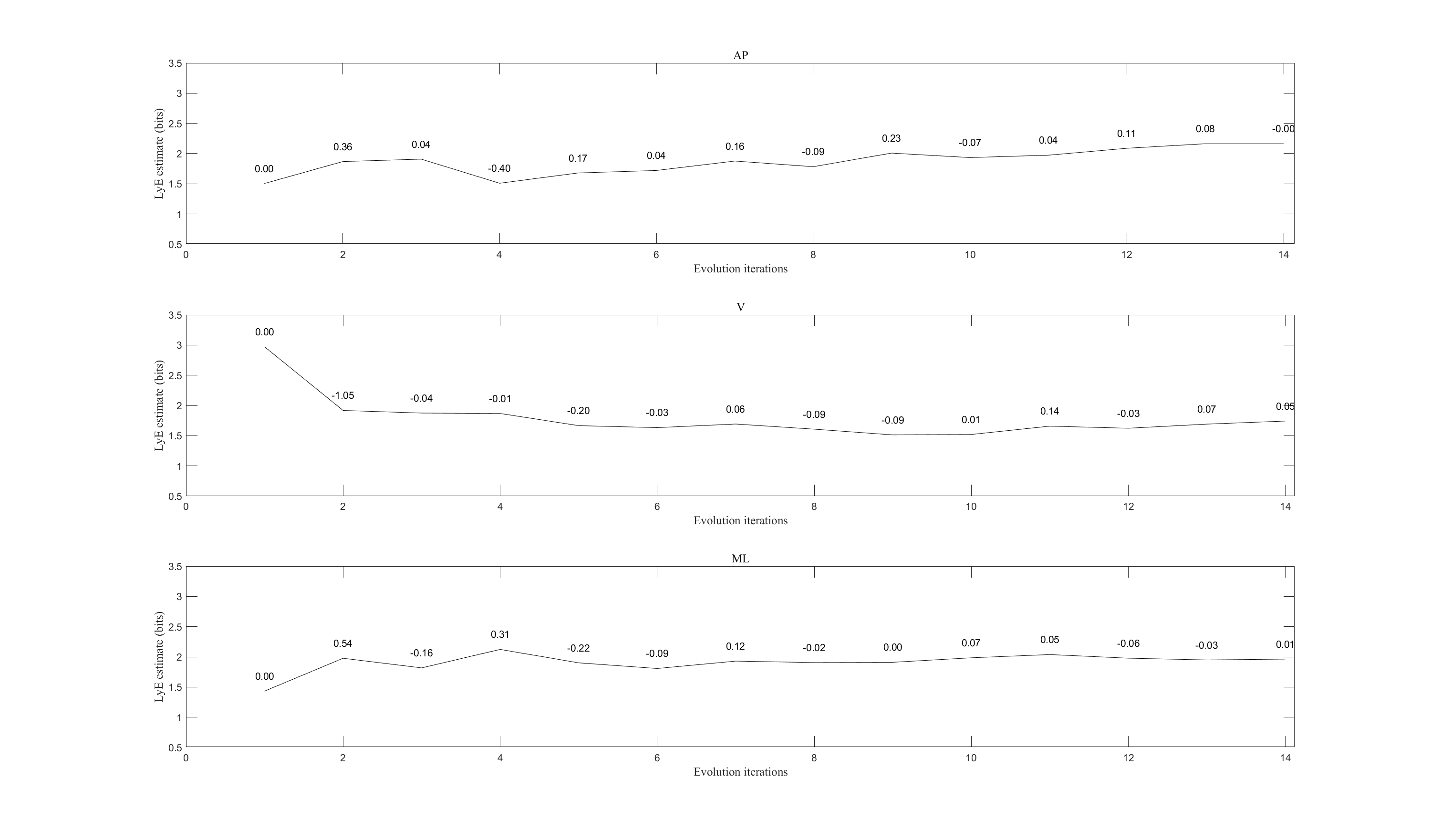

Supplement: Supplementary file 2 — Supplementary Information. [file 41598_2020_79584_MOESM2_ESM.zip › Participant12_trial3.png]

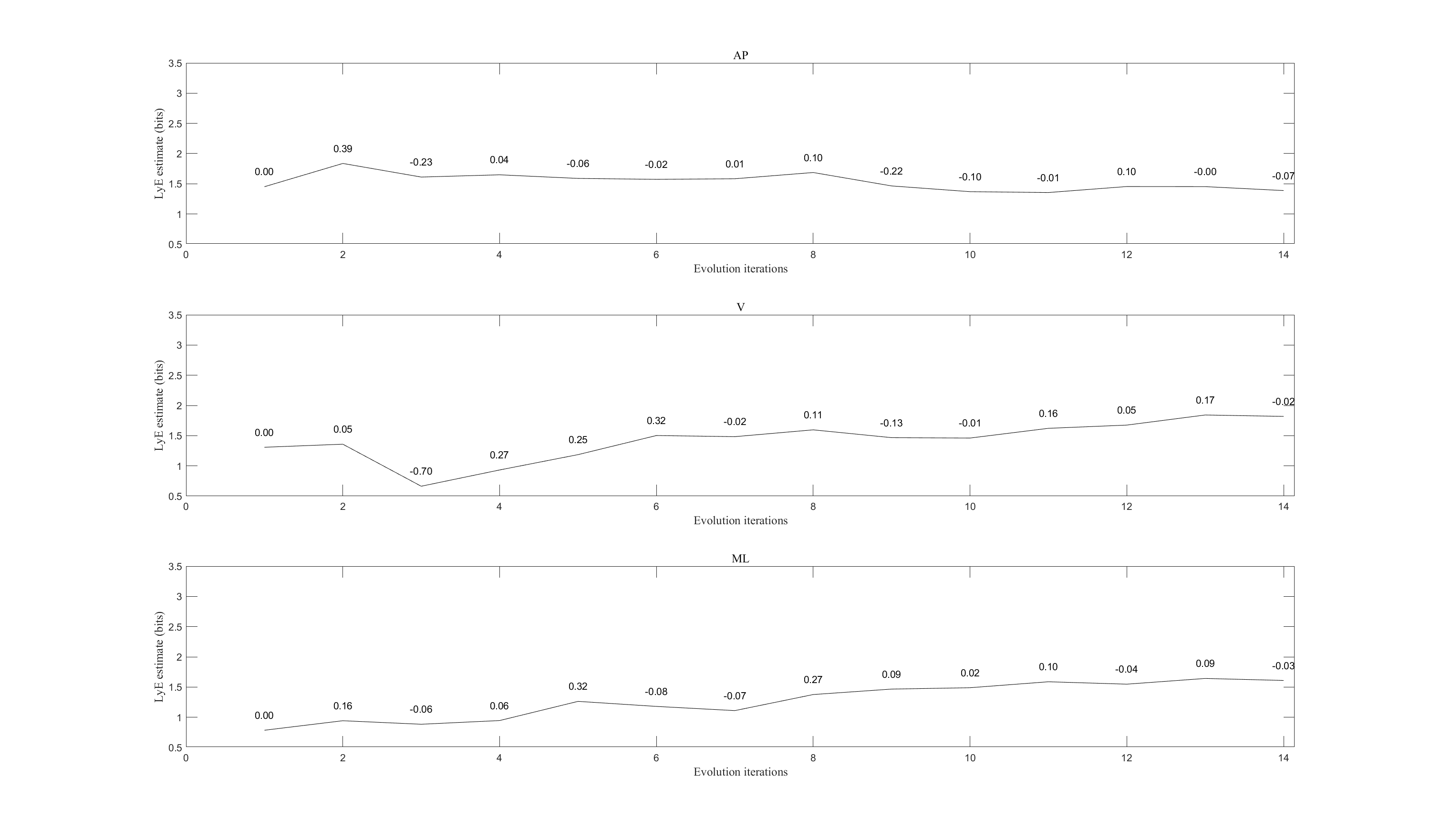

Supplement: Supplementary file 2 — Supplementary Information. [file 41598_2020_79584_MOESM2_ESM.zip › Participant12_trial4.png]

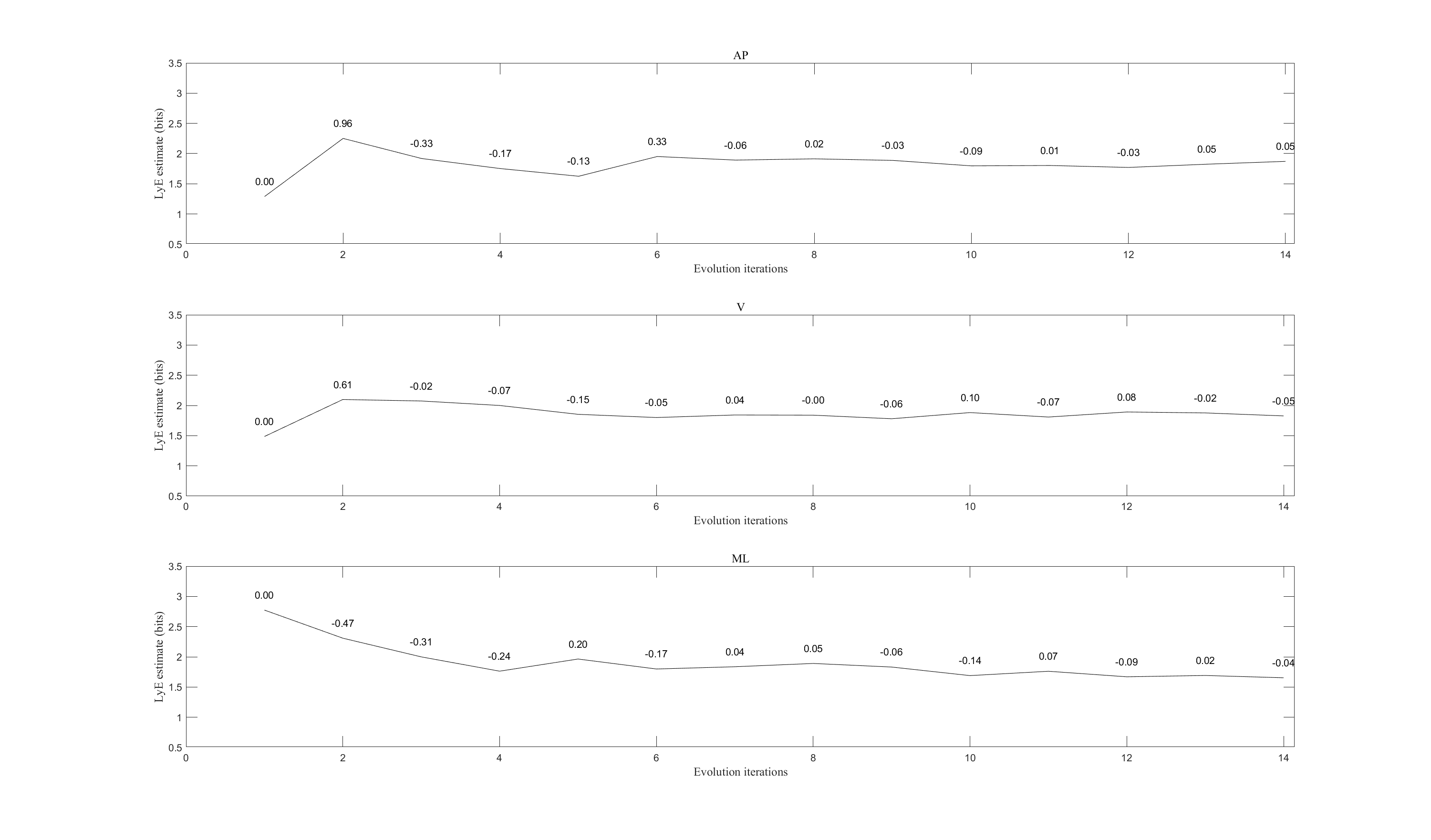

Supplement: Supplementary file 2 — Supplementary Information. [file 41598_2020_79584_MOESM2_ESM.zip › Participant12_trial5.png]

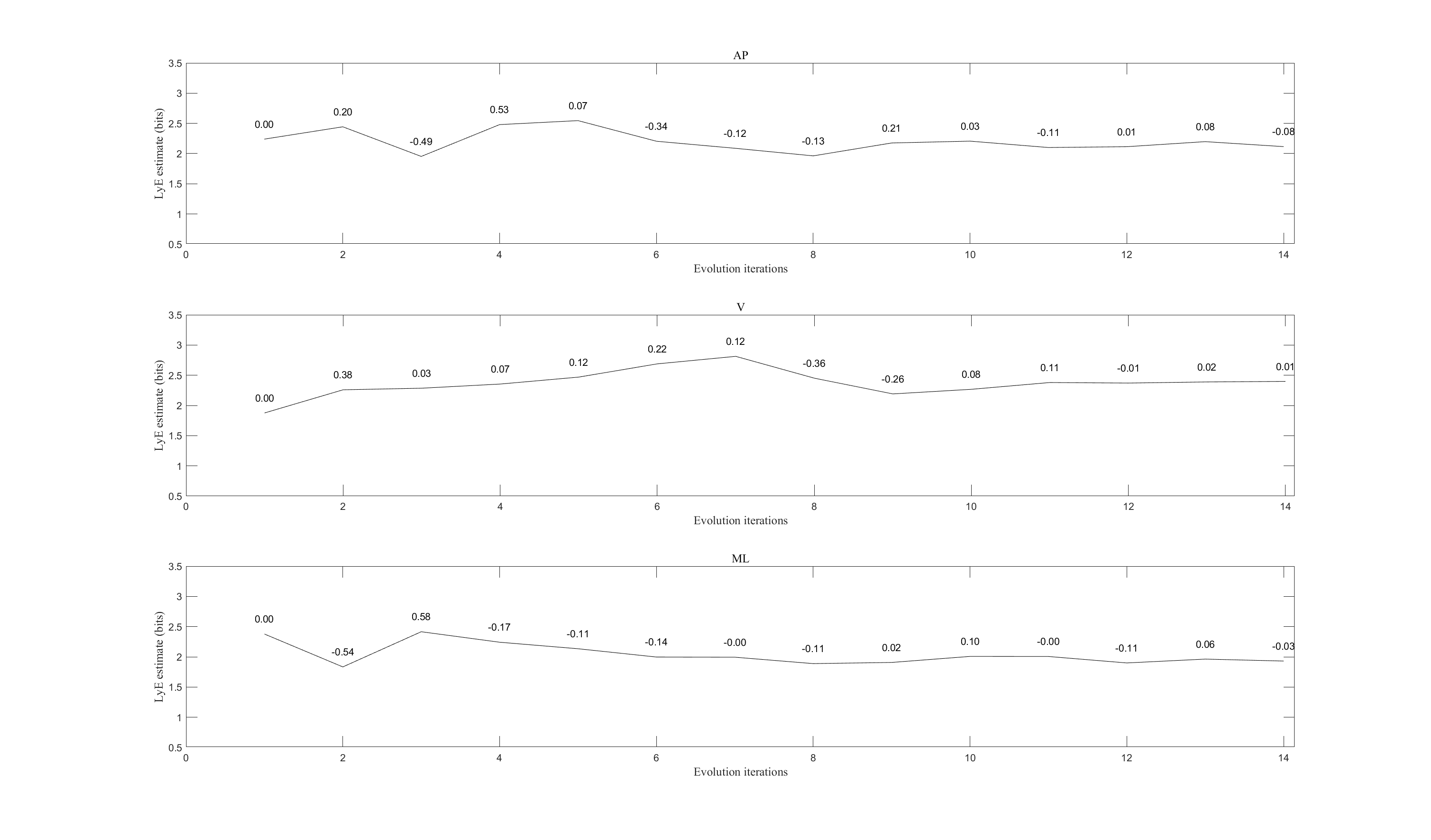

Supplement: Supplementary file 2 — Supplementary Information. [file 41598_2020_79584_MOESM2_ESM.zip › Participant12_trial6.png]

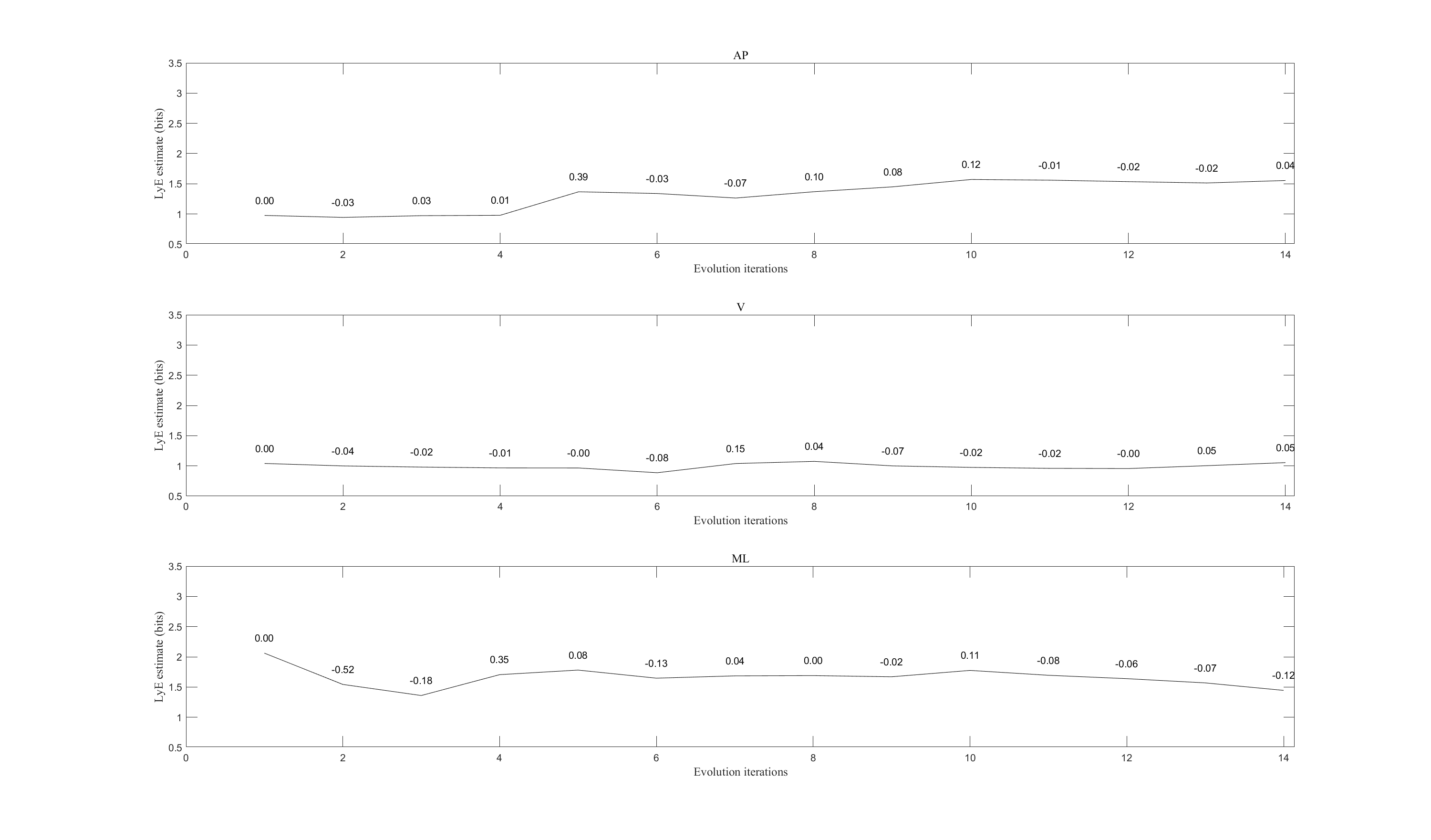

Supplement: Supplementary file 2 — Supplementary Information. [file 41598_2020_79584_MOESM2_ESM.zip › Participant12_trial7.png]

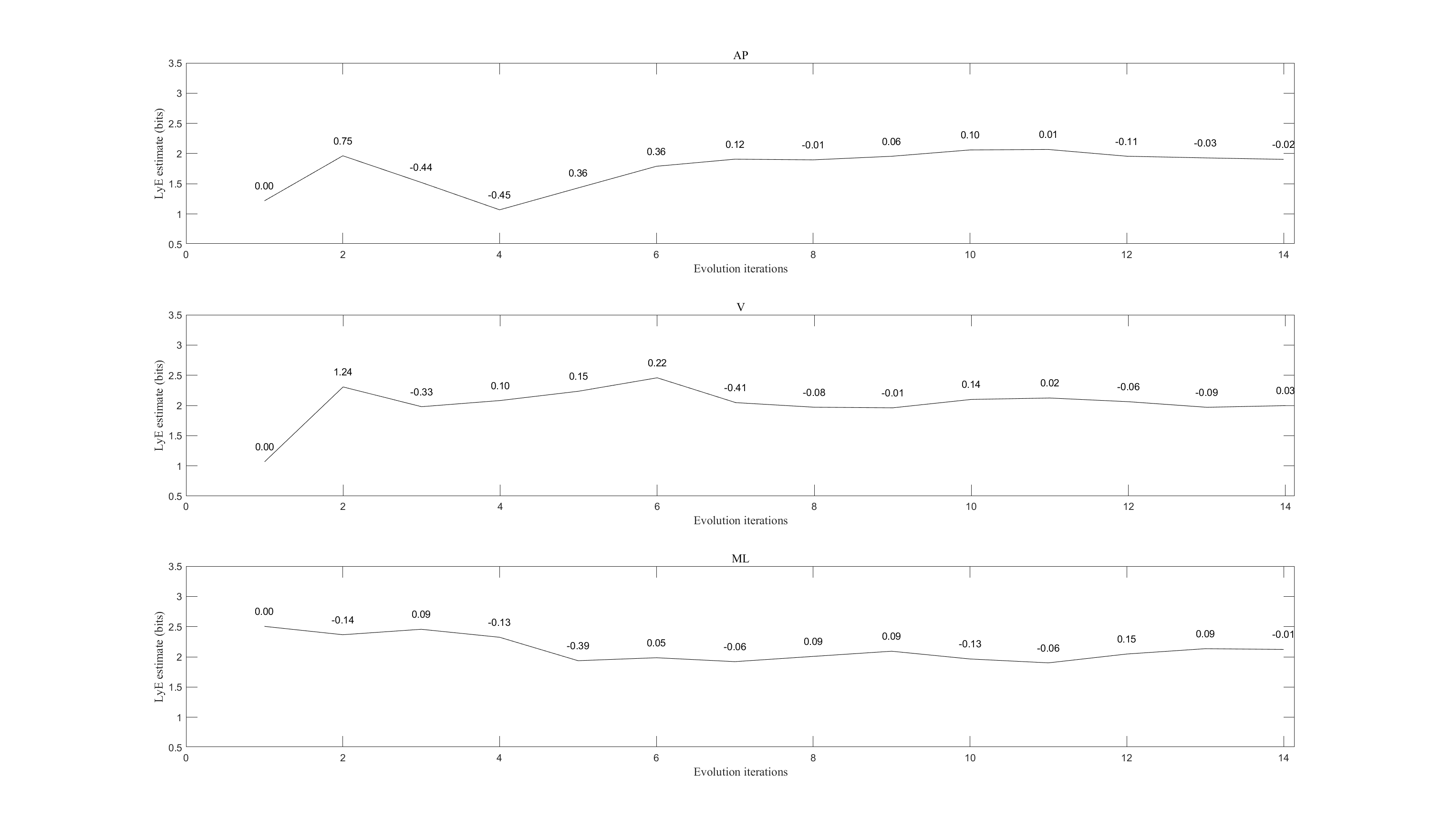

Supplement: Supplementary file 2 — Supplementary Information. [file 41598_2020_79584_MOESM2_ESM.zip › Participant12_trial8.png]

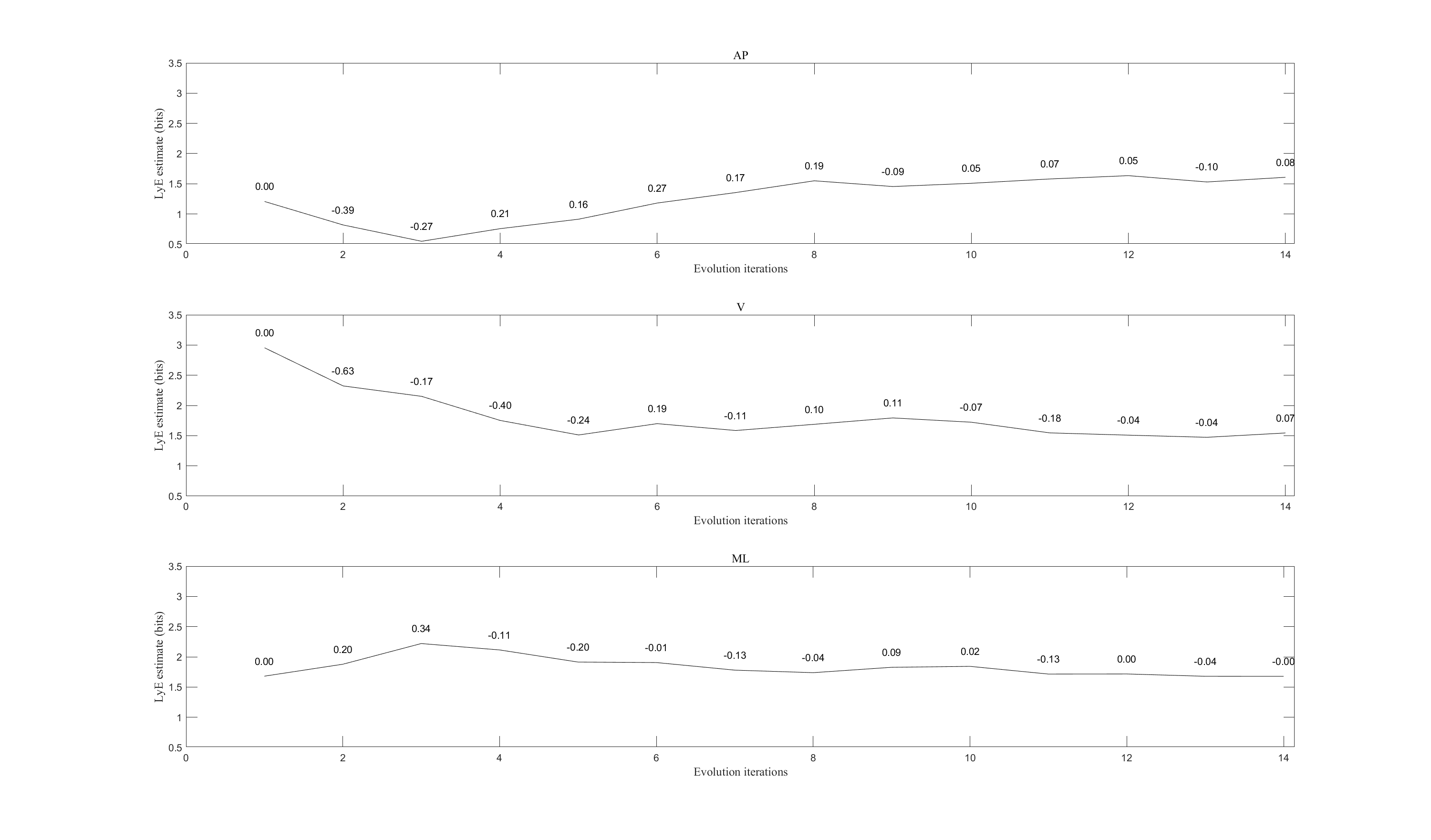

Supplement: Supplementary file 2 — Supplementary Information. [file 41598_2020_79584_MOESM2_ESM.zip › Participant12_trial9.png]

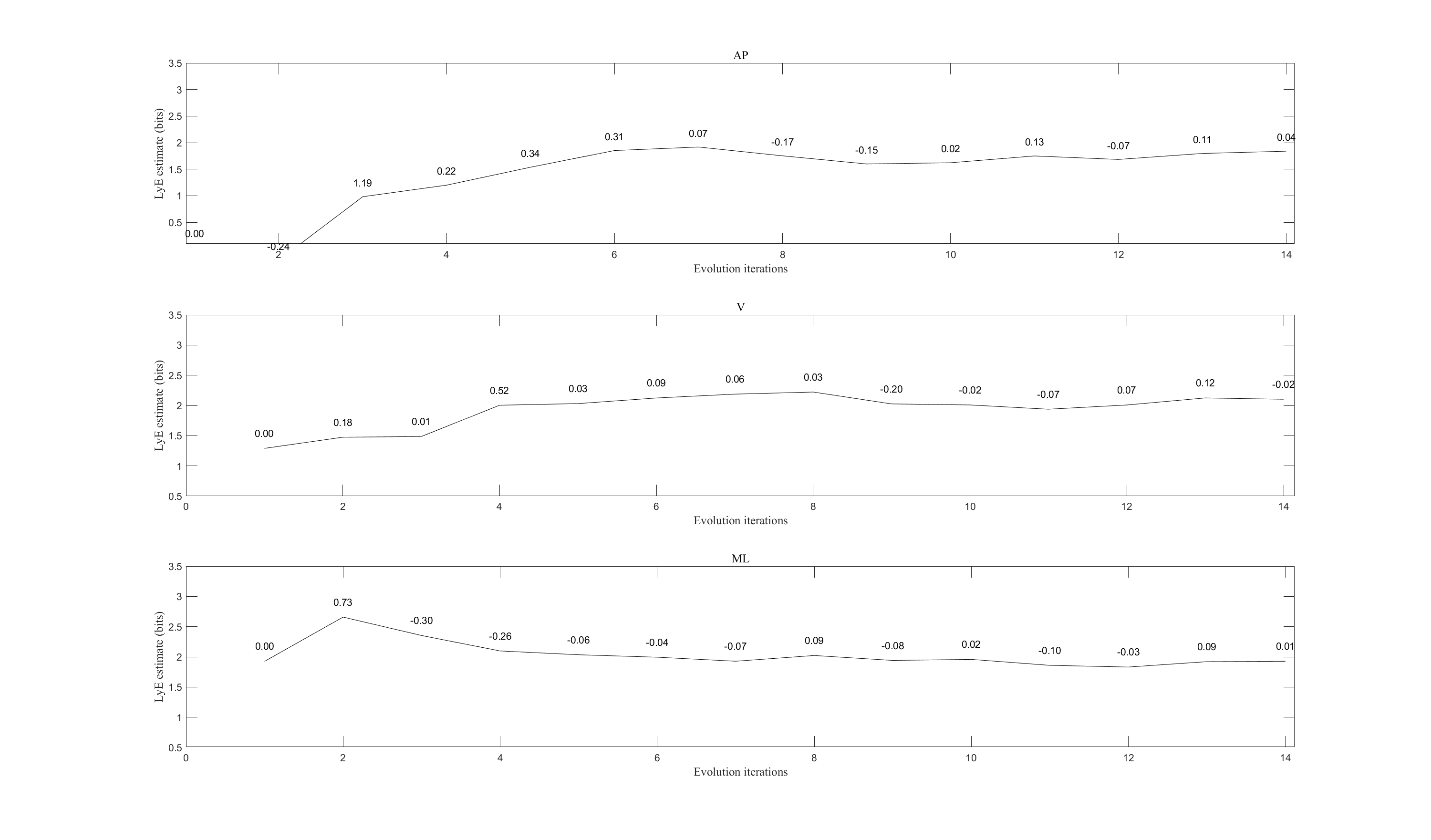

Supplement: Supplementary file 2 — Supplementary Information. [file 41598_2020_79584_MOESM2_ESM.zip › Participant13_trial1.png]

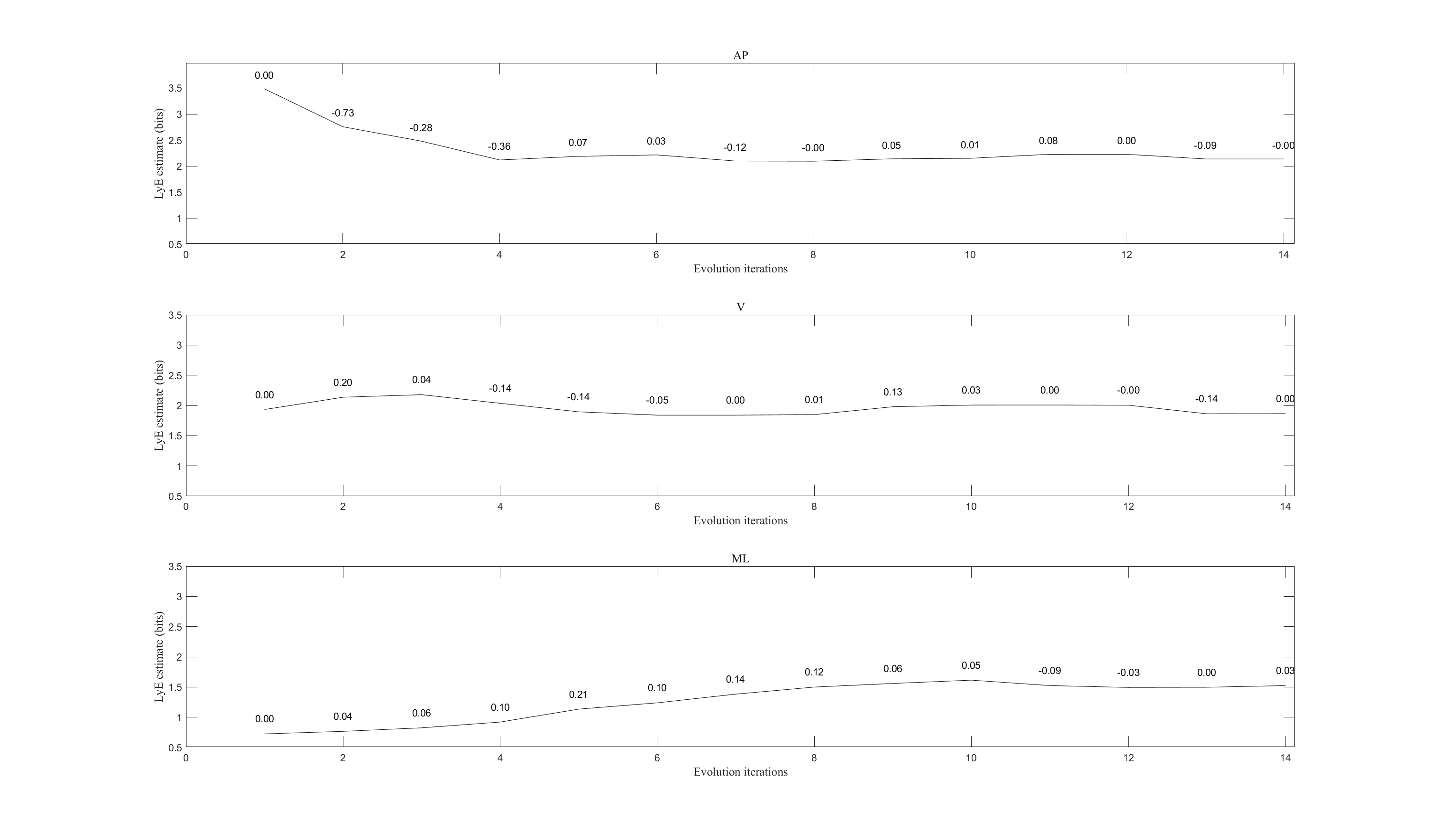

Supplement: Supplementary file 2 — Supplementary Information. [file 41598_2020_79584_MOESM2_ESM.zip › Participant13_trial10.png]

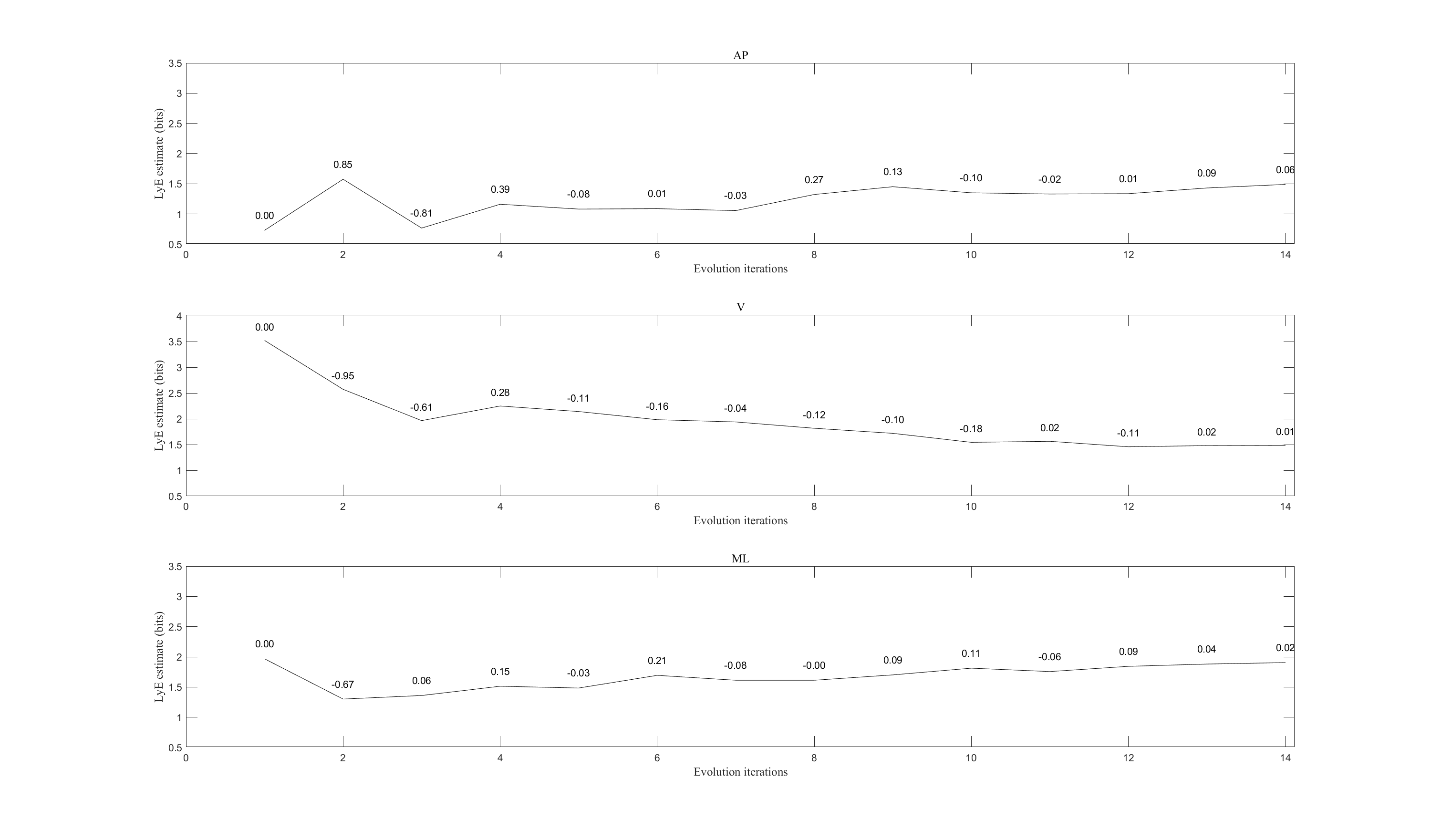

Supplement: Supplementary file 2 — Supplementary Information. [file 41598_2020_79584_MOESM2_ESM.zip › Participant13_trial11.png]

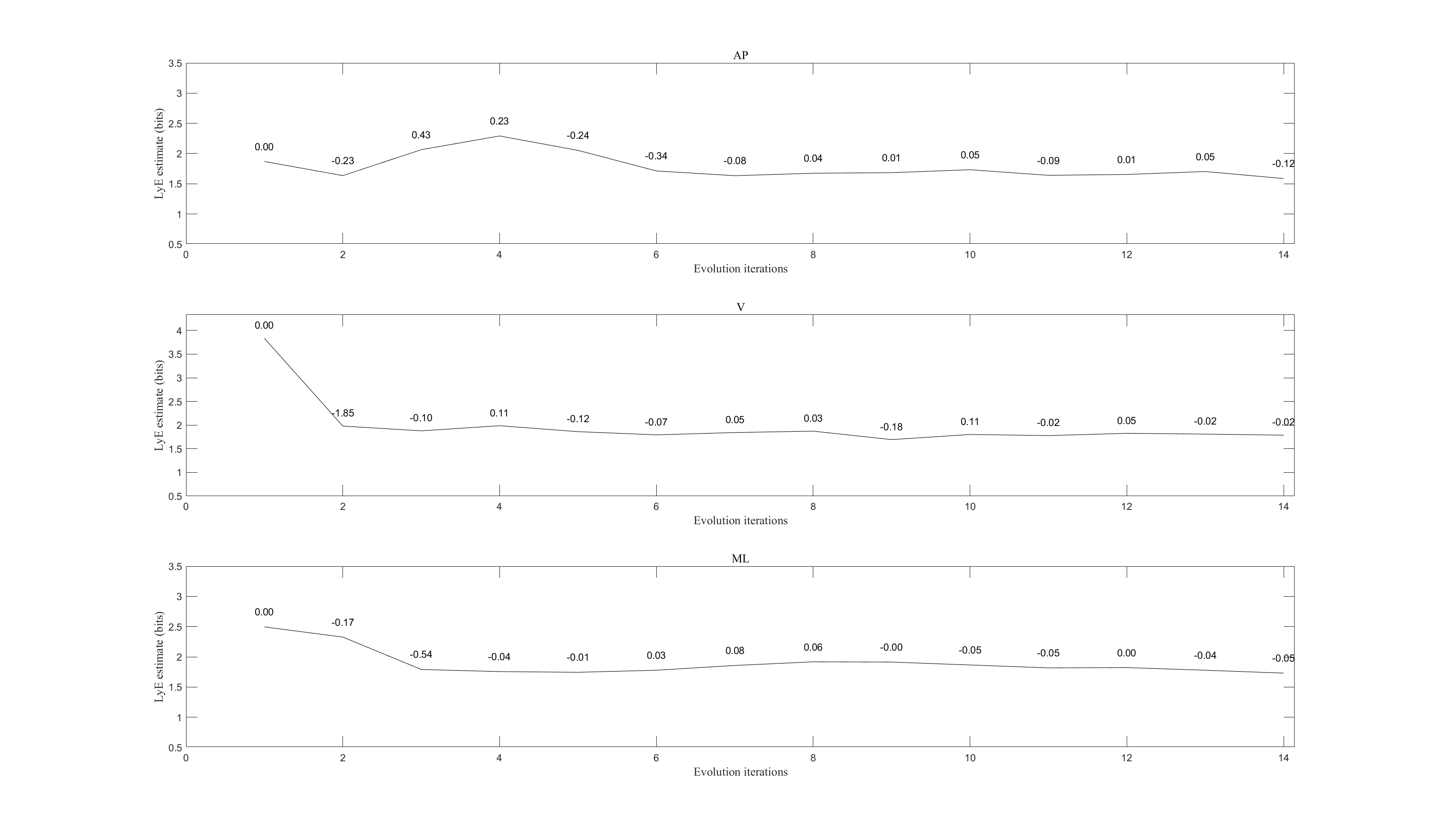

Supplement: Supplementary file 2 — Supplementary Information. [file 41598_2020_79584_MOESM2_ESM.zip › Participant13_trial12.png]

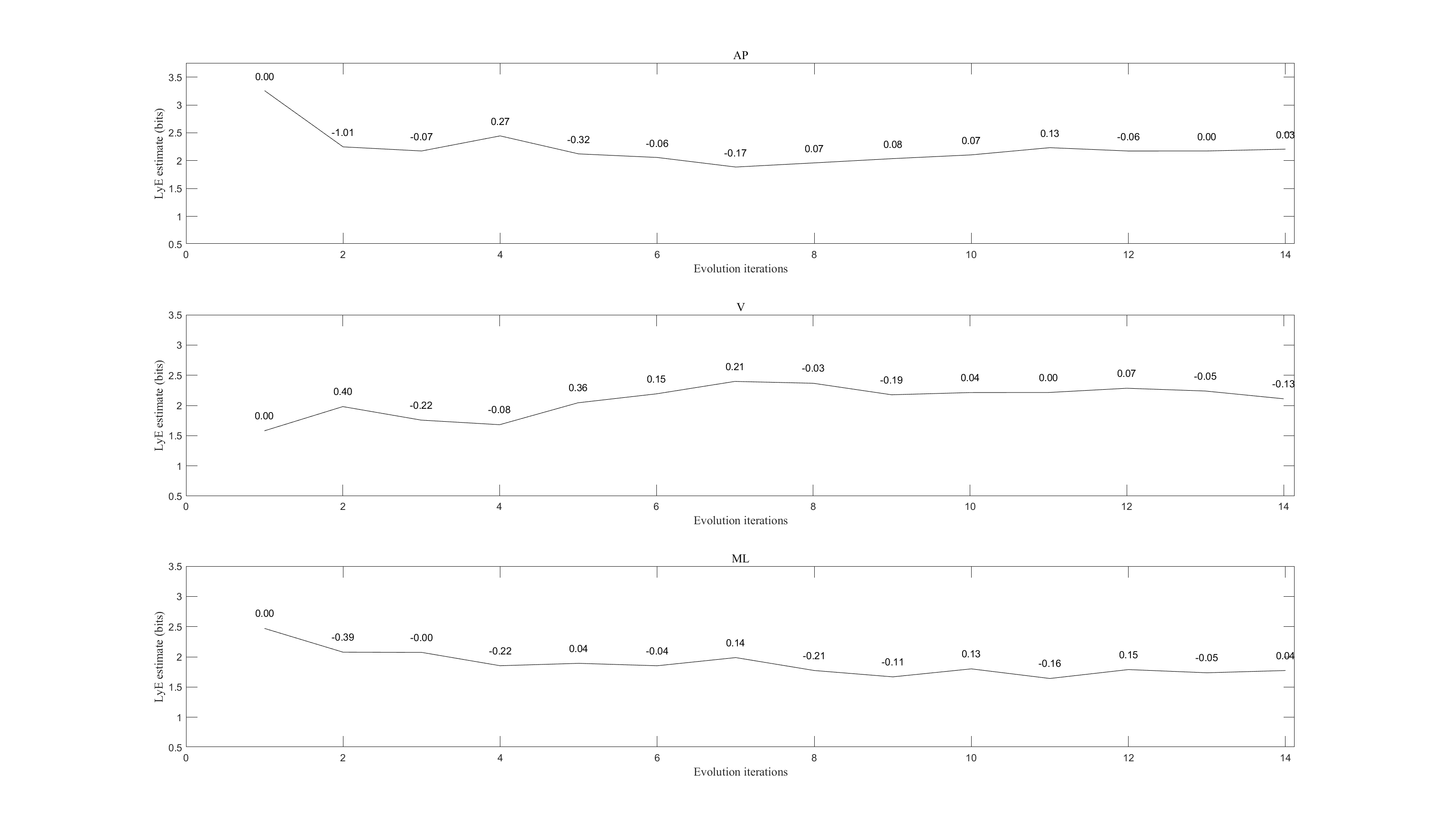

Supplement: Supplementary file 2 — Supplementary Information. [file 41598_2020_79584_MOESM2_ESM.zip › Participant13_trial2.png]

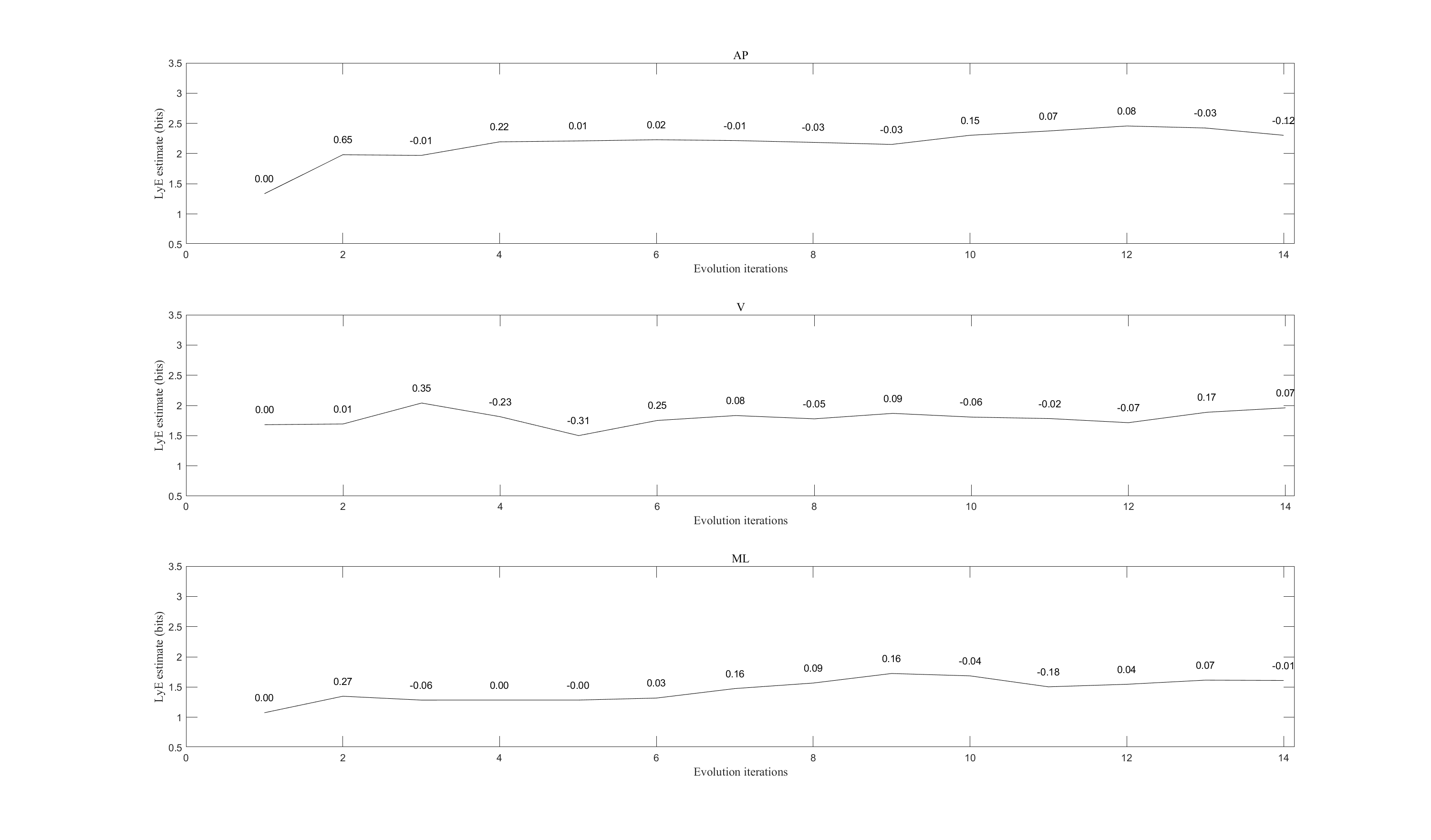

Supplement: Supplementary file 2 — Supplementary Information. [file 41598_2020_79584_MOESM2_ESM.zip › Participant13_trial3.png]

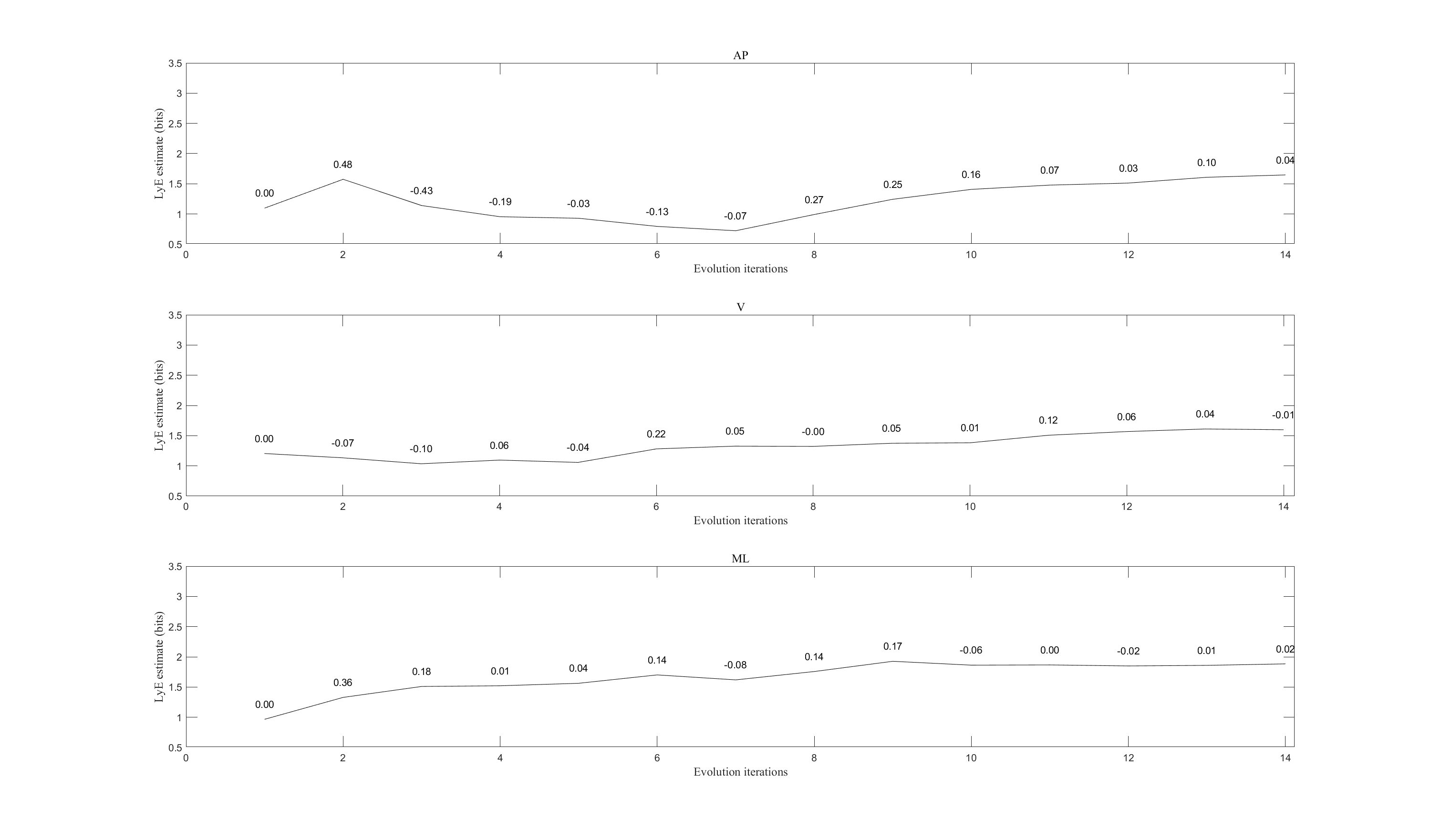

Supplement: Supplementary file 2 — Supplementary Information. [file 41598_2020_79584_MOESM2_ESM.zip › Participant13_trial4.png]

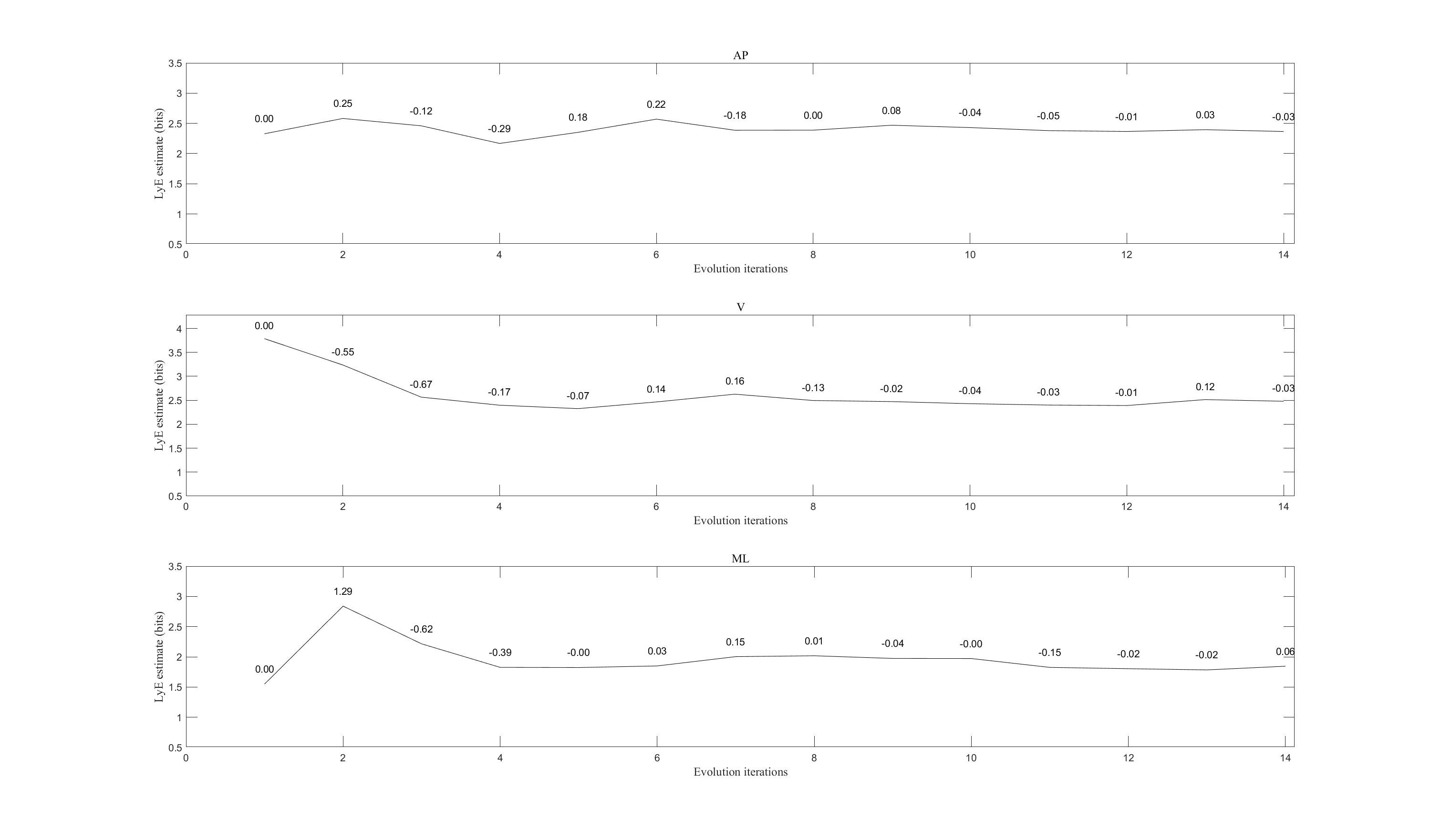

Supplement: Supplementary file 2 — Supplementary Information. [file 41598_2020_79584_MOESM2_ESM.zip › Participant13_trial5.png]

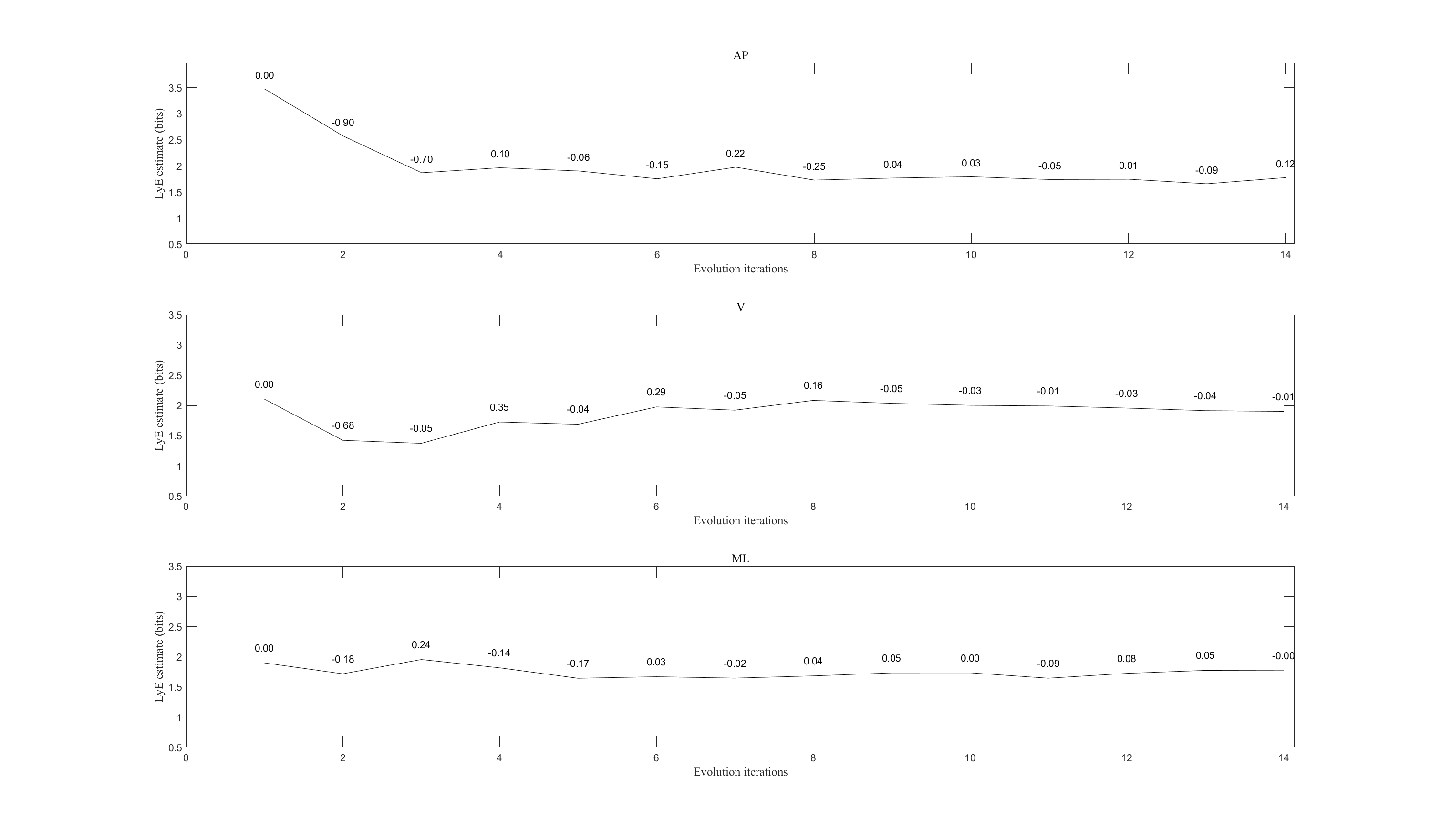

Supplement: Supplementary file 2 — Supplementary Information. [file 41598_2020_79584_MOESM2_ESM.zip › Participant13_trial6.png]

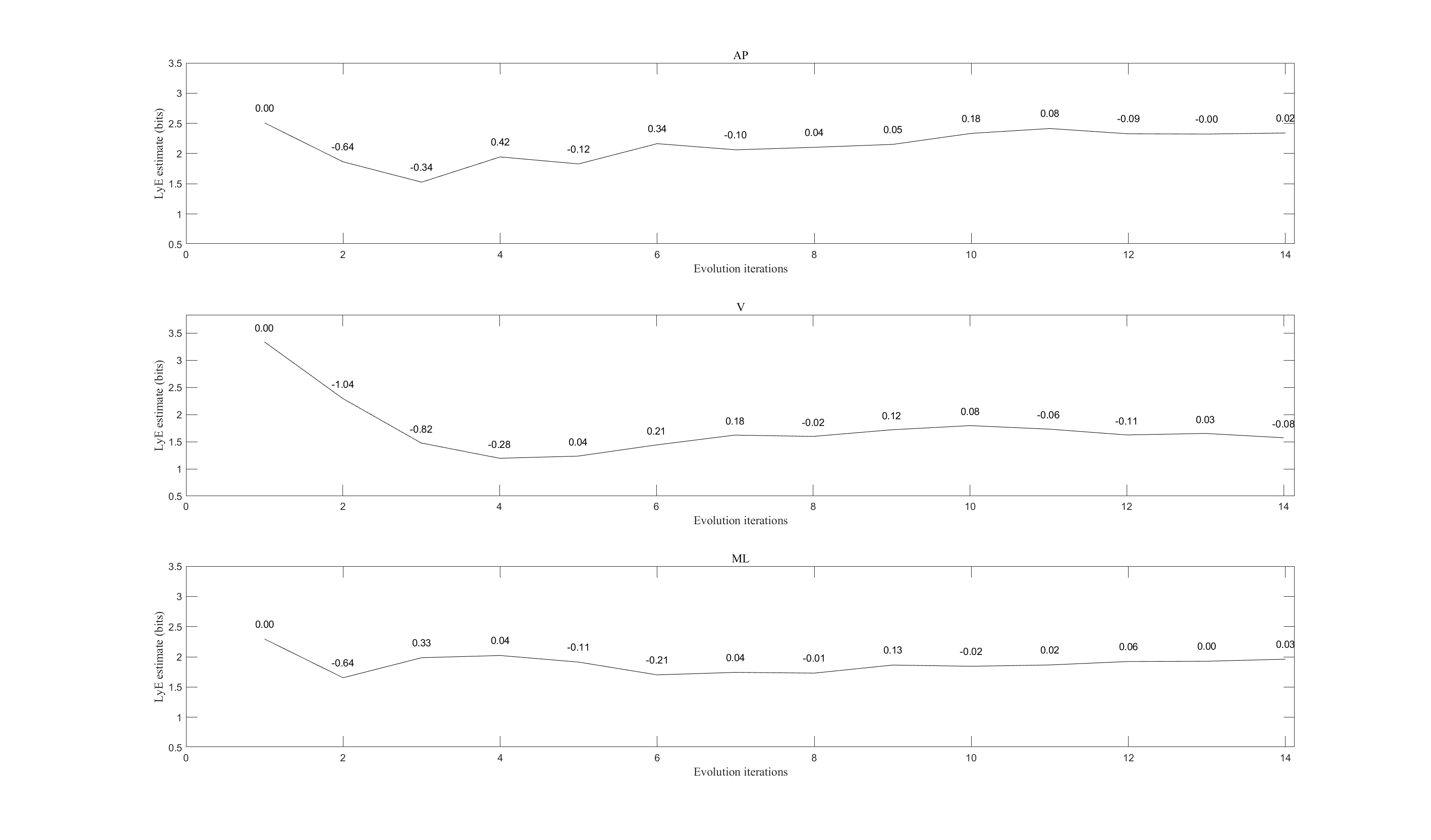

Supplement: Supplementary file 2 — Supplementary Information. [file 41598_2020_79584_MOESM2_ESM.zip › Participant13_trial7.png]

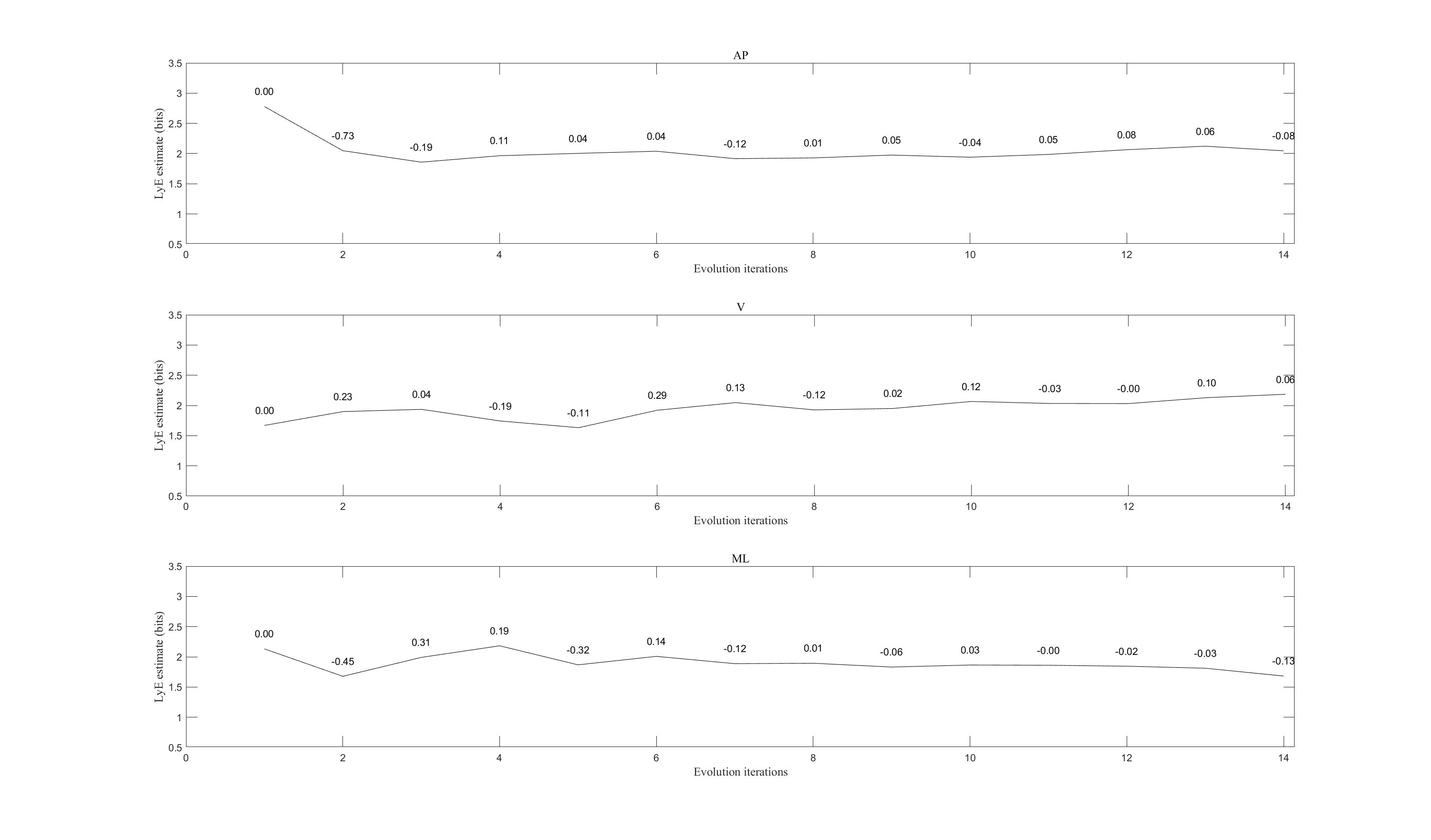

Supplement: Supplementary file 2 — Supplementary Information. [file 41598_2020_79584_MOESM2_ESM.zip › Participant13_trial8.png]

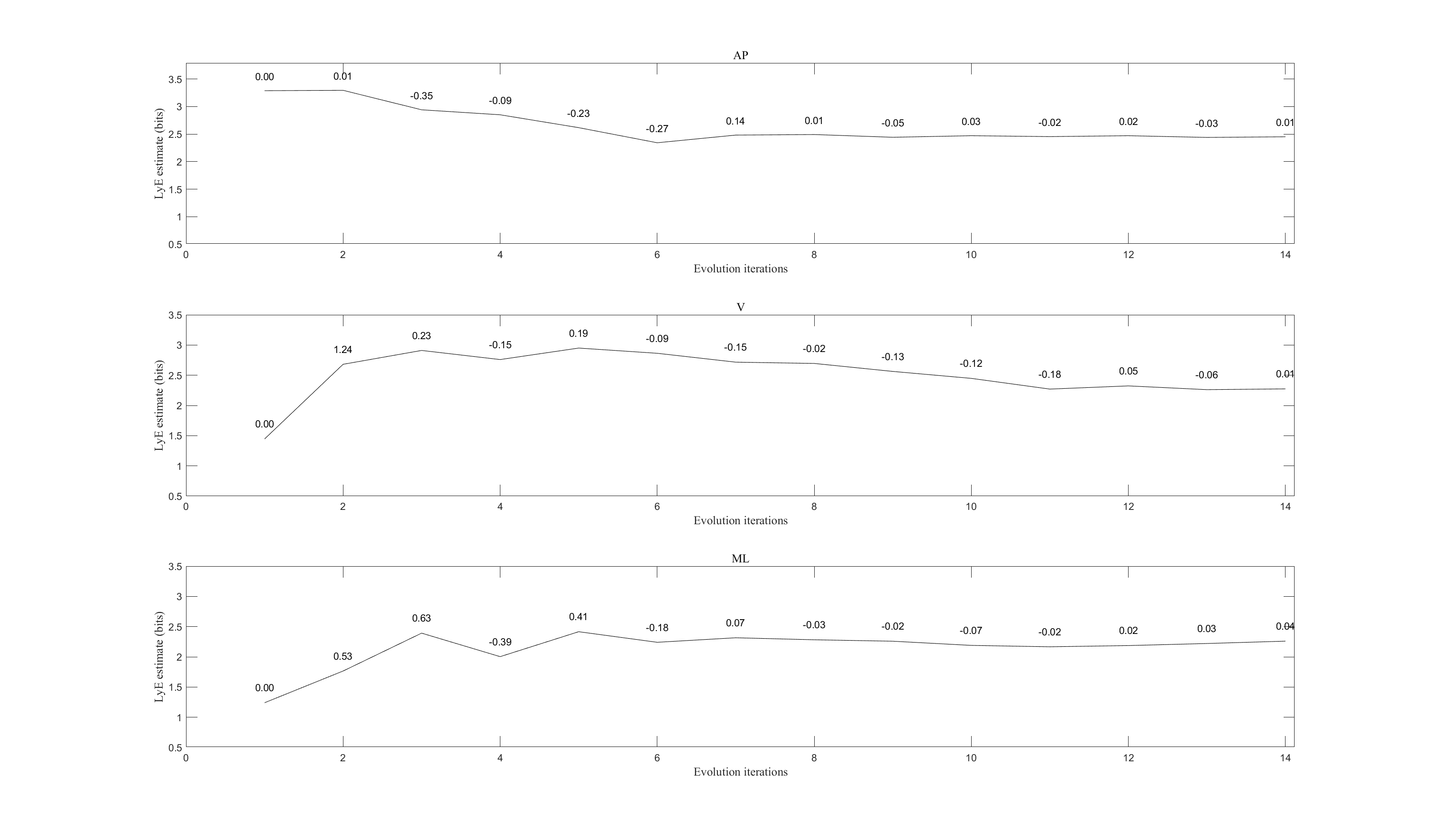

Supplement: Supplementary file 2 — Supplementary Information. [file 41598_2020_79584_MOESM2_ESM.zip › Participant13_trial9.png]

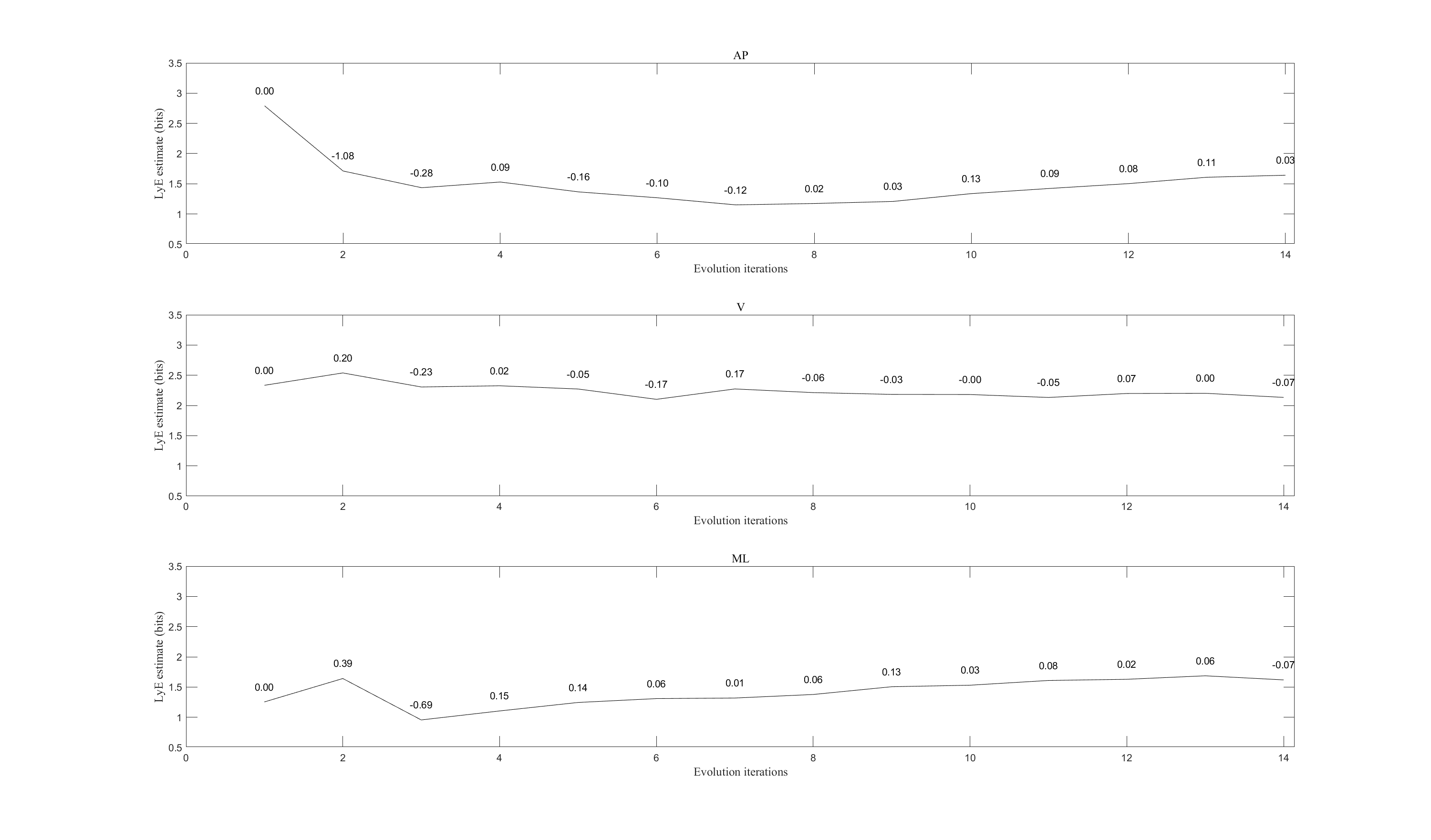

Supplement: Supplementary file 2 — Supplementary Information. [file 41598_2020_79584_MOESM2_ESM.zip › Participant14_trial1.png]

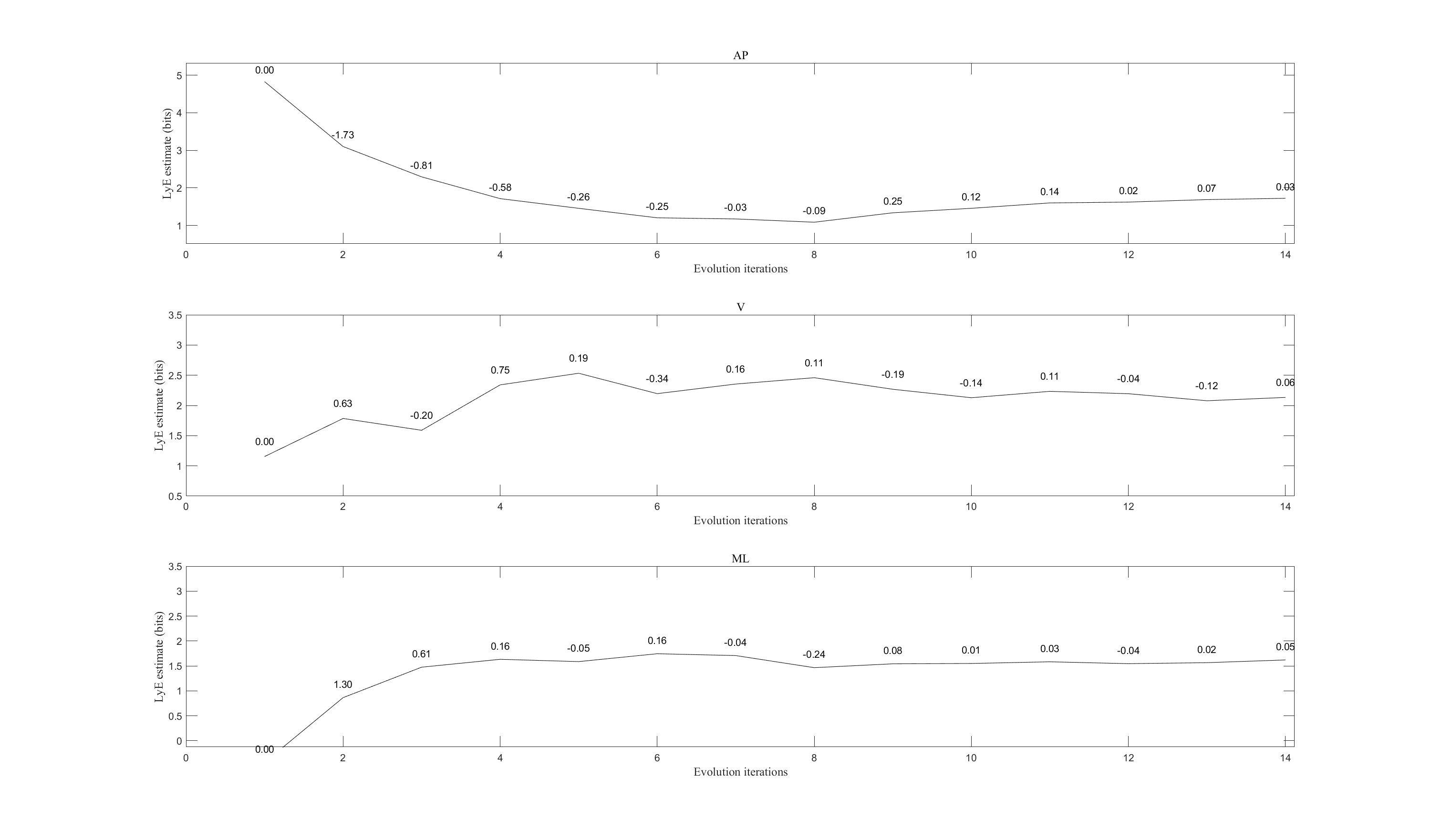

Supplement: Supplementary file 2 — Supplementary Information. [file 41598_2020_79584_MOESM2_ESM.zip › Participant14_trial10.png]

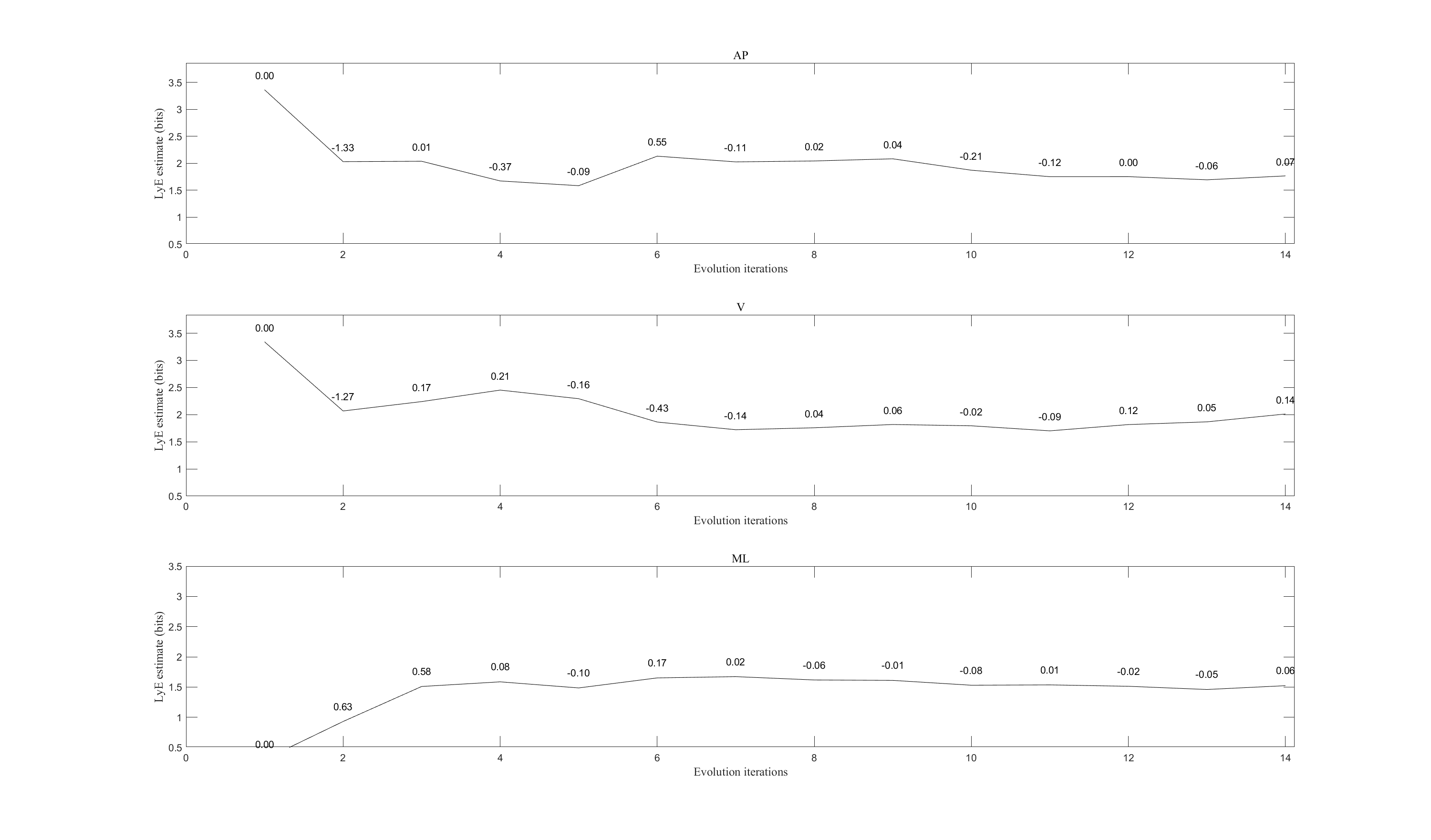

Supplement: Supplementary file 2 — Supplementary Information. [file 41598_2020_79584_MOESM2_ESM.zip › Participant14_trial11.png]

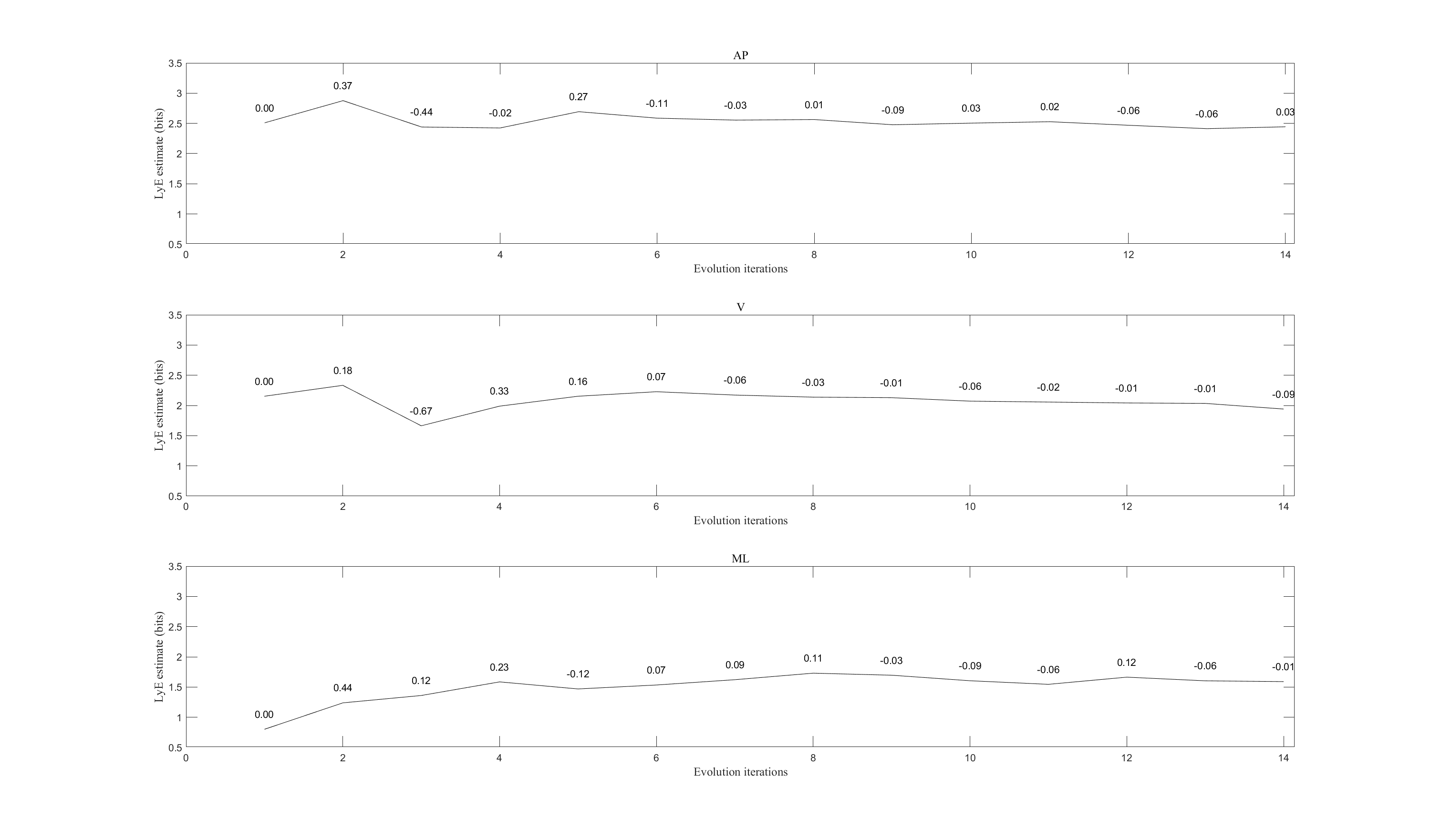

Supplement: Supplementary file 2 — Supplementary Information. [file 41598_2020_79584_MOESM2_ESM.zip › Participant14_trial12.png]

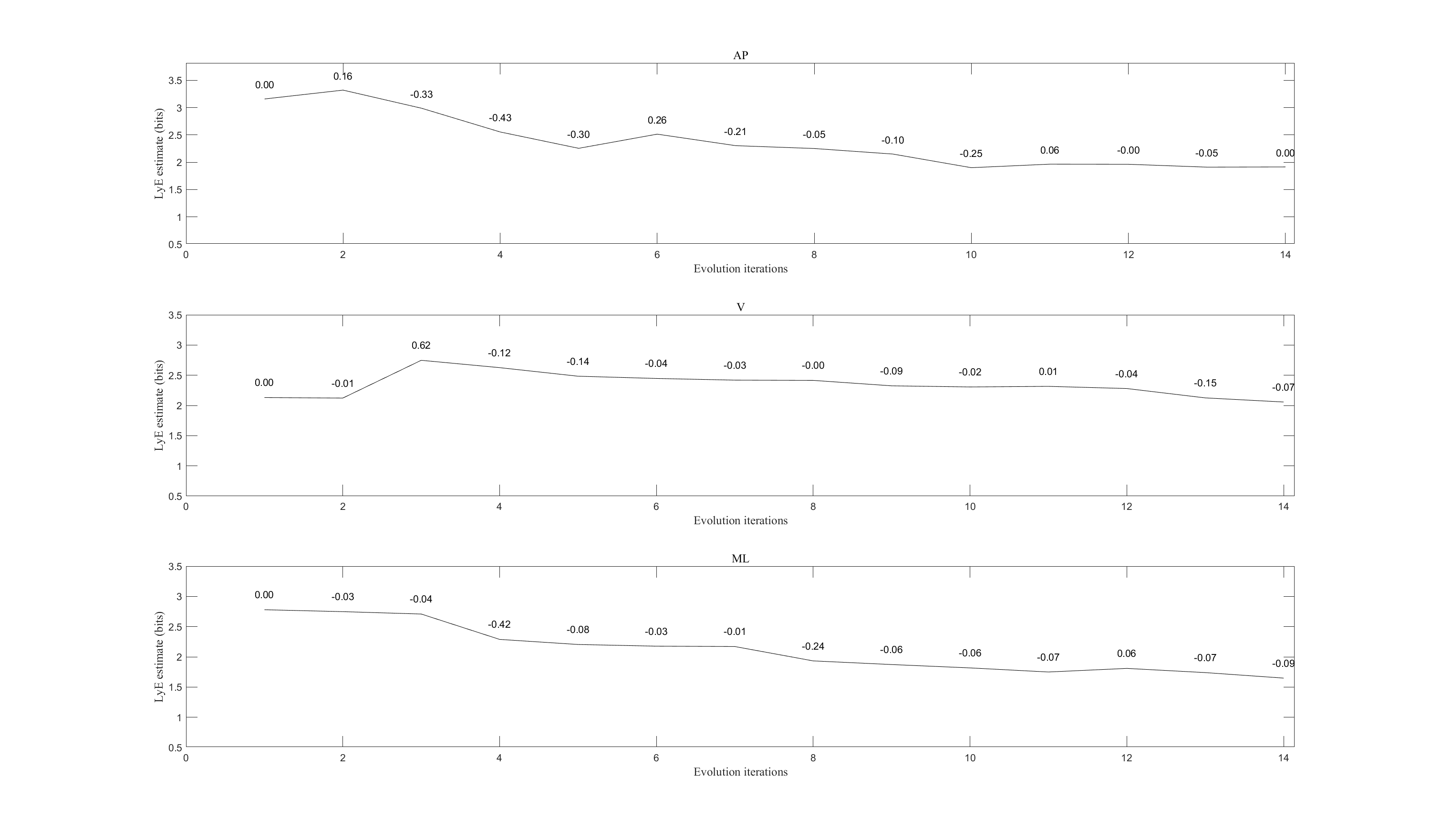

Supplement: Supplementary file 2 — Supplementary Information. [file 41598_2020_79584_MOESM2_ESM.zip › Participant14_trial2.png]

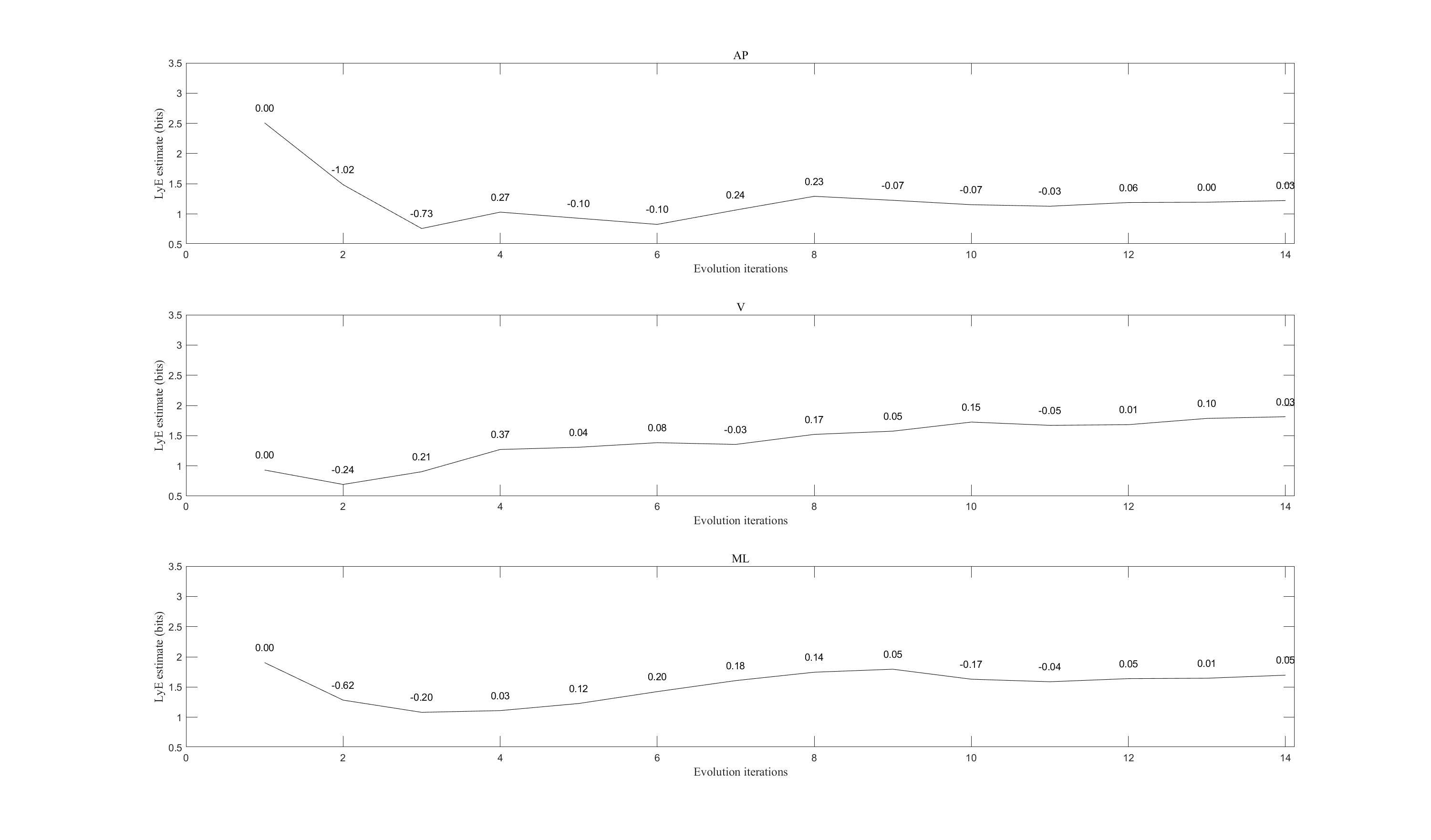

Supplement: Supplementary file 2 — Supplementary Information. [file 41598_2020_79584_MOESM2_ESM.zip › Participant14_trial3.png]

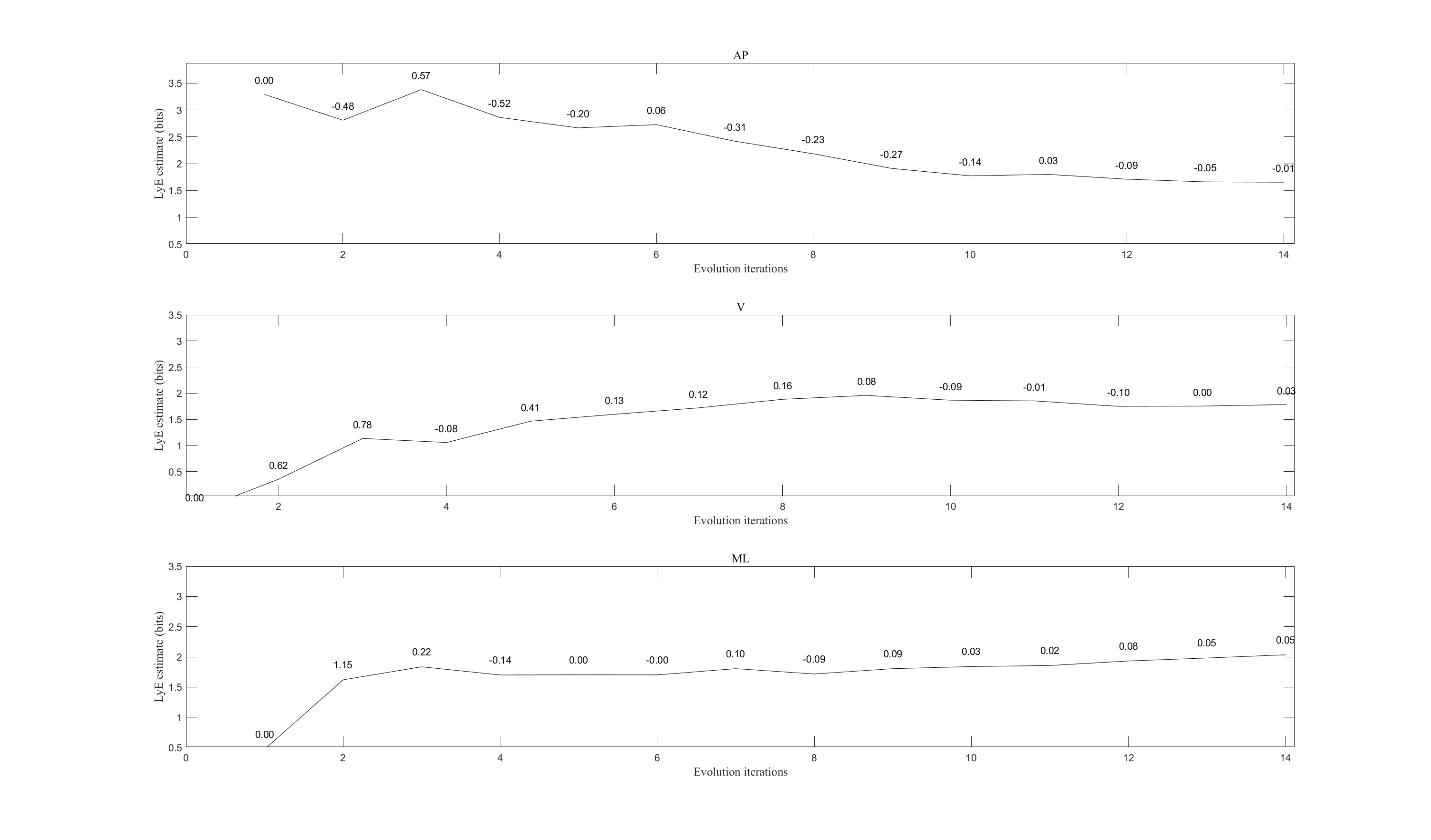

Supplement: Supplementary file 2 — Supplementary Information. [file 41598_2020_79584_MOESM2_ESM.zip › Participant14_trial4.png]

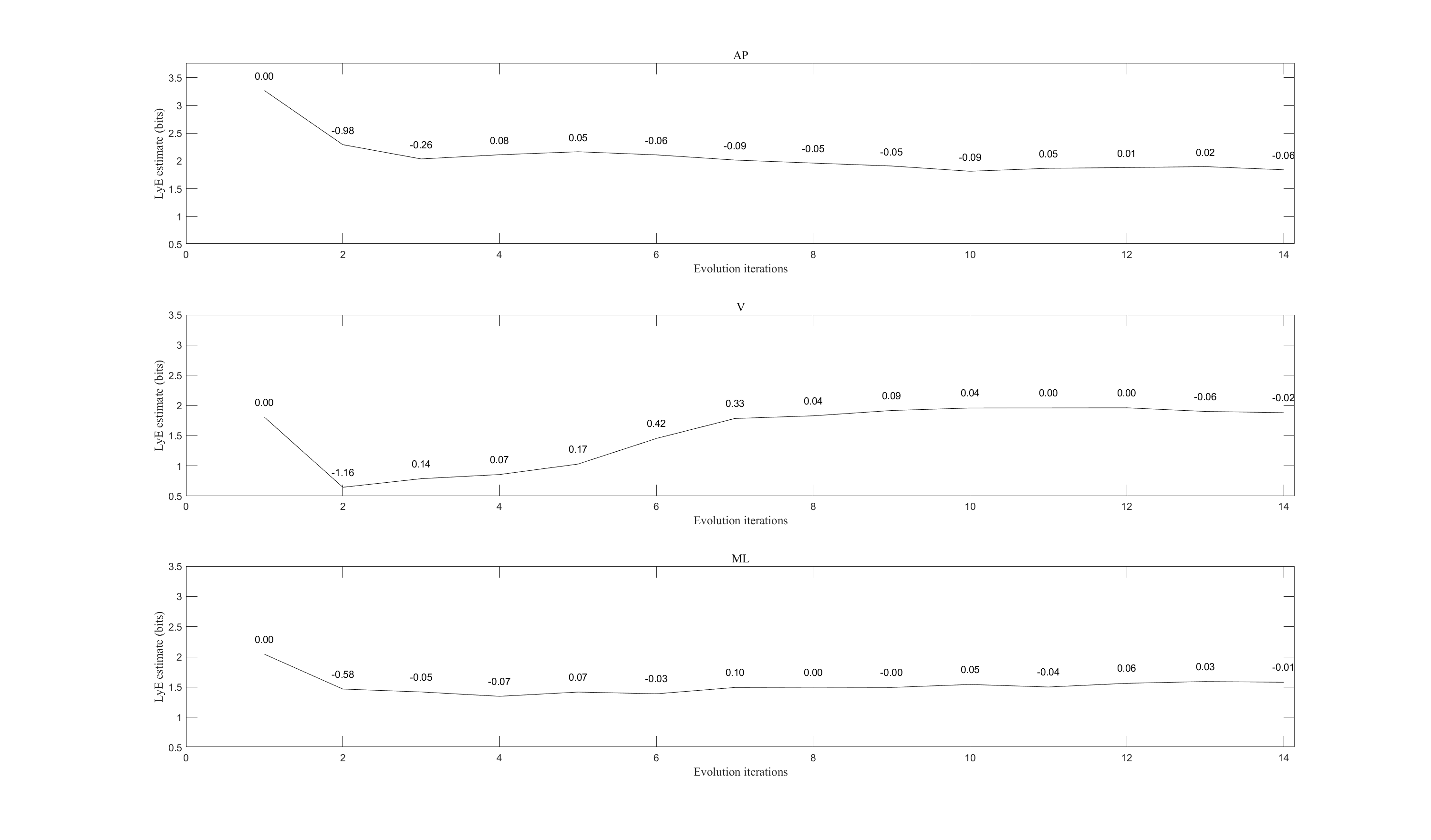

Supplement: Supplementary file 2 — Supplementary Information. [file 41598_2020_79584_MOESM2_ESM.zip › Participant14_trial5.png]

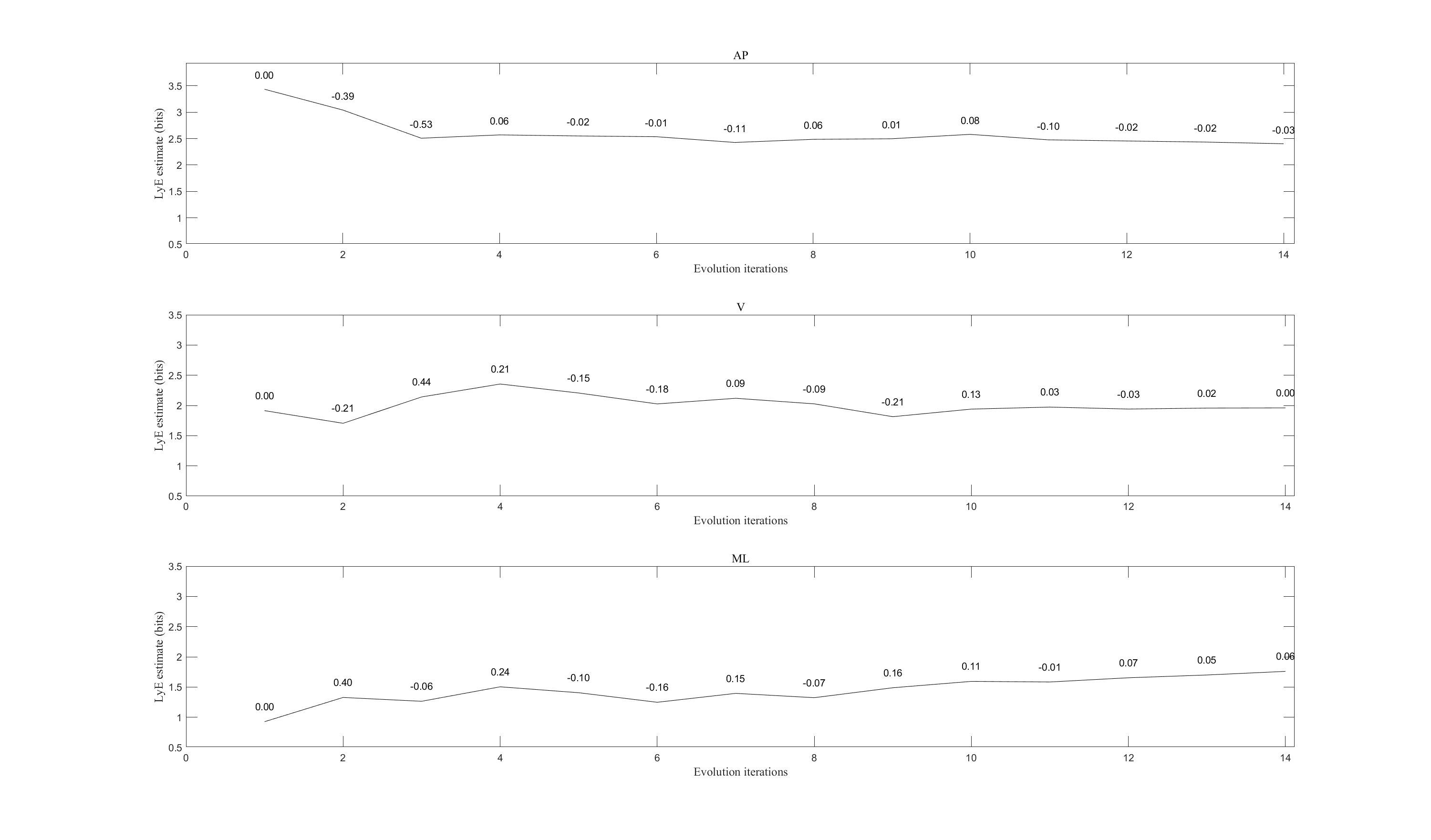

Supplement: Supplementary file 2 — Supplementary Information. [file 41598_2020_79584_MOESM2_ESM.zip › Participant14_trial6.png]

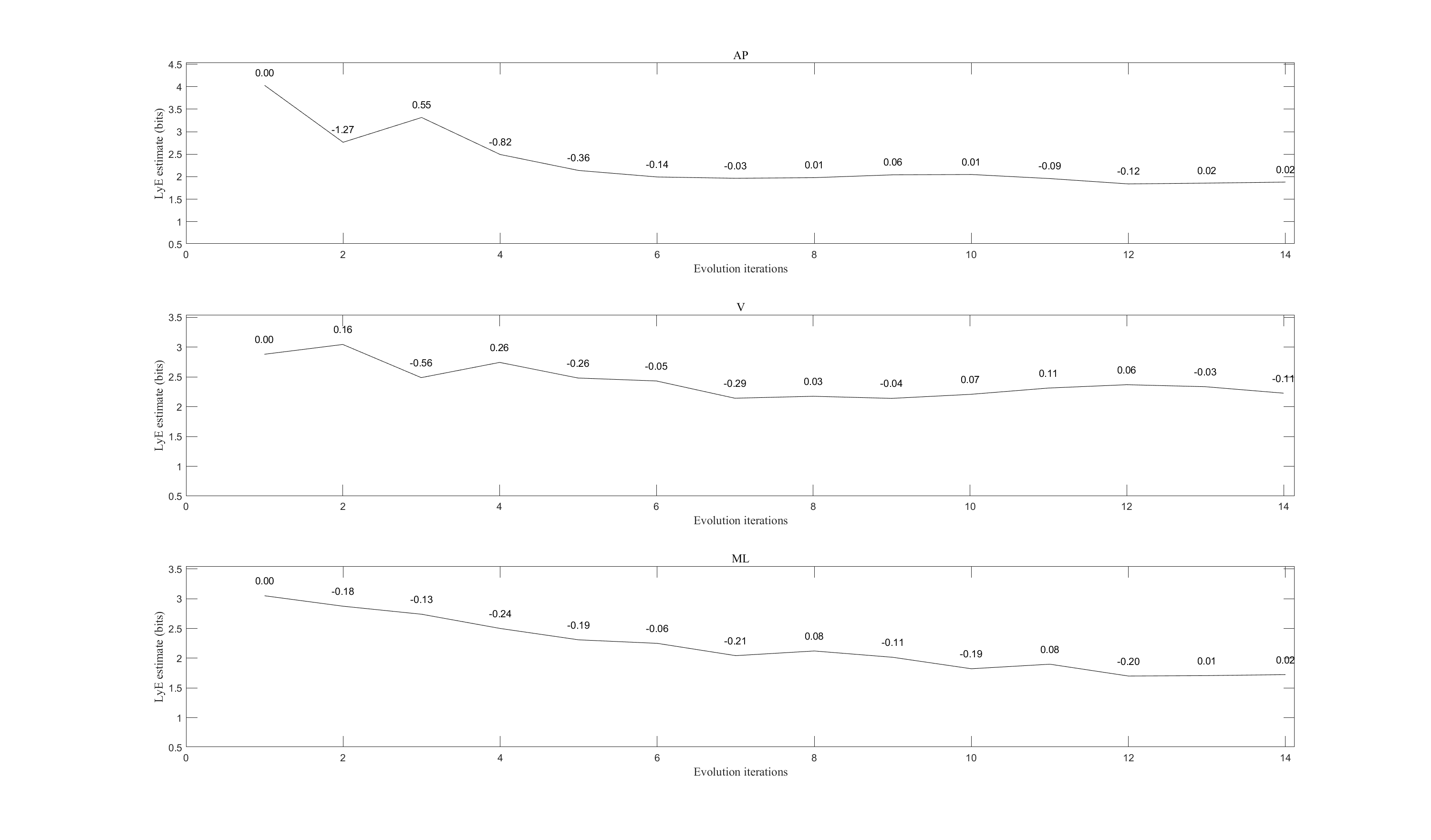

Supplement: Supplementary file 2 — Supplementary Information. [file 41598_2020_79584_MOESM2_ESM.zip › Participant14_trial7.png]

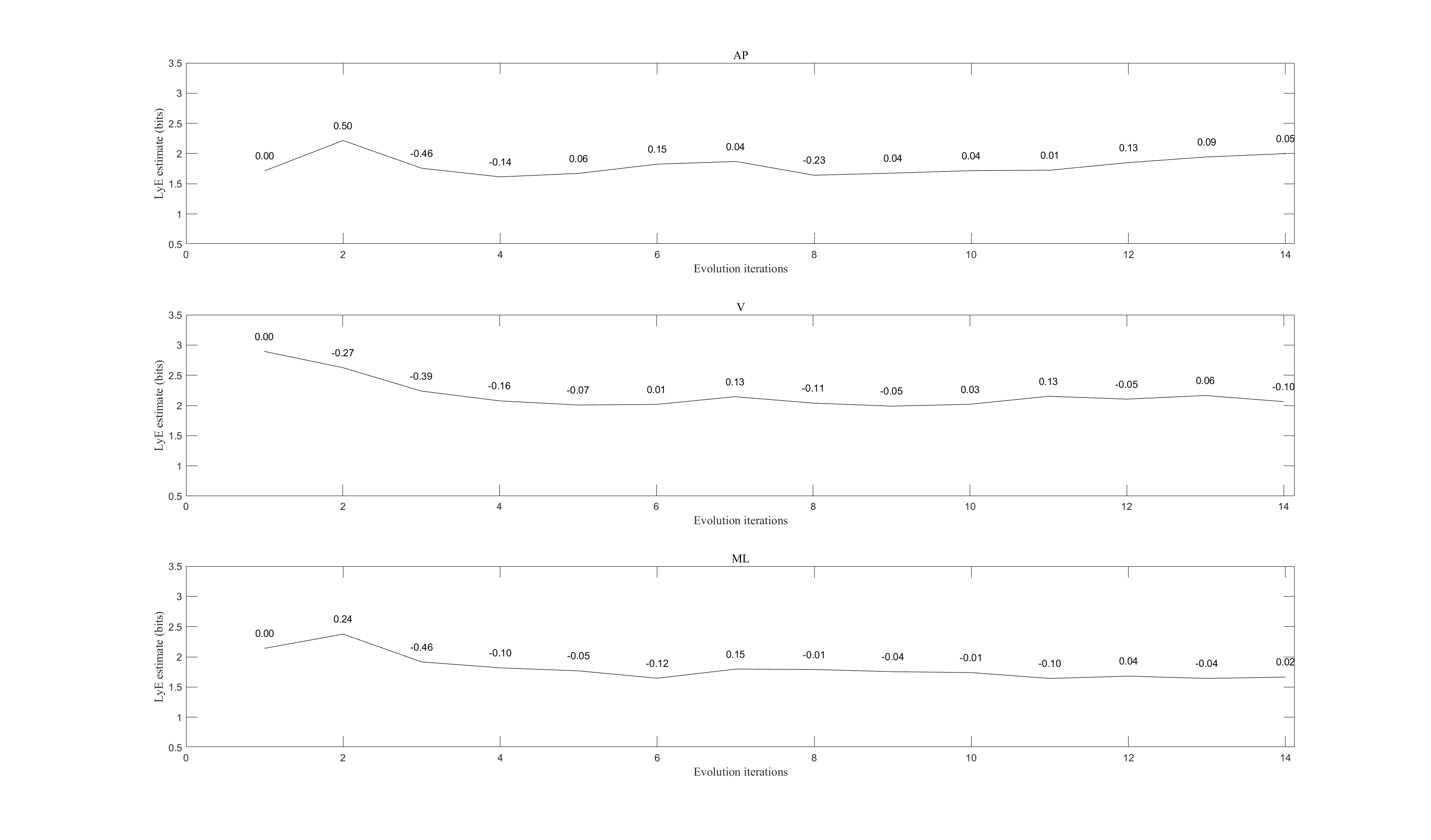

Supplement: Supplementary file 2 — Supplementary Information. [file 41598_2020_79584_MOESM2_ESM.zip › Participant14_trial8.png]

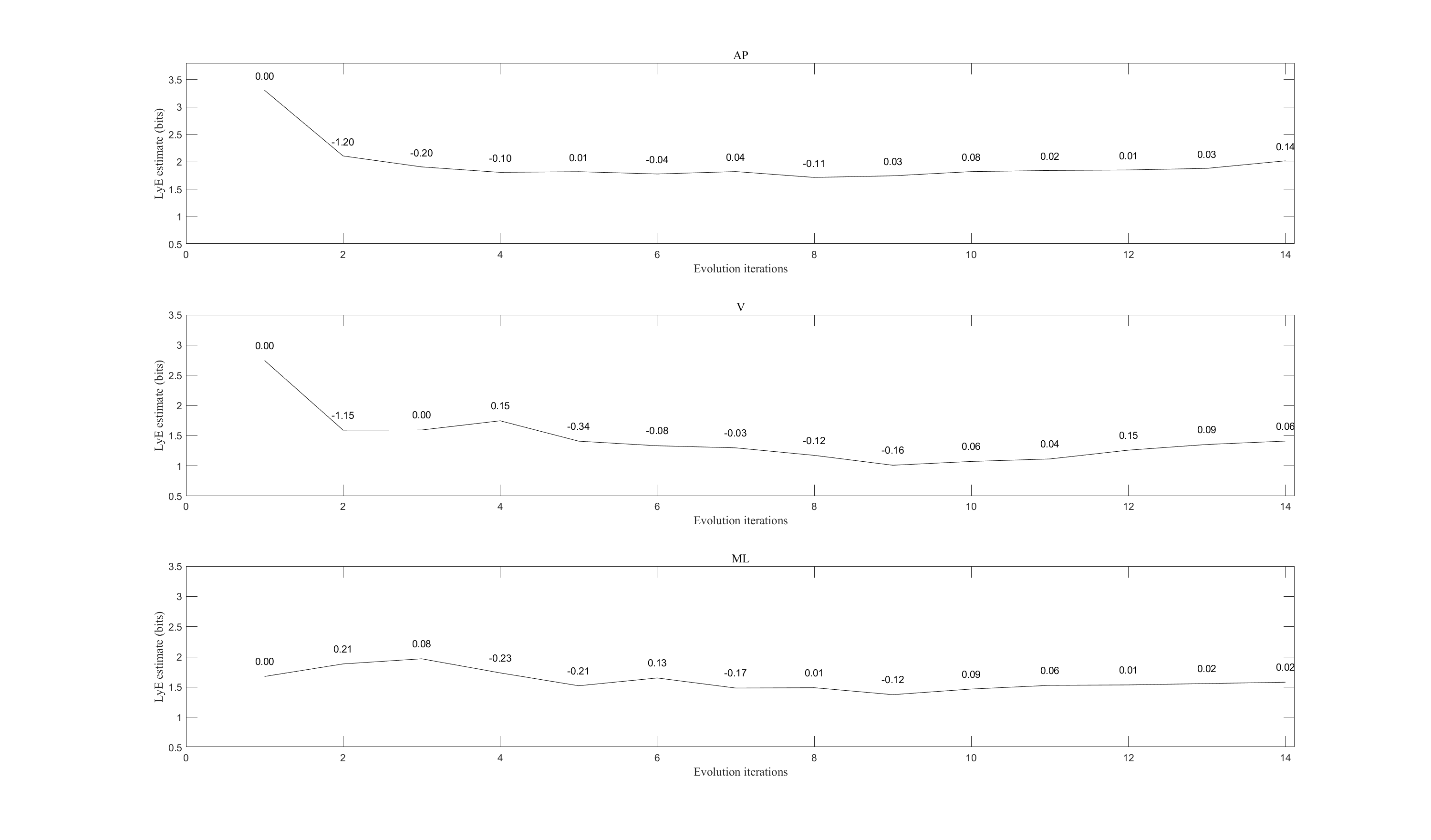

Supplement: Supplementary file 2 — Supplementary Information. [file 41598_2020_79584_MOESM2_ESM.zip › Participant14_trial9.png]

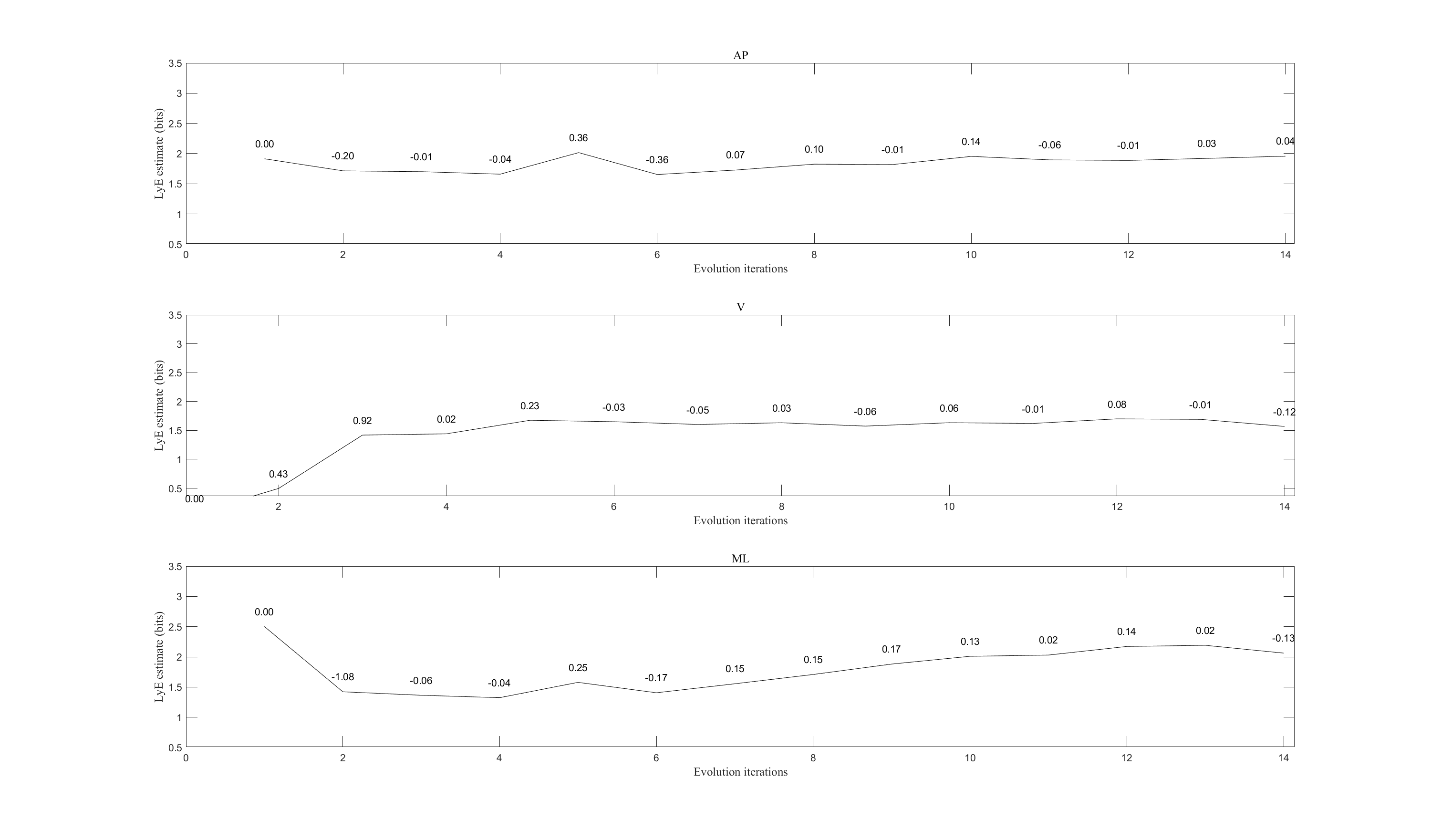

Supplement: Supplementary file 2 — Supplementary Information. [file 41598_2020_79584_MOESM2_ESM.zip › Participant15_trial1.png]

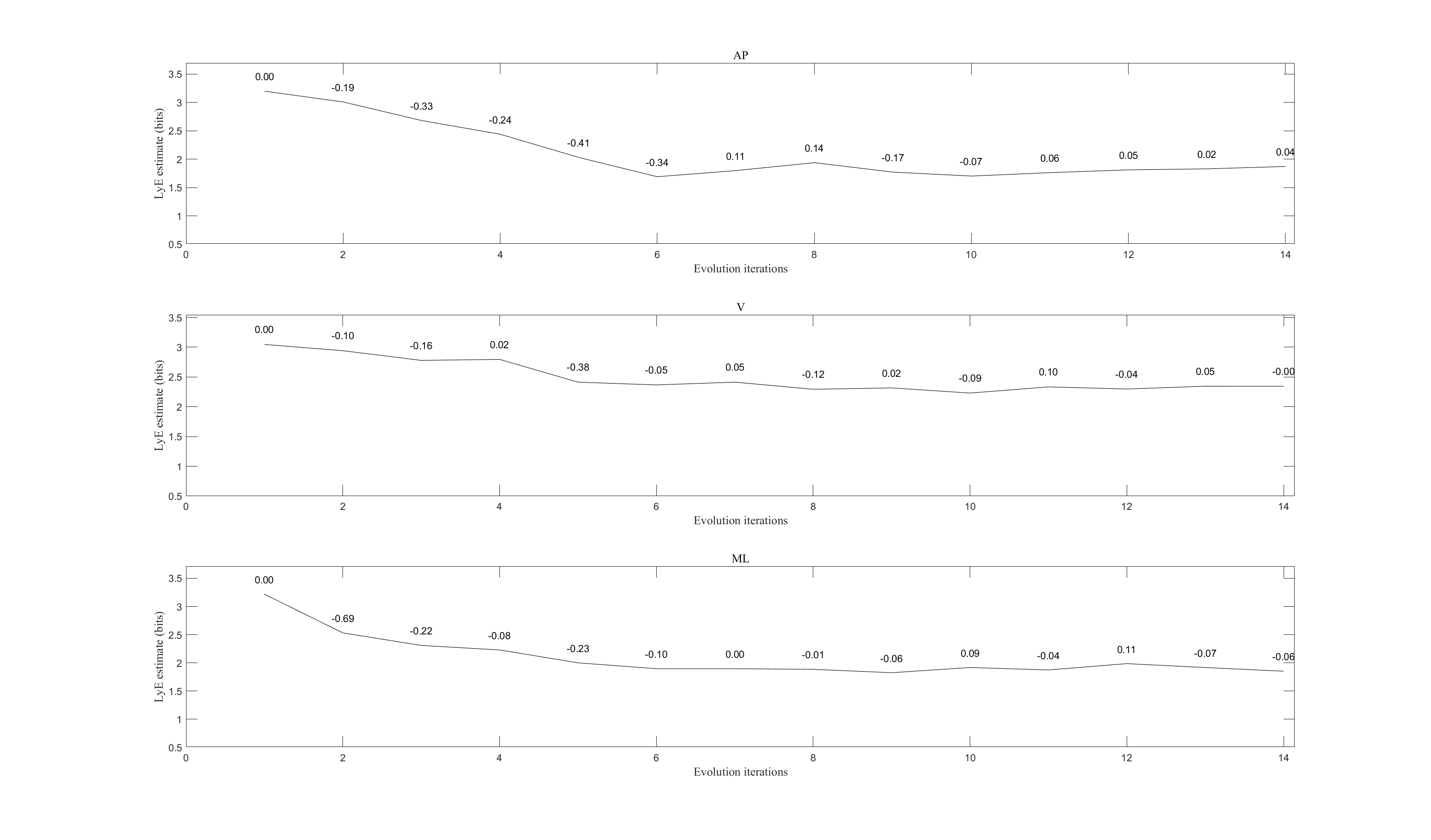

Supplement: Supplementary file 2 — Supplementary Information. [file 41598_2020_79584_MOESM2_ESM.zip › Participant15_trial10.png]

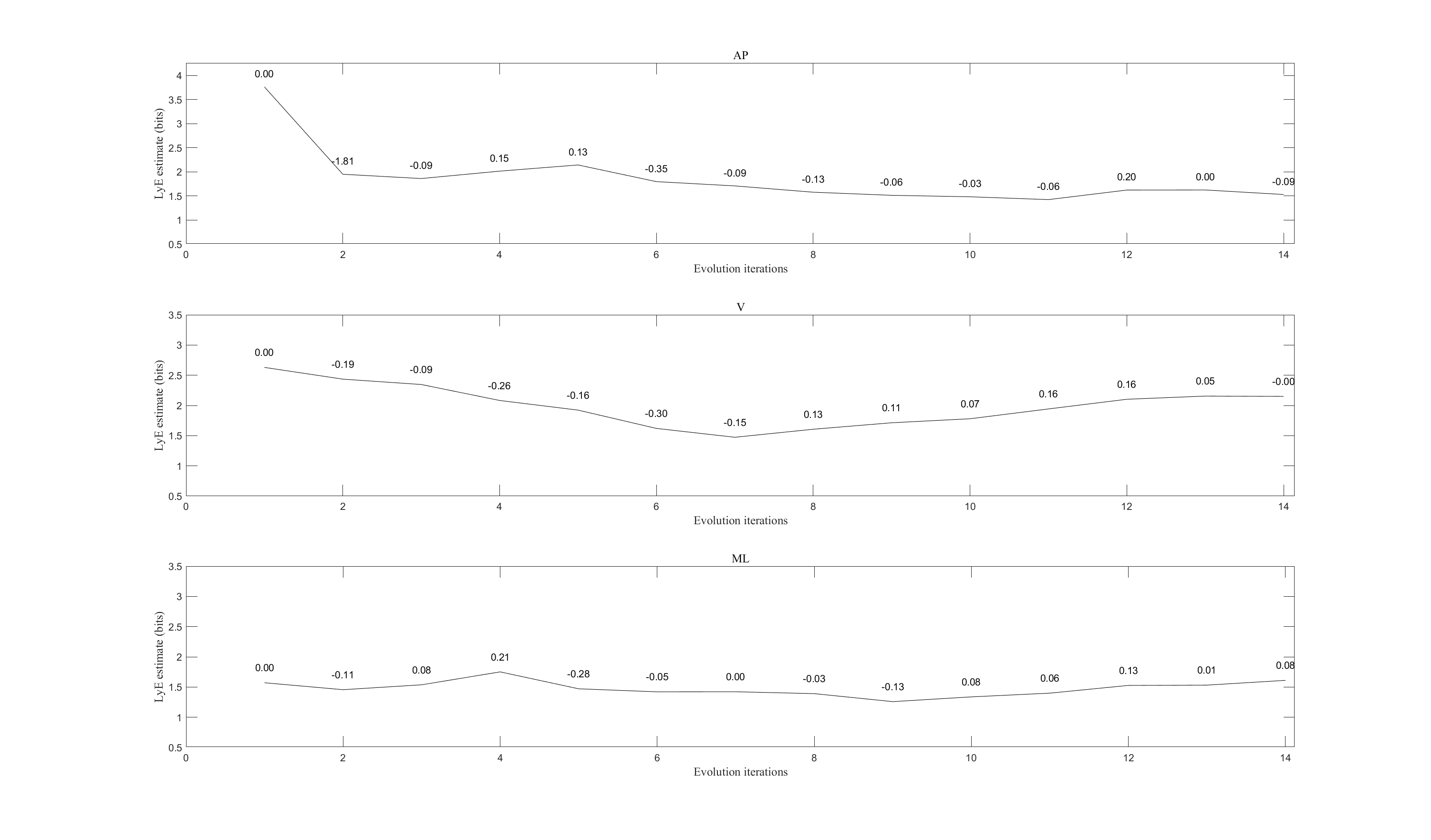

Supplement: Supplementary file 2 — Supplementary Information. [file 41598_2020_79584_MOESM2_ESM.zip › Participant15_trial11.png]

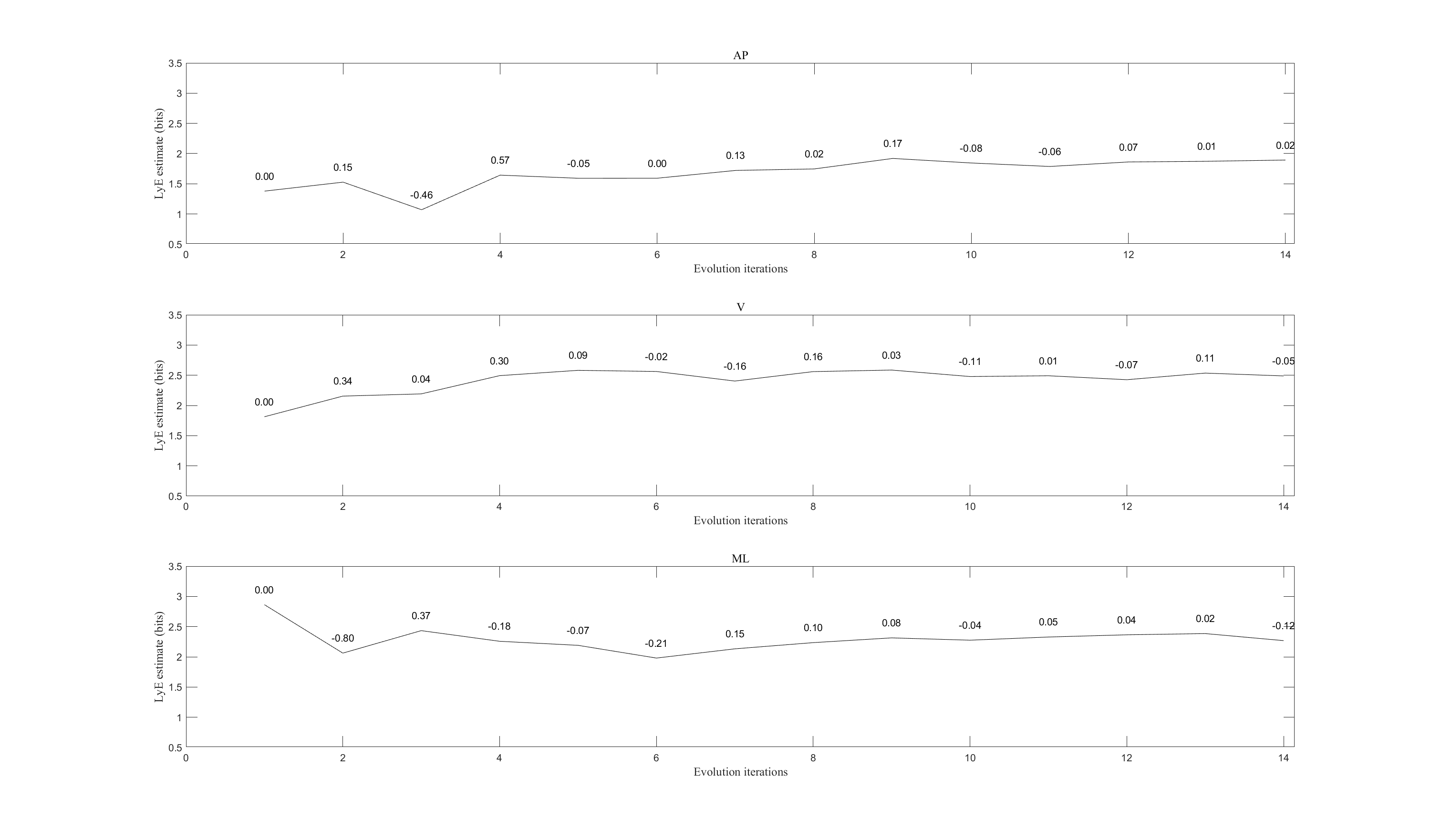

Supplement: Supplementary file 2 — Supplementary Information. [file 41598_2020_79584_MOESM2_ESM.zip › Participant15_trial12.png]

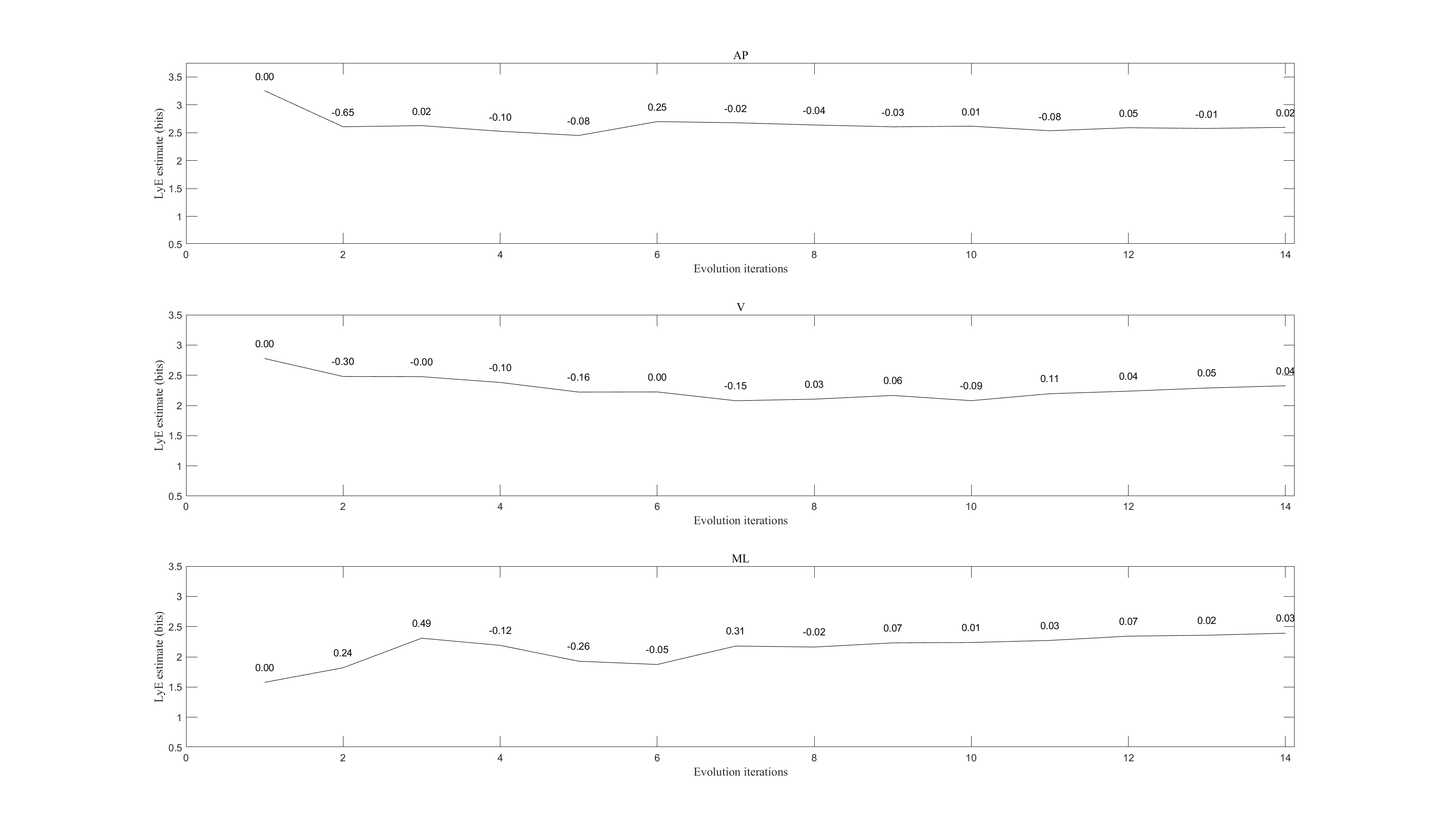

Supplement: Supplementary file 2 — Supplementary Information. [file 41598_2020_79584_MOESM2_ESM.zip › Participant15_trial2.png]

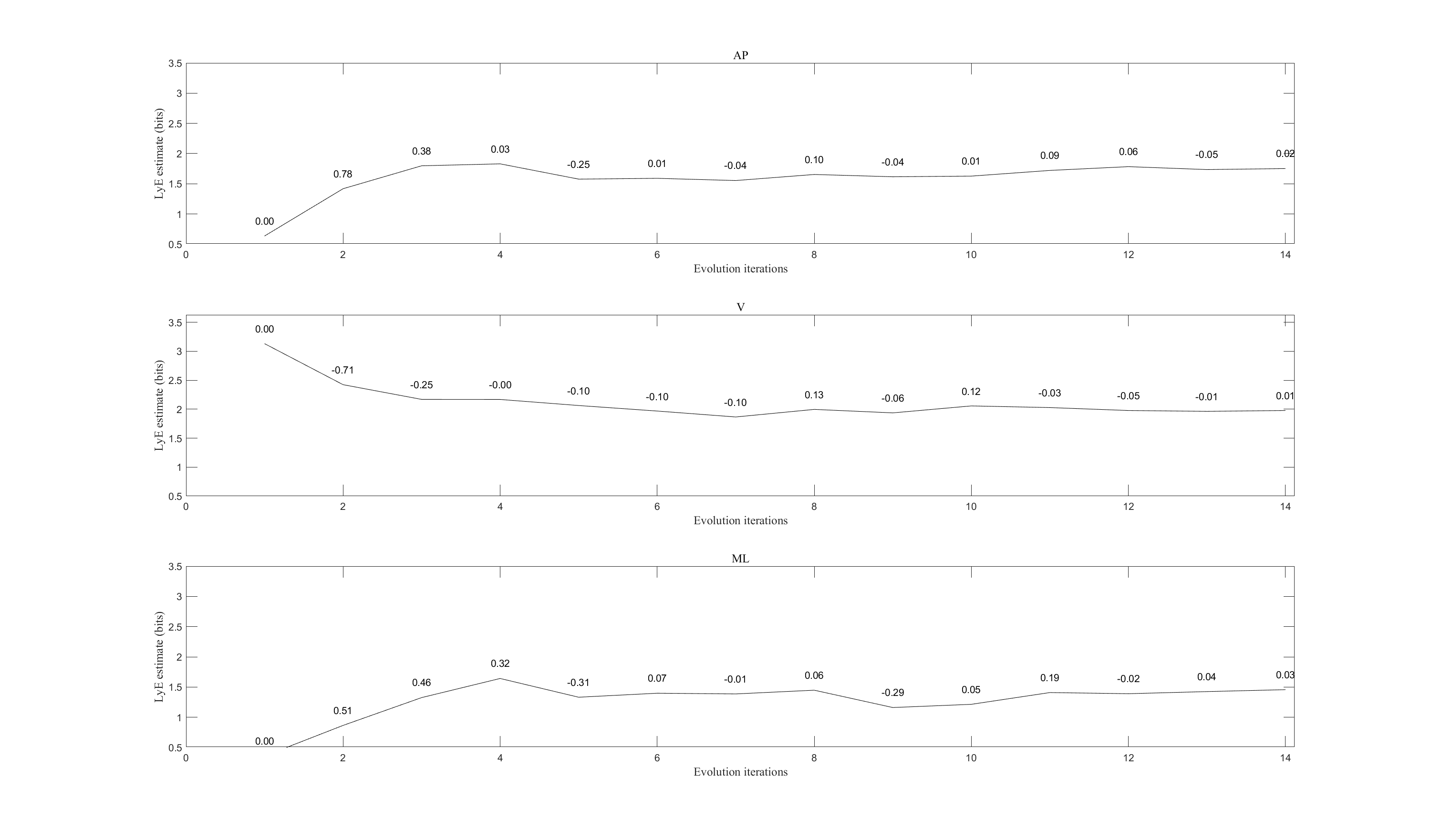

Supplement: Supplementary file 2 — Supplementary Information. [file 41598_2020_79584_MOESM2_ESM.zip › Participant15_trial3.png]

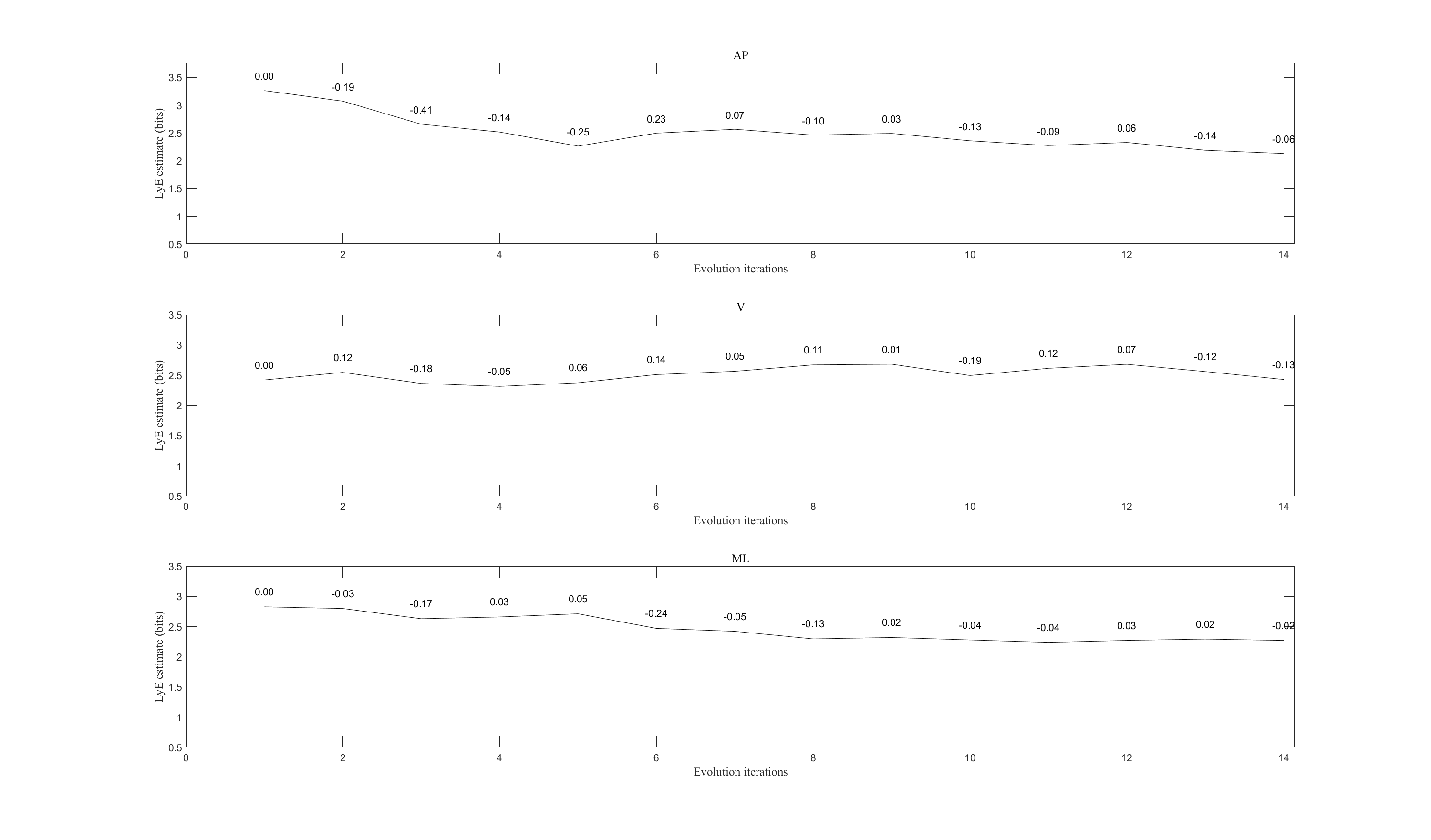

Supplement: Supplementary file 2 — Supplementary Information. [file 41598_2020_79584_MOESM2_ESM.zip › Participant15_trial4.png]

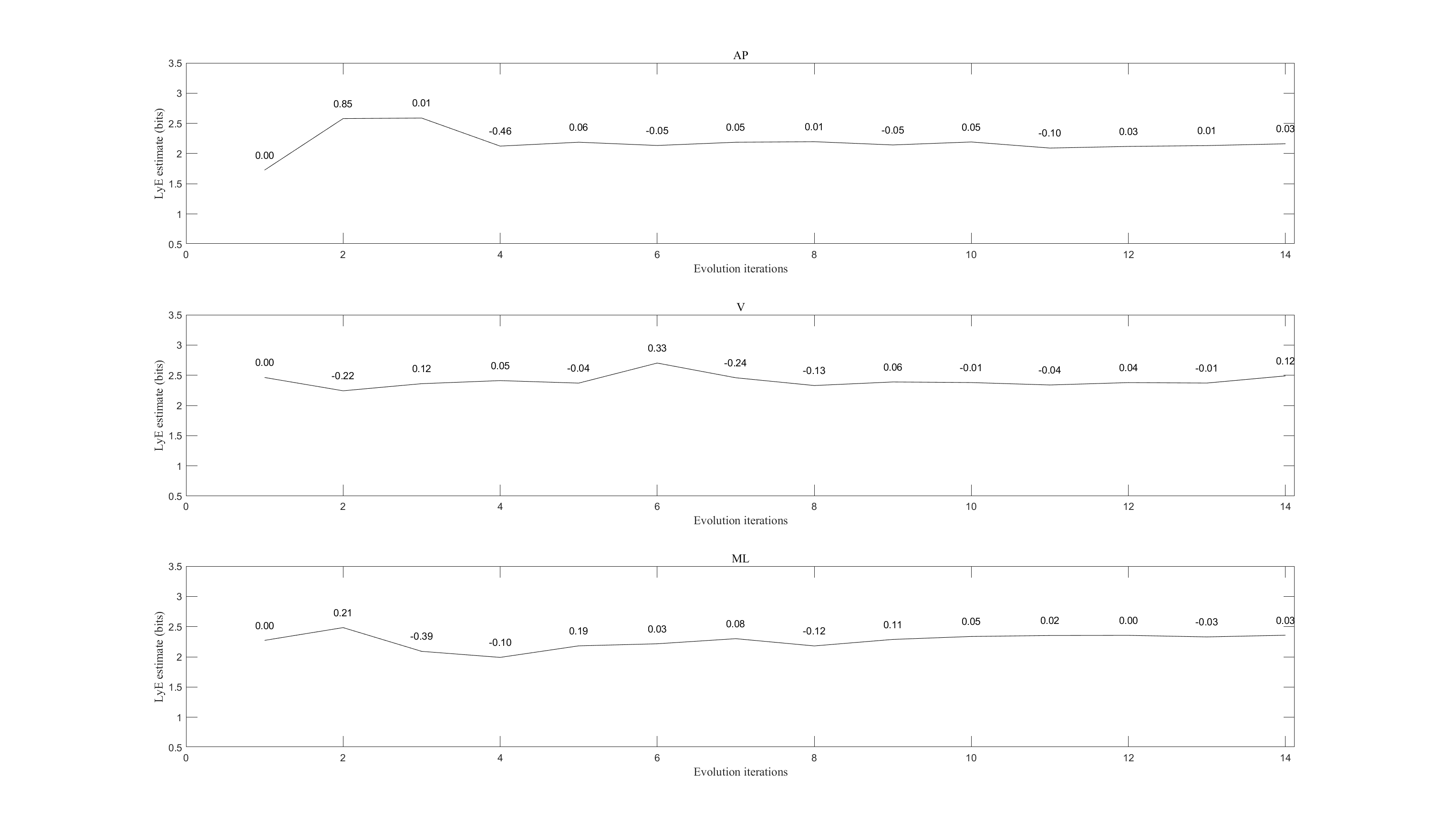

Supplement: Supplementary file 2 — Supplementary Information. [file 41598_2020_79584_MOESM2_ESM.zip › Participant15_trial5.png]

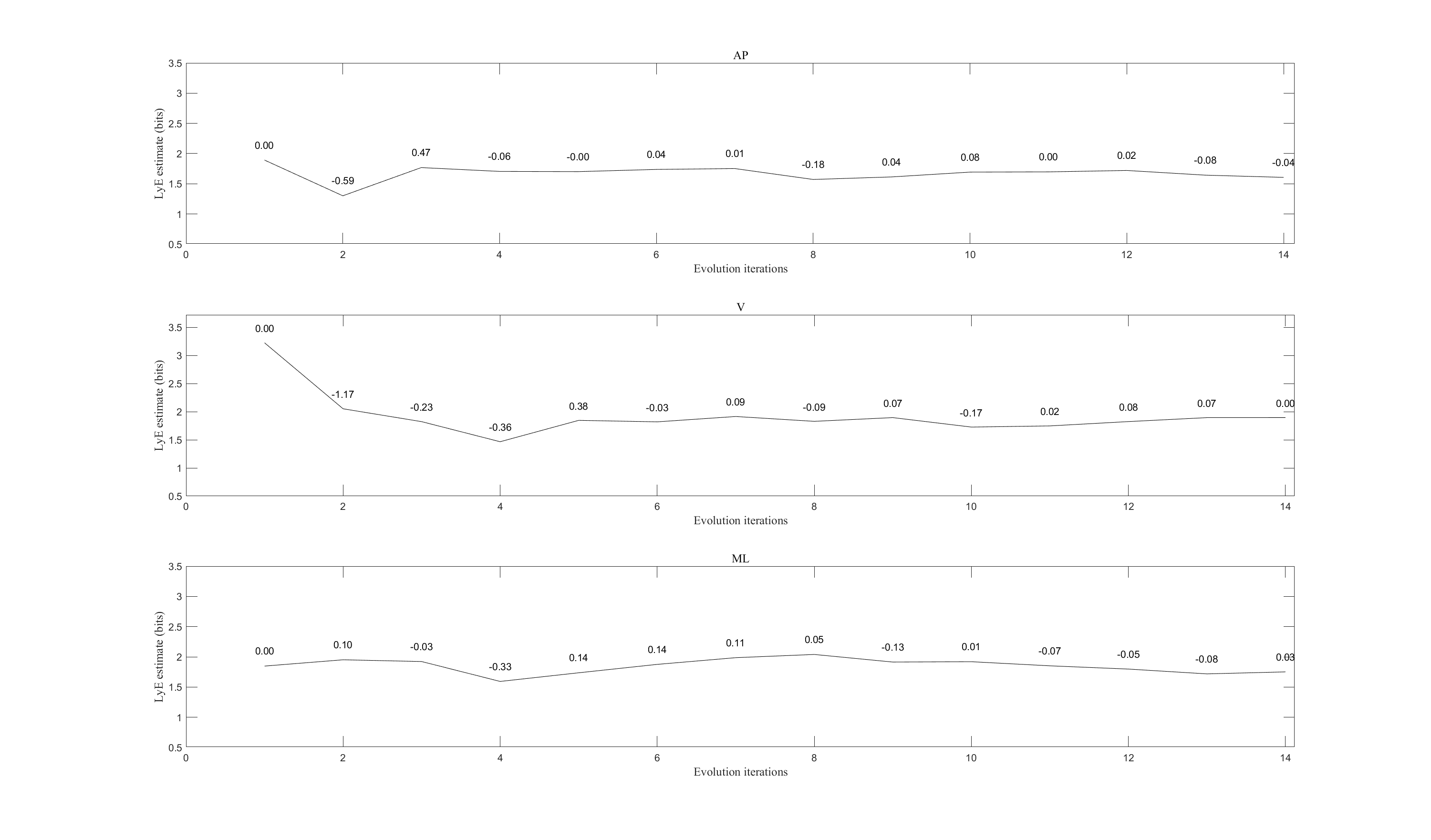

Supplement: Supplementary file 2 — Supplementary Information. [file 41598_2020_79584_MOESM2_ESM.zip › Participant15_trial6.png]

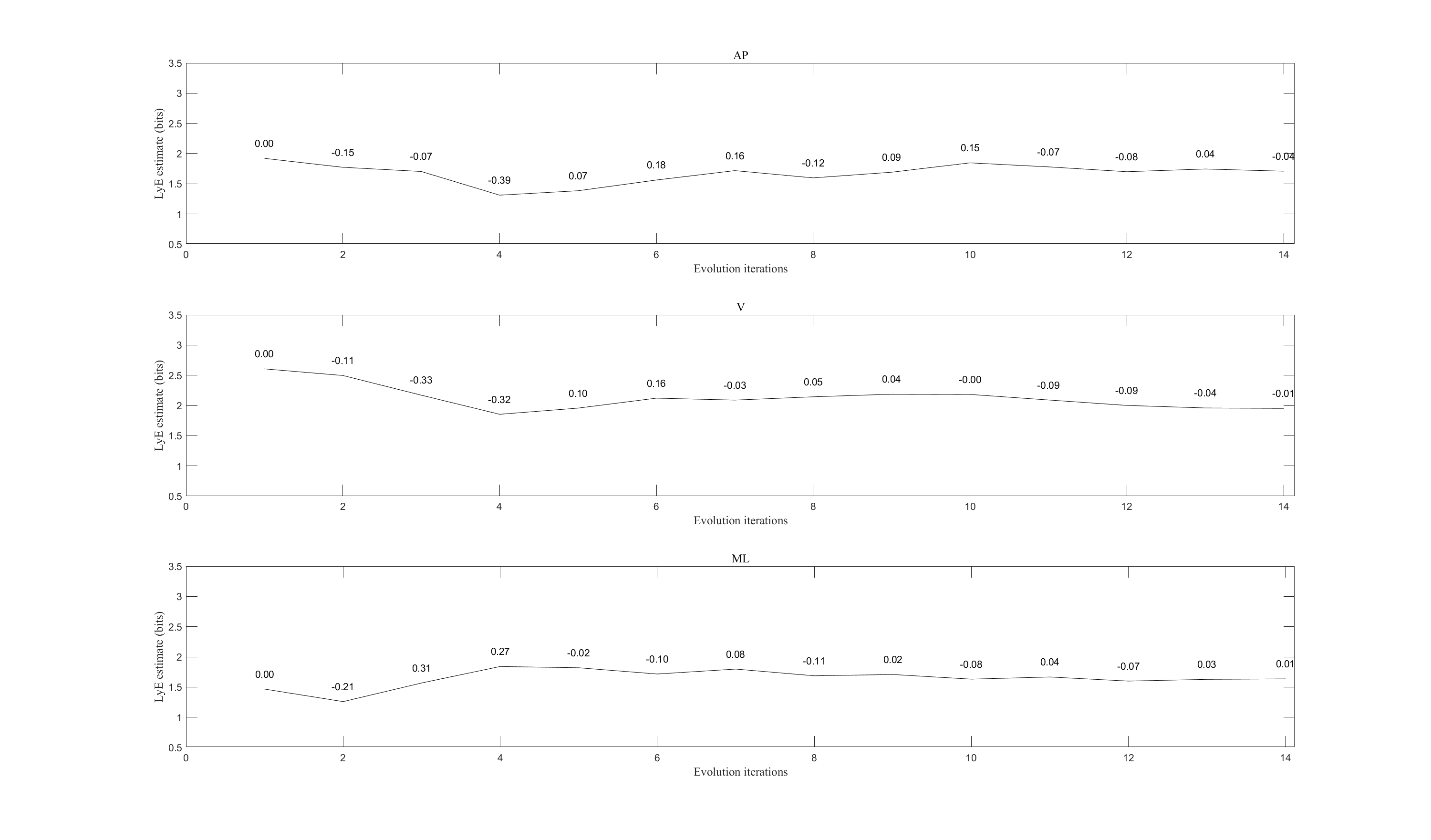

Supplement: Supplementary file 2 — Supplementary Information. [file 41598_2020_79584_MOESM2_ESM.zip › Participant15_trial7.png]

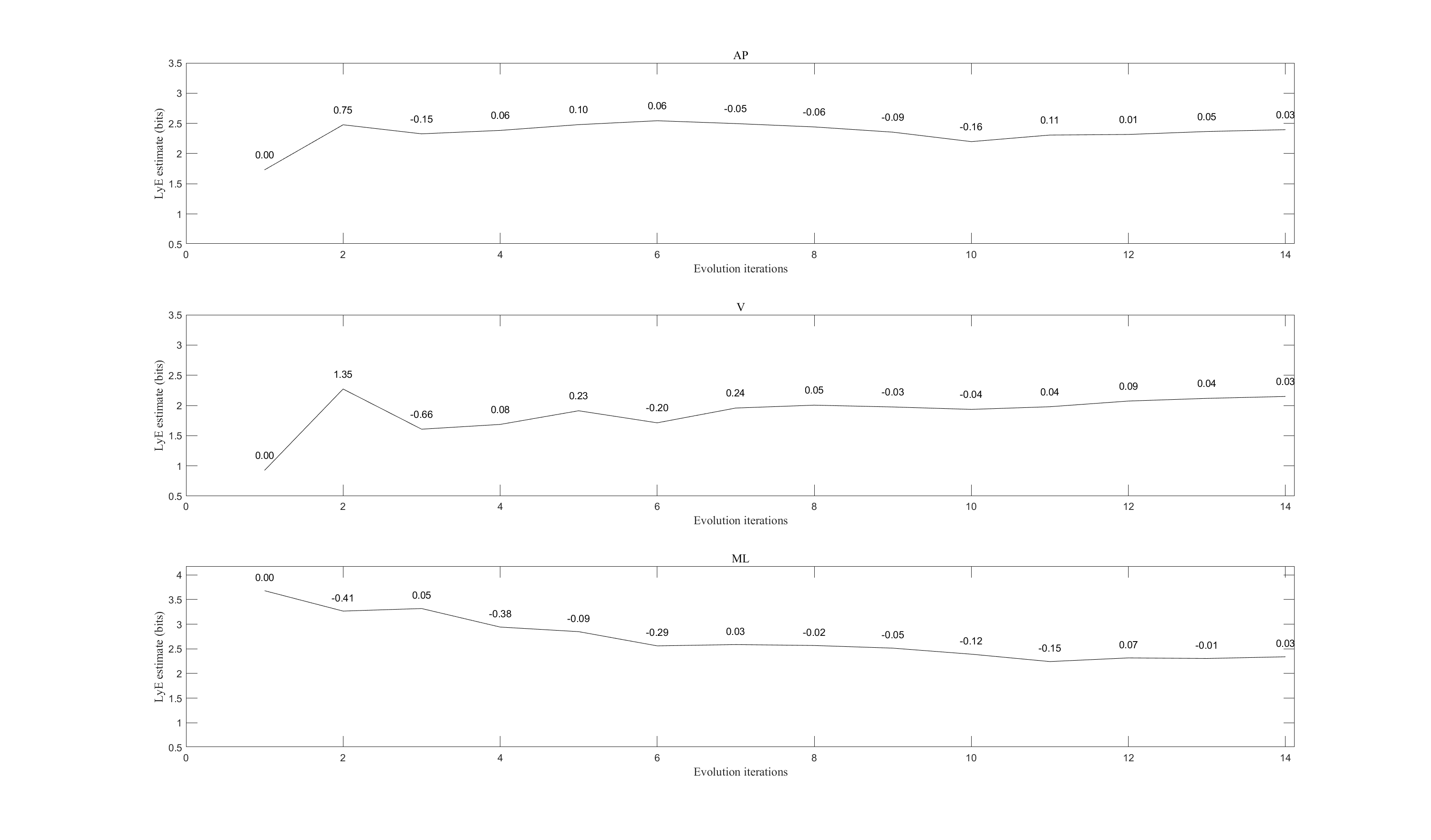

Supplement: Supplementary file 2 — Supplementary Information. [file 41598_2020_79584_MOESM2_ESM.zip › Participant15_trial8.png]

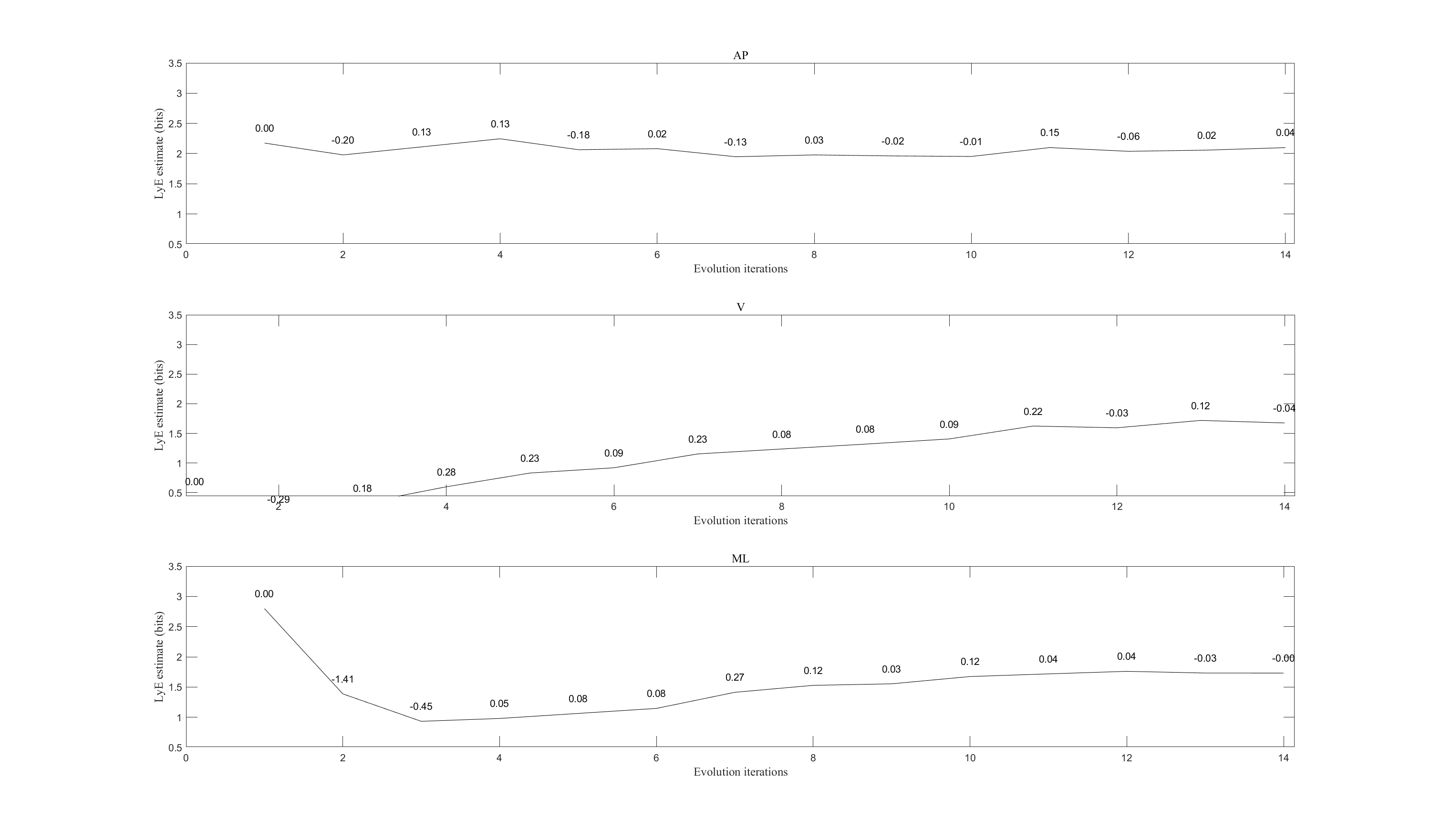

Supplement: Supplementary file 2 — Supplementary Information. [file 41598_2020_79584_MOESM2_ESM.zip › Participant15_trial9.png]

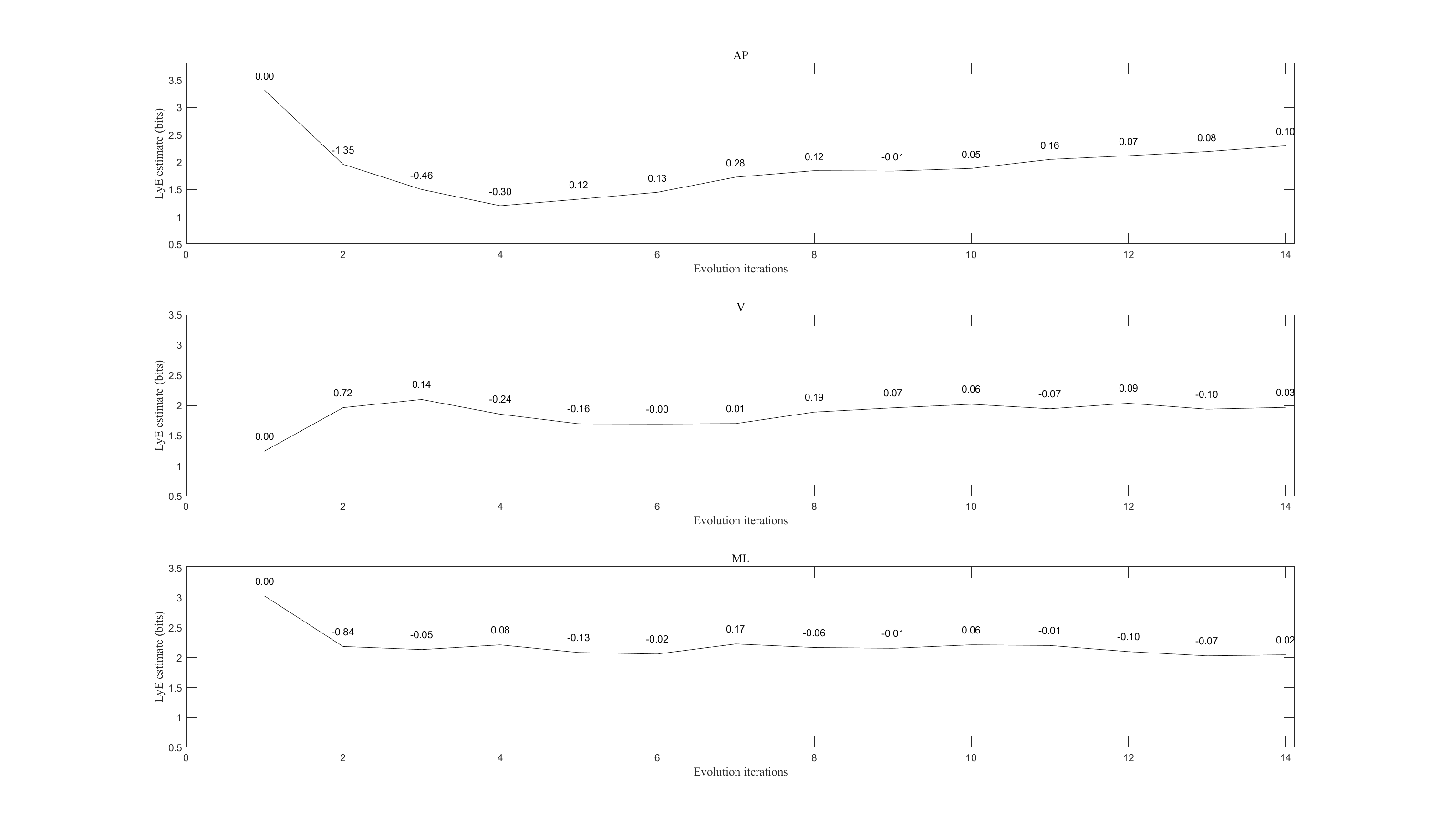

Supplement: Supplementary file 2 — Supplementary Information. [file 41598_2020_79584_MOESM2_ESM.zip › Participant16_trial1.png]

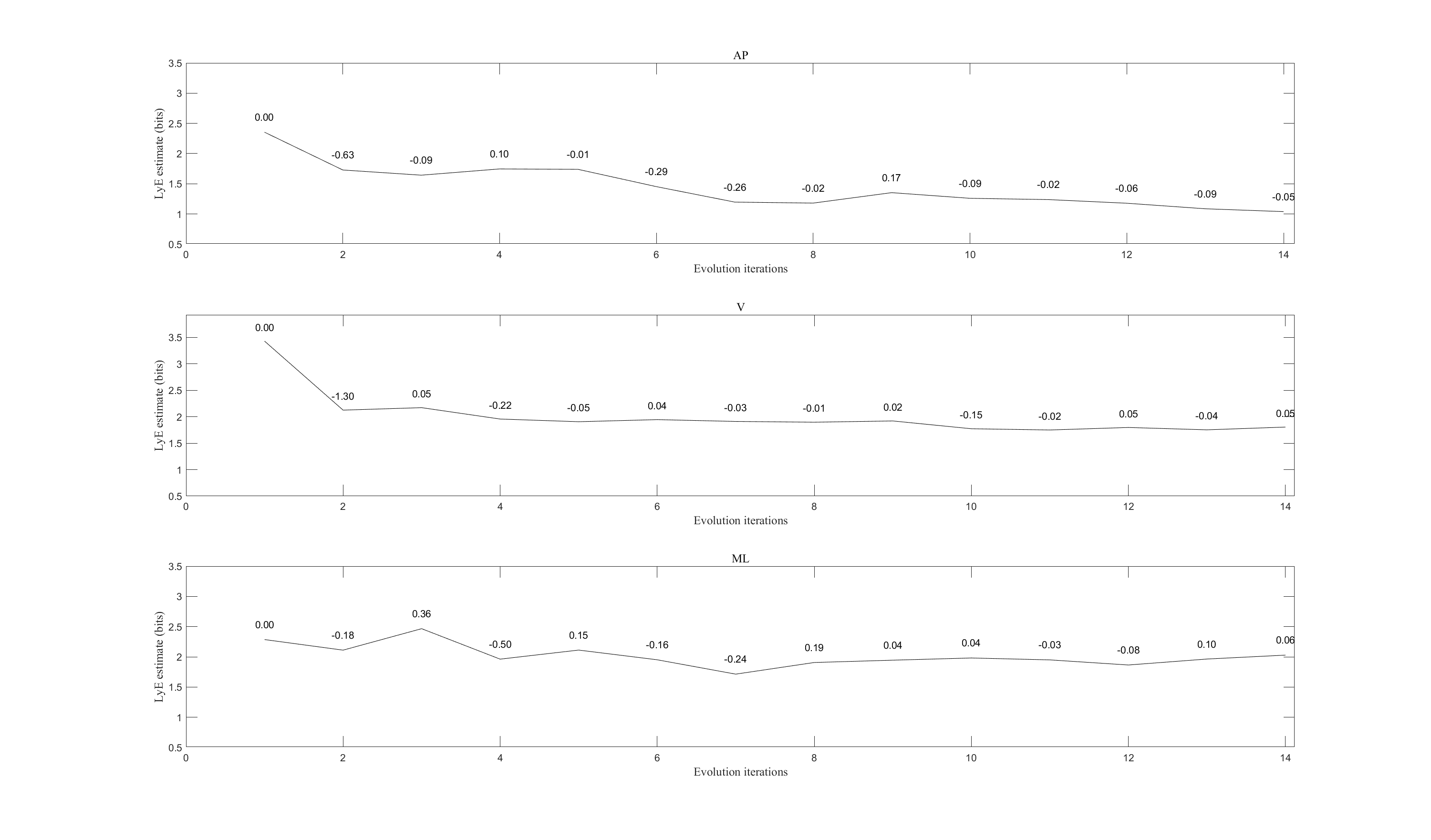

Supplement: Supplementary file 2 — Supplementary Information. [file 41598_2020_79584_MOESM2_ESM.zip › Participant16_trial10.png]

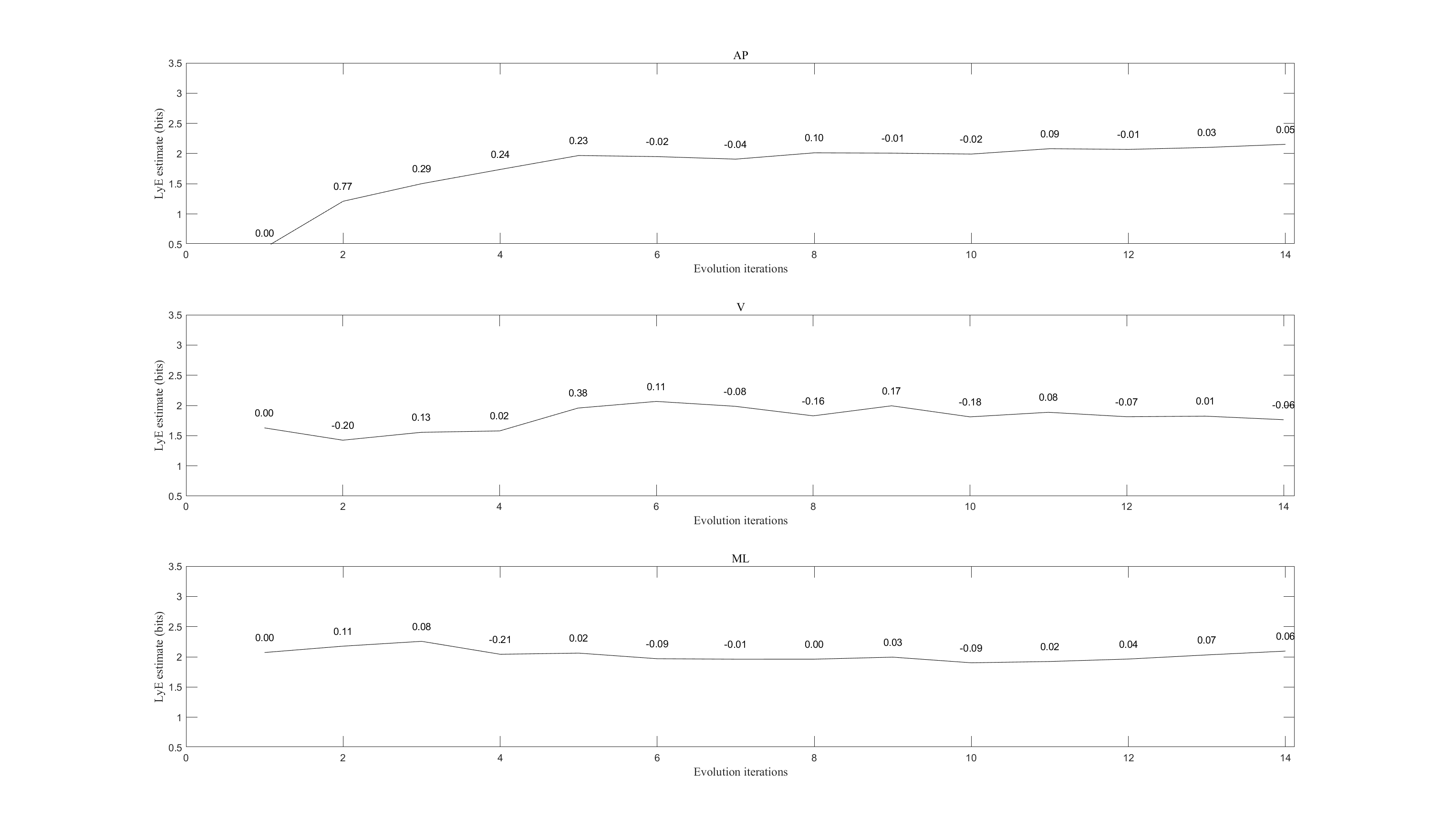

Supplement: Supplementary file 2 — Supplementary Information. [file 41598_2020_79584_MOESM2_ESM.zip › Participant16_trial11.png]

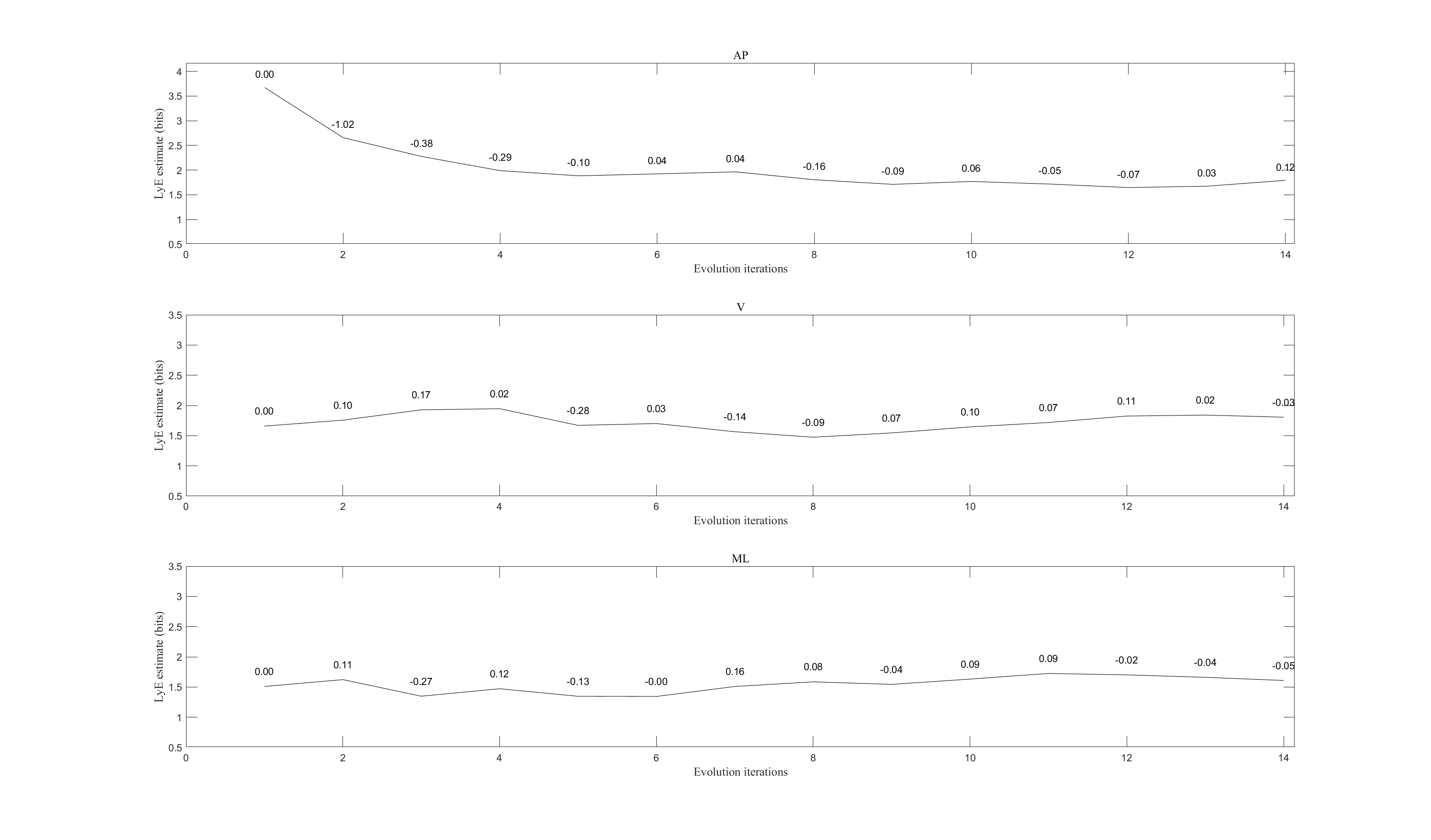

Supplement: Supplementary file 2 — Supplementary Information. [file 41598_2020_79584_MOESM2_ESM.zip › Participant16_trial12.png]

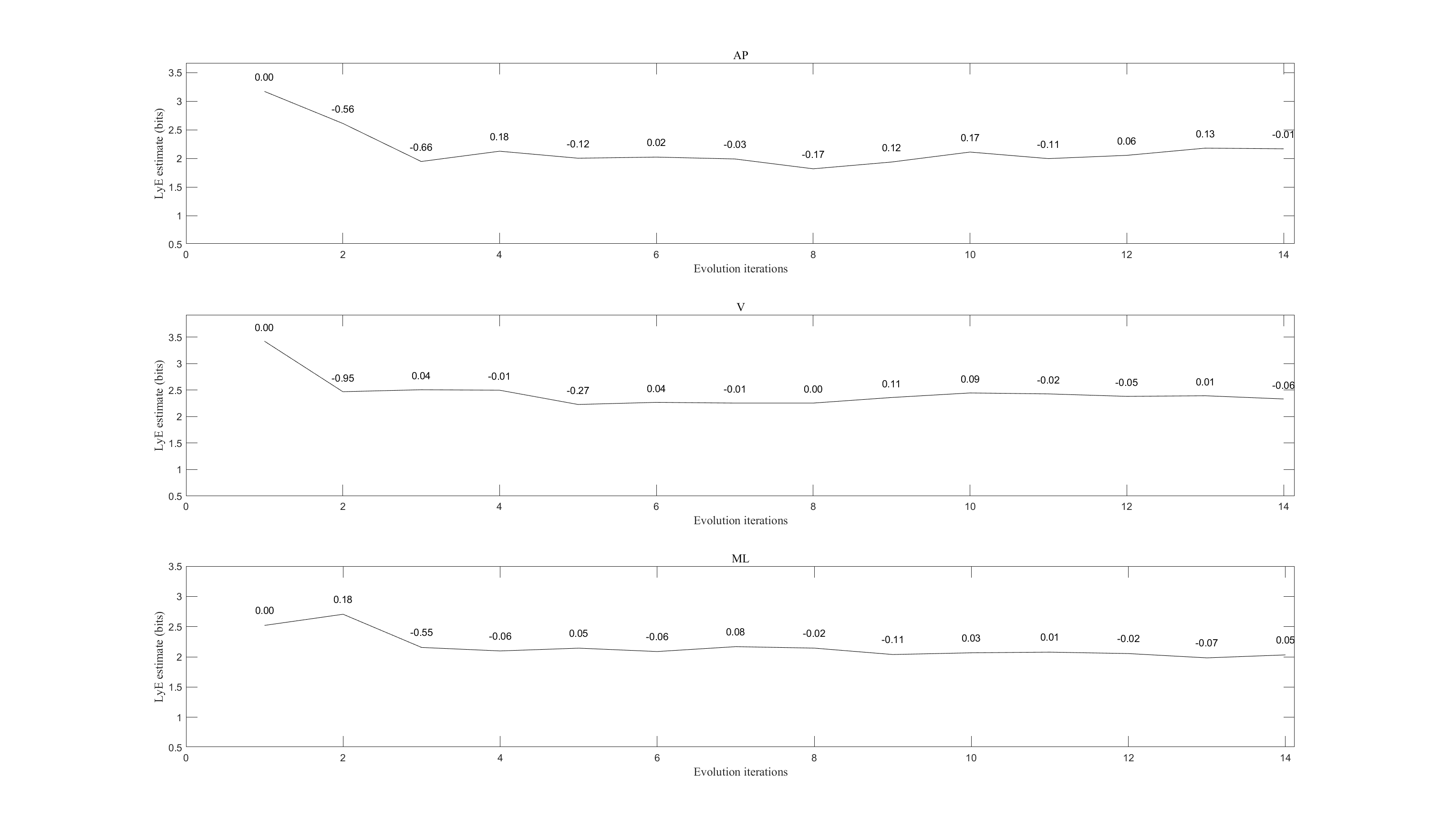

Supplement: Supplementary file 2 — Supplementary Information. [file 41598_2020_79584_MOESM2_ESM.zip › Participant16_trial2.png]

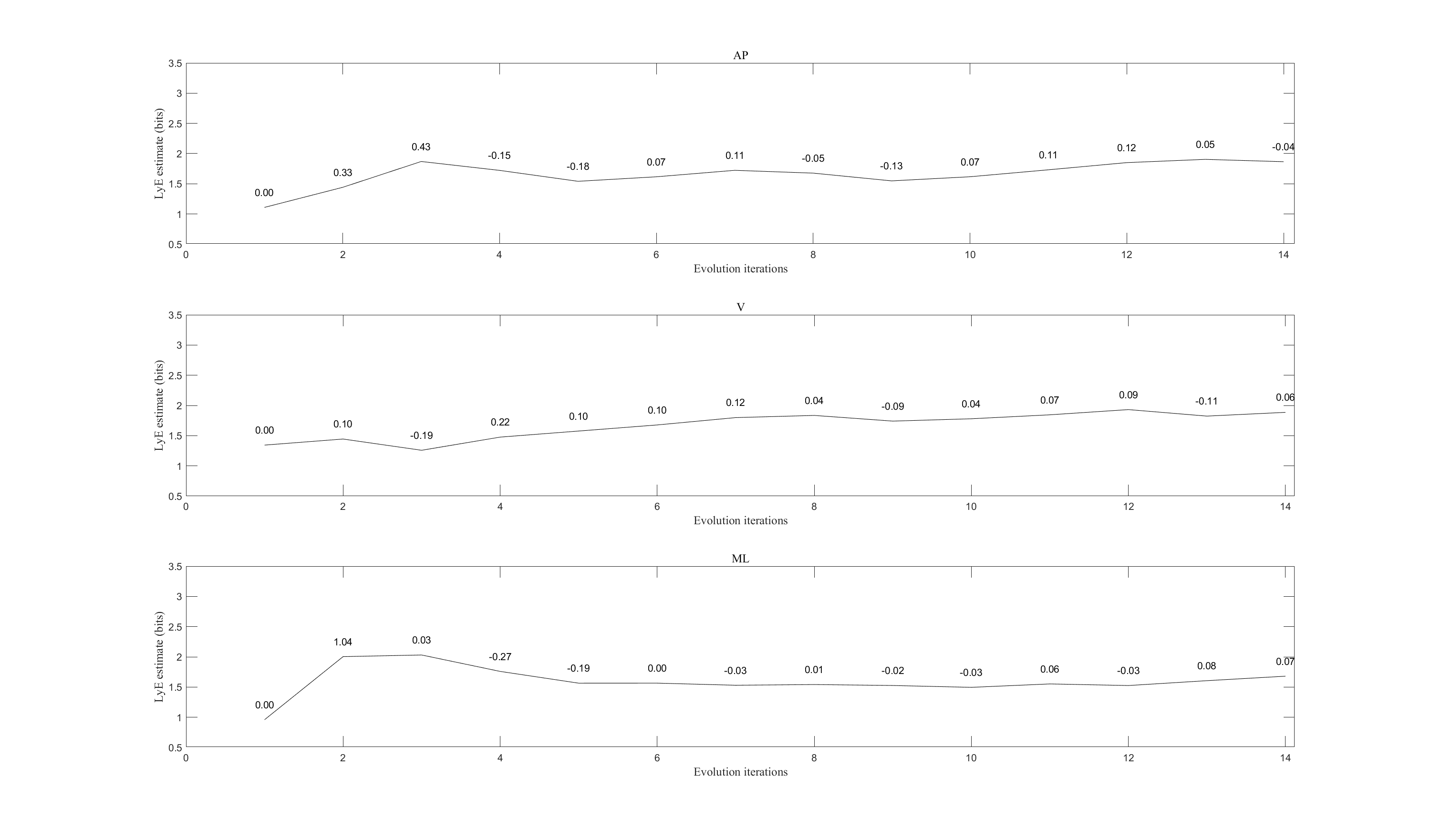

Supplement: Supplementary file 2 — Supplementary Information. [file 41598_2020_79584_MOESM2_ESM.zip › Participant16_trial3.png]

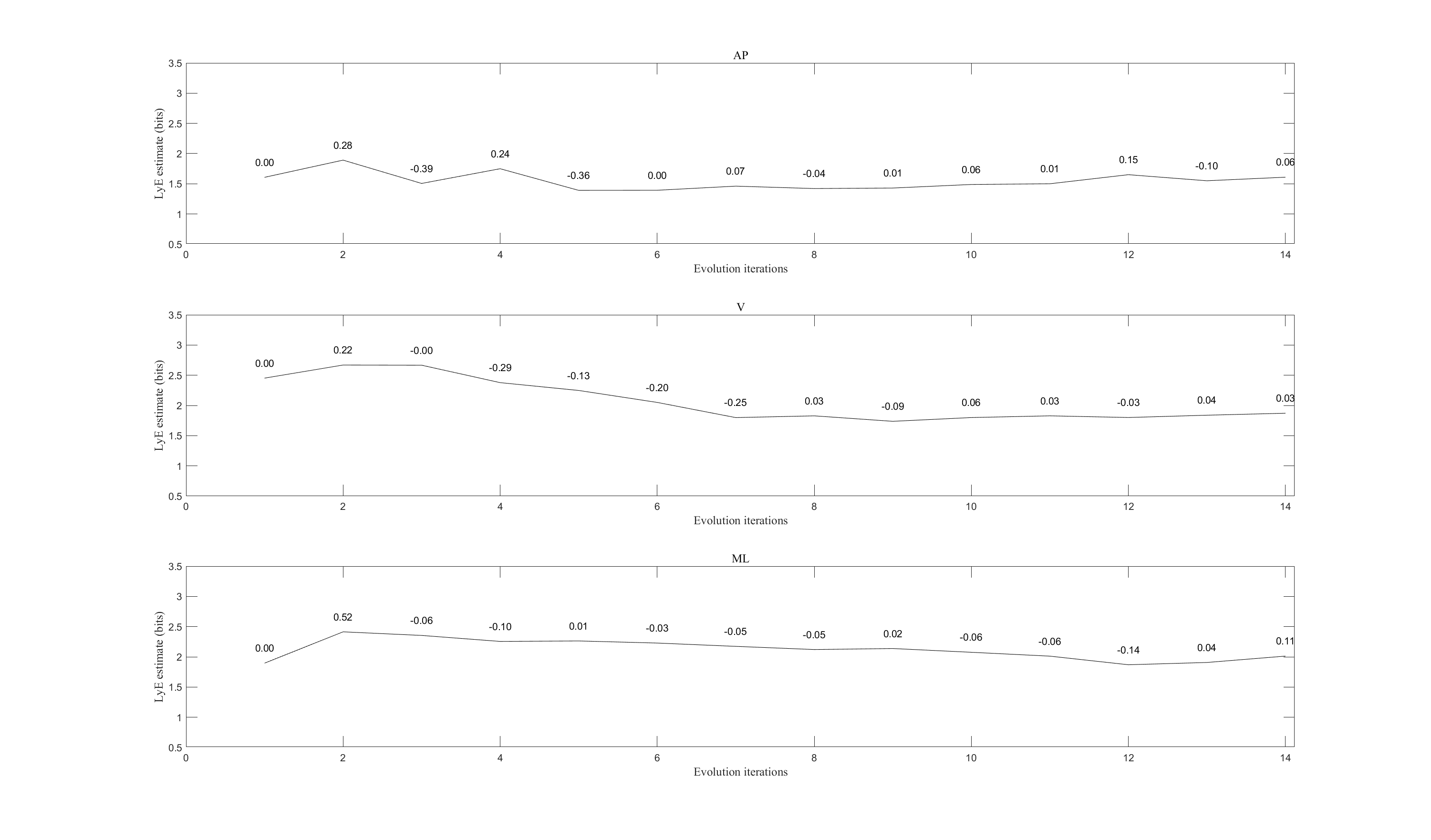

Supplement: Supplementary file 2 — Supplementary Information. [file 41598_2020_79584_MOESM2_ESM.zip › Participant16_trial4.png]

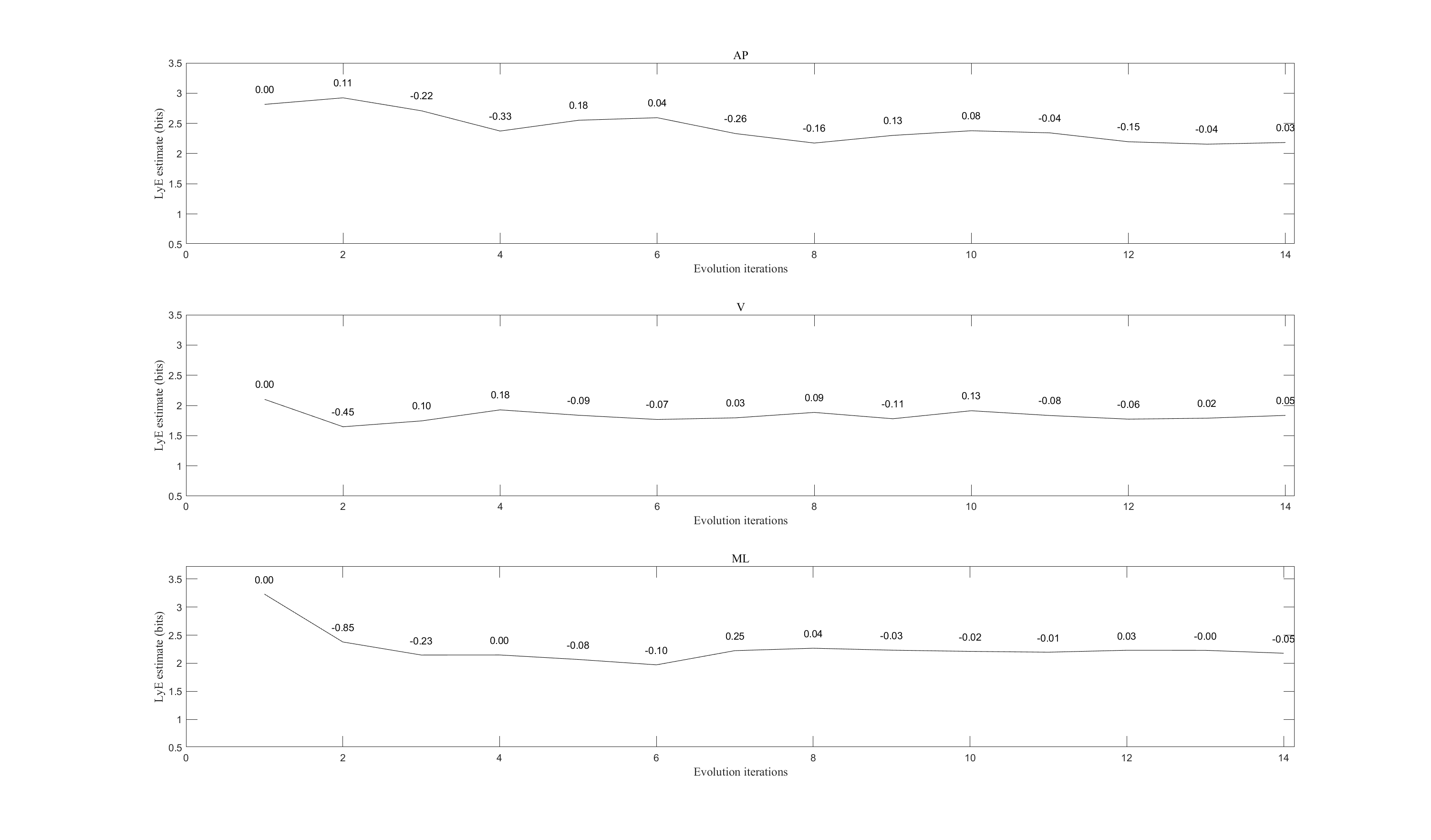

Supplement: Supplementary file 2 — Supplementary Information. [file 41598_2020_79584_MOESM2_ESM.zip › Participant16_trial5.png]

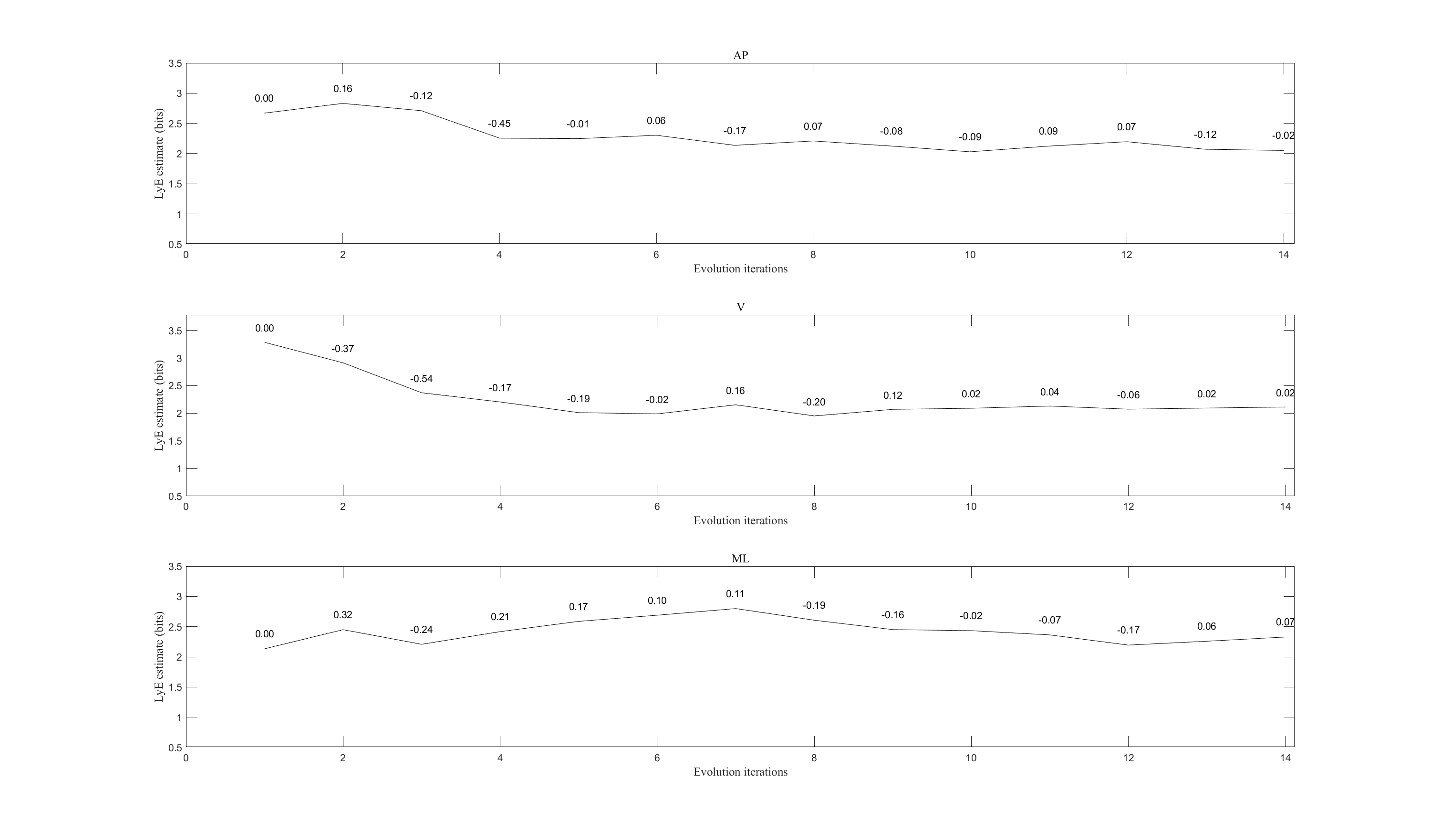

Supplement: Supplementary file 2 — Supplementary Information. [file 41598_2020_79584_MOESM2_ESM.zip › Participant16_trial6.png]

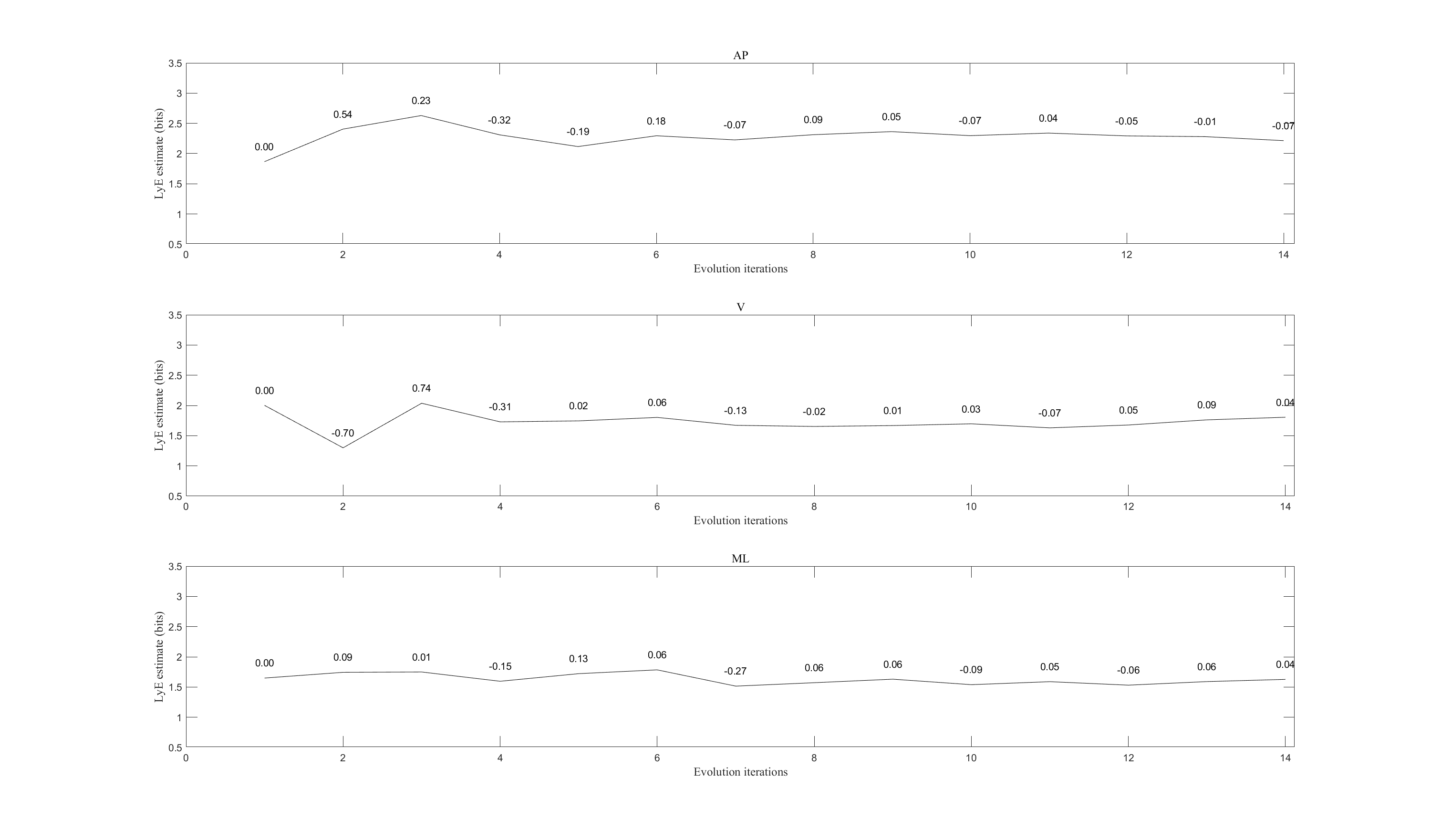

Supplement: Supplementary file 2 — Supplementary Information. [file 41598_2020_79584_MOESM2_ESM.zip › Participant16_trial7.png]

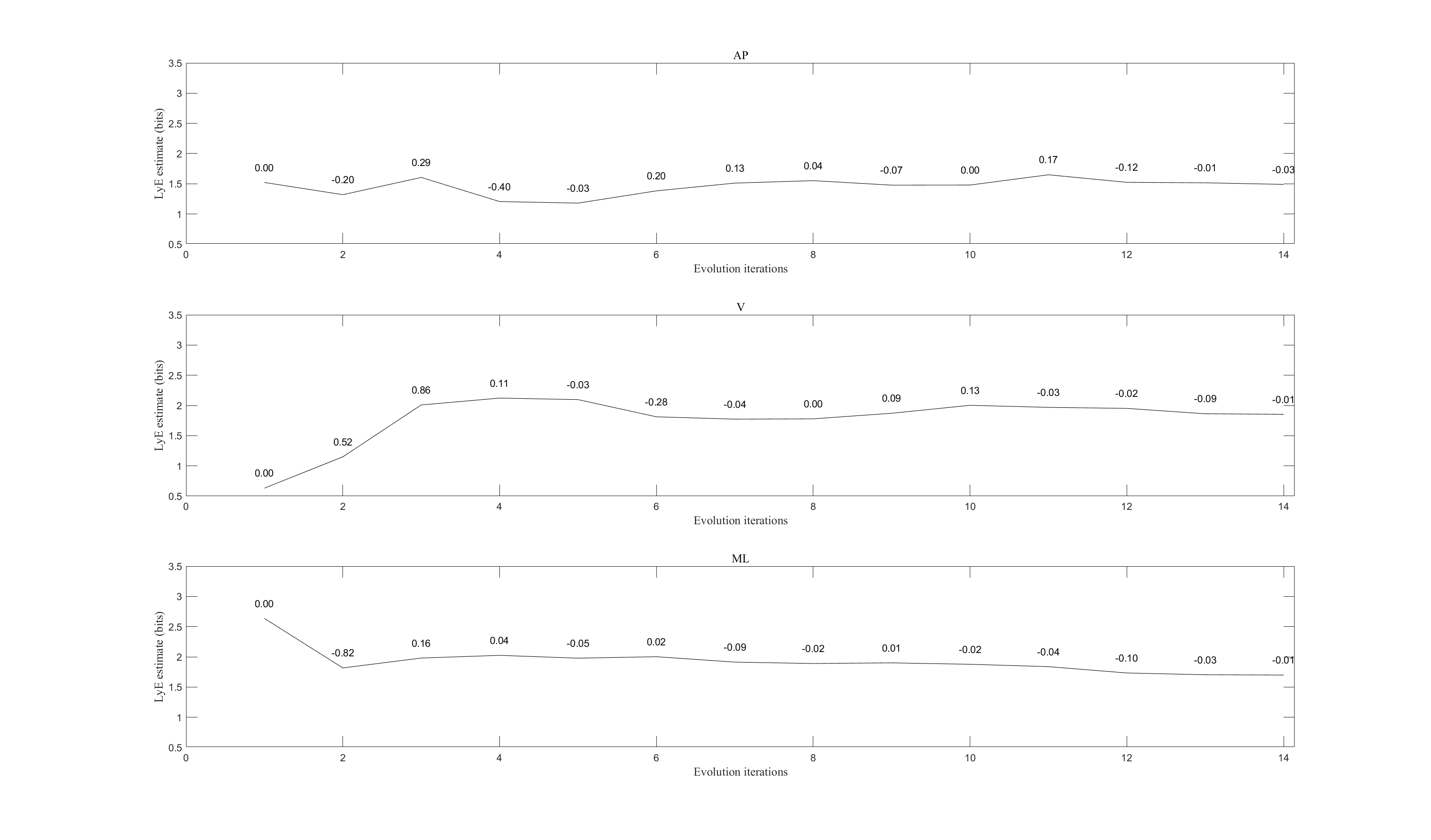

Supplement: Supplementary file 2 — Supplementary Information. [file 41598_2020_79584_MOESM2_ESM.zip › Participant16_trial8.png]

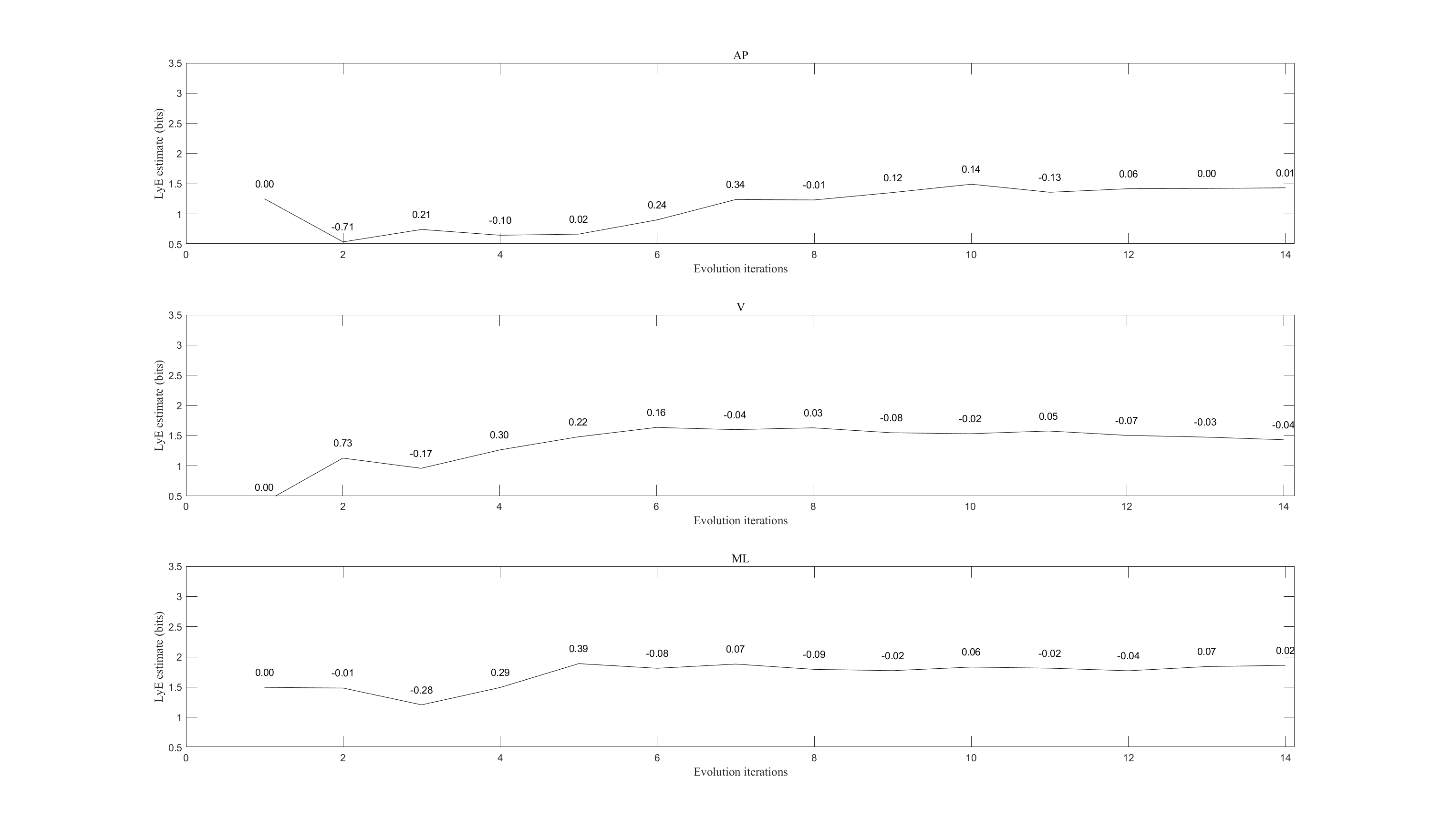

Supplement: Supplementary file 2 — Supplementary Information. [file 41598_2020_79584_MOESM2_ESM.zip › Participant16_trial9.png]

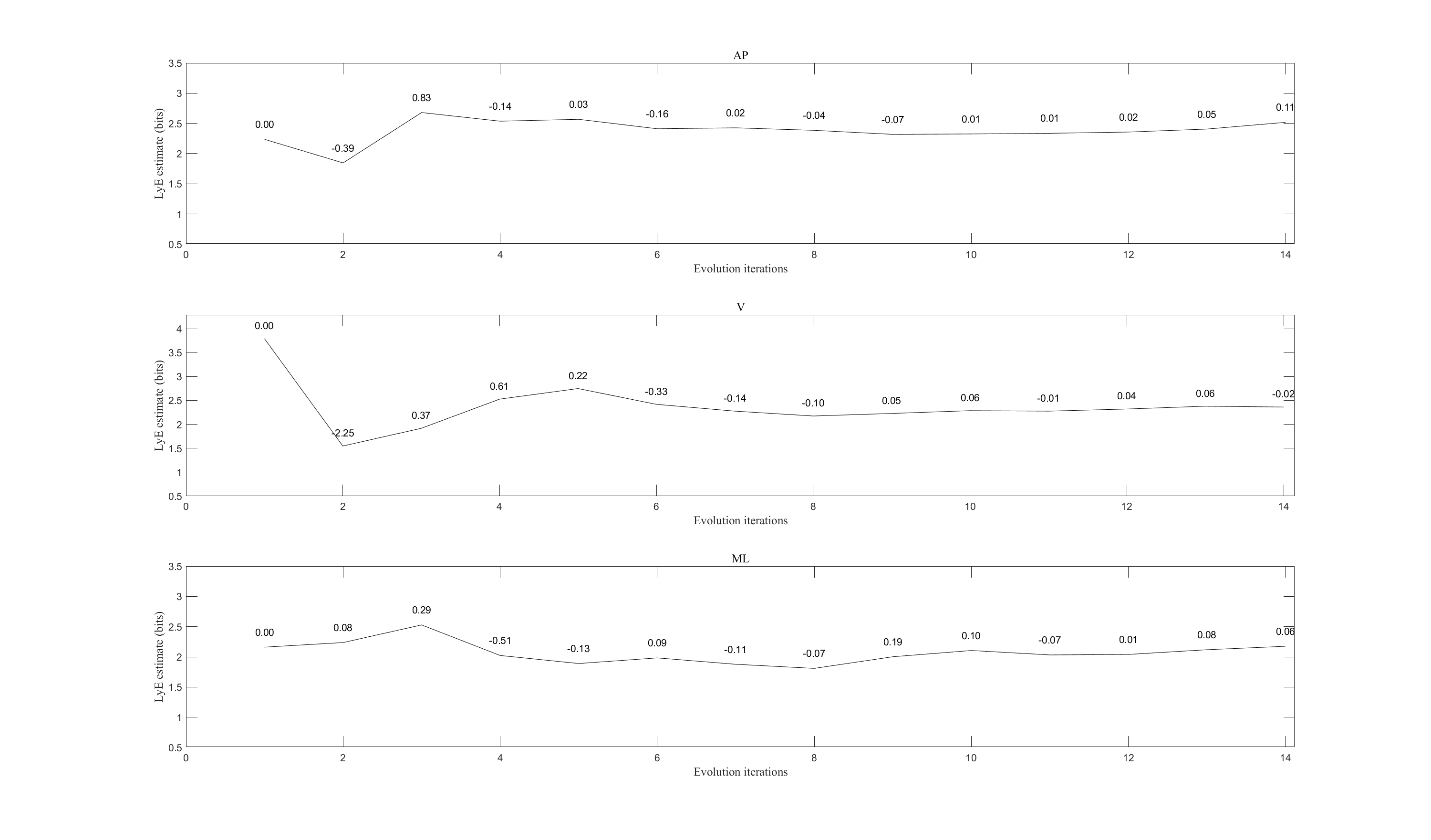

Supplement: Supplementary file 2 — Supplementary Information. [file 41598_2020_79584_MOESM2_ESM.zip › Participant17_trial1.png]

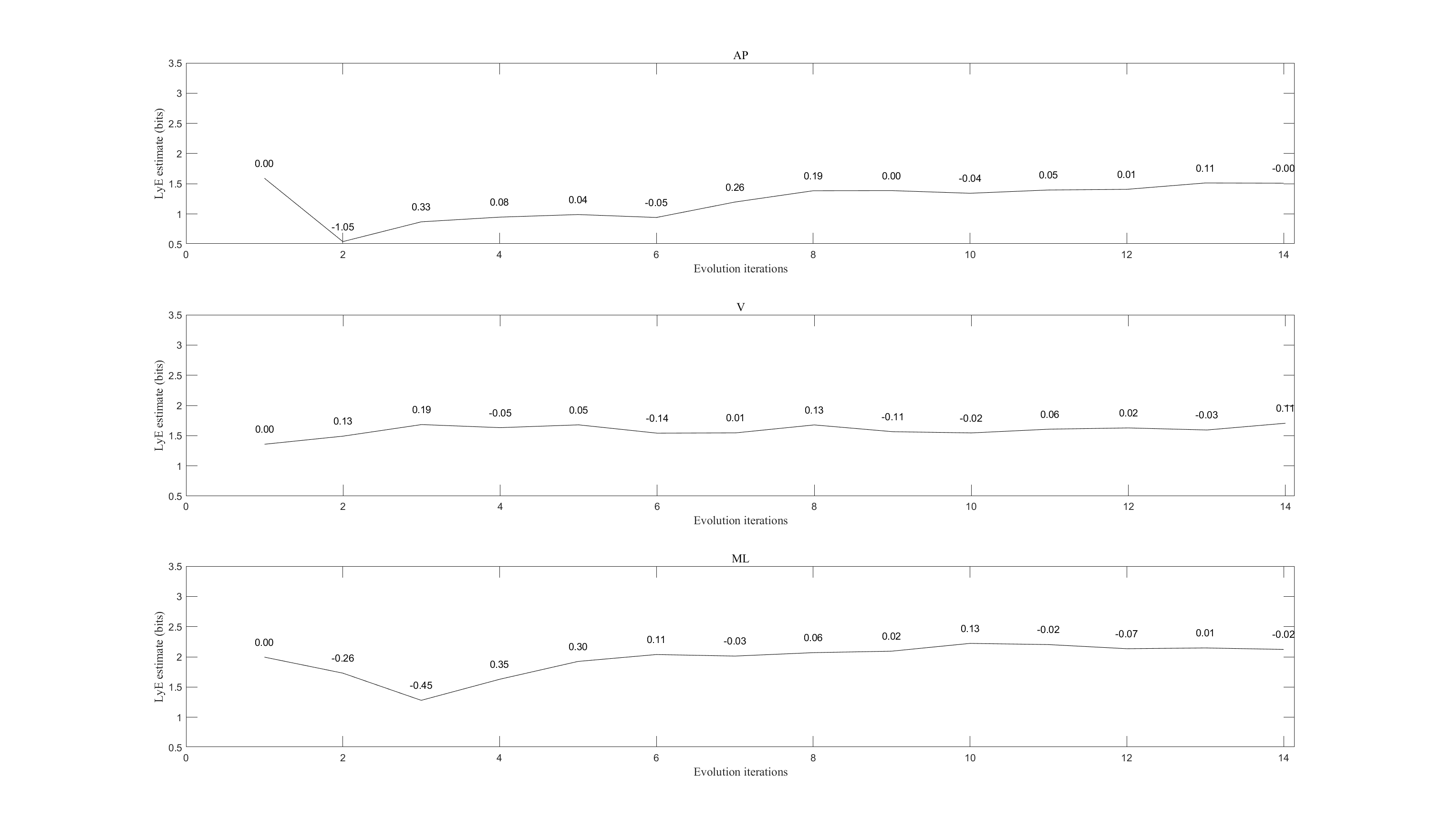

Supplement: Supplementary file 2 — Supplementary Information. [file 41598_2020_79584_MOESM2_ESM.zip › Participant17_trial10.png]

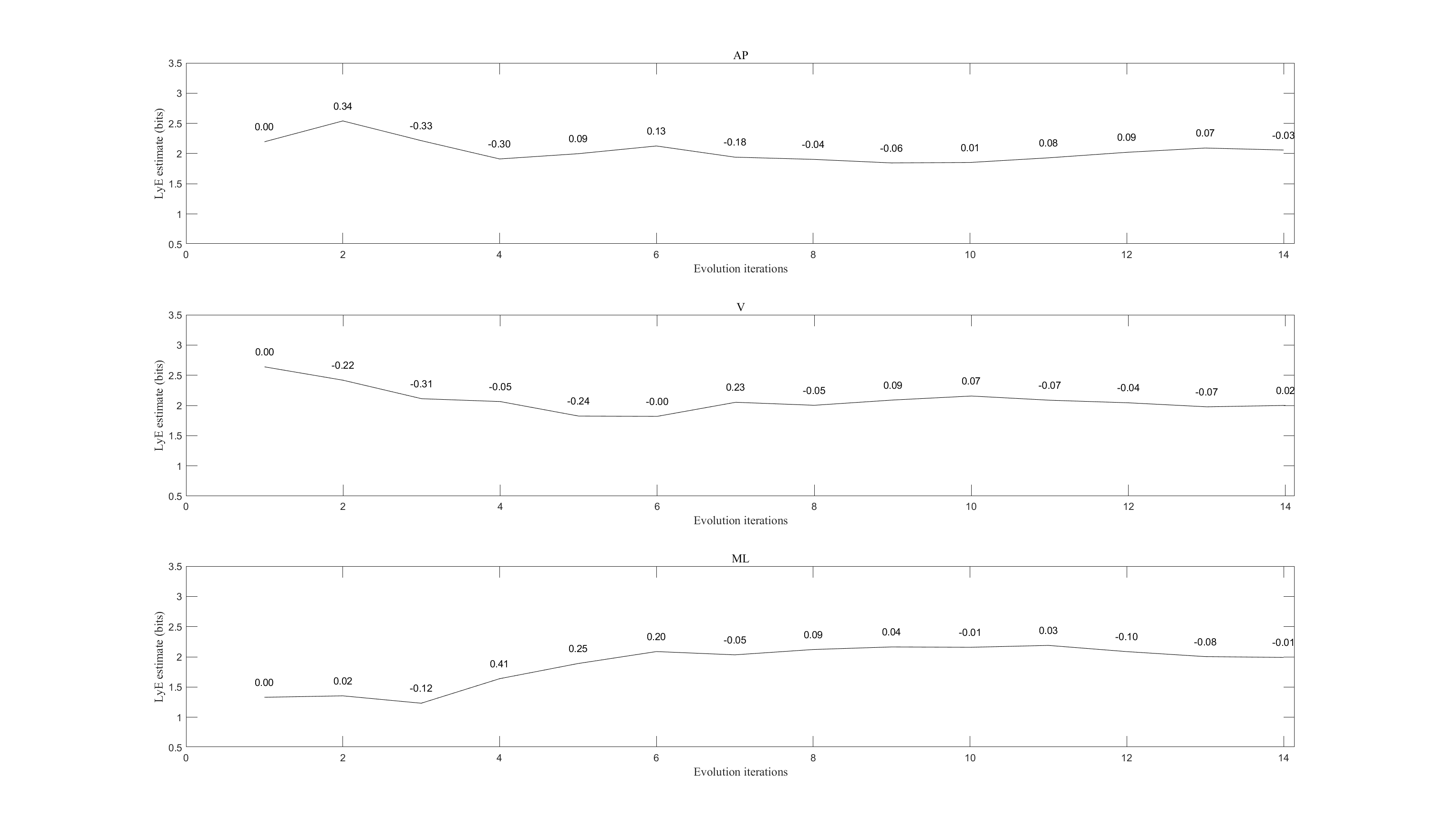

Supplement: Supplementary file 2 — Supplementary Information. [file 41598_2020_79584_MOESM2_ESM.zip › Participant17_trial11.png]

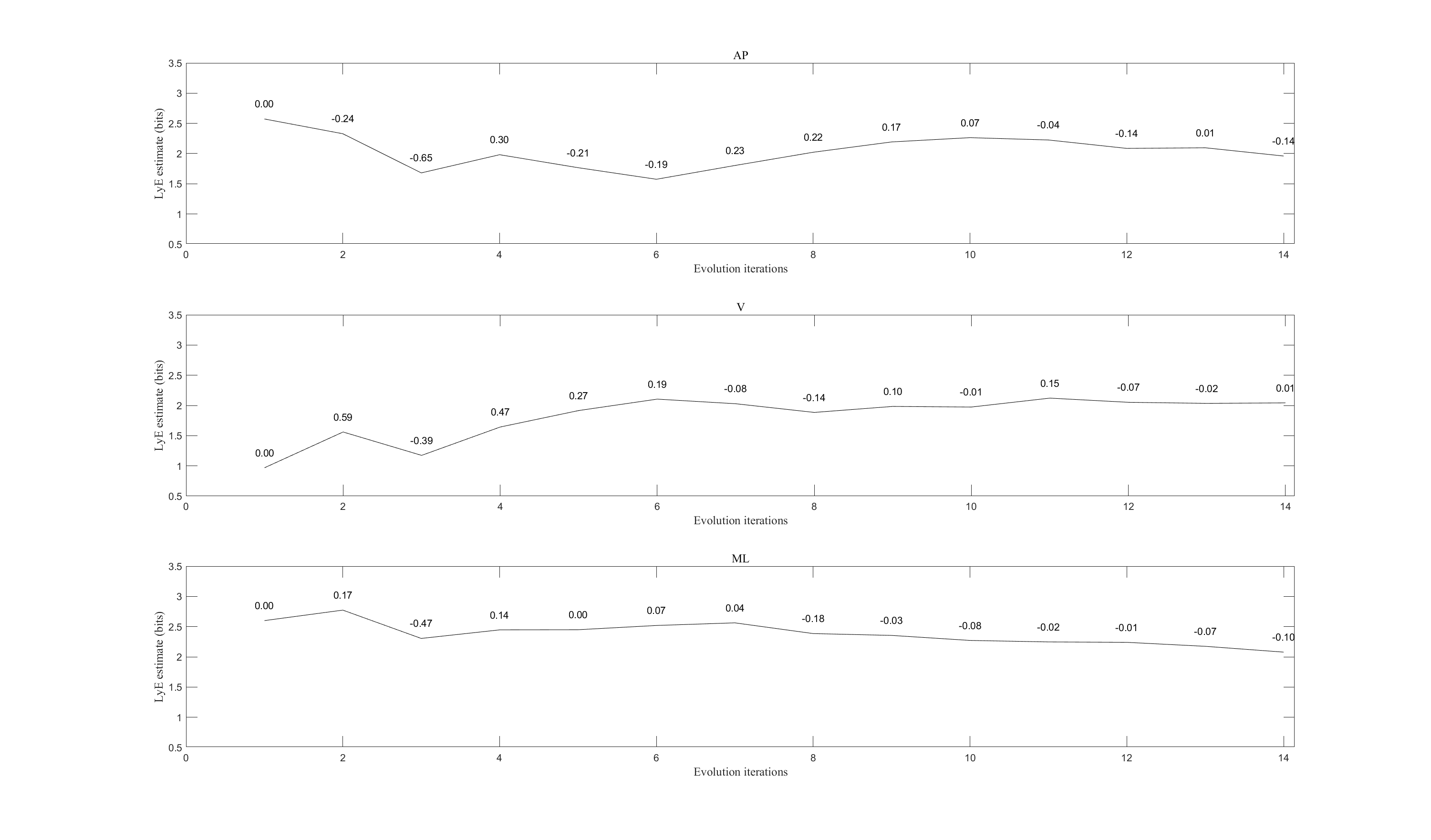

Supplement: Supplementary file 2 — Supplementary Information. [file 41598_2020_79584_MOESM2_ESM.zip › Participant17_trial12.png]
